# CMOS-compatible ferroelectric tunnel junctions integrate stochastic sampling and deterministic computing for image generation

**DOI:** 10.1038/s41467-026-72969-6

**Published:** 2026-05-08

**Authors:** Ryun-Han Koo, Jonghyun Ko, Wonjun Shin, Sangwoo Ryu, Jiseong Im, Sung-Ho Park, Joon Hwang, Minsuk Song, Youngchan Cho, Jangsaeng Kim, Gyuweon Jung, Daewoong Kwon, Jong-Ho Lee

**Affiliations:** 1 Department of Electrical and Computer Engineering and Inter-university Semiconductor Research Center, Seoul National University, Seoul, Republic of Korea; 2 Department of Semiconductor Convergence Engineering, Sungkyunkwan University, Suwon, Republic of Korea; 3 SK Hynix Inc, Icheon, Republic of Korea; 4 Department of Nanoscale Semiconductor Engineering, Hanyang University, Seoul, Republic of Korea; 5 Department of Electronic Engineering, Sogang University, Seoul, Republic of Korea

**Keywords:** Electrical and electronic engineering, Electronic devices

## Abstract

Recent progress in generative modeling has intensified the need for compact, energy-efficient hardware platforms. Yet, implementing image generation directly in hardware remains challenging due to the conflicting requirements of stochastic latent space sampling and deterministic decoding. Here, we show a unified hardware framework based on hafnium-oxide ferroelectric tunnel junctions (FTJs) that intrinsically support both functionalities within a single device array. Leveraging the CMOS- and VLSI-compatible fabrication of hafnia ferroelectrics, we realize dual-mode operation: random telegraph noise generation for controllable stochastic sampling, and high-fidelity vector–matrix multiplication enabled by non-volatile multi-level conductance states. Voltage and sampling-time tuning provide fine control over randomness and reliability, enabling high-quality image generation for tasks such as handwritten digit synthesis (MNIST) and high-resolution facial image generation (CelebA). Circuit-level demonstrations confirm stable performance over 10^5^ cycles, surpassing prior hardware-based approaches and illustrating a viable route toward scalable, on-chip generative AI accelerators.

## Abstract (web summary)

AI image generation is limited by conflicting needs for stochastic sampling and deterministic decoding. Koo et al. present a ferroelectric tunnel junction array using controllable random telegraph noise and nonvolatile multilevel conductance to unify both functions, enabling high-quality digit and facial image synthesis.

## Introduction

In recent years, deep learning has evolved beyond conventional tasks such as image classification, with image generation emerging as a pivotal capability in domains including autonomous systems, media synthesis, and synthetic data augmentation^1–3^. Unlike classification, which is predominantly deterministic, generative models require stochastic sampling from a latent space to yield diverse and realistic outputs. This intrinsic randomness poses substantial challenges for direct hardware implementation, particularly in reconciling the conflicting demands of stochastic sampling and deterministic decoding within a compact and monolithically integrated platform^4–7^. As a result, prior efforts have largely depended on hybrid architectures or off-chip software-based stochastic modules, constraining system scalability, energy efficiency, and integration density^5–14^. Moreover, in mature CMOS-based technologies, separate studies have been proposed to perform each role, implementing stochastic functionality^8^ and handling deterministic models^11,12^.

Hafnia-based ferroelectrics have emerged as a compelling candidate for hardware-based computing owing to their integration compatibility and material properties. These ferroelectric thin films can be synthesized via atomic-layer deposition (ALD), a wafer-scale, low-temperature process that is inherently compatible with state-of-the-art silicon complementary metal-oxide-semiconductor (CMOS) technologies^15–18^. The ALD technique affords sub-nanometer precision in controlling film thickness and stoichiometry, enabling engineered defect densities and spatial distributions. This precise defect control, coupled with post-deposition annealing optimization, allows fine-tuning of trap depth and location within the hafnia, directly modulating random telegraph noise (RTN) amplitude and time constants. Such control is critical for ensuring reproducible stochastic behavior across large-scale device arrays. Furthermore, ALD’s inherent capability for conformal three-dimensional growth facilitates seamless incorporation into high-density, 4*F*^2^-scalable device architectures^19–22^.

Beyond their integration and defect-engineering advantages, hafnia-based ferroelectric tunnel junctions (FTJs) intrinsically support both stochastic and deterministic functionalities. RTN generated by electron trapping and detrapping at engineered defect sites can be exploited for controllable random sampling in generative models, with bias voltage tuning providing direct control over sampling statistics. Concurrently, FTJs exhibit stable, non-volatile, and multi-level ferroelectric switching, enabling energy-efficient and deterministic vector–matrix multiplication (VMM) operations essential for neural inference and decoding. This voltage-programmable dual-mode capability permits seamless switching between stochastic and deterministic modes within a single array, eliminating the need for separate hardware blocks.

Several recent hardware approaches have targeted generative models but have addressed only part of this dual requirement^5–13^ (Table 1). Bi_2_O_2_Se-based noise generators have shown controllable physical noise for generative adversarial networks (GANs) but relied on software-based decoding and lacked CMOS process compatibility. ReRAM-based accelerators have demonstrated efficient GAN training speedup, yet have used only virtual noise inputs. Analog RRAM arrays have exploited intrinsic read/write noise for diversity enhancement, but the absence of integrated decoding has limited scalability. While recent studies leverage device noise to realize continuous weight distributions for classification, our work focuses on hardware-native generation by using RTN for latent sampling and FTJ-based VMM for decoding within a single array^23,24^.

**Table 1:** Comparison of our work with previously reported hardware generative models

Work	Latent sampling	Decoder mapping	Device type	CMOS compatibility	Array size	Task	Implementation	Cell/circuit area	Speed*	Power**	TOPS/W
This work	O	O	Both FTJ	O	400	MNIST, CelebA	Circuit-level	2 × 2 µm^2^	36 ms	282 µW (FTJ-only)	106 (FTJ & ADC)
^5^	O	-	Bi2O2Se	X	1 (Single Device)	MNIST	Device-level	10 × 10 −40 × 40 µm^2^	10 ms	98.1–337 µW	-
^6^	O	-	ReRAM	X	1 (Single Device)	TRNG-only	Circuit-level		50–250 ms	1.23– 862 µW	-
^7^	O	-	ReRAM	X	1 (Single Device)	TRNG-only	Circuit-level	2.5 × 2.5 µm2	100 ms	1.47 mW	-
^8^	-	O	ReRAM	O	Only Simulation	MNIST	Simulation-level	-	-	-	4.6
^9^	-	O	1T-1R	O	1000	MNIST	Circuit-level	-	-	-	-
^10^	O	-	CMOS	O	-	TRNG	Circuit-level	0.01 mm^2^	11.6–312 ns	8.33–523 µW	-
^11^	O	-	CMOS	O	-	TRNG	Circuit-level	0.0023 mm^2^	100 ps	1.2 mW	-
^12^	-	O	CMOS	O	-	Neural Networks	Commercial	-	-	-	2.3
^13^	O	-	MTJ	O	1	P-bit	Circuit-level	0.15 × 130 µm^2^	695 ms	20–100 µW	-

*Sampling time per bit.

**Normalized for 30 fps Human Face Image (128 × 128) Generation.

Tera operations per second (TOPS).

Leveraging the integration and defect-engineering strengths of hafnia FTJs, we present an integrated framework for hardware-native image generation in which the same FTJ array performs both stochastic latent vector sampling and deterministic decoding. By exploiting voltage-tunable RTN, our devices generate latent vectors with controlled mean and variance, closely matching target distributions from software-trained models. The same devices, under different biasing, enable reliable VMM decoding through multi-level ferroelectric switching. It enables balanced and diverse image synthesis, demonstrated for both handwritten digit generation (MNIST) and high-resolution facial synthesis (CelebA)^25^. In MNIST, optimal biasing conditions yield uniform class coverage without mode collapse, while in CelebA, a wide range of facial attributes, such as pose, hairstyle, and complexion, are faithfully reproduced. Circuit-level validation confirms robust operation over 10^5^ cycles and scalability to wafer-scale arrays. This dual-mode FTJ platform thus addresses the longstanding challenge of co-implementing diversity and determinism in hardware-based generative models, offering an energy- and area-efficient, CMOS-compatible pathway toward next-generation neuromorphic and AI accelerators. Supplementary Fig. 1 shows the schematic of FTJ operation, which can be configured to switch between stochastic and deterministic modes as needed for the image generation task.

## Results

### Device characteristics

**Fig. 1:**
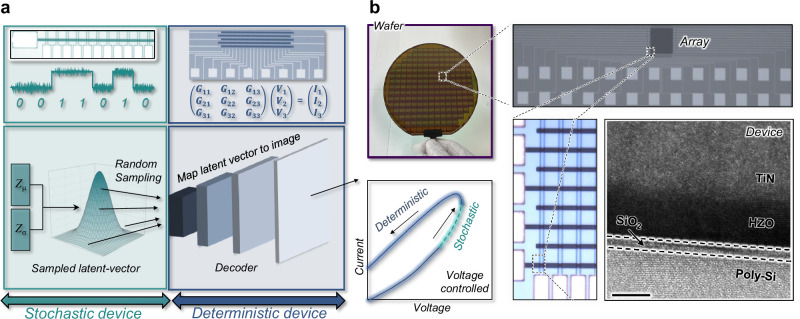
Overview of the FTJ-based hardware VAE system, including its schematic design, structural details, and TEM image. **a** A schematic diagram illustrating the process of generating synthetic images using the proposed FTJ-based hardware VAE. The VAE network consists of two parts: stochastic latent space sampling using an electrical noise source and deterministic decoding through VMM. **b** An optical microscopy image showing the overall wafer and a 20 × 20 NOR FTJ array, and a low-resolution cross-sectional TEM image of the FTJ, confirming the overall gate stack structure (scale bar, 5 nm in the TEM image).

Figure 1a shows a schematic diagram of the proposed FTJ-based hardware variational autoencoder (VAE). The VAE is divided into two functional blocks^26–28^. The first block performs random sampling in the latent space to generate a latent vector through a stochastic electrical noise source, which requires a device that exhibits inherent randomness. The second block decodes the sampled latent vector into an image through a cascade of VMM^26^. This stage, therefore, demands a non-volatile memory device that operates deterministically and reliably. Because these two requirements (stochasticity for sampling and determinism for VMM) are intrinsically contradictory, earlier studies either implemented only the sampling^5^, only the decoding^6^, or used separate devices for each function^7^. The off-chip integration incurs penalties in area, wiring, power, and latency due to complex interconnections and synchronization overhead.

Our FTJ-based circuit system overcomes this limitation by exploiting the voltage-dependent dual nature of FTJs. Detailed electrical analysis reveals distinct stochastic and deterministic operating regions resulting from dominant trap states in the tunnel barrier. By adjusting the read voltage, a single FTJ array can switch between stochastic sampling and deterministic VMM, enabling a unified, CMOS-compatible platform for hardware-based image generation. In practice, we partition the roles on the same array for inference. A small sampler region uses the RTN window near 3.2 V, and the rest of the array executes VMM at 2 V, so no weight reprogramming is required. Figure 1b shows an optical micrograph of the processed 6-inch wafer containing the FTJ arrays used in this study. The magnified view (middle inset) highlights a 20 × 20 NOR-type FTJ array, in which the top-electrode word-lines (WLs) are formed by Ti/TiN/Al/TiN (30/30/300/30 nm) interconnects, and the *n*⁺-poly-Si bit-lines (BLs) run orthogonally underneath. Each WL/BL intersection contains a 2 µm × 2 µm FTJ, comprising a 7-nm ferroelectric HZO layer and a 1.2-nm SiO_2_ tunnel barrier. The array, therefore, integrates 400 FTJs. To stabilize the ferroelectric orthorhombic (*o*-phase) of HZO, TiN was sputtered onto the HZO layer, followed by rapid thermal annealing (RTA) at 700 °C for 30 s in N_2_ ambient. The cross-sectional TEM image (right inset in Fig. 1b) confirms the intended TiN/HZO/SiO_2_/*n*⁺-poly-Si stack, validating the structural integrity of the fabricated devices. Further details of the fabrication process are provided in Supplementary Fig. 2 and the Methods section.

**Fig. 2:**
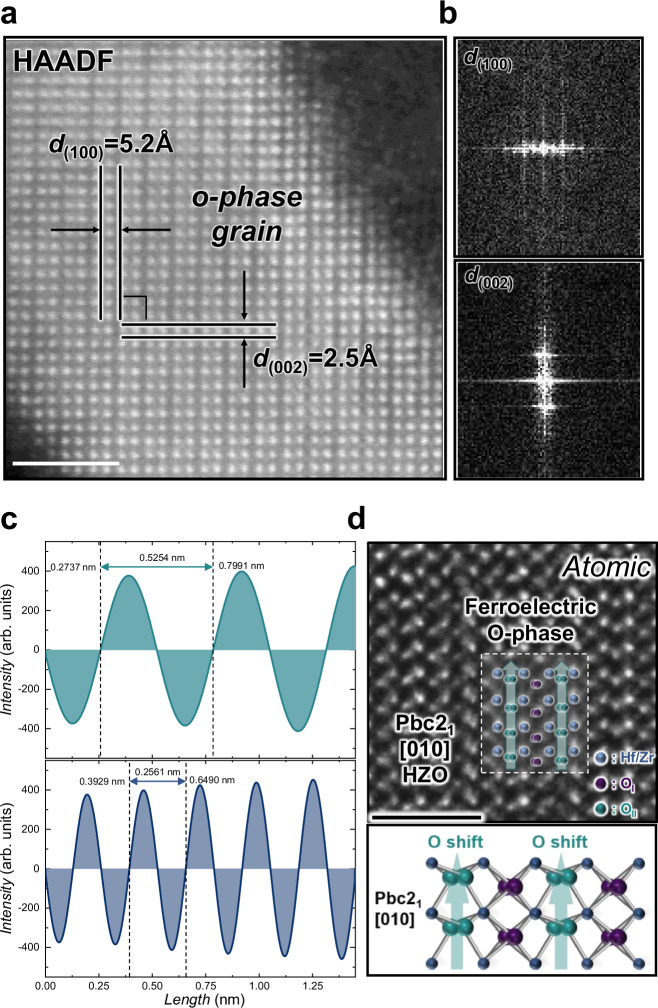
Atomic-resolution HAADF-TEM analysis of the ferroelectric *o*-phase structure in HZO. **a** HAADF-TEM image clearly displaying the atomic arrangement of the *o*-phase grain, showing lattice spacings of *d*_(100)_ = 5.2 Å and *d*_(002)_ = 2.5 Å (scale bar, 2 nm). **b** Corresponding Fast Fourier Transform (FFT) patterns confirming crystal orientations along the (100) and (002) planes. **c** Intensity profiles extracted from the HAADF image, quantitatively demonstrating the periodicity corresponding to the measured lattice distances. **d** Magnified atomic-resolution HAADF image of the *o*-phase structure, highlighting atomic shifts within the Pbc2_1_ space group and schematically illustrating the atomic displacement associated with ferroelectricity (scale bar, 1 nm).

The atomic-resolution high-angle annular dark-field transmission electron microscopy (HAADF-TEM) images presented in Fig. 2 were acquired using an FEI Themis Z microscope operating at 300 kV, equipped with spherical aberration correction and employing the negative spherical aberration imaging (NCSI) technique^29–31^. This advanced imaging method utilized a negative spherical aberration coefficient (Cs = −15 µm) and optimized defocus settings, enabling exceptional visualization of both heavy (Hf-Zr) and lighter (O) atomic columns simultaneously. Such precise imaging conditions were critical in clearly resolving subtle atomic displacements that underpin the ferroelectric properties of HZO. Details of the TEM sample preparation are provided in the Methods section. As shown in Fig. 2a, the atomic arrangement of the orthorhombic-phase (o-phase) grain of HZO is clearly revealed, with lattice spacings precisely measured as *d*_(100)_ = 5.2 Å and *d*_(002)_ = 2.5 Å. The corresponding Fast Fourier Transform (FFT) patterns in Fig. 2b further confirm the crystallographic orientations along the (100) and (002) planes, affirming the highly ordered crystalline structure of the *o*-phase^31^. Quantitative intensity profiles extracted along these crystallographic directions, shown in Fig. 2c, clearly demonstrate the periodicity consistent with the measured lattice distances, providing robust confirmation of structural uniformity at the atomic scale. A magnified atomic-resolution image (Fig. 2d) highlights explicit atomic displacements within the Pbc2_1_ space group. In this image, the displacement of O_II_ sites along the crystallographic b-axis is distinctly visible, whereas O_I_ sites remain centrally aligned, clearly indicating a break in centrosymmetry essential to the ferroelectric polarization in HZO. The atomic arrangement visualized in the HAADF image closely aligns with the expected atomic lattice model for the Pbc2₁ space group, as illustrated by the superimposed schematic atomic model. In addition, the crystalline phases of HZO films before and after TiN capping and annealing were analyzed by GIXRD, as shown in Supplementary Fig. 3. Collectively, these observations robustly validate the structural integrity and precise atomic arrangement of the ferroelectric o-phase in the HZO material^29^.

**Fig. 3:**
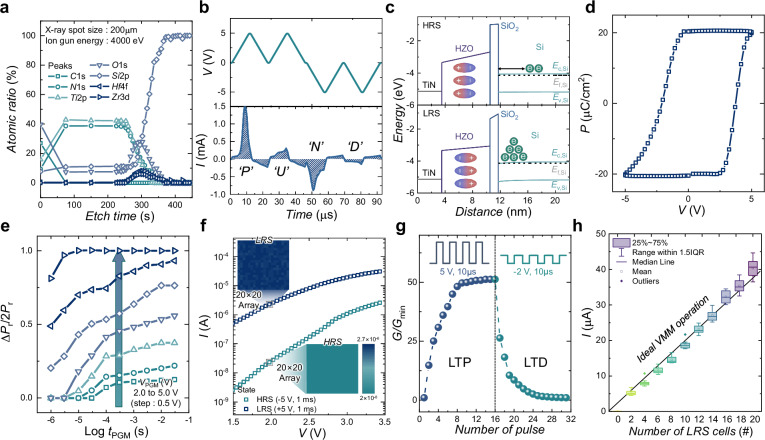
Overview of the structural, electrical, and functional characteristics of the FTJ. **a** Depth XPS analysis results, confirming the TiN–HZO–SiO_2_–*n*^+^ poly-Si structure. **b** PUND pulse waveforms (top) and *I* curves (bottom) showing polarization switching in the HZO layer. **c** Band diagrams of FTJ under different polarization states. HRS (top) and LRS (bottom). **d** P–V curve showing stable remnant polarization (2*P*_r_ > 40 μC/cm^2^) in an HZO layer. **e** Δ*P*/2*P*_r_ versus *t*_*P*GM_ as a parameter of *V*_PGM_. **f** I–V curves of the FTJ in LRS and HRS states. The inset shows the conductance map of the 20 × 20 NOR FTJ array, with a clear TER ratio (>70), indicating distinct LRS and HRS states. **g** LTP-LTD characteristics of the FTJ. **h** VMM results from the 20 × 20 NOR FTJ array, demonstrating highly uniform VMM operation.

Figure 3a shows the depth profile of the FTJ obtained from XPS analysis. This analysis was performed using a 200 μm X-ray spot size and an ion gun energy of 4000 eV. As the etching time progresses, the sequential increase in Ti2p/N1s, Hf4f/Zr3d, and Si2p/O1s components confirms the formation of the intended TiN–HZO–SiO₂–*n*⁺ poly-Si stack. Figure 3b shows the pulse waveforms applied for the positive-up-negative-down (PUND) measurement (top) and the resulting current (*I*) curves (bottom). The differences in current between the positive (P) and up (U) pulses, as well as between the negative (N) and down (D) pulses, indicate the displacement current caused by polarization switching, confirming the successful induction of ferroelectricity in the HZO layer^16,32^.

Figure 3c shows the band diagrams of the FTJ under different polarization states. The upper panel of Fig. 3c illustrates the case when a negative pulse is applied to the TiN top electrode, causing the positive charge of the ferroelectric dipole to point toward the TiN. This induces a negative electric field toward the Si electrode, increasing the tunneling resistance due to depletion in the bottom electrode (high resistive state, HRS). Conversely, the lower panel of Fig. 3c shows the case where a positive pulse is applied to the TiN top electrode, aligning the negative charge of the dipole toward the TiN. This induces a positive electric field toward the Si electrode, reducing the tunneling resistance (low resistive state, LRS). These results demonstrate the capability of the FTJ to function as a non-volatile memory device with multiple resistance states by controlling the dipole orientation^33–35^.

Figure 3d shows the polarization-voltage (*P*–*V*) curve obtained from the PUND measurements, in which the remnant polarization (*P*_r_) of the HZO layer in the fabricated FTJ is measured to be 2Pᵣ > 40 μC/cm², demonstrating stable polarization switching. Polarization endurance characteristics are provided in Supplementary Fig. 4. Figure 3e shows the polarization switching ratio (Δ*P*/2*P*_r_) as a function of pulse width (*t*_PGM_) and pulse amplitude (*V*_PGM_). As *t*_PGM_ and *V*_PGM_ increase, more polarization switching occurs, indicating partial polarization. Figure 3f shows *I*–*V* curves of the fabricated FTJ in both the LRS and HRS states. The HRS is induced by applying a −5 V, 1 ms pulse, while the LRS is induced by applying a 5 V, 1 ms pulse. The inset of Fig. 3f shows the conductance map of the entire 20 × 20 FTJ array programmed in both the LRS and HRS states. Although a slight variation in the LRS is observed, the device shows a sufficient tunneling electroresistance (TER) ratio (>70), ensuring a clear distinction between the two states. For the FTJ to function as a hardware synaptic device capable of implementing the decoder function, it must not only exhibit two distinct states (LRS and HRS) but also demonstrate gradual conductance modulation in response tng-term potentiatioo continuous pulse inputs, enabling long-term potentiation (LTP) and long-term depression (LTD) characteristics. Figure 3g shows the LTP-LTD characteristics of the fabricated FTJ. LTP is achieved using continuous 5 V, 10 μs pulses, and LTD is achieved using continuous −2 V, 10 μs pulses, confirming the potential of the FTJ as a synaptic device^36–38^. The update curves exhibit nonlinearity with *β*_LTP_ = 4.9 and *β*_LTD_ = 8.2. Based on these measured nonlinearities, the decoder uses a device-informed 4-bit (16-state) conductance partition, and weight mapping assigns targets according to the *β*-dependent update characteristics. Results of 50 consecutive LTP/LTD cycles are shown in Supplementary Fig. 5. Supplementary Fig. 6 shows the mean LTP and LTD trajectories across 50 devices as lines, with standard-deviation error bars at each pulse index. In the subsequent simulations, the decoder weights reflect both effects. The nonlinearity is incorporated through the *β*-based 16-bin target mapping, and the level-to-level variation is injected using the measured spreads from Supplementary Fig. 6. Polarization retention characteristics across devices are provided in Supplementary Fig. 7. Figure 3h demonstrates VMM results obtained from the fabricated 20 × 20 NOR-type FTJ array by randomly programming cells to HRS/LRS and stepwise increasing the number of activated (LRS) devices under a 2 V read bias, showing that the column-summed current scales linearly with the activated-device count and thus confirming array-level VMM linearity in the deterministic regime. For decoder VMM, we operate at a low read voltage of 2 V, 10 ms to stay in the RTN-free regime and to reduce power and IR drop while preserving linear accumulation. The uniform conductance states of the FTJs enable accurate and consistent VMM operation, confirming the device’s suitability for hardware implementation of neural network decoder functions. As the number of activated FTJs increases in increments of two, the summed current increases linearly, reflecting precise and predictable accumulation behavior essential for VMM operations^36–38^.

### Noise behavior and stochastic sampling

**Fig. 4:**
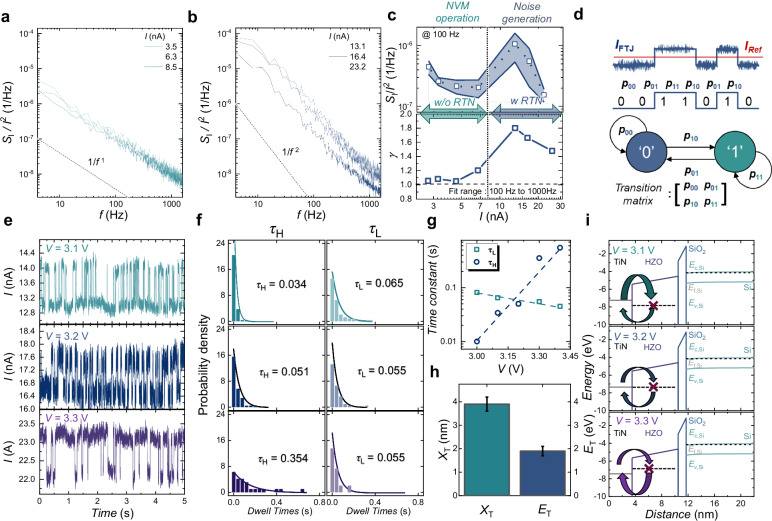
Analysis of RTN behavior and noise mechanisms in the FTJ with varying electrical conditions. **a** Relationship between *S*_I_/*I*^2^ and frequency for currents *I* < 10 nA, showing the dominance of 1/*f* noise. **b** Relationship between *S*_I_/*I*^2^ and frequency for currents *I* > 10 nA, exhibiting 1/*f*² noise indicative o*f* RTN presence. **c** Plots of *S*_I_/*I*^2^ versus current at 100 Hz (top) and γ versus current (bottom), highlighting a change in the noise mechanism around *I* = 10 nA. The value of γ is fitted in the 100–1000 Hz frequency range. **d** Illustration of generating a 0/1 sequence based on the FTJ current response, modeled as a Markov chain. **e** DC transient measurements at different voltages (*V* = 3.1 V, 3.2 V, 3.3 *V*), showing varying dominance of low and high-current states. **f** Histograms of time durations in high (*τ*_H_) and low (*τ*_L_) current states, fitted to Poisson distributions. **g** Relationship between extracted *τ*_H_ and *τ*_L_ as a function of *V*. **h** Extracted *X*_T_ and *E*_T_ of the dominant trap causing RTN in the FTJ. **i** Energy band diagrams of the FTJ under different applied voltages (*V* = 3.1 V, 3.2 V, and 3.3 V).

To analyze the stochastic characteristics of the FTJ, low-frequency noise (LFN) spectroscopy is employed. LFN spectroscopy is a non-destructive technique that transforms the current noise of electronic devices into the frequency domain, enabling the analysis of the power spectral density (PSD) variations with respect to frequency. This provides a deep understanding of the various physical parameters influencing current fluctuations^39–43^. The configuration for LFN and RTN measurements is shown in Supplementary Fig. 8. Figures 4a, b show the relationship between the normalized PSD (*S*_I_/*I*²) and frequency for different *I* values. The area of the measured device is 2 × 2 μm^2^, and the current *I* is adjusted according to the amplitude of the *V* applied to the TiN electrode. Interestingly, the dependency of *S*_I_/*I* ² on frequency changes with *I*. For currents below 10 nA, the PSD exhibits 1/*f* noise behavior (Fig. 4a)^44–46^, while for currents above 10 nA, the noise approaches 1/*f*^2^ behavior (Fig. 4b), indicating the presence of RTN. Reproducibility of low-current 1/*f* noise and high-current 1/*f*^2^ noise across 10 devices is shown in Supplementary Figs. 9 and 10, respectively. Figure 4c shows the relationship between *S*_I_/*I*² and current at 100 Hz (top) and the relationship between the frequency exponent (γ = -∂ln(*S*_I_/*I*²)/ ∂ln(*f*)) and current (bottom). We extracted the exponent *γ* by fitting log(*S*_I_/*I*²) versus log *f* within 100–1000 Hz to minimize low-frequency drift and measurement noise. Both *S*_I_/*I*² and γ increase sharply beyond *I* = 10 nA, suggesting a change in the noise generation mechanism and the presence of RTN^47–49^. Consequently, the region where *I* < 10 nA, exhibiting lower *S*_I_/*I*^2^ and no RTN (Supplementary Fig. 9), can be used for non-volatile memory operations and decoder implementation, while the region where *I* > 10 nA, characterized by higher noise and the presence of RTN, can be utilized for latent vector sampling (Supplementary Fig. 10).

Supplementary Fig. 11a and Supplementary Fig. 11b show the DC transient measurement results for *V* = 2.9 V (*I* = 8.5 nA, Supplementary Fig. 11a) and *V* = 3.2 V (*I* = 17 nA, Supplementary Fig. 11b). The measurements are sampled at 3200 Hz for 5 seconds. Unlike at *V* = 2.9 V, a distinct two-level RTN is observed at *V* = 3.2 V. Supplementary Fig. 11c and Supplementary Fig. 11d show the time-lag plot of the DC transient measurements. At *V* = 2.9 V (Supplementary Fig. 11c), where no RTN is present, the plot shows a single, concentrated cluster. In contrast, at *V* = 3.2 V (Supplementary Fig. 11d), where RTN is observed, four discrete clusters are presented, corresponding to transitions between two different states (high and low-current levels). Supplementary Fig. 11e and Supplementary Fig. 11f show histograms for *V* = 2.9 V and *V* = 3.2 V, respectively. When no RTN is present, the current distribution follows a single Gaussian curve, whereas with RTN, the current is distributed into two Gaussian curves with distinct means, corresponding to the two current states. Using the noise in the current, we generate a random sequence of ‘0’s (*I* < *I*_Ref_) and ‘1’s (*I* > *I*_Ref_) by comparing the reference current (*I*_Ref_) to the instantaneous measured *I*. Figure 4d shows the process of generating a 0/1 sequence based on the current response of FTJ. This process can be modeled as a Markov chain^50,51^, where transitions between the two states (0 and 1) follow a transition matrix with transition probabilities. In real circuits, noise in *I*_Ref_ and device variation may introduce inconsistencies in distinguishing the two states. When RTN is absent, the current distribution follows a single Gaussian distribution (Supplementary Fig. 11e), making it difficult to distinguish between the two states. On the other hand, when RTN is present, the current distribution clearly separates into two distinct states (Supplementary Fig. 11f), making the distinction robust even with variations in *I*_Ref_. Supplementary Fig. 12a shows how noise in *I*_Ref_ affects the proportion of the 0 and 1 states. Supplementary Fig. 12b shows the bit-error ratio (BER) of ‘0’s to ‘1’s as a function of *I*_Ref_ variation. In the absence of RTN, most current values cluster around a single mean, causing significant shifts in the 0/1 ratio as *I*_Ref_ changes. However, in the presence of RTN, the two states are clearly distinguishable, and the 0/1 sequence is generated with uniform robustness against noise and variations.

In the region where RTN is present, we conducted DC transient measurements at different voltages (*V* = 3.1, *V* = 3.2, and *V* = 3.3 V) to observe the effect of *V* on RTN behavior (Fig. 4e). At *V* = 3.1 V, the low-current state dominates, while at *V* = 3.2 V, the low and high-current states occur with similar frequency. At *V* = 3.3 V, the high-current state becomes more prevalent. Figure 4f shows histograms of the time spent in the high-current and low-current states, respectively, based on the DC transient measurements. The solid lines represent fits to Poisson distributions, with the fitted *τ* values shown in the inset. Both the high-current and low-current time constants follow Poisson distributions, and as *V* increases, the τ for the high-current state (*τ*_H_) increases, while the *τ* for the low-current state (*τ*_L_) decreases. Figure 4g shows the relationship between the extracted time constant, *τ*_H_ and *τ*_L_, and the applied voltage, highlighting that RTN behavior in FTJs originates from the interaction between dominant trap sites and the top electrode. To identify the trap energy level (*E*_T_) and physical depth (*X*_T_) of the trap responsible for RTN, we analyze the fractional trap occupancy using the following equation^52,53^:

1 $${\tau }_{{{\rm{H}}}}/{\tau }_{{{\rm{L}}}}={e}^{\frac{{E}_{{{\rm{T}}}}-{E}_{{{\rm{F}}}}}{{k}_{{{\rm{B}}}}T}}$$

By differentiating both sides with respect to *V*, the following expression is obtained:

2 $$\frac{\partial }{\partial V}\left[{{\mathrm{ln}}}(\tau _{{{\rm{H}}}}/{\tau }_{{{\rm{L}}}})\right]\propto \frac{{X}_{{{\rm{T}}}}/{T}_{{{\rm{ox}}}}q}{{k}_{{{\rm{B}}}}T}$$

Here, *k*_B_ is the Boltzman constant, *T* is the absolute temperature, *T*_ox_ is the equivalent oxide thickness, and *q* is the elementary charge. This equation allows for extraction of *X*_T_ from the voltage dependency of RTN time constant. However, since this model assumes a single dielectric layer, modifications are necessary for FTJs, which consist of two dielectric layers (SiO_2_ and HZO) sandwiched between a TiN top electrode and an *n*^+^-Si bottom electrode. To account for the equivalent oxide thickness (EOT) of each layer, *X*_T_ is redefined as:

3 $${X}_{{{\rm{T}}}}=\left[1-\frac{{k}_{{{\rm{B}}}}T}{q}\frac{\partial {{\mathrm{ln}}}(\tau _{{{\rm{H}}}}/{\tau }_{{{\rm{L}}}})}{\partial V}\left(1+\frac{{T}_{{{\rm{SiO}}}2}}{{T}_{{{\rm{HZO}}}}}\frac{{\varepsilon }_{{{\rm{HZO}}}}}{{\varepsilon }_{{{\rm{SiO}}}2}}\right)\right]{T}_{{{\rm{HZO}}}}+{T}_{{{\rm{SiO}}}2}$$

Here, *T*_SiO2_ and *T*_HZO_ are the physical thicknesses of the SiO_2_ and HZO layer, while *ε*_SiO2_, and *ε*_HZO_ denote their respective permittivities. Additionally, the sign of $\frac{\partial {{\mathrm{ln}}}(\tau _{{{\rm{H}}}}/{\tau }_{{{\rm{L}}}})}{\partial V}$ provides insight into the electrode with which the trap interacts. A positive sign indicates interaction with the TiN top electrode, while a negative sign suggests interaction with the *n*^+^-Si bottom electrode. Based on Fig. 4g, the RTN source in the FTJ is attributed to a trap located 3.9 nm from the top TiN electrode, with an energy level 1.9 eV below the conduction band edge (*E*_C_) of the HZO layer. This conclusion is summarized in Fig. 4h. These extracted trap parameters align with typical characteristics introduced during the ALD and annealing processes of HZO, corroborating the validity of the analysis. Figure 4i depicts the energy band diagrams of the FTJ under different applied voltages. At *V* = 3.1 V, the Fermi level (*E*_F_) of TiN is higher than the *E*_T_, resulting in faster electron capture than emission, which prolongs the low-current state. At *V* = 3.2 V, *E*_F_ aligns closely with *E*_T_, leading to nearly equal capture and emission rates, and an approximately balanced occurrence of high and low-current states. At *V* = 3.3, *E*_F_ is lower than *E*_T_, leading to faster emission than capture, thereby prolonging the high-current state^52,53^. When the bias moves away from this alignment window (*V* = 3.1–3.3 V), the two rates become highly imbalanced and *τ*_L_ and *τ*_H_ diverge, the two-level signature collapses into a single preferred state, and the noise becomes 1/*f* due to Poole-Frenkel-assisted transport through a distributed ensemble of traps. This bias-dependent mechanism links the band alignment in Fig. 4i and the dwell-time trends in Fig. 4g to the observed change from RTN to 1/*f* noise. Note that RTN appears only in the mid bias window ~3.1–3.3 V where *τ*_H_ and *τ*_H_ are comparable. It is suppressed at or below 2.9 V and at or above 3.5 V. Therefore, decoder VMM is performed at a low read voltage of 2 V in the RTN-free regime to reduce power and IR drop while preserving linear accumulation. RTN is utilized ~3.2 V for stochastic latent sampling.

To further validate that the RTN characteristics originate from ALD-related traps and remain controllable across the wafer, we measured the HZO thickness and RTN uniformity over a 6-inch wafer (Supplementary Fig. 13). The thickness map and device-wise dwell-time statistics (Supplementary Fig. 14 and Supplementary Fig. 15) confirm uniform Poisson-type RTN behavior with minor device-to-device variation caused by small differences in trap depth and energy level. By iteratively adjusting the read bias in 1 mV steps according to the *τ*_L_/*τ*_H_ imbalance, the distributions were equalized across all devices (Supplementary Fig. 16 and Supplementary Fig. 17). This simple voltage-trimming method, readily implemented through word- or bit-line DAC control, enables parallel generation of uniform random bitstreams even with slight ALD-induced variability. Notably, the equalized read voltages fall within a narrow 3.25–3.30 V window (Supplementary Figs. 16 and 17), implying that the required tuning range is only on the order of tens of millivolts. Therefore, a compact limited-range 6–7-bit fine-trim bias DAC ( ≈ 1 mV LSB) is sufficient, and this trimming is required only for the RTN sampler region rather than the full VMM array.

The ability to control RTN behavior through voltage adjustments provides an effective mechanism for neural network-based image generation systems. Firstly, by modulating the applied voltage, FTJs not only enable the generation of RTN but also allow for its suppression, enabling seamless transitions between stochastic and deterministic operations. We further note that deterministic VMM is reliable in the 1/*f* regime, whereas 1/*f*^2^ RTN is not suitable for VMM and is used for stochastic sampling. The central limit theorem rationale and the Q–Q analysis supporting this distinction are provided in Supplementary Figs. 18–21, and Supplementary Note 1. In addition, when RTN exists, the high/low-current state ratio can be precisely tuned by adjusting the sampling time, facilitating controlled stochasticity for latent vector sampling. This dual controllability (voltage-dependent modulation of RTN existence and time-constant tuning) effectively addresses the challenge of balancing randomness and reliability in hardware-based image generation. This voltage-controlled RTN, which is combined with the non-volatile memory characteristics of FTJs, demonstrates significant potential for neuromorphic computing systems by supporting both stochastic and deterministic operations.

### Circuit-level random bitstream generation

**Fig. 5:**
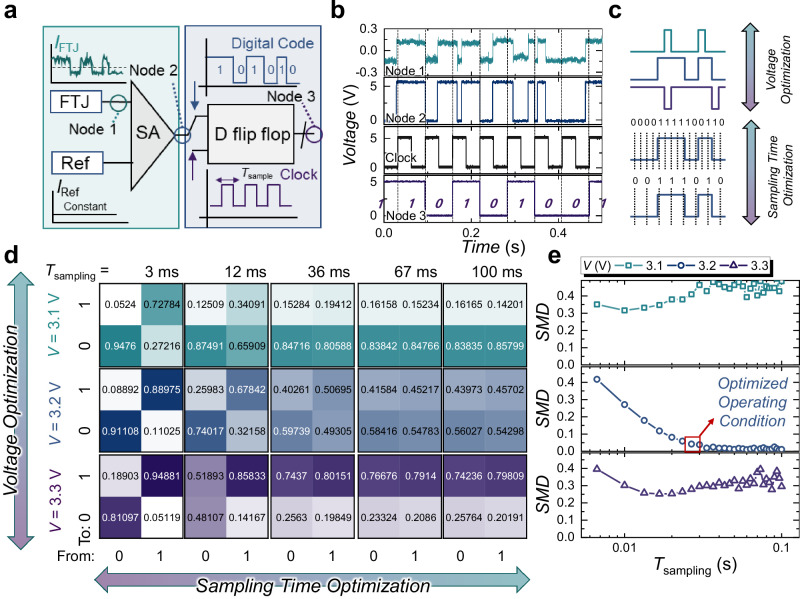
Circuit implementation and optimization of FTJ-based random number generation. **a** Schematic diagram of the FTJ-based circuit used to verify the output of a random source output. The circuit includes an FTJ, a sense amplifier, and a D flip-flop to generate a random bitstream. **b** Transient results of the FTJ-based circuit, showing how RTN is converted into a voltage signal (Node 1), digitalized (Node 2), and outputted as a random sequence (Node 3). **c** Optimized bitstream output at Node 3 by adjusting FTJ bias *V* and *T*_Sampling_. **d** Monte–Carlo transition matrix for state transitions in RTN, with transition probabilities P_00_, P_01_, P_10_, and P_11_, with a function of *V* and *T*_Sampling_. **e** SMD as a function of *T*_Sampling_ for different *V*, showing that *V* = 3.2 V and *T*_Sampling_ = 36 ms is the optimized operating condition.

Figure 5 shows the schematic of a circuit that integrates an FTJ and peripheral components to generate a random latent vector for hardware-based stochastic operations. The roles of each node are defined as follows: Node 1 represents the voltage output converted from the FTJ current via an *I*-to-*V* converter. Node 2 shows the digitized output from Node 1, obtained by comparing the FTJ voltage with a reference voltage using a voltage sense amplifier. The sense amplifier outputs a digital code 1 when the FTJ voltage is higher, and 0 otherwise. Node 3 captures the random sequence of 0 s and 1 s at constant intervals, sampled by a clock signal through a D flip-flop. Notably, the sampling time (*T*_Sampling_) can be adjusted by tuning the input clock of the D flip-flop. Figure 5b presents the transient response measured from the actual circuit shown in Fig. 5a, comprising the FTJ, voltage sense amplifier, and D flip-flop. These measurements are obtained using an oscilloscope and demonstrate the transformation of RTN into a random latent vector. The Node 1 voltage transient plot in Fig. 5b shows how the RTN-induced current is converted into a voltage signal that oscillates between two levels, reflecting the original FTJ current fluctuations. Node 2 shows the digitized voltage output from the voltage sense amplifier, indicating the comparison result between the FTJ voltage and the reference voltage. Finally, Node 3 displays the random bitstream output sampled by the D flip-flop at intervals determined by the clock signal. By tuning the FTJ bias voltage and *T*_Sampling_, the randomness of the bitstream at Node 3 can be optimized, as depicted in Fig. 5c. A custom PCB implementation of the RTN-based bitstream generator is shown in Supplementary Fig. 22. Note that the RTN-based sampler is implemented in hardware (Fig. 5a, b), whereas the decoder VMM is realized in hardware-informed software. In this implementation, weights are mapped to 4-bit (16-state) conductance levels derived from the measured LTP/LTD nonlinearities (*β*_LTP_ = 4.9, *β*_LTD_ = 8.2; Fig. 3g), and array-level non-idealities are incorporated by applying a multiplicative noise factor with variance σ extracted from the 20 × 20 array measurements at 2 V (Fig. 3h). Supplementary Note 2 provides a detailed explanation of the mapping strategy of the VAE decoder.

To visualize the randomness of the bitstream through *V* and *T*_Sampling_ adjustment, we modeled the state transitions in RTN using a Markov Chain transition matrix (MCTM). MCTM is suitable for analyzing random bitstreams by modeling the transition probability from the previous state to the next state through each element^50,51^. In RTN, we encode the low-current state as 0 and the high-current state as 1, with transition probabilities dependent on different *V* and *T*_Sampling_ conditions, as shown in Fig. 5d. *P*_00_ represents the probability that the next observation remains at 0 after observing state 0, and *P*_01_ is the probability of transitioning from state 0 to state 1. Similarly, *P*_10_ and *P*_11_ represent the transition probabilities from state 1 to states 0 and 1, respectively. The sum of *P*_00_ and *P*_01_ is 1, as is the sum of *P*_10_ and *P*_11_, because they account for all possible outcomes. When the *T*_sampling_ is too short, the system captures the FTJ signal before it has sufficient time to transition between states. This leads to repeated observation of the same state, causing *P*_00_ (remaining at 0) and *P*_11_ (remaining at 1) to approach 1, reducing the overall randomness. Ideally, all transition probabilities should converge to 0.5 to ensure maximum randomness. Our observations indicate that increasing *T*_Sampling_ brings the transition matrix closer to this ideal state. However, excessively long sampling periods compromise operational efficiency by delaying latent vector sampling, highlighting the importance of optimizing *T*_Sampling_ to balance randomness and latency.

Interestingly, as shown in Fig. 5d, even with sufficient *T*_Sampling_, we noted that for *V* = 3.1 V and *V* = 3.3 V, the inherent imbalance in the frequencies of 0 s and 1 s results in *P*_01_ and *P*_10_ values deviating from the ideal 0.5. To quantitatively evaluate the uniformity of the MCTM, we introduce a metric called squared matrix deviation (SMD), which measures how far each element of the matrix deviates from 0.5 (ideal random source). SMD is defined as:

4 $${SMD}=\sum {\left({P}_{{{\rm{ij}}}}-0.5\right)}^{2}$$

A smaller SMD value indicates a more ideal noise source, with all transition probabilities approaching 0.5. Figure 5e shows the relationship between SMD and *T*_Sampling_ for different voltage conditions. As *T*_Sampling_ increases, SMD generally decreases, indicating improved uniformity in the transition probabilities. However, at *V* = 3.1 V and *V* = 3.3 V, SMD saturates above 0.2, even in a long sampling period, reflecting an inherent imbalance in the RTN behavior. This imbalance is consistent with the observation in Fig. 4g, where the high and low states exhibit unequal frequencies. In contrast, at *V* = 3.2 V, where all the transition probabilities are nearly equal, SMD approaches 0 with sufficiently long *T*_Sampling_ (>36 ms). Based on this analysis, we identify the optimal conditions for latent vector sampling as *V* = 3.2 V and *T*_Sampling_ = 36 ms. These conditions minimize SMD, ensuring uniform randomness in the MCTM while minimizing sampling latency. We validated randomness using NIST SP 800-22 on raw and XOR-processed RTN bitstreams, which are provided in the Supplementary Fig. 23 and Supplementary Note 3.

### Image generation performance on handwritten digits

Based on the circuit presented in Fig. 5a, we evaluated the feasibility of generating images resembling the MNIST dataset using the VAE architecture by optimizing both the applied *V* and the *T*_Sampling_. To generate MNIST images, the encoder network maps an input MNIST image (784 pixels) through two hidden layers to a 2-dimensional latent space, following a structure of 784-512-256-2. The decoder then takes the 2-dimensional latent space as its input and reconstructs an output image with the same dimensions as the input, following a structure of 2-256-512-784. To generate latent vectors, we sampled the bitstream of 0 s and 1 s 20 times using the Node 3 output from the circuit shown in Fig. 4a. The detailed training parameters for MNIST and CelebA are summarized in Supplementary Table 1. The bitstream follows a binomial distribution derived from the MCTM. Let *N* represent the number of 1 s observed in the 20 samples. The sampled latent vectors, *Z*_1_ and *Z*_2_, are calculated as follows:

5 $${Z}_{{{\rm{i}}}}=\frac{N-\mu }{\sigma }\cdot R,i=1,2$$

where *μ* is the expected mean (*E*[*N* ]=*n*⋅*p*, with *n* = 20 samples and *p* is 0.5), *σ* is the standard deviation ($\sqrt{n\cdot p\cdot (1-p)}$), and *R* is the desired range for the latent space (set to *R* = 4, a typical value for MNIST dataset). The calculated values of *Z*_1_ and *Z*_2_ are normalized to fall within the range of −4 to 4, ensuring compatibility with the VAE latent space. This normalization is necessary to ensure the latent vectors map to a bounded region, enabling the decoder to generate realistic images^2,3^. The detailed RTN-based VAE architecture, training flow, and latent vector mapping algorithm using FTJ RTN are illustrated in Supplementary Fig. 24 and Supplementary Table 2.

**Fig. 6:**
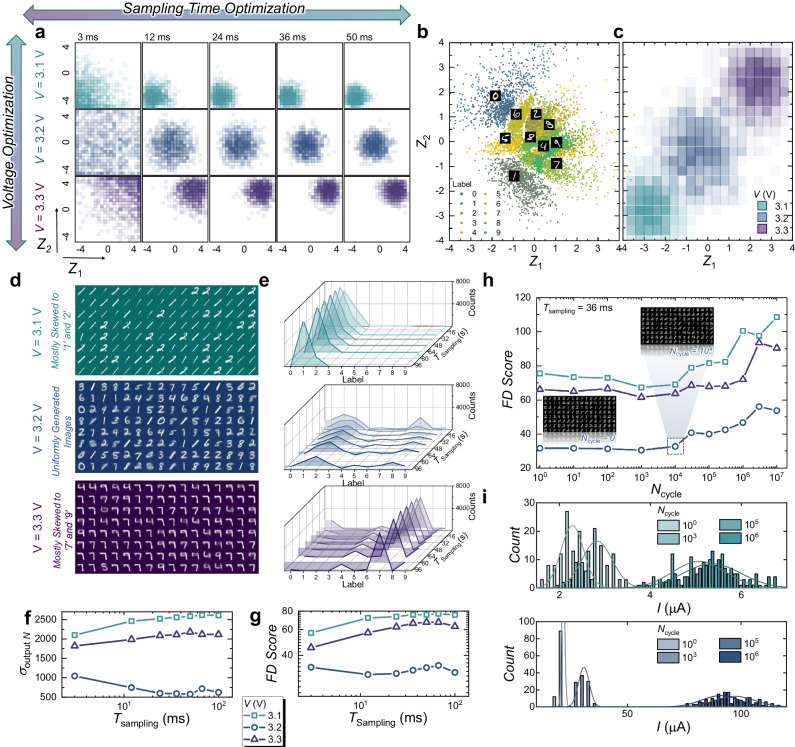
Evaluation of latent space distribution and image generation performance under varying voltage and sampling conditions. **a** Distribution of generated latent vectors for each *V* and *T*_Sampling_, with biased distributions observed at 3.1 V and 3.3 V. **b** Latent space for the actual MNIST dataset, showing the ideal distribution for accurate image generation. **c** Distribution of random variables in the latent space generated by varying *V* at fixed *T*_Sampling_ = 36 ms. **d** Generated MNIST images at different voltages. At *V* = 3.1 V, only digits 1 and 2 are generated, while *V* = 3.2 V produces all digits evenly, and *V* = 3.3 V is biased toward digits 7 and 9. **e** Distribution of generated images by label across different voltages and sampling conditions, with *V* = 3.2 V showing the most uniform distribution. **f** *σ*_output N_ (standard deviation of images per label), of generated images by label, with *V* = 3.2 V and *T*_Sampling_ = 36 ms showing the lowest deviation. **g** FD score of generated images, with *V* = 3.2 V and *T*_Sampling_ = 36 ms, presenting the lowest FD score, indicating the highest image diversity. **h** FD score versus *N*_cycle_s, confirming robust FTJ operation in the absence of RTN. **i** Cycle-dependent current distribution in HRS (up) and LRS (down). Histograms of cell current in LRS at (**a**) *N*_cycle_ = 10^0^, (**b**) *N*_cycle_ = 10^3^, (**c**) *N*_cycle_ = 10^5^, and (**d**) *N*_cycle_ = 10^6^.

Figure 6a shows the distribution of the generated latent vectors in the 2D plane under different *V* and *T*_Sampling_ conditions, while Fig. 6b shows the latent space of the actual MNIST dataset for comparison. For all *V*s, if *T*_Sampling_ is not sufficiently large, the values of *P*_00_ and *P*_11_ increase, causing the latent vector components (*Z*_1_, *Z*_2_) to take extreme values near −4 or 4. As a result, the sampled latent vectors become widely spread, diverging significantly from the latent vector distribution of the actual MNIST data. This divergence can lead to the generation of unrealistic data. As *T*_Sampling_ increases, the SMD decreases, and the variance of Z_1_ and *Z*_2_ also reduces. Consequently, at *T*_Sampling_ = 36 ms, the SMD reaches its minimum and saturates, achieving a desirable latent vector distribution. While further increasing *T*_Sampling_ slightly reduces the SMD, the improvement is negligible, making the 36 ms an optimal choice when considering speed and efficiency. Figure 6c shows the latent vector distribution generated at *T*_Sampling_ = 36 ms for different voltages. At *V* = 3.1 V and *V* = 3.3 V, even with the optimized *T*_Sampling_, the generated latent space is not uniformly distributed and exhibits bias toward specific regions. As shown in Fig. 5d, this bias arises from the inherent imbalance between the high-current state and low-current state. For *V* = 3.1 V, the bias toward values smaller than 0 is due to the frequent occurrence of the low state in the RTN. Conversely, for *V* = 3.3 V, the bias toward values >0 results from the predominance of the high state in the RTN. In contrast, at *V* = 3.2 V and *T*_Sampling_ = 36 ms, the generated latent vector distribution closely resembles the latent space of the actual MNIST dataset, as shown in Fig. 6c. This condition is consistent with the results of SMD optimization, ensuring realistic and meaningful image generation. Training loss evolution and example MNIST outputs during VAE training are shown in Supplementary Fig. 25. The images generated by the decoder using latent vectors sampled at *T*_Sampling_ = 36 ms are presented in Fig. 6d. At *V* = 3.1 V, the decoder predominantly generates the digits 1 and 2, while at *V* = 3.2 V, all digits are evenly generated. At *V* = 3.3 V, the images are biased toward digits 7 and 9. This observation aligns with the latent vector distributions shown in Figs. 6b, c. Specifically, at *V* = 3.1 V, the latent vectors are predominantly distributed to regions in the latent space corresponding to the digits 1 and 2, whereas at *V* = 3.3 V, the vectors are primarily distributed to regions corresponding to 7 and 9. In contrast, at *V* = 3.2 V, the latent space uniformly includes regions corresponding to all digits from 0 to 9, resulting in a balanced representation of the entire dataset. Figure 6d shows MNIST images generated by the VAE. Effects of RTN operating region and sampling time on MNIST generation are presented in Supplementary Fig. 26.

Figure 6e shows the number of images generated for each digit label across various *V* and *T*_Sampling_ conditions. A total of 10,000 images spanning 10 labels (0-9) are generated. For diverse image generation, ideally, there should be no bias toward any specific label, with ~1000 images generated per label. However, for *V* = 3.1 V and *V* = 3.3 V, certain labels dominate the output, with over half of the total generated images biased toward these labels. In contrast, for *V* = 3.2 V, the distribution of generated images is relatively uniform across all labels. To quantitatively assess the uniformity of image generation, we calculated the standard deviation of the number of images generated for each label (*σ*_output N_) and the Fréchet Distance (FD) score to evaluate the diversity of the generated images^2,3^. We report the FD score rather than the Inception Score for MNIST because the Inception Score relies on an ImageNet-pretrained classifier that is poorly matched to 28 × 28, single-domain digit images, leading to misleading results^54^. Figures 6f, g show that both the *σ*_output N_ and the FD score reach their minimum values when *V* is set to 3.2 V and *T*_Sampling_ above 36 ms. Additionally, the decoder, implemented as a deep neural network, assumes that the VMM operations within the network are processed by the FTJ in regions without RTN. As the image generation cycle repeats, cycling stress reduces the on/off ratio of the FTJ, leading to potential errors in VMM operations. To account for this effect, the changes in the on/off ratio as a function of the number of write cycling stress (*N*_cycle_) are incorporated into the simulation. Figure 6h presents the FD score as a function of *N*_cycle_, demonstrating that stable image generation is maintained even up to 10^5^ program/erase cycles. Figure 6i further shows the cycle-dependent current distributions in HRS (top) and LRS (bottom): histograms at (a) *N*_cycle_ = 10^0^, (b) 10^3^, (c) 10^5^, and (d) 10^6^. The HRS peak broadens and drifts more strongly than LRS, increasing their overlap and effectively reducing the on/off ratio, which correlates with the slight FD increase at high cycle counts. Effects of RTN operating region and write-cycle count on MNIST generation are shown in Supplementary Fig. 27. This result confirms the robustness and reliability of the system under repeated operation. Note that the read voltage of 2 V is much lower than the 5 V write voltage and therefore introduces negligible electrical stress. The TER ratio as a function of the number of applied read pulses (*N*_read cycle_) under the read condition of 2 V and 1 ms is shown in Fig. S28, and it remains unchanged up to 10^7^ cycles.

### Image generation performance on face data

**Fig. 7:**
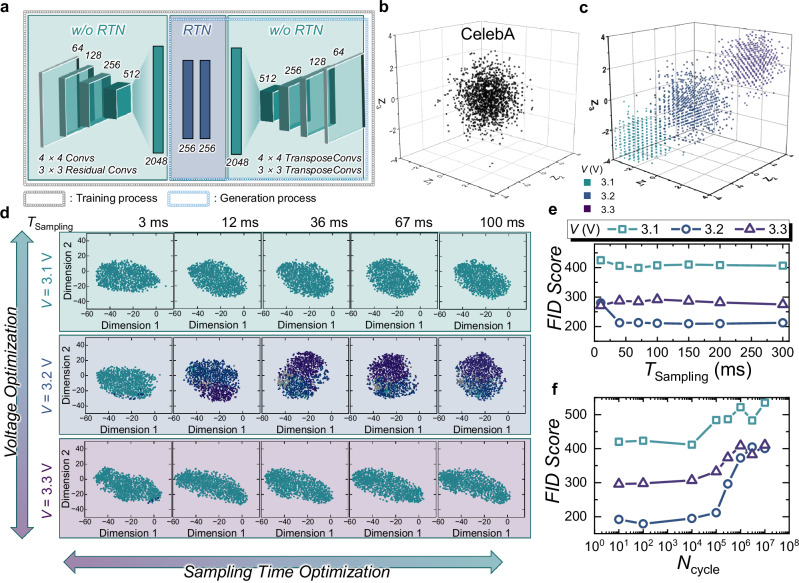
Evaluation of FTJ-based random variable generation for CelebA image synthesis using a VAE model. **a** Schematic of the VAE model for generating CelebA images, showing the encoder and decoder network layers. **b** Latent space mapping pattern of CelebA images, showing a well-distributed Gaussian dimensions. **c** Distribution of FTJ-generated random variables in the latent space at *V* = 3.2 V, showing well-centred results compared to *V* = 3.1 V and 3.3 V. **d** *t*-SNE embeddings of the generated outputs under different voltages. At *V* = 3.1 V and 3.3 V, the embeddings are biased toward extreme regions of the distribution, indicating reduced diversity, whereas *V* = 3.2 V yields a broader and more balanced distribution of generated outputs. **e** FID score as a function of *T*_Sampling_ for different *V*s, showing the optimal condition at *V* = 3.2 V and *T*_Sampling_ = 36 ms. **f** FID score versus the *N*_cycle_ to the FTJ in the decoder, indicating stable image generation up to 10^5^ cycles.

To expand the FTJ-based random variable generation method for larger color image generation, we trained a larger VAE network using the CelebA dataset^25^. Figure 7a shows the encoder structure of the VAE, which maps images to a 256-dimensional latent space using a combination of six layers, including 4 × 4 convolutional layers, 3 × 3 residual blocks, and a fully connected layer. The decoder, designed for image generation, consists of eight layers, including a fully connected layer, 4 × 4 transpose convolutional layers, and 3 × 3 transpose convolutional layers. Figure 7b shows the latent vectors derived from the CelebA dataset during training, where three dimensions (*Z*_1_, *Z*_2_, *Z*_3_) are randomly selected from the 256-dimensional latent space. These dimensions exhibit Gaussian distributions centered at 0, consistent with the characteristics of the latent vectors observed in the MNIST dataset. Figure 7c presents the three-dimensional distribution of random variables generated using the FTJ under different voltage conditions (*V* = 3.1 V, 3.2 V, and 3.3 V) at *T*_Sampling_ = 36 ms. Similar to the analysis in Fig. 6b, three dimensions are randomly selected from the 256-dimensional latent space of the generated vectors. At *V* = 3.2 V, the distribution is well-centered around the origin, reflecting a balanced sampling process. Figure 7d shows *t*-SNE visualizations of the outputs generated by the VAE under different voltage conditions. At *V* = 3.1 V and *V* = 3.3 V, the distributions are biased toward extreme regions, indicating limited diversity in the generated outputs. In contrast, at *V* = 3.2 V, the distribution is broader and more balanced, consistent with more diverse generation and reflecting a well-balanced latent space distribution. CelebA training loss evolution is shown in Supplementary Fig. 29. Effects of RTN operating region and sampling time on CelebA generation are presented in Supplementary Fig. 30. Figure 7e presents the Fréchet Inception Distance (FID) score as a function of *T*_Sampling_ under different *V*s, indicating that the optimized condition is achieved at *V* = 3.2 V and *T*_Sampling_ = 36 ms. We report FID as the primary quantitative metric for generated CelebA images because, in line with prior observations that the Inception Score can be unreliable and misleading on non-ImageNet, single-class datasets, whereas FID more faithfully tracks perceptual quality and diversity^54–57^. Supplementary Fig. 30, Supplementary Fig. 31 and Supplementary Note 6 show *t*-SNE visualizations of ideally software-generated images and CelebA images generated under hardware constraints, respectively. As the generation settings approach the identified optimal point, the *t*-SNE plot resembles that of the ideal software-generated images. This convergence in the embedding space substantiates our claim that the proposed optimal condition best aligns the generator with the target data manifold. Figure 7f presents the FID score as a function of the *N*_cycle_ applied to the FTJ in the decoder, showing that stable image generation is maintained up to 10⁵ write cycles. Effects of RTN operating region and write-cycle count on CelebA generation are shown in Supplementary Fig. 32. Also, the change in FID for CelebA images generated under varying IR drop is presented in Supplementary Fig. 33. This indicates that the proposed FTJ array can generate high-quality images without being electrically affected by IR drop. Supplementary Note 7 describes how non-idealities affect the CelebA FID. In particular, it reports the impact of the measured update-curve nonlinearity and the level-to-level variation on the FID in our device-informed simulations.

We note that the optimized latent sampling condition uses a 36 ms sampling window per FTJ. For real-time generation at 30 frames per second with a 256-dimensional latent vector and 16 parallel bitstreams, the sampler must provide 256 × 30 × 16 samples per second, which corresponds to 122,880 samples per second. Under a 36 ms sampling window, a single FTJ provides about 27 samples per second, which implies, on the order of a few thousand FTJs, ~4552 devices dedicated to RTN sampling. This sampling overhead represents a trade-off of bitstream-based RTN sampling, and it can be reduced by algorithm-level choices such as lowering the latent dimension or reducing the number of bitstreams. For completeness, the throughput, minimum arraying for the decoder VMM, ADC and read energy overhead under the same 30 fps target are discussed in Supplementary Note 8. Table 1 compares the proposed method with previous works on generative models implemented with various devices. Unlike previous works, which only support partial operations (either stochastic sampling or reliable VMM), this study provides a complete solution that integrates both operations. Additionally, this method includes circuit-level validation through actual measurements using a large-scale FTJ array, extending beyond the device-level and simulation-level approaches of prior studies. The FTJ-based platform is also quantitatively benchmarked against the mature CMOS-based designs in area, power per sample, and latency (Table 1). For VMM specifically, our FTJ arrays achieve <10 fJ/MAC, over ×40 lower than the ~430 fJ/MAC reported for 8‑bit MACs on a widely deployed TPU^11–13^, and sustain ~1.31 TOPS per 256 × 256 array at 10 MHz, with throughput scaling through array replication. Finally, we define the Area–Power–Randomness Cost (APRC) as *APRC* = (*A* × *P*)/*η*_NIST_, where *A* is the sampling-hardware area, *P* is the power during stochastic operation, and *η*_NIST_ is the NIST SP 800-22 pass ratio. A smaller *APRC* indicates a better trade-off among area, power, and random quality. Figure S34 visualizes the area–power–randomness comparison in a 3D space, and Fig. S35 summarizes the same results using *APRC* for direct numerical comparison. The detailed definitions of *A* and *P* and the fair-comparison methodology are provided in Supplementary Note 9.

## Discussion

In this study, we proposed a unified approach to hardware-based image generation, utilizing the electrical characteristics of FTJs to integrate stochastic sampling and deterministic decoding within a single device platform. By harnessing RTN characteristics and the non-volatile memory functionality of FTJ, we addressed the limitations of conventional systems, which require separate components and off-chip integration. Precise control of the applied voltage and sampling time enabled effective tuning of RTN parameters, allowing for the generation of optimized latent vectors and reliable decoding within the same wafer. This approach significantly improved efficiency and scalability in image generation. Furthermore, circuit-level validation demonstrated the robustness of our system, achieving successful generation of handwritten digit images (MNIST dataset) and high-resolution facial images (CelebA dataset). These results underscore the potential of FTJ-based systems for real-time, energy-efficient generative AI applications. The proposed CMOS-compatible and VLSI-scalable FTJ-based integrated systems present a promising solution for seamlessly integrating stochastic and deterministic operations in next-generation microelectronics, supporting further developments in media content creation, autonomous systems, and related applications.

## Methods

### Fabrication process of FTJ arrays

A 200-nm SiO_2_ layer was thermally grown on an RCA-cleaned Si wafer, followed by the deposition of a 100-nm polycrystalline silicon (poly-Si) layer using low-pressure chemical vapor deposition. The poly-Si bottom electrode was doped with n-type dopants to ensure conductivity. Photolithography and dry etching processes were subsequently conducted to define the patterned bottom electrode structure. A thin tunneling oxide layer (SiO_2_, ~1.2 nm) was grown by chemical oxidation. Next, a 7-nm-thick Hf_0.5_Zr_0.5_O_2_ (HZO) ferroelectric film was deposited using thermal ALD, employing alternating cycles of Hf and Zr precursors at a 1:1 ratio for 44 cycles. A 100-nm-thick TiN top electrode was deposited using sputtering, after which the top-electrode pattern was defined through photolithography and dry etching. Finally, RTA at 700 °C for 30 s was performed to crystallize the HZO layer into its ferroelectric phase. A detailed schematic illustration of the fabrication steps is provided in Supplementary Fig. 2.

### Electrical measurements

The ferroelectric characteristics of the fabricated HZO-based FeFET devices were examined using a probe station in combination with a Keysight B1500A semiconductor parameter analyzer. Direct current measurements were carried out with a Keysight B1500A analyzer equipped with a source measurement unit. For pulse characterization, the B1500A was paired with both a waveform generator/fast measurement unit (B1530A, WGFMU) and a high-voltage semiconductor pulse generator (B1525A, SPGU). Array-level characterization employed a probe card connected to the B1500A and a Keysight E5250A switching matrix. Signals were routed to multiple channels through the switching matrix and operated via LABVIEW control. Unless otherwise stated, all electrical tests and characterizations were conducted in ambient atmosphere at room temperature.

### Transmission electron microscopy sample preparation

TEM samples were prepared using a focused ion beam (Helios G4 HX) and further refined through low-energy ion milling (below 2 keV) to minimize surface damage and optimize imaging conditions.

### LFN measurement protocol

LFN characterization was performed using a Keysight B1500A semiconductor parameter analyzer, an SR570 low-noise current preamplifier (Stanford Research Systems), and a Keysight 35670 A dynamic signal analyzer. During measurement, the B1500A supplied the gate bias to the device under test, while the drain current was fed into the SR570. The preamplifier converted the current fluctuations into voltage signals, which were subsequently processed by the dynamic signal analyzer to extract the power spectral density.

Bandwidth-related artifacts were also considered. The SR570 maintains both amplitude and phase accuracy within its specified operating bandwidth. In low-noise mode, the nominal bandwidths are 2 kHz, 20 kHz, and 200 kHz for sensitivity ranges of 100 nA, 1 µA, and 10 µA, respectively. Since the frequency window adopted in this work was well below these cut-off limits, no appreciable spectral distortion is anticipated.

## Supplementary information

Supplementary Information

Transparent Peer Review file

## Source data

Source Data

## Data Availability

All data supporting the findings of this study are available within the Article, Supplementary Information, and Source Data file. The CelebA dataset used in this study is publicly available at https://mmlab.ie.cuhk.edu.hk/projects/CelebA.html. Source data are provided with this paper.

## Code availability

The simulations in this study were performed using methods and parameters described in the Article and Supplementary Information.
