# Supplementary material for: CMOS-compatible ferroelectric tunnel junctions integrate stochastic sampling and deterministic computing for image generation
Source: Nat Commun. 2026 May 8;17:6236. doi: 10.1038/s41467-026-72969-6 (PMC13369162; doi:10.1038/s41467-026-72969-6)
Supplement: Supplementary file 1 — Supplementary Information [file 41467_2026_72969_MOESM1_ESM.pdf]

Supplementary Information

CMOS compatible ferroelectric tunnel junctions integrate stochastic sampling and deterministic computing for image generation

Ryun-Han Koo[1†], Jonghyun Ko[1†], Wonjun Shin[2†], Sangwoo Ryu[1,3], Jiseong Im[1], Sung-Ho Park[1], Joon Hwang[1], Minsuk Song[4], Youngchan Cho[2], Jangsaeng Kim[5], Gyuweon Jung[1], Daewoong Kwon[4], and Jong-Ho Lee[1,]*

[1]Department of Electrical and Computer Engineering and Inter-university Semiconductor Research Center, Seoul National University, Seoul 08826, Republic of Korea
[2]Department of Semiconductor Convergence Engineering, Sungkyunkwan University, Suwon 16419, Republic of Korea
[3]SK Hynix Inc, Icheon 17336, Republic of Korea
[4]Department of Nanoscale Semiconductor Engineering, Hanyang University, Seoul 04763, Republic of Korea
[5]Department of Electronic Engineering, Sogang University, Seoul 04107, Republic of Korea

[†] These authors contributed equally: Ryun-Han Koo, Jonghyun Ko, Wonjun Shin

** Corresponding author (e-mail: jhl@snu.ac.kr)*

Keywords: *Ferroelectrics, Low-frequency Noise (LFN), Random Telegraph Noise (RTN), Variational Autoencoder (VAE), Hardware computing*

Contents

Supplementary Figure 1–35

Supplementary Table 1-2

Supplementary Note 1–9

Supplementary References

Supplementary Figures

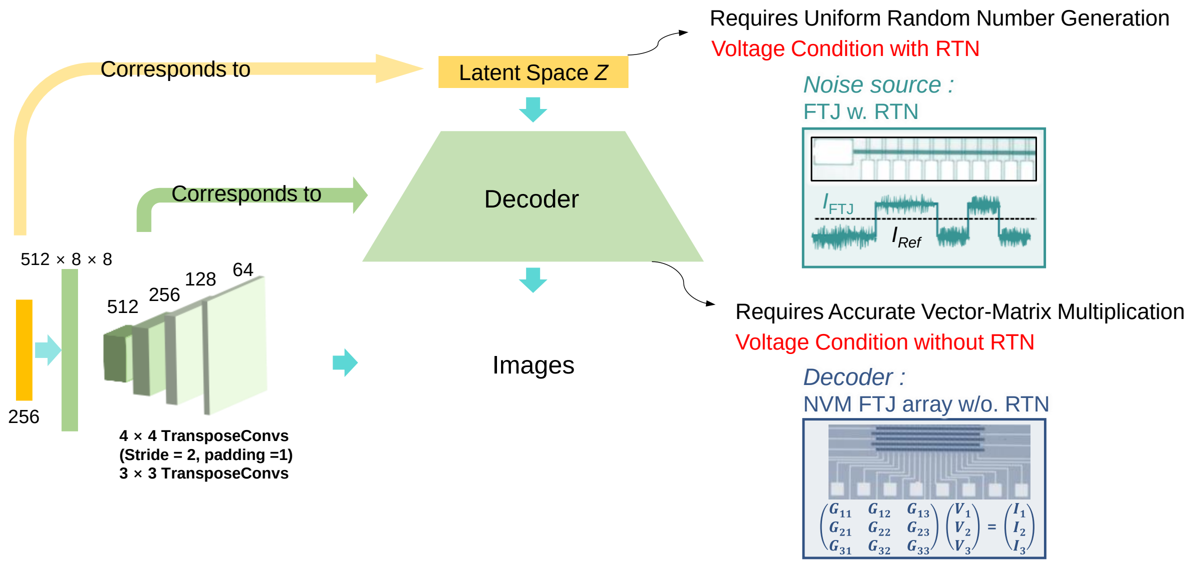

Supplementary Figure 1. VAE image generation flow: latent vector z is stochastically sampled (FTJ under RTN for uniform RNG) and fed to a transposed convolution decoder whose deterministic weights are realized by an NVM FTJ crossbar operated without RTN, producing images.

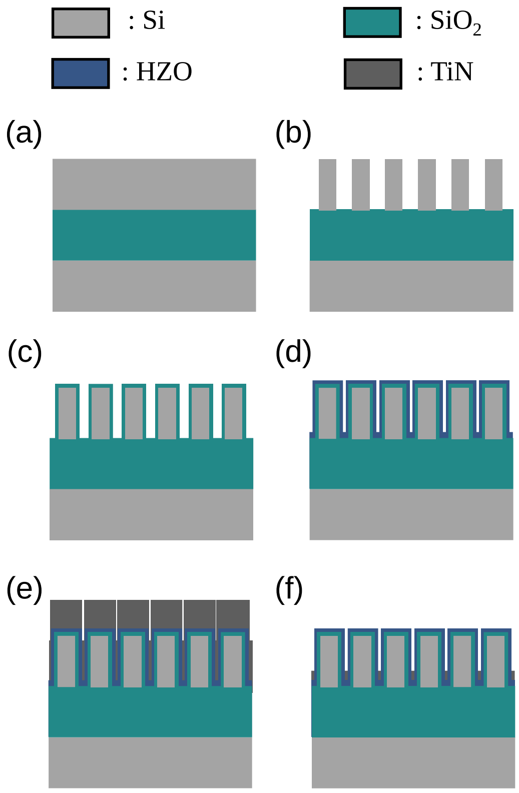

Supplementary Figure 2. Schematic illustration of the fabrication process for the MFIS-FTJ array. (a) A 200-nm SiO_2 layer is thermally grown on an RCA-cleaned Si wafer, followed by the deposition of a 100-nm polycrystalline silicon (poly-Si) layer using low-pressure chemical vapor deposition (LPCVD). The poly-Si bottom electrode is doped with n-type dopants to ensure conductivity. (b) Photolithography and dry etching processes are performed to define the patterned bottom electrode structure. (c) A thin tunneling oxide (SiO_2, ~1.2 nm) is grown by chemical oxidation. (d) A 7-nm-thick $Hf_{0.5}Zr_{0.5}O_2$ (HZO) ferroelectric film is deposited using thermal atomic layer deposition (ALD) with alternating cycles of Hf and Zr precursors at a ratio of 1:1 for 44 cycles. (e) A 100-nm-thick TiN top electrode is deposited using sputtering. (f) The top electrode pattern is defined through photolithography and dry etching. Finally, a rapid thermal annealing (RTA) treatment at 700°C for 30 s is conducted to crystallize the HZO layer into its ferroelectric phase.

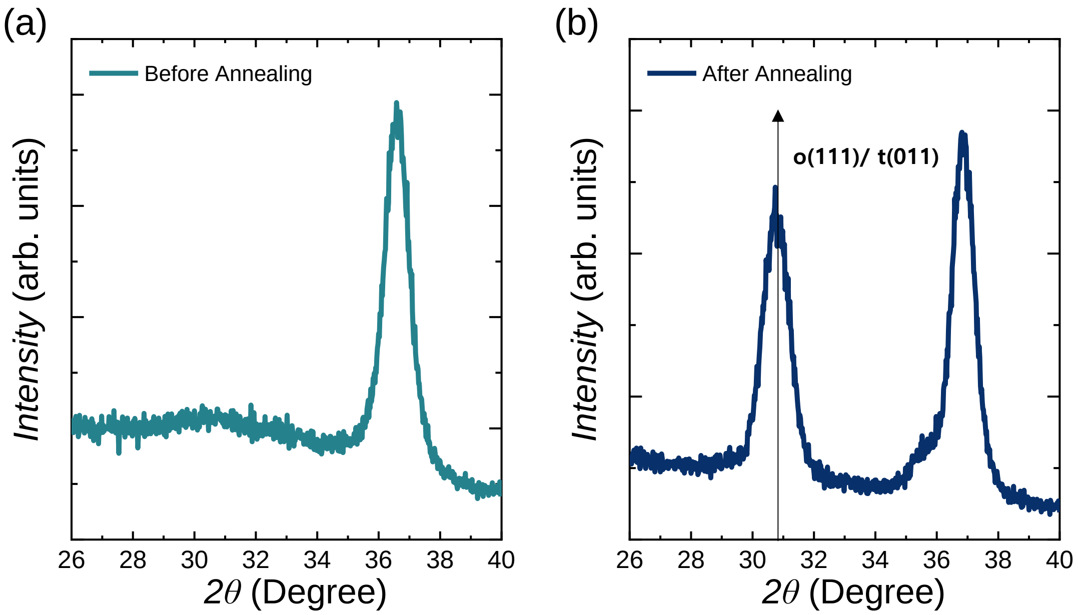

Supplementary Figure 3. Grazing-incidence X-ray diffraction (GIXRD) patterns of HZO films. (a) As-deposited HZO without annealing, showing no evidence of the orthorhombic phase. (b) HZO capped with TiN and subjected to rapid thermal annealing, where strain induced by the TiN overlayer stabilizes the ferroelectric orthorhombic o-(111) phase along with the tetragonal t-(011) reflection.

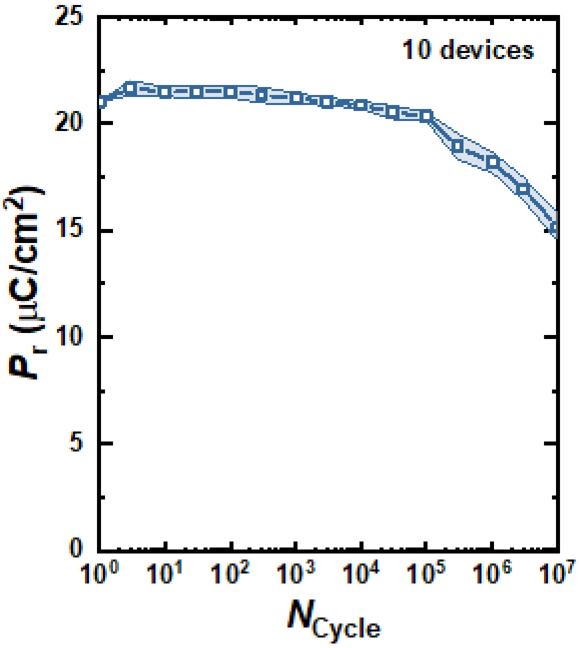

Supplementary Figure 4. Polarization endurance characteristics of the fabricated FTJ device. P_r values are normalized by the initial P_r ($P_{r,initial}$). One rectangular program pulse (5 V, 10 μs) followed by one erase pulse (−5 V, 10 μs) constitutes a single cycle (N_{cycle} = 1). The devices exhibit a slight initial increase in polarization, indicative of a wake-up phenomenon, followed by minor degradation beyond 10^5 cycles, demonstrating robust and stable endurance characteristics. The measurements were performed on 10 devices, and the standard deviation is represented by error bars (shaded in light blue).

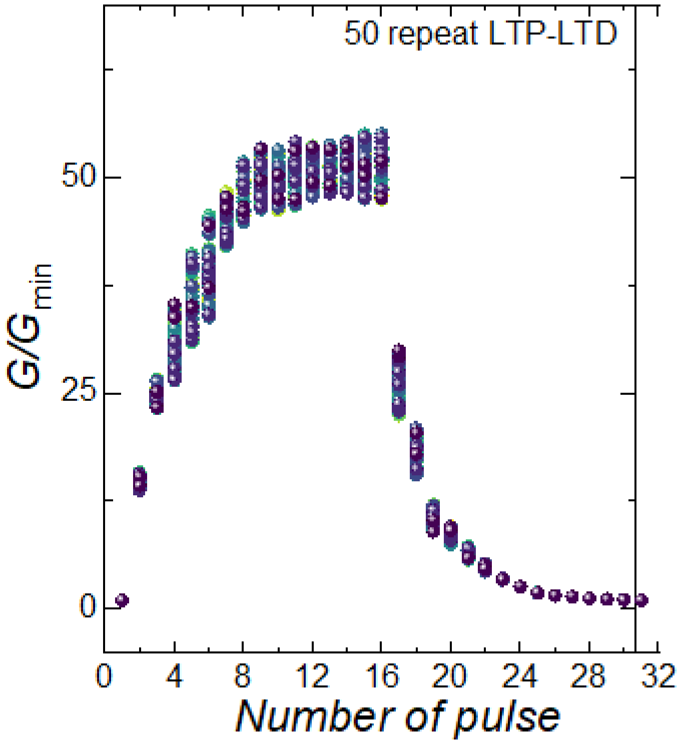

Supplementary Figure 5. Repetitive long-term potentiation (LTP) and long-term depression (LTD) characteristics of the HZO-based FTJ device. Normalized conductance (G/G_{min}) as a function of the number of applied pulses over 50 consecutive LTP–LTD cycles.

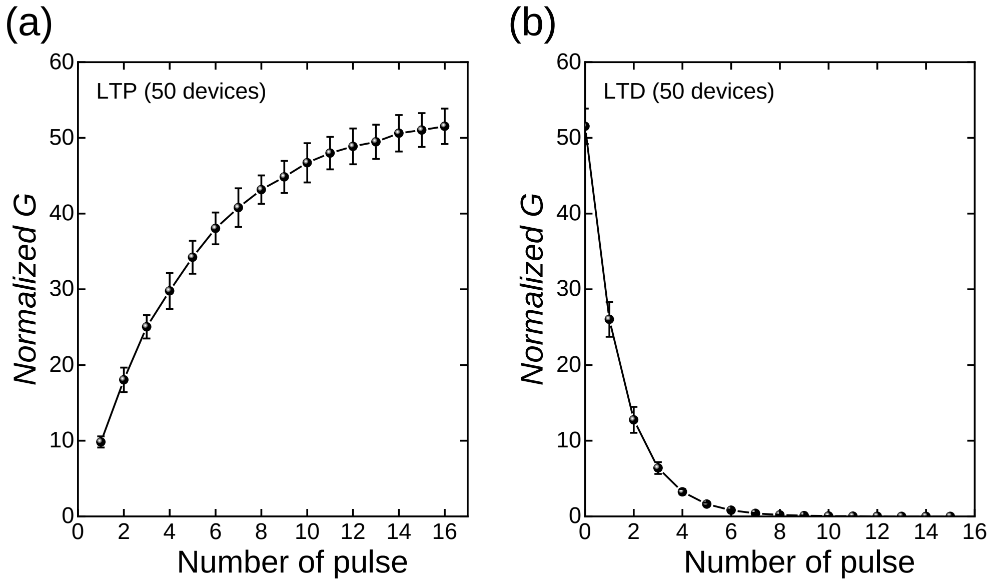

Supplementary Figure 6. Averaged (a) LTP and (b) LTD trajectories across 50 devices with standard-deviation error bars.

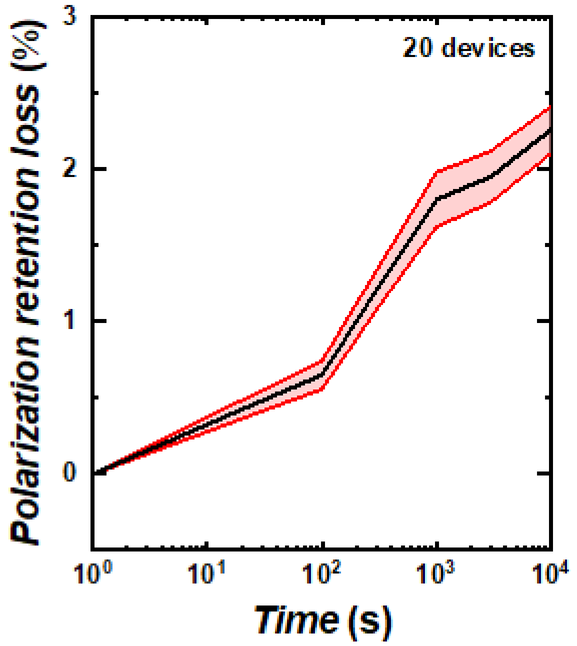

Supplementary Figure 7. Retention characteristics of polarization in 20 HZO-based FTJ devices. Polarization retention loss (%) as a function of elapsed time (log scale) measured across 20 devices. The average trend (black line) and standard deviation (shaded area) indicate minimal polarization loss (< 3%) up to 10^4s.

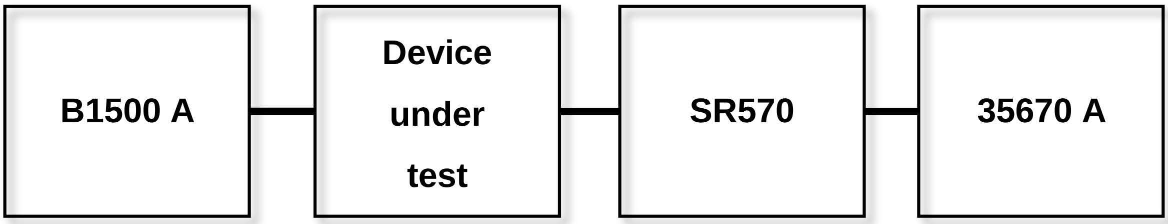

Supplementary Figure 8. Experimental setup for low-frequency noise (LFN) and random telegraph noise (RTN) measurements in HZO-based FTJ devices. Schematic of the measurement configuration integrating a semiconductor parameter analyzer (B1500A) for DC bias application, a low-noise current preamplifier (SR570) for signal amplification, and a dynamic signal analyzer (35670A) for power spectral density (PSD) analysis. Representative current–time waveforms with and without RTN and their corresponding PSD plots are shown to illustrate the signal acquisition process.

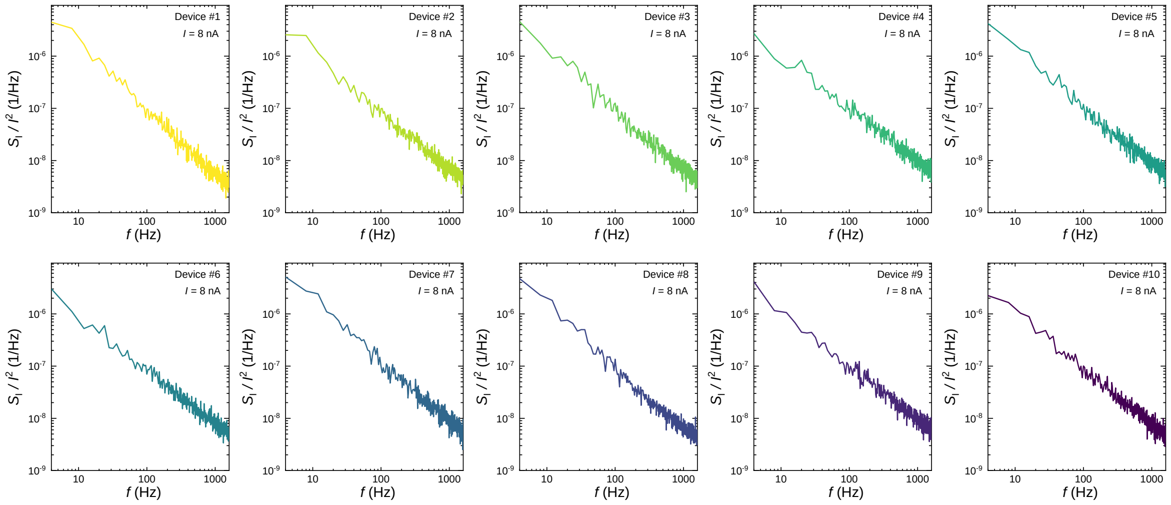

Supplementary Figure 9. Low-frequency noise spectra of 10 HZO-based FTJ devices at I = 8 nA. S_I/I^2 versus frequency for 10 different devices, all exhibiting $1/f$ noise behavior in the low-current regime. The consistency across devices confirms the reproducibility of the low-noise operating region suitable for deterministic non-volatile memory operations.

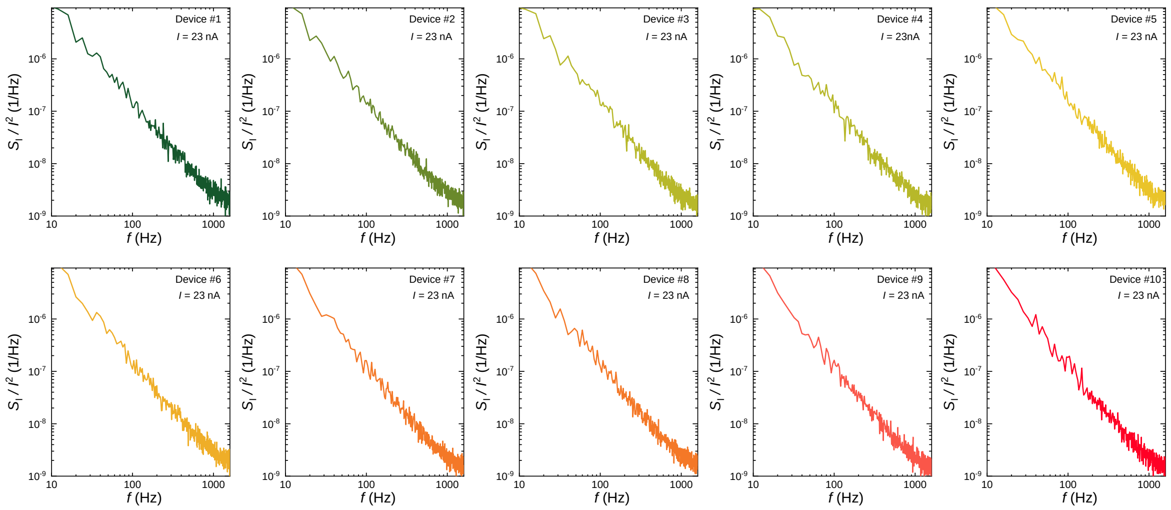

Supplementary Figure 10. Low-frequency noise spectra of 10 HZO-based FTJ devices exhibiting 1/f^2 behavior. S_I/I^2 versus frequency for 10 different devices measured in the high-current regime, where random telegraph noise (RTN) dominates. The clear 1/f^2 dependence across all devices confirms the stochastic switching dynamics between discrete current states.

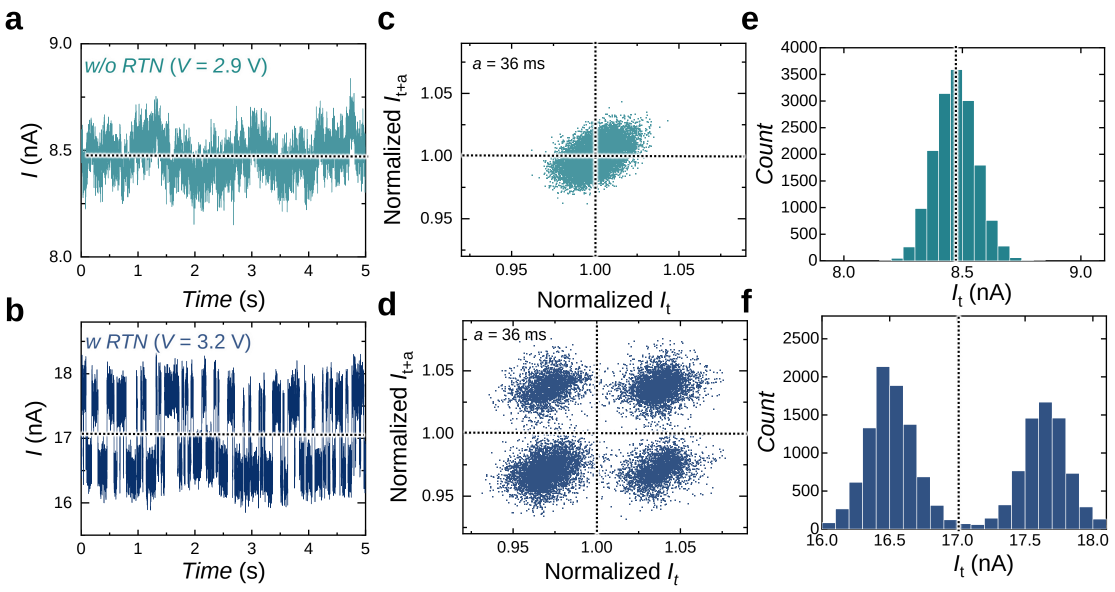

Supplementary Figure 11. Analysis of RTN behavior and noise mechanisms in the FTJ with varying electrical conditions. (a) DC transient measurement at V = 2.9 V, showing no RTN behavior. (b) DC transient measurement at V = 3.2 V, clearly exhibiting RTN. (c) Time-lag plot at V = 2.9 V. (d) Time-lag plot at V = 3.2 V. h Histogram of current distribution at V = 2.9 V. (e) Histogram of current distribution at V = 3.2 V. (f) Illustration of generating a 0/1 sequence based on the FTJ current response, modeled as a Markov chain.

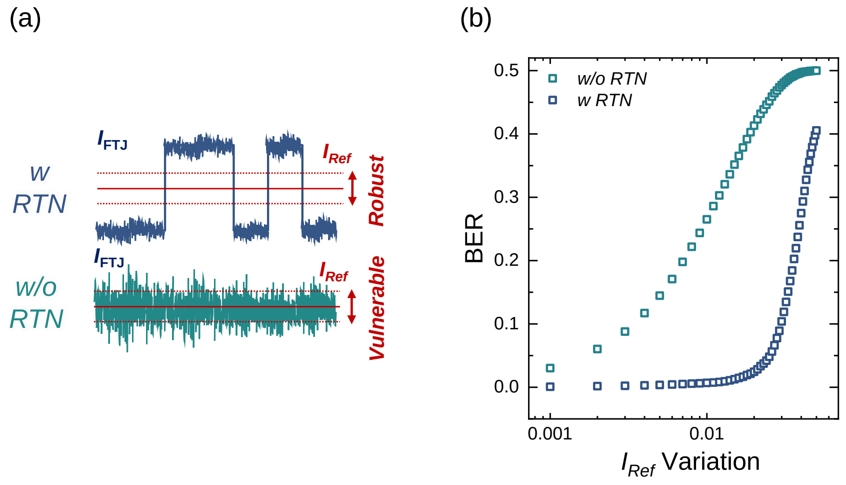

Supplementary Figure 12. Robustness to I_{Ref} drift with and without RTN. (a) Schematic explaining why an RTN-driven two-level current provides larger decision margins around I_{Ref} than a low-noise signal, making sensing less sensitive to I_{Ref} drift. (b) Bit-error rate (BER) versus I_{Ref} variation for RTN operation (V = 3.3 V) and non-RTN operation (V = 2.9 V). RTN operation shows lower BER for the same drift magnitude.

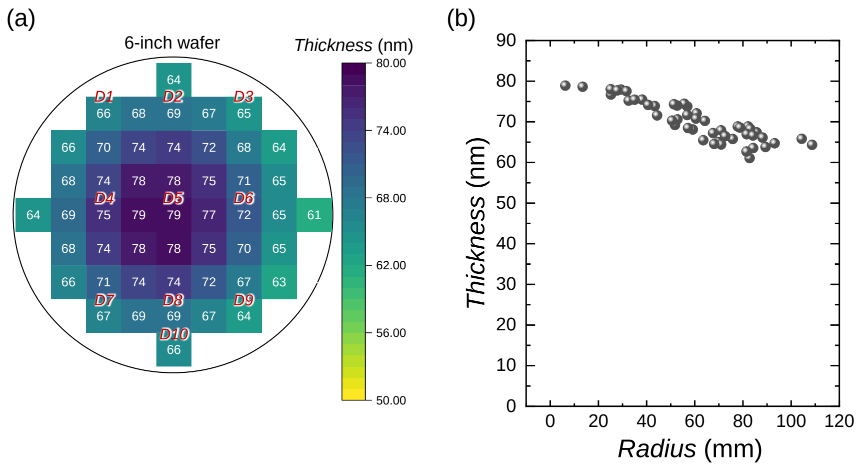

Supplementary Figure 13. Wafer-scale thickness uniformity of ALD HZO. (a) Six-inch wafer map showing ellipsometry-measured HZO thickness at 49 sampled points. The locations of the 10 devices (D1 to D10) used for RTN characterization are also marked on the map. (b) Thickness versus radial distance from the wafer center, showing a slight decreasing trend toward the edge.

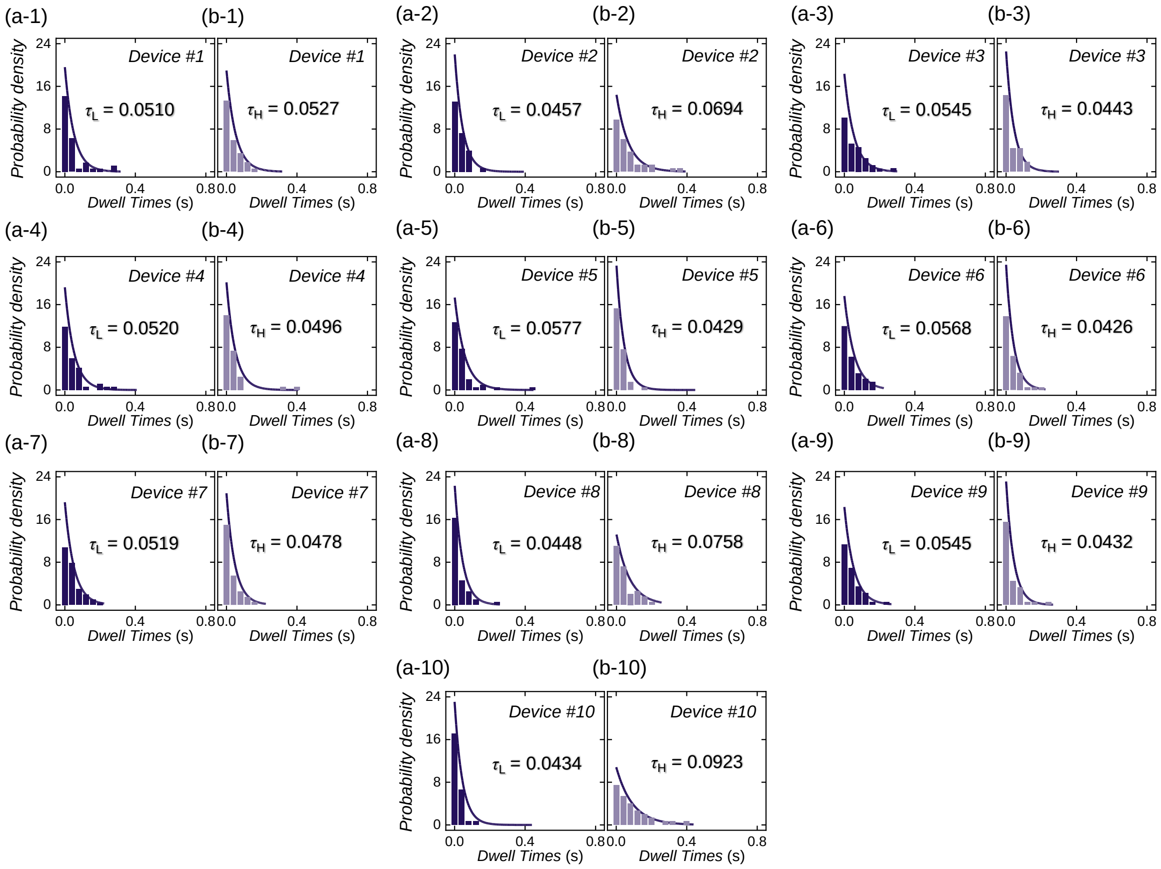

Supplementary Figure 14. RTN dwell-time statistics at a single bias (a) Histograms of the low-state dwell time τ_L for 10 devices measured at V = 3.28 V with Poisson (exponential) fits. (b) Histograms of the high-state dwell time τ_H for the same devices and bias with Poisson fits. Across devices the RTN statistics follow Poisson processes and the occupancy of the two levels is balanced at this bias.

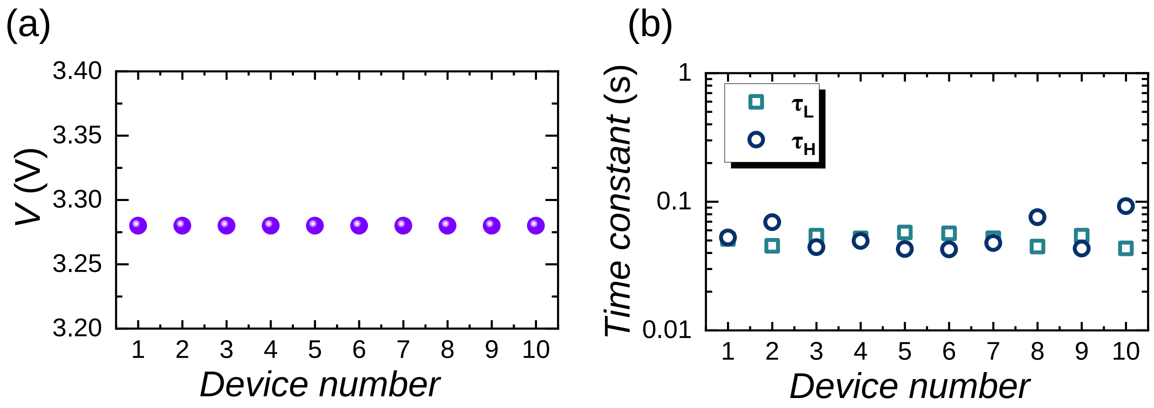

Supplementary Figure 15. Device-wise bias and fitted time constants. (a) Applied read voltage for each device used in Fig. R1. (b) Dwell-time constants extracted from Poisson fitting, reported per device for both τ_L and τ_H.

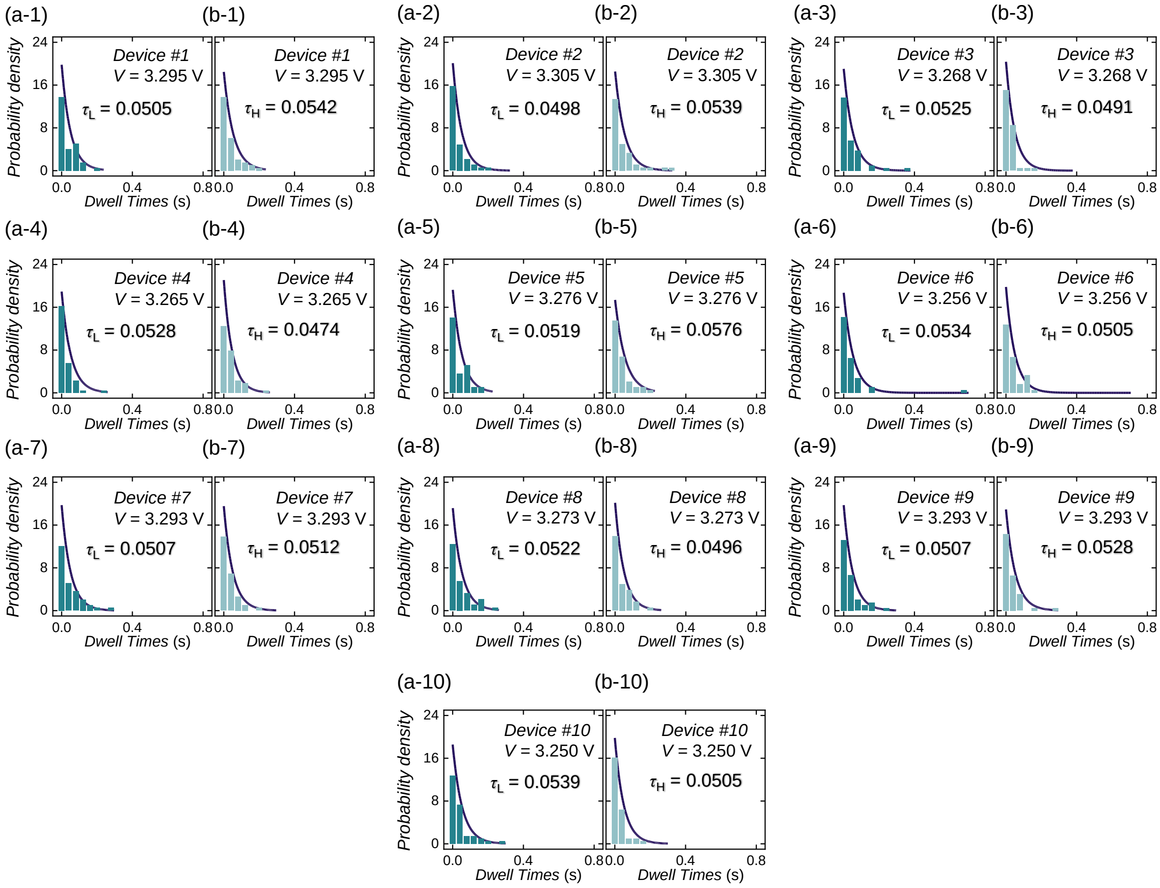

Supplementary Figure 16. Bias equalization across devices for balanced occupancy. (a) Histograms of τ_L with Poisson fits for 10 devices, each biased at its own equalized voltage in the range 3.25–3.30 V to balance state occupancy. (b) Corresponding τ_H histograms with Poisson fits. Compared with Fig. R1, per-device bias equalization yields more balanced occupancy. Inset shows the applied voltage for each device.

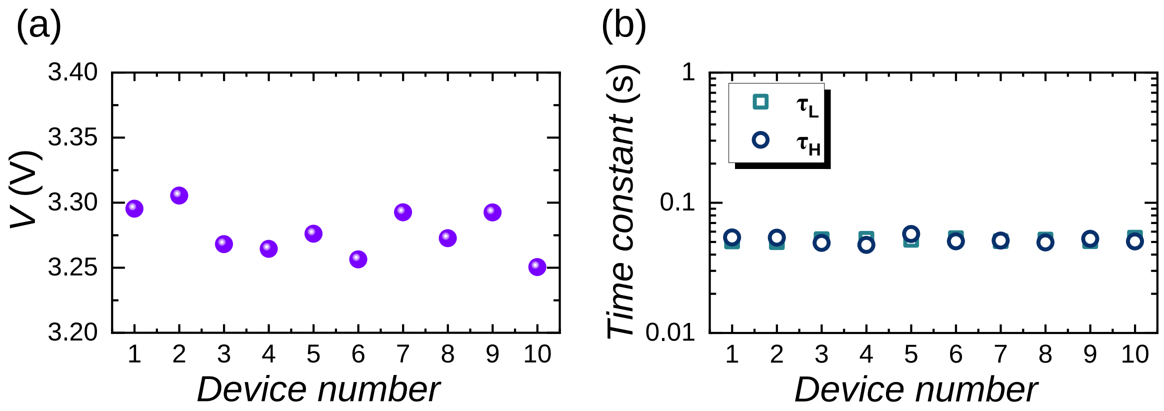

Supplementary Figure 17. Summary of equalized bias and fitted constants. (a) Optimized read voltage for each device. (b) Dwell-time constants τ_L and τ_H under the optimized bias for each device.

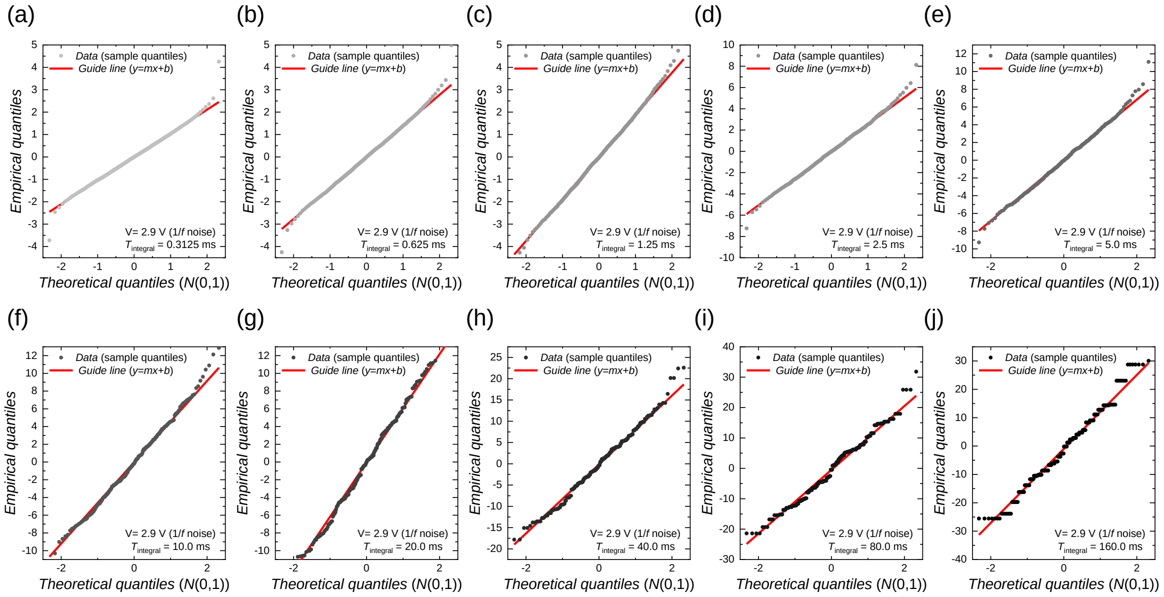

Supplementary Figure 18. Q-Q plots of the standardized block sums $Z_N = (S_N - \mu_N)/(\sigma N^{1/2})$) for a 1/*f* noise signal (*V* = 2.9 V). The *x*-axis shows theoretical quantiles of *N*(0,1), and the *y*-axis shows empirical quantiles of Z_N. Dots shows the empirical quantiles; the red line is a data-based guideline, *y* = *mx* + *b*. Panels correspond to the different integration times: (a) 0.3125 ms, (b) 0.625 ms, (c) 1.25 ms, (d) 2.50 ms, (e) 5.0 ms, (f) 10.0 ms, (g) 20.0 ms, (h) 40.0 ms, (i) 80.0 ms, (j) 160.0 ms). The 1/*f* noise aligns with the 45-degree line even at short integration time, indicating rapid convergence to normality.

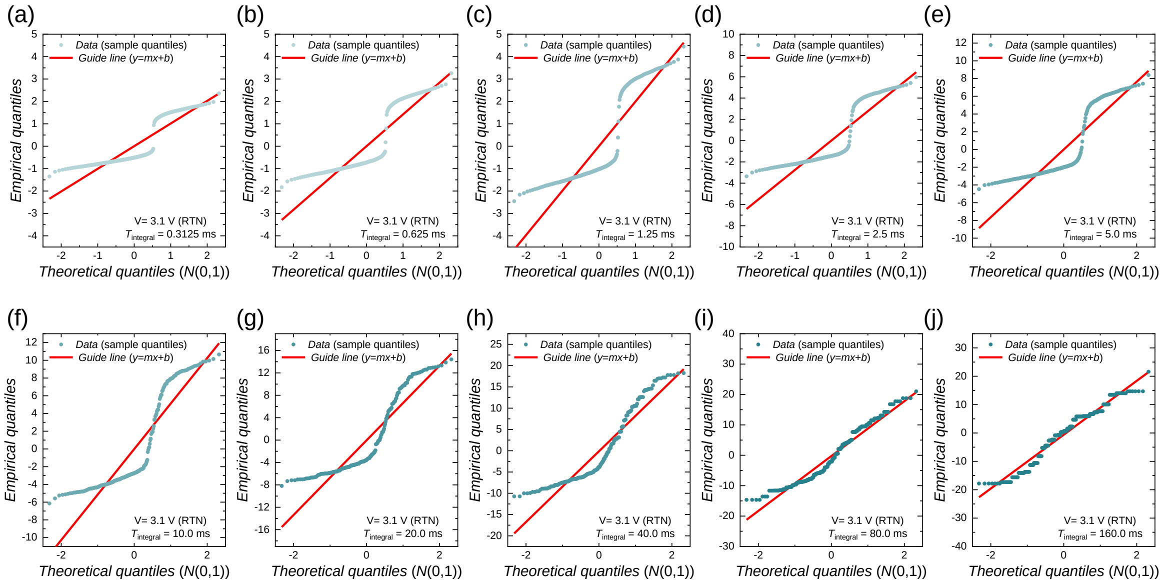

Supplementary Figure 19. QQ plots of the standardized block sums $Z_N = (S_N - \mu_N)/(\sigma N^{1/2})$ for an RTN signal (V = 3.1 V). The x-axis shows theoretical quantiles of $N(0,1)$, and the y-axis shows empirical quantiles of Z_N. Dots show the empirical quantiles; the red line is a data-based guideline, $y = mx + b$. Panels correspond to the different integration times: (a) 0.3125 ms, (b) 0.625 ms, (c) 1.25 ms, (d) 2.50 ms, (e) 5.0 ms, (f) 10.0 ms, (g) 20.0 ms, (h) 40.0 ms, (i) 80.0 ms, (j) 160.0 ms). The RTN signal retains an S-shaped curvature and heavy tails due to long-range correlation.

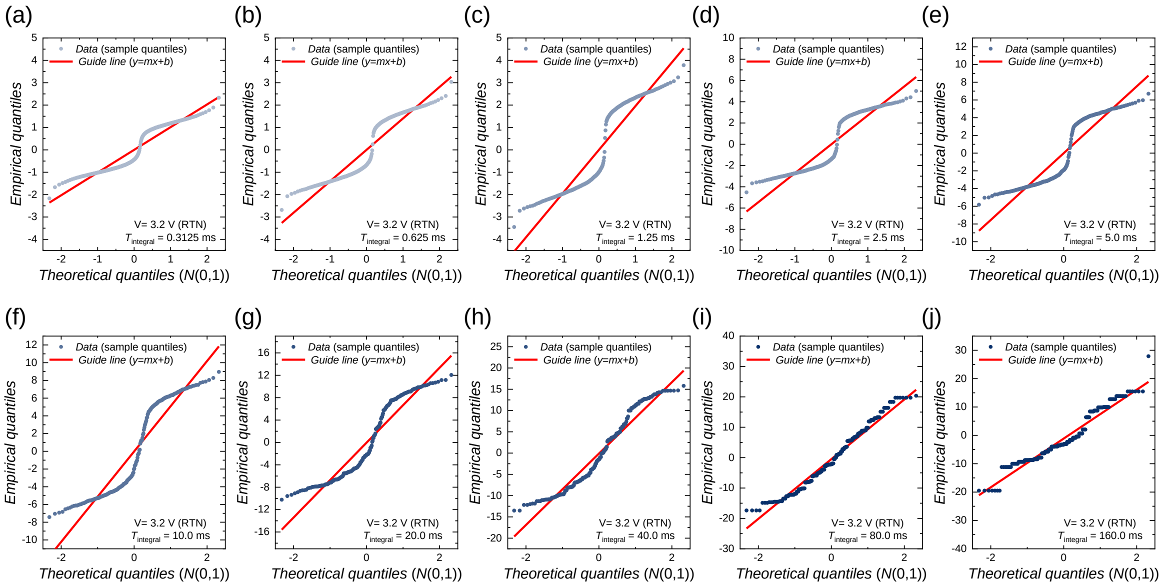

Supplementary Figure 20. QQ plots of the standardized block sums $Z_N = (S_N - \mu_N)/(\sigma_N{}^{1/2})$) for an RTN signal ($V$ = 3.2 V). The x-axis shows theoretical quantiles of N(0,1), and the y-axis shows empirical quantiles of Z_N. Dots show the empirical quantiles; the red line is a data-based guideline, $y = mx + b$. Panels correspond to the different integration times: (a) 0.3125 ms, (b) 0.625 ms, (c) 1.25 ms, (d) 2.50 ms, (e) 5.0 ms, (f) 10.0 ms, (g) 20.0 ms, (h) 40.0 ms, (i) 80.0 ms, (j) 160.0 ms). The RTN signal retains an S-shaped curvature and heavy tails due to long-range correlation.

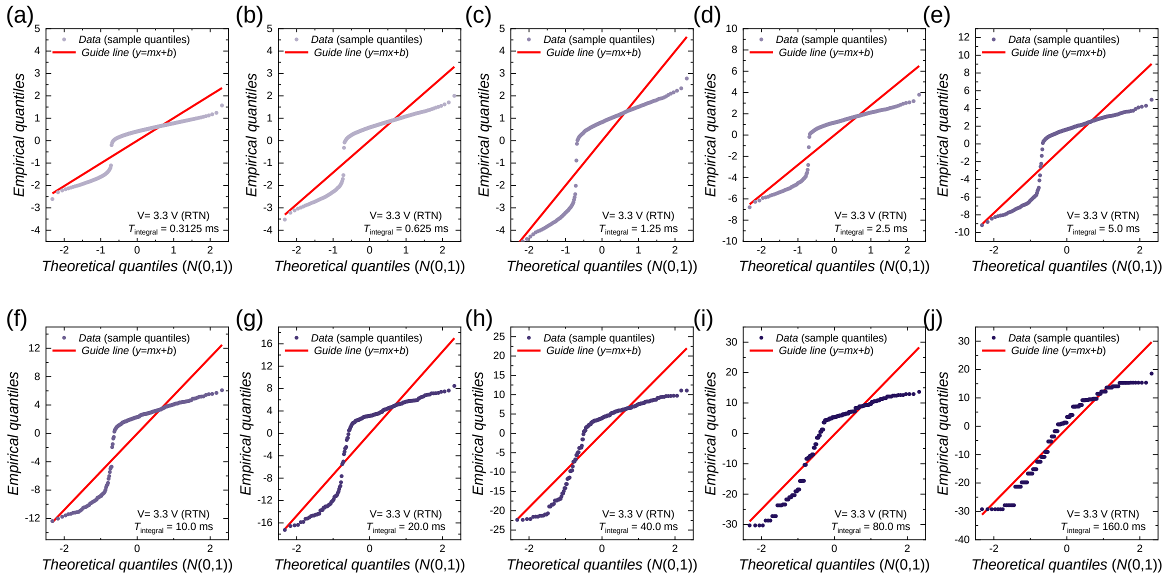

Supplementary Figure 21. QQ plots of the standardized block sums $Z_N = (S_N - \mu_N)/(\sigma_N^{1/2})$ for an RTN signal (V = 3.3 V). The x-axis shows theoretical quantiles of $N(0,1)$, and the y-axis shows empirical quantiles of Z_N. Dots show the empirical quantiles; the red line is a data-based guideline, $y = mx + b$. Panels correspond to the different integration times: (a) 0.3125 ms, (b) 0.625 ms, (c) 1.25 ms, (d) 2.50 ms, (e) 5.0 ms, (f) 10.0 ms, (g) 20.0 ms, (h) 40.0 ms, (i) 80.0 ms, (j) 160.0 ms). The RTN signal retains an S-shaped curvature and heavy tails due to long-range correlation.

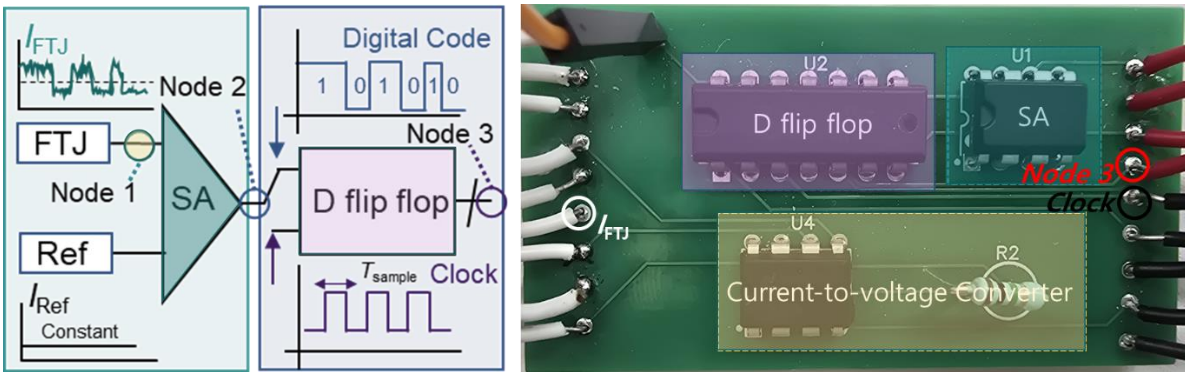

Supplementary Figure 22. Custom PCB implementation for RTN-based random bitstream generation. (Left) Block diagram of the measurement circuit integrating an FTJ device, sense amplifier (SA), D flip-flop, and current-to-voltage converter for converting RTN-induced current fluctuations into digital bit sequences. (Right) Photograph of the fabricated PCB, highlighting the main functional blocks including the D flip-flop (U2), sense amplifier (U1), and current-to-voltage converter (U4).

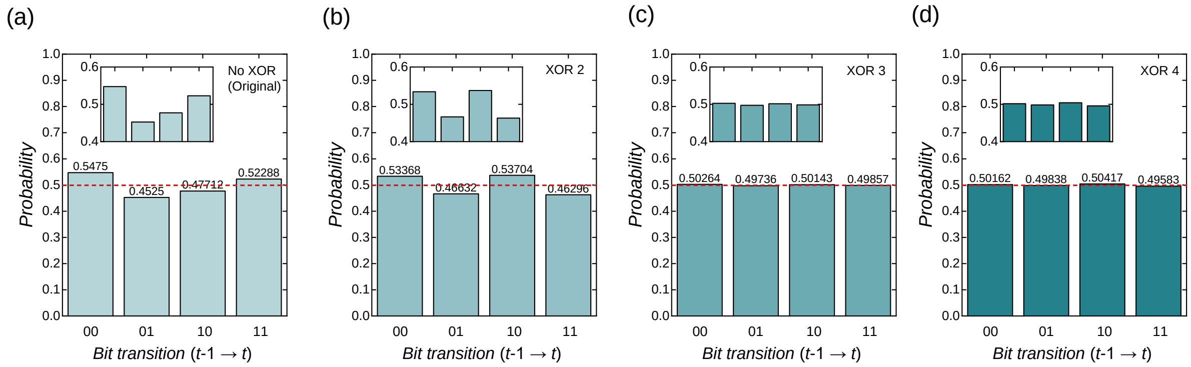

Supplementary Figure 23. XOR post-processing improves Markov transition balance. (a) Bit-transition probability *P* for the raw, no-XOR bitstream. (b) Same metric after XOR-2 combining. (c) Same metric after XOR-3 combining. Increasing XOR depth drives the transition probabilities toward the ideal 0.5.

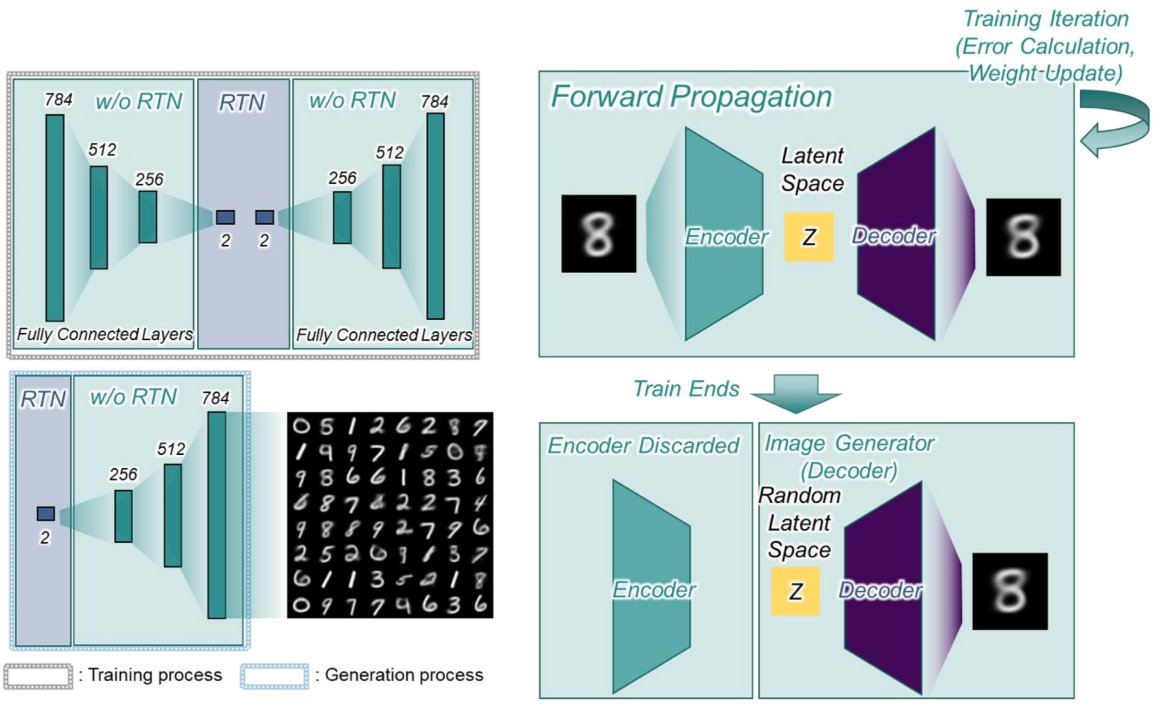

Supplementary Figure 24. Schematic of the variational autoencoder (VAE) architecture incorporating RTN-based latent space sampling. (Left) Network structure used during training, consisting of an encoder (784–512–256–2), RTN-based stochastic latent vector generator, and decoder (2–256–512–784). Fully connected layers without RTN are used for deterministic vector–matrix multiplications, while the RTN block enables stochastic latent space sampling. (Right) VAE training process, where the encoder maps input images to the latent space, followed by decoding and weight updates through forward propagation. After training, the encoder is discarded, and the decoder alone is used to generate images from random latent vectors provided by the FTJ-based RTN source.

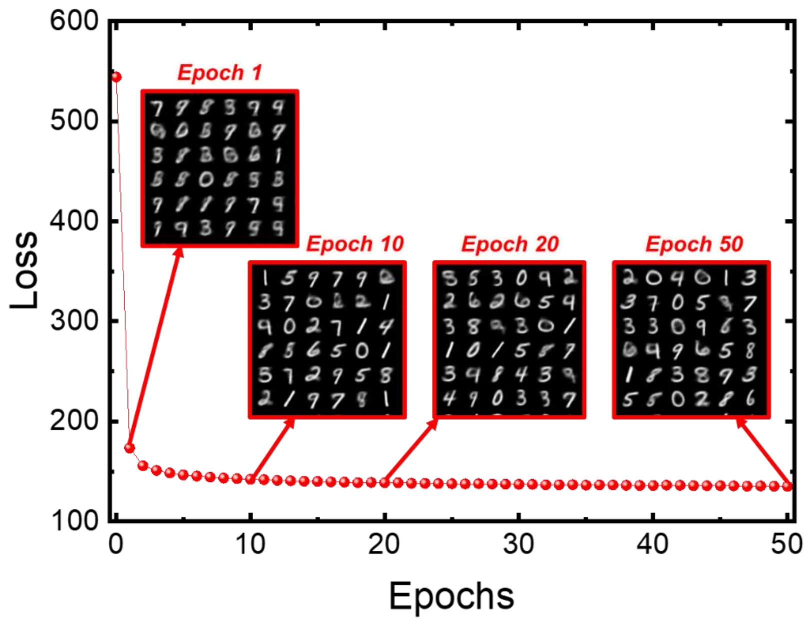

Supplementary Figure 25. Training loss evolution and generated image quality during VAE training on the MNIST dataset. Total training loss (sum of binary cross-entropy and KL divergence) plotted as a function of training epochs, showing rapid convergence within the first few epochs followed by gradual refinement. Insets display representative generated images at epochs 1, 10, 20, and 50, illustrating progressive improvement in digit clarity and diversity as training proceeds.

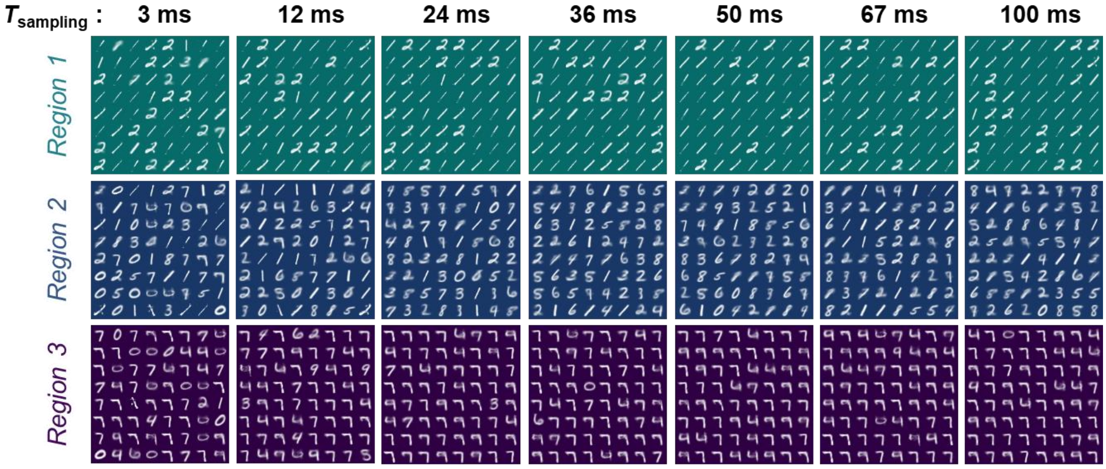

Supplementary Figure 26. Effect of RTN operating region and sampling time on VAE-generated MNIST images. Generated images from the trained VAE using FTJ-based latent vector sampling under three different RTN operating regions (Region 1–3) and varying sampling times. Region 2 produces the most diverse and balanced digit distribution when the sampling time is optimized (e.g., 36 ms), whereas Region 1 and Region 3 show strong biases toward specific digits due to inherent imbalances in the high/low current state probabilities.

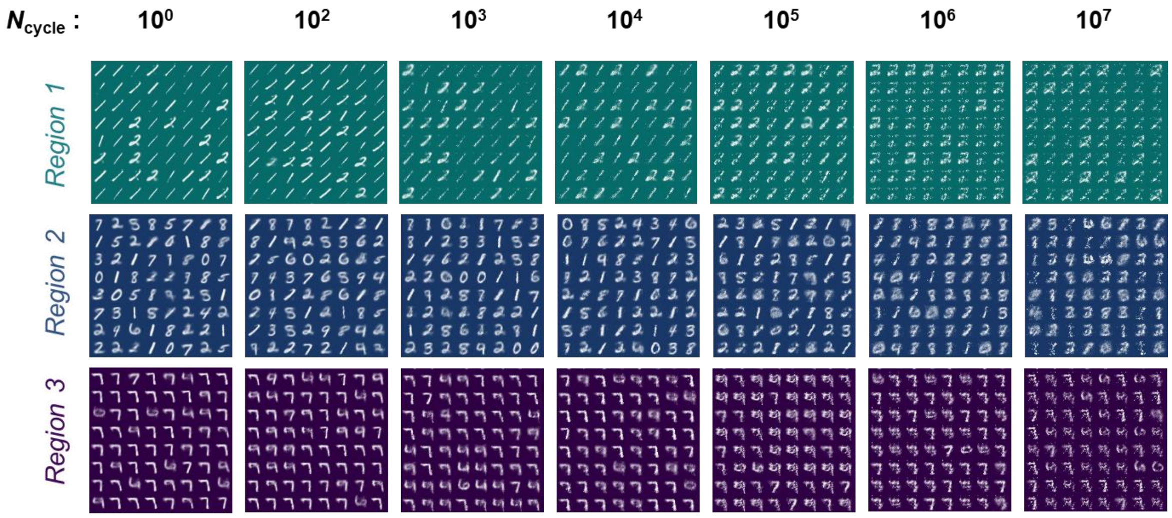

Supplementary Figure 27. Effect of RTN operating region and write cycle count on VAE-generated MNIST images. Generated images from the trained VAE using FTJ-based latent vector sampling under three RTN operating regions (Region 1–3) after different numbers of write cycles applied to the FTJ devices in the decoder array. Region 2 maintains high image diversity and clarity even after extended cycling (up to 10^5 cycles), whereas Regions 1 and 3 exhibit strong bias toward specific digits and degraded generative quality due to inherent current state imbalance.

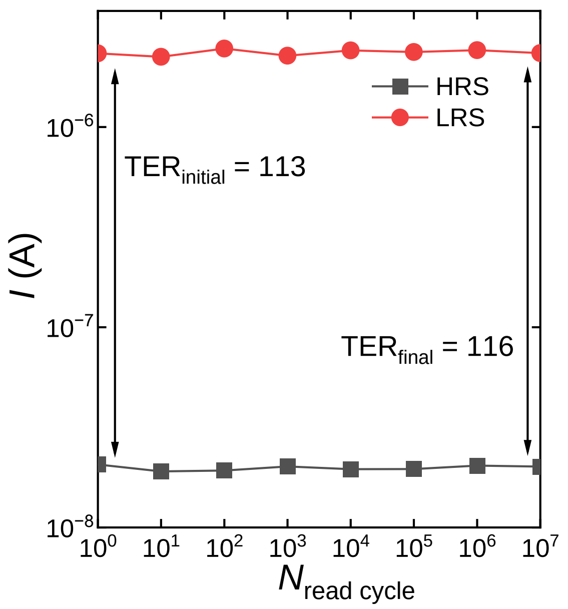

Supplementary Figure 28. Read pulse endurance test. HRS/LRS read current evolution versus the $N_{read\ cycle}$ (V_{read} = 2 V, 10 ms), demonstrating negligible read-induced fatigue.

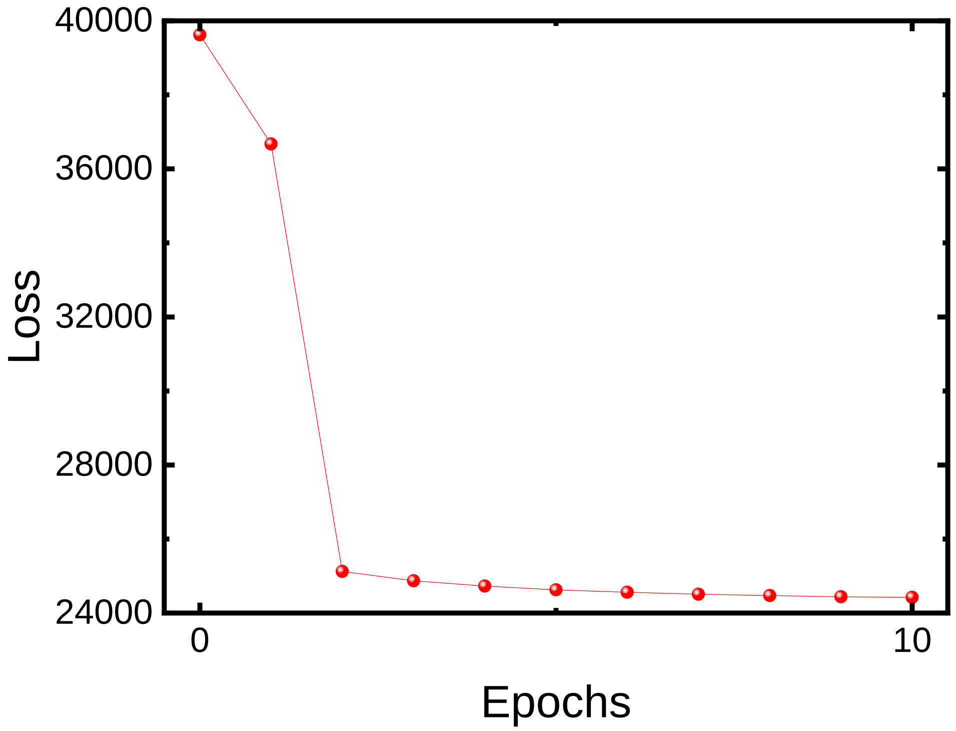

Supplementary Figure 29. Training loss evolution and generated image quality during VAE training on the CelebA dataset. Total training loss (sum of binary cross-entropy and KL divergence) plotted over training epochs. Insets show representative generated face images at epochs 1, 5, and 10, demonstrating progressive improvement in facial detail, diversity, and realism as the training proceeds.

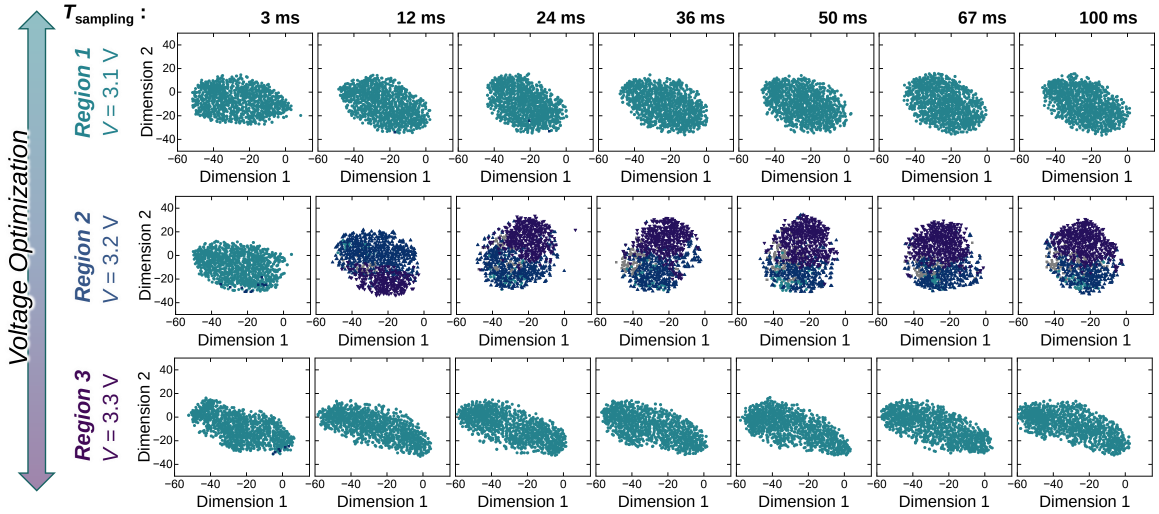

Supplementary Figure 30. *t*-SNE embeddings of 10k generated CelebA images per condition. Rows show Region 1 to Region 3 and columns sweep T_{sampling} from 3 ms to 100 ms. Points are colored by *k*-means clusters defined in PCA space. Layouts are aligned to reveal how distribution coverage and cluster structure vary with region and T_{sampling}.

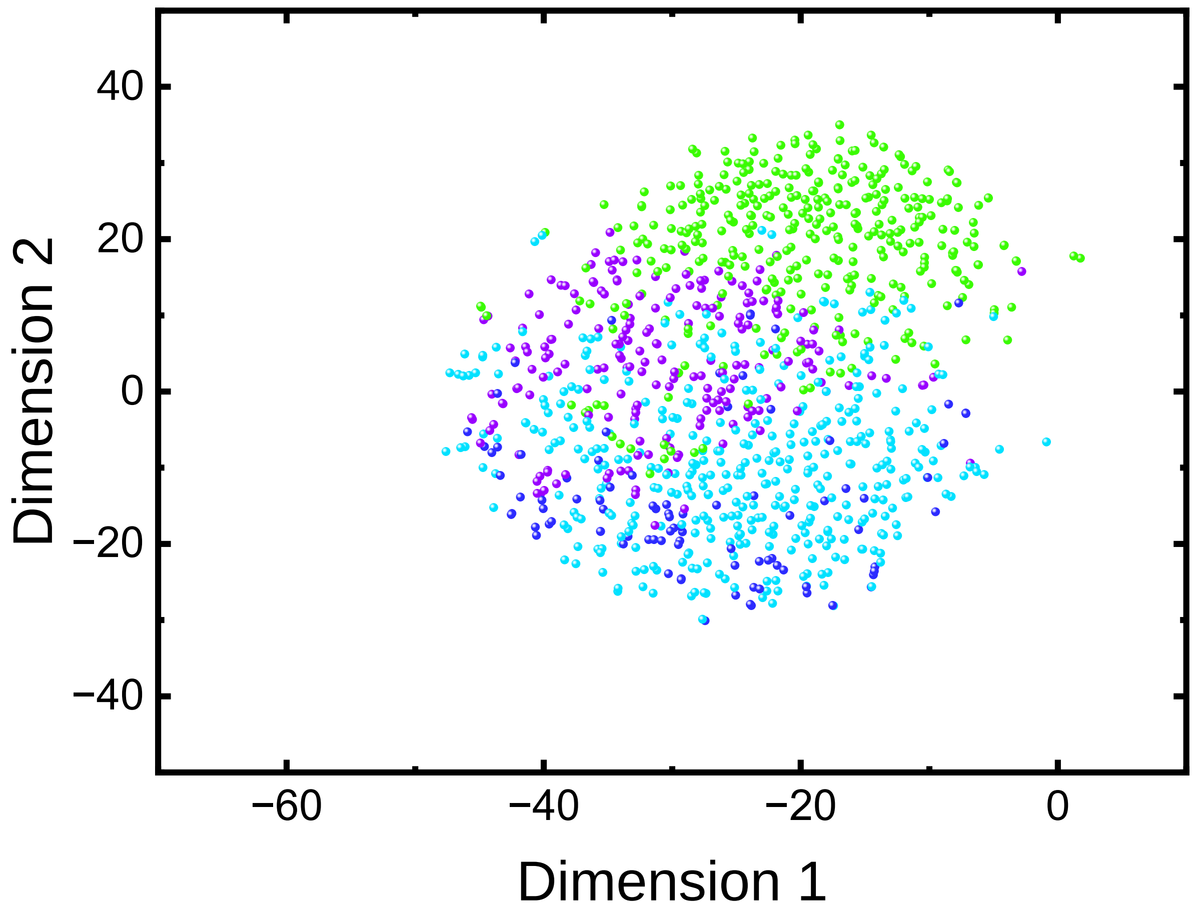

Supplementary Figure 31. *t*-SNE embeddings of ramdomly chosen 10k original CelebA images per condition. Points are colored by *k*-means clusters defined in PCA space.

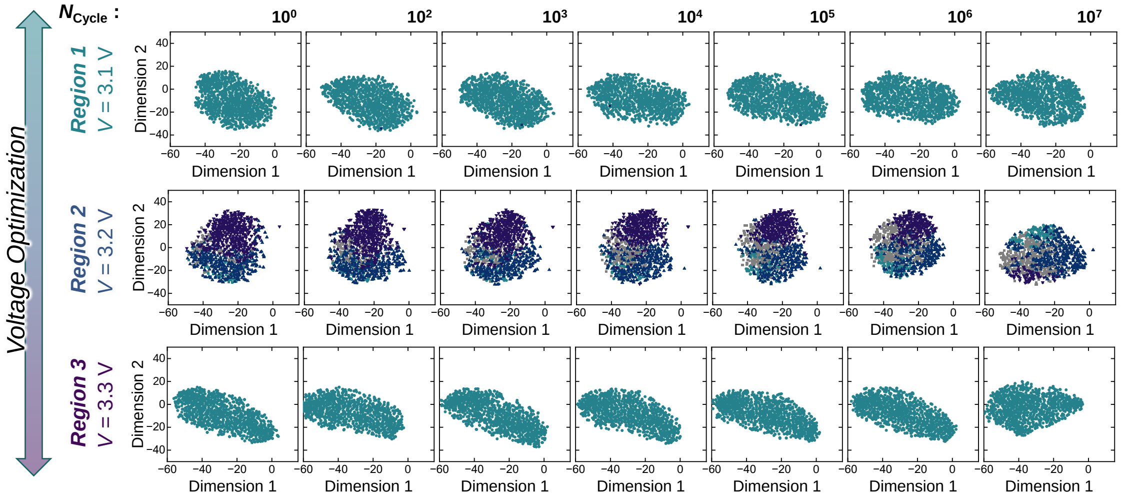

Supplementary Figure 32. *t*-SNE visualization of the effect of RTN operating region and write cycle count on FTJ-based latent vector sampling for CelebA generation. *t*-SNE plots of feature embeddings for CelebA images generated by the trained VAE using FTJ-based latent vector sampling under three RTN operating regions (Regions 1-3) after different numbers of write cycles applied to the FTJ devices in the decoder array. Region 2 preserves the broadest and most balanced embedding distribution even after extended cycling (up to 10^5 cycles), indicating robust diversity and realism, whereas Regions 1 and 3 show biased clustering patterns associated with color and feature imbalance arising from asymmetric high/low current state distributions.

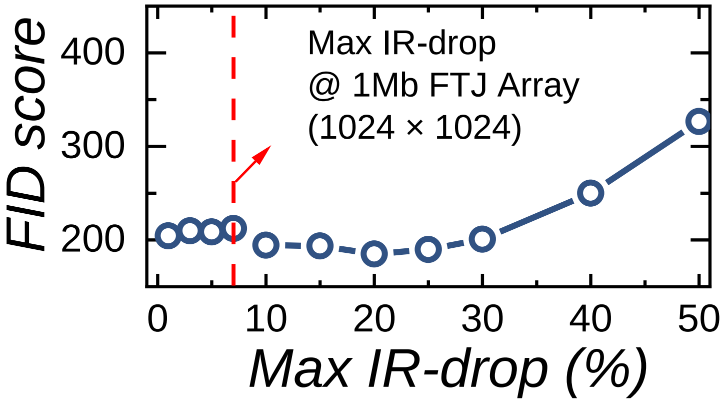

Supplementary Figure 33. FID degrades with increasing IR-drop. Red dashed line marks the worst-case IR-drop for a 1 Mb (1024×1024) FTJ array.

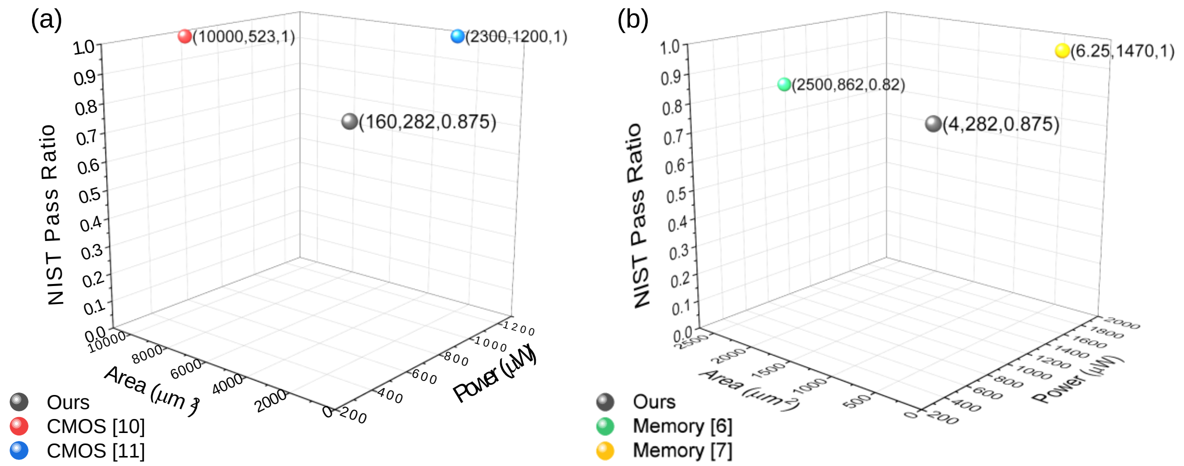

Supplementary Figure 34. Three-dimensional area-power-randomness comparison using area as *x* axis, power as *y* axis, and NIST pass ratio as *z* axis. (a) 3D scatter plot comparing the proposed FTJ sampler with CMOS-based implementations using macro-level area and power that include peripheral overhead. (b) 3D scatter plot comparing cell-level sampling metrics of the proposed FTJ sampler with device-noise-based memory works, using reported cell footprint and stochastic-operation power.

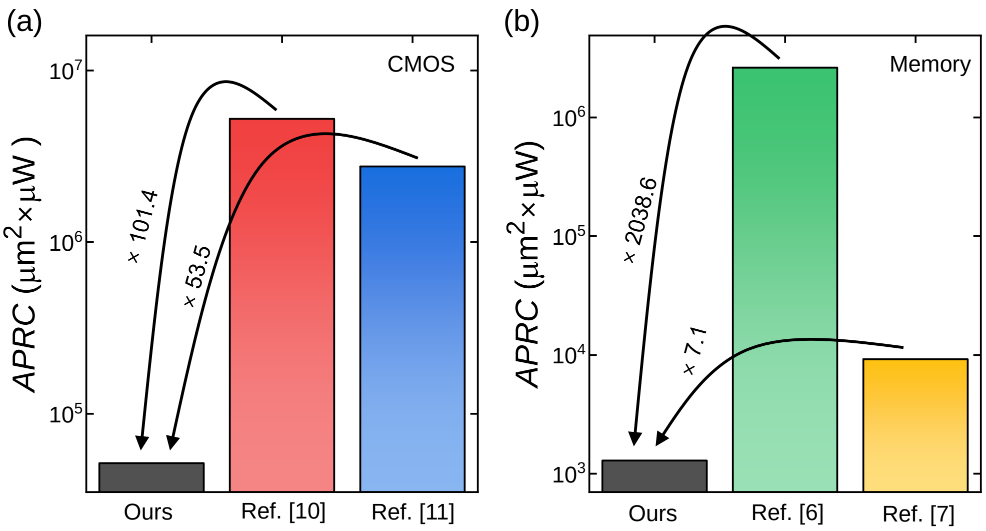

Supplementary Figure 35. Area–Power–Randomness Cost (*APRC*) comparison. (a) *APRC* box plots for the proposed work and CMOS-based implementations. (b) *APRC* box plots for the proposed work and device-noise-based memory implementations.

Supplementary Table

Learning Parameter		
**Dataset**	MNIST	Celeb-A
**Epochs**	50	10
**Learning Rate**	0.001	0.002
**Batch Size**	64	128
**Optimizer**	Adam	

Supplementary Table 1. Learning parameters of the VAE for different datasets. Summary of training configurations for MNIST and CelebA datasets, including network architecture, learning rate, optimizer type, batch size, number of epochs, and latent space dimensionality. These parameters were optimized to achieve stable convergence and high-quality image generation for each dataset.

Latent Vector Mapping Algorithm Using RTN. Total Sampling Iteration N_{sample}, Sampling Time Step t, Sampled Sequence X_t, Minimum value *Min*, Maximum Value *Max*, Latent Vector Z, Latent Vector Dimension D

Requirements: Markov Chain Transistion Matrix (MCTM) reflecting RTN noise transient behavior.

for $i = 0$ **to** D **do**

  $X_0 = 0$ or 1   // Random Initialization

  $Count = 0$

  for $t = 0$ **to** N_{sample} **do**

    $\mathbf{X_{t+1}} \leftarrow Update(\text{MCTM}, X_t)$ // Update X_{t+1} according to MCTM and X_t

  for $t = 0$ **to** N_{sample} **do**

    if $X_t == 1$ **then**

      $Count \leftarrow Count + 1$

  $Z_i \leftarrow Min + (Max - Min) \times Count / N_{sample}$ // Z_i is i-th element of Latent vector Z

Supplementary Table 2. Latent vector mapping algorithm using FTJ RTN. Table describing the process of generating and mapping latent vectors from FTJ-based random telegraph noise (RTN) for use in variational autoencoder (VAE) image generation. The algorithm includes RTN signal acquisition, binary sequence conversion via threshold comparison, bitstream sampling, normalization to the latent space range, and feeding the resulting vectors into the VAE decoder for image synthesis.

Supplementary Note 1. Central limit behavior and Q–Q analysis for 1/*f* and RTN regimes

We analyze the statistics of time integrated read current using a quantile–quantile framework and we connect the observed behavior to the applicability of the central limit theorem (CLT). The method is as follows. Consecutive current samples are grouped into non overlapping blocks of N points and summed to form S_{N}. The sums are standardized as $Z_{\mathrm{N}} = (S_{\mathrm{N}} - \mu_{\mathrm{N}})/(\sigma_N N^{1/2}))$ where μ_{N} and σ_N are the empirical mean and standard deviation of S_{N}. We then plot the empirical quantiles of Z_{N} against the theoretical quantiles of a standard normal distribution. If the integrated signal is effectively Gaussian, the Q–Q points follow a straight line with unit slope. Deviations from linearity reveal non Gaussian tails or correlation that violates the independence assumption required for the central limit theorem.

Figure R17 shows the Q–Q plots for the 1/*f* regime at V = 2.9 V. The integration window is swept from 0.3125 ms to 160 ms. The points align closely with the 45 degree reference over all windows, which indicates rapid convergence toward a normal distribution under time integration. This behavior supports reliable deterministic accumulation during VMM in the 1/*f* regime.

Figures R18, R19, and R20 show the RTN regime at V = 3.1 V, V = 3.2 V, and V = 3.3 V. Each figure sweeps the same set of integration windows. The Q–Q curves display persistent *S* shaped curvature with heavy tails that remain even as the window increases. The curvature indicates that long range temporal correlation survives the integration and that successive samples are not independent. As a result Z_{N} does not converge to a standard normal distribution within practical windows. This behavior is consistent with a two level process whose dwell times are set by τ_{L} and τ_{H} and whose transitions retain memory over many samples.

Taken together these results explain the operating policy used in the manuscript. In the 1/*f* regime the time integration inherent to VMM averages out short range fluctuations so the output approaches its mean and deterministic accumulation is reliable. In the RTN regime the long correlation prevents convergence to a Gaussian distribution within practical windows, so the integrated output retains stochasticity that is useful for latent sampling but not for precise multiplication. For this reason we operate the decoder VMM at low read voltage in the RTN free region (V= 2.0 V) and we use bias near 3.2 V to obtain balanced τ_{L} and τ_{H} for stochastic sampling.

Supplementary Note 2. Detailed explanation of the mapping strategy of the VAE decoder.

Our scheme assumes tiled FTJ macros (Supplementary Note Figure 1) scalable from ~1 kb (32 × 32 array) to 1 Mb (1024 × 1024 array) effective memory capacity. Each real-valued weight is realized by a differential pair of FTJs to encode positive/negative weight values. Inputs are driven on word-lines using pulse-width modulators (PWMs), where pulse duration is proportional to the input value. The resulting bit-line currents are integrated per column, and the integrator outputs are digitized by ADCs to produce the VMM result.

Weights are programmed as FTJ conductance using method [5] with verify-and-adjust pulses until the device falls within the target bin of the quantized weight. Referring to the LTP/LTD characteristics, a single FTJ supports 32 stable conductance levels. Using a differential pair yields 64 effective levels per weight parameter. Input precision is set by the PWM (input DAC) timing resolution. For internal decoder layers, we use 5-bit ADCs, which we found sufficient given the 64-level weight quantization. For the final layer, we use an 8-bit ADC to preserve image detail before de-normalizing to pixel space. This split (5-bit internal / 8-bit output) maintained CelebA image quality while containing area and energy.

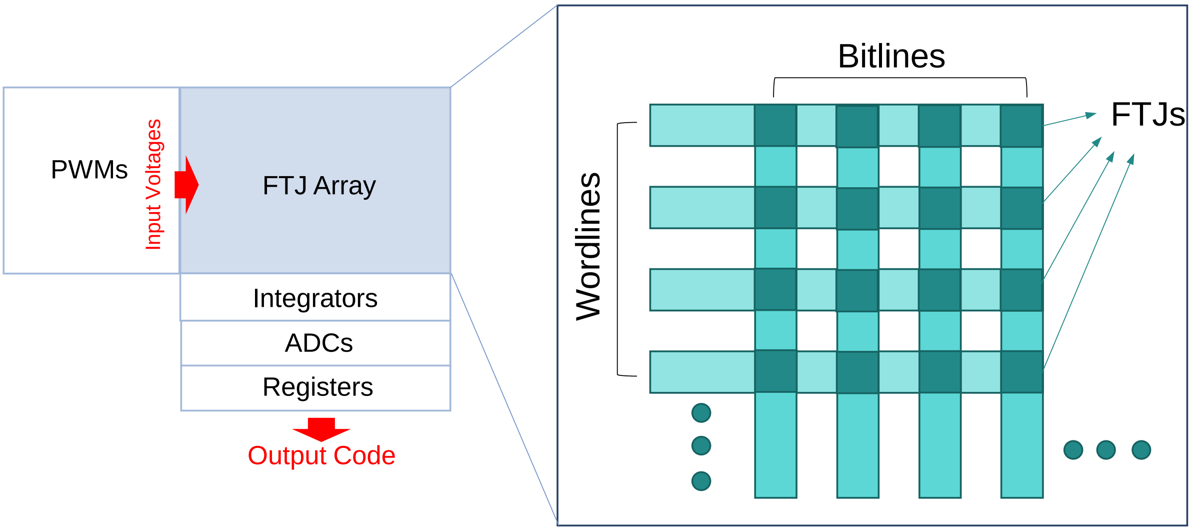

Supplementary Note Figure 1. FTJ cross-bar VMM macro for the VAE decoder. Inputs are applied as voltage pulses on word-lines. bit-line currents are integrated and digitized by ADCs, and the output code is latched in registers.

Supplementary Note 3. Randomness tests and XOR post-processing

We evaluated the statistical quality of the RTN bitstreams using the NIST SP 800-22 test suite. The raw bitstream was obtained directly from the RTN regime without any post-processing. For each condition we collected 40 kbits and applied the standard NIST decision rule with a significance level α equal to 0.01. The representative sampling setting corresponds to the balanced RTN condition used in the main text. This evaluation complements the Markov transition analysis where we quantify the conditional probability of a bit given the previous bit.

Table SN1 reports the NIST SP 800-22 results for the raw bitstream with no post-processing. Once the proportion of zeros and ones is balanced, several tests pass. A few mathematically strict items remain below threshold, which is consistent with the short-range dependence inherent to a two-state RTN process. Fig. S22(a) shows the bit-transition probability between successive bits for this raw case and confirms a small deviation from the ideal value of one half.

To improve independence when required, we applied lightweight XOR post-processing that is widely used in hardware random number generators. We define XOR2 as the exclusive OR of every two consecutive raw bits from the same RTN stream, and XOR3 as the exclusive OR of every three consecutive raw bits from the same stream. Fig. S22(b) and Fig. S22(c) show that increasing the XOR depth progressively drives the transition probability toward one half, indicating suppression of short-range memory. Consistently, Table SN2 and Table SN3 show more NIST items passing for XOR2 and near-saturated improvement for XOR3.

These results should be interpreted in the context of our target application. Our goal is on-chip image generation rather than cryptographic true random number generation. For balanced latent sampling and good image quality, unbiasedness and weak dependence are sufficient. The raw RTN already meets this requirement under the balanced operating point, and XOR post-processing offers a simple and low-overhead path to approach i.i.d.-like behavior when

stronger independence is desired. Tables SN1 to SN3 and Fig. SN1 provide the full numerical summary and transition analysis supporting these conclusions.

No XOR
(Original)

NIST 800-22	P-value	Result
Frequency	0.0	**Fail**
Block Frequency	0.0	**Fail**
Runs	0.0	**Fail**
Longest Runs	0.7439	**Pass**
Matrix Rank	0.6875	**Pass**
Discrete Fourier Transform (DFT)	0.0379	**Pass**
Non-Overlapping Template	0.9999	**Pass**
Overlapping Template	0.0	**Fail**
Maurer's Universal Statistical Test	0.0012	**Fail**
Linear Complexity	0.0	**Fail**
Serial	0.0	**Fail**
Approximate Entropy	0.0	**Fail**
Cumulative Sums (Forward)	0.0	**Fail**
Cumulative Sums (Backward)	0.0	**Fail**
Random Excursions	0.0306	**Pass**
Random Excursions Variant	0.3214	**Pass**

Table SN1. NIST SP 800-22 results for the original bitstream (no XOR).

XOR 2

NIST 800-22	P-value	Result
Frequency	0.0	**Fail**
Block Frequency	0.0	**Fail**
Runs	0.0	**Fail**
Longest Runs	0.2107	**Pass**
Matrix Rank	0.6440	**Pass**
Discrete Fourier Transform (DFT)	0.1772	**Pass**
Non-Overlapping Template	0.9999	**Pass**
Overlapping Template	0.0	**Fail**
Maurer’s Universal Statistical Test	0.0	**Fail**
Linear Complexity	0.0	**Fail**
Serial	0.0	**Fail**
Approximate Entropy	0.0	**Fail**
Cumulative Sums (Forward)	0.0	**Fail**
Cumulative Sums (Backward)	0.0	**Fail**
Random Excursions	0.1188	**Pass**
Random Excursions Variant	0.2650	**Pass**

Table SN2. NIST SP 800-22 results for XOR2 post-processed bitstream.

XOR 3

NIST 800-22	P-value	Result
Frequency	0.6579	Pass
Block Frequency	0.7528	Pass
Runs	0.1228	Pass
Longest Runs	0.5260	Pass
Matrix Rank	0.9603	Pass
Discrete Fourier Transform (DFT)	0.1041	Pass
Non-Overlapping Template	0.9999	Pass
Overlapping Template	0.0	Fail
Maurer's Universal Statistical Test	0.8979	Pass
Linear Complexity	0.0	Fail
Serial	0.2163	Pass
Approximate Entropy	0.2177	Pass
Cumulative Sums (Forward)	0.8687	Pass
Cumulative Sums (Backward)	0.8687	Pass
Random Excursions	0.3290	Pass
Random Excursions Variant	0.2412	Pass

Table SN3. NIST SP 800-22 results for XOR3 post-processed bitstream.

Supplementary Note 4. Detailed Explanation of the VAE training and image generation simulation of the MNIST dataset.

In this study, we employ a VAE to model the distribution of handwritten digits in the MNIST dataset and to generate synthetic samples that reflect the learned manifold [6,7]. The MNIST dataset comprises 60,000 training and 10,000 test images, each of size 28×28 pixels in grayscale. We load both splits using the torchvision.MNIST class with a ToTensor() transform function, which scales pixel intensities to the [0, 1] range. We use a batch size of 64 for both training and evaluation, shuffling the training set at each epoch to ensure stochasticity.

In this study, we employ a VAE to model the distribution of handwritten digits in the MNIST dataset and to generate synthetic samples that reflect the learned manifold [6,7]. The MNIST dataset comprises 60,000 training and 10,000 test images, each of size 28×28 pixels in grayscale. We load both splits using the torchvision.MNIST class with a ToTensor() transform function, which scales pixel intensities to the [0, 1] range. We use a batch size of 64 for both training and evaluation, shuffling the training set at each epoch to ensure stochasticity.

Our VAE architecture for MNIST is a three-layer fully connected network. The encoder begins by flattening each input image into a 784-dimensional vector and then projecting it through two successive linear layers of size 512 and 256, each followed by a ReLU activation. From the 256-dimensional hidden layer, two separate linear layers output the latent mean vector μ and the log-variance vector $\log \sigma 2$, each of dimension 2. We then apply the reparameterization trick by sampling ε from a standard normal distribution and computing $z = \mu + \exp(0.5 \log \sigma 2) \times \varepsilon$, which allows back-propagation through the stochastic sampling step [6,7]. The decoder mirrors the encoder: it maps the 2-dimensional latent code z through linear layers of size 256 and 512 with ReLU activations, and finally reconstructs a 784-dimensional output via a linear layer followed by a sigmoid nonlinearity, producing pixel intensities in [0, 1].

Training proceeds for 50 epochs using the Adam optimizer with a learning rate of 1×10^{-3}. At each iteration, we compute the total loss as the sum of two terms: (1) the binary cross-entropy reconstruction loss (BCE) between the original and reconstructed pixel values, summed over all pixels in the batch; and (2) the Kullback-Leibler divergence (KLD) between the approximate posterior and the unit normal prior, given by $-0.5 \sum(1 + \log \sigma^2 - \mu^2 - \sigma^2)$ [6,7]. Gradients of the combined loss are back-propagated and the network weights are updated accordingly. After each epoch, we switch to evaluation mode and compute the average loss

over the entire test set without gradient updates. This monitoring ensures that the model generalizes beyond the training data and avoids overfitting.

Supplementary Note 5. Detailed explanation of the VAE training and image generation simulation on the CelebA dataset.

We extend our VAE framework from the MNIST digit domain to high-resolution face images drawn from the CelebA dataset [8]. All experiments were implemented in PyTorch and run on GPUs when available. The CelebA dataset consists of over 200,000 celebrity face images, with each image randomly horizontally flipped and resized to 128×128 pixels before conversion to a tensor. We used a batch size of 128 and eight worker threads for data loading, enabling efficient I/O and augmentation on the fly.

We extend our VAE framework from the MNIST digit domain to high-resolution face images drawn from the CelebA dataset [8]. All experiments were implemented in PyTorch and run on GPUs when available. The CelebA dataset consists of over 200,000 celebrity face images, with each image randomly horizontally flipped and resized to 128×128 pixels before conversion to a tensor. We used a batch size of 128 and eight worker threads for data loading, enabling efficient I/O and augmentation on the fly.

Our VAE architecture is a convolutional encoder-decoder augmented with residual blocks [9] to stabilize deep feature extraction. The encoder begins with a 4×4 convolution (stride 2, padding 1) mapping three-channel inputs (128×128) to 64 feature maps ($64 \times 64 \times 64$), each followed by batch normalization and ReLU activations. A residual block preserves identity mapping while adding representational capacity at 64 channels. We then successively downsample to 128 and 256 channels via 4×4 strided convolutions, each paired with batch normalization, ReLU, and another residual block at 128 channels. A final 4×4 convolution brings the spatial resolution to 8×8 with 512 channels; after flattening (producing 32,768 features), a fully connected layer reduces to a 2,048-dimension latent embedding. From this shared representation, two linear projections compute the 256-dimension latent mean vector μ and log-variance vector $\log \sigma^2$.

We trained this network for 10 epochs using the Adam optimizer (learning rate 2×10^{-3}). At each minibatch, the total loss combines a BCE summing reconstruction error over all pixels and a KLD measuring deviation of the approximate posterior $N(\mu, \sigma^2)$ from the unit Gaussian prior. Although an MSE term is also computed in the code, our reported results use only BCE + KLD. After every epoch, we switched to evaluation mode and sampled 64 latent vectors to generate synthetic face images.

Supplementary Note 6. Methodology for *t*-SNE Embedding of CelebA Images

This note describes the procedure used to embed a large set of the generated CelebA images into two dimensions for visualization and analysis. The method is designed to preserve local neighborhood structure in a fixed feature space while remaining scalable and reproducible.

Each image is first mapped to a high dimensional feature vector by a pretrained encoder (ResNet-50 pretrained with ImageNet, which is supported in torchvision module). The classifier head is removed. Every feature vector is normalized to unit length using the L_2 norm. This step reduces scale effects and stabilizes the geometry used in later stages.

Before applying t-SNE, the features are compressed by principal component analysis (PCA) to 128 dimensions. PCA is fit once on the normalized real-base subset, and the same linear projection is then applied to all samples. This linear reduction concentrates signal and suppresses high dimensional noise, which improves the stability and efficiency of the non-linear embedding.

For each point in the PCA space we form a local neighborhood distribution using a Gaussian kernel centered on that point. The kernel bandwidth is chosen separately for each point by a binary search so that the resulting conditional distribution has the target perplexity, which acts as an effective neighbor count. We use a default perplexity of 30 and cap it so it does not exceed about one third of the number of fitted points minus one. The pairwise relations are then symmetrized by averaging the two directional conditionals to produce a single similarity value for each pair. In the 2D space we measure similarity with the Student t-kernel, which assigns higher similarity to nearby points and tapers slowly with distance. The 2D coordinates are initialized from the top principal components and refined for a fixed number of iterations using the standard *t*-SNE update with early exaggeration in the initial phase and an automatic learning rate schedule. For large data, we fit *t*-SNE on a uniform random subset with a fixed seed and place the remaining points by out of sample interpolation. Each held out point is mapped to the inverse distance weighted average of the 2D coordinates of its *k*-nearest fitted neighbors found in the PCA space, with a small positive constant added to distances for numerical stability. The result is a two-dimensional layout that preserves local neighbor structure and is suitable for visualization and downstream analysis.

Supplementary Note 7. Impact of Weight Non-Idealities on CelebA FID

Aggressive weight quantization and device non-idealities can degrade neural network performance when the induced weight or read error exceeds the model tolerance. At the same time, compute-in-memory studies have shown that neural networks can remain robust to low-bit quantization and moderate device noise when the quantization strategy and operating conditions are properly optimized. Motivated by these observations, we extended our CelebA decoder simulations to explicitly incorporate the measured multi-level non-idealities.

Fig. SN7-1 compares the CelebA FID under the optimized latent sampling condition (V = 3.2 V and $T_{sampling}$ = 36 ms) for three decoder-weight settings: (i) an ideal 8-bit baseline, (ii) a device-informed 4-bit quantization that reflects the measured LTP/LTD nonlinearity, and (iii) the same 4-bit mapping with additional conductance variation injected according to the measured spreads in Fig. R2, which corresponds to the reviewer's notion of quantization noise. The FID is 210.4 for the ideal 8-bit case, 218.3 when only the device-informed nonlinear 4-bit mapping is applied, and 227.3 when both the nonlinear mapping and the measured variation are included. This negligible change indicates that, under the optimized latent sampling condition, decoder performance is relatively tolerant to the tested level of 4-bit quantization and conductance variation, consistent with prior compute-in-memory reports that low-bit weight quantization and moderate device noise can be tolerated when the mapping and operating conditions are properly optimized

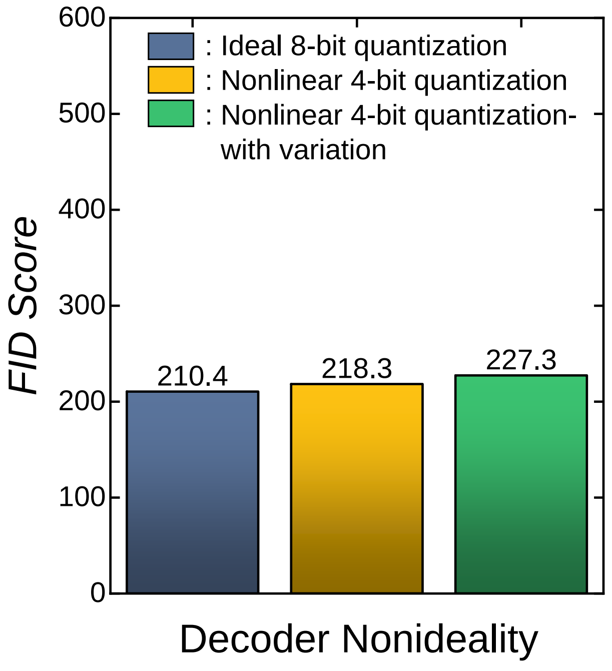

Fig. SN7-1. CelebA FID under decoder weight non-idealities at the optimized sampling condition. CelebA FID comparison at V = 3.2 V and $T_{sampling}$ = 36 ms for three decoder-weight settings: ideal 8-bit weights, device-informed 4-bit mapping reflecting the measured LTP/LTD nonlinearity, and the same 4-bit mapping with additional conductance variation injected according to the measured spreads in Fig. S6.

To clarify what dominates the quality metric, Fig.SN 7-2 shows that even with ideal 8-bit weights, the FID is highly sensitive to the latent sampling regime. Under the same ideal 8-bit weights, the FID is 210.4 in region 2, but it degrades to 456.6 in region 1 and 375.1 in region 3. These results confirm that, in our current framework, the primary bottleneck is the latent sampling distribution governed by RTN statistics. At the same time, the decoder is comparatively robust to the tested weight quantization and variation. We thank the reviewer for raising this important point, and we will continue to improve decoder accuracy and robustness through tighter multi-level programming control and variability reduction in future work.

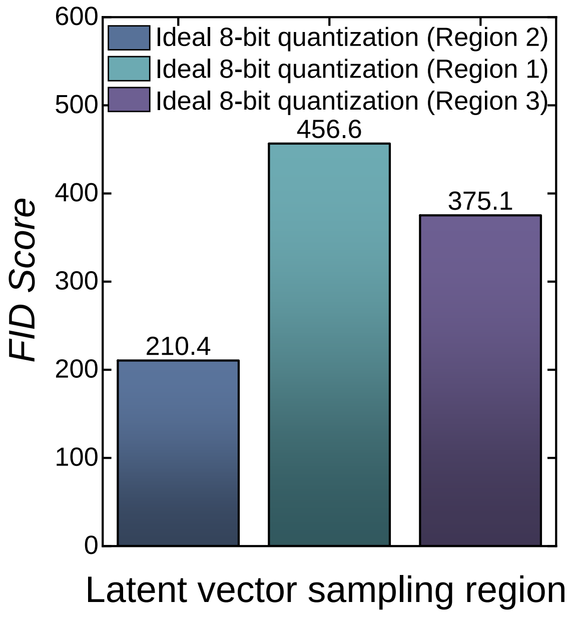

Fig. SN7-2. CelebA FID sensitivity to latent sampling regime with ideal decoder weights. CelebA FID evaluated using ideal 8-bit decoder weights across three latent sampling regimes (regions 1–3). The plot highlights that FID is strongly dependent on the RTN-governed sampling condition and is minimized at the optimized regime (region 2).

Supplementary Note 8. Throughput, array, and energy overhead in terms of RTN Sampling, VMM operation, and ADCs (30 fps, CelebA)

RTN sampling: To sustain 30 fps, a 256-dimensional latent vector must be sampled 30 times per second. Our study uses bitstream sampling with 16 streams, so real-time operation requires 256 × 30 × 16 = 122,880 samplings. Assuming a 36 ms sampling window per FTJ, a single device can provide ~27 samples. Thus ~4,552 FTJs are needed solely for RTN sampling. As software optimization is not our focus, this number can be reduced in practice by lowering the latent dimension or the number of bitstreams.

RTN sampling: To sustain 30 fps, a 256-dimensional latent vector must be sampled 30 times per second. Our study uses bitstream sampling with 16 streams, so real-time operation requires 256 × 30 × 16 = 122,880 samplings. Assuming a 36 ms sampling window per FTJ, a single device can provide ~27 samples. Thus ~4,552 FTJs are needed solely for RTN sampling. As software optimization is not our focus, this number can be reduced in practice by lowering the latent dimension or the number of bitstreams.

VMM operation and minimum arraying: We assume multiple arrays, since a single 2-D array cannot cover the full VAE decoder at once. In our decoder, one 128 × 128 image generation requires about 3.57 Giga-MAC. At 30 fps this is ~100 GOPS (~0.1 TOPS). With 256 × 256 FTJ arrays and differential weights (two devices per parameter for positive/negative value representation), the minimum array count is 413 (256 for fully connected layers and 157 for the transposed convolutional layers) [5]. A 256 × 256 array performs 131,072 operations (MACs) per cycle (counting multiply and add separately as 256 × 256 × 2). Even at a modest 10 MHz operation of the macro, it yields ~1.31 TOPS per array. The different layers demand different workloads (especially convolution). For instance, the last layer with 128 × 128 kernel slides, which is the slowest layer, still meets real-time targets; at 10 MHz the per-inference time is ~1.64 ms, so additional replicated arrays for parallelism are not required.

ADC overhead and read energy: The total ADC energy equals the number of ADC activations per inference and counts the sampling energy. For our network, the ADC operates 23,396,352 times per inference. Assuming 1 pJ/sample [10], this is ~23 μJ total, or ~6.44 fJ per MAC. The read-path energy from synaptic currents, in a worst-case estimate (read time 100 ns, LRS current 2.6 μA, read voltage 2 V, and 3.57 × 10^9 operations with all weights and inputs at 1), is ~1.85 mJ per inference (~600 fJ/MAC). In a more realistic setting, shorter read time (5 ns) [11] and average current ~0.1× LRS from training statistics, the read energy drops to ~9 μJ per inference (~3 fJ/MAC), which is lower than the ADC contribution.

Supplementary Note 9. Definition of APRC and Fair Comparison Methodology

To compare stochastic sampling implementations using area, power, and random quality in a single metric, we define the Area–Power–Randomness Cost as $APRC = (A \times P) / \eta_{\mathrm{NIST}}$. Here A denotes the area of the stochastic sampling hardware, P denotes the power consumption during stochastic operation, and η_{NIST} denotes the NIST SP 800-22 test pass ratio used as a quantitative measure of random quality. With this definition, a smaller APRC indicates a more favorable trade-off because it simultaneously reflects a smaller footprint, lower power, and higher randomness quality.

A practical issue in benchmarking is that prior studies do not report area and power at a uniform level of completeness. CMOS TRNG and random-generator works typically provide a complete circuit implementation, so a macro-level comparison is meaningful because the reported area and power include the peripheral circuitry required to produce a digital random bitstream. In our manuscript, this digital-circuit comparison corresponds to Refs. 1 and 2. By contrast, many device-noise-based memory works report only the device or cell footprint and the device-level power during stochastic operation, while omitting a complete sampling and digitization circuit scheme. In such cases, a macro-level area comparison would require arbitrary assumptions about unreported overhead, which would reduce transparency. In our manuscript, this memory-based comparison corresponds to Refs. 3 and 4. We therefore separate the comparison into two groups and report APRC at the macro level for the CMOS group and at the cell level for the memory group. This separation is intended to keep the comparison fair and technically consistent given what each class of work reports.

For the digital-circuit group, we explicitly include the peripheral overhead required for stochastic sampling in our FTJ system so that the comparison is performed at the same macro level as CMOS implementations. Specifically, the area A includes the DAC area and the sampling sense-amplifier area, both estimated under a 40 nm CMOS technology, and the corresponding power consumption P is included in the same manner. Using this macro definition, our FTJ scheme uses $A = 160\ \mu\mathrm{m}^2$, $P = 282\ \mu\mathrm{W}$, and $\eta_{\mathrm{NIST}} = 0.875$. Ref. 1 reports $A = 10{,}000\ \mu\mathrm{m}^2$, $P = 523\ \mu\mathrm{W}$, and $\eta_{\mathrm{NIST}} = 1$, while Ref. 2 reports $A = 2{,}300\ \mu\mathrm{m}^2$, $P = 1{,}200\ \mu\mathrm{W}$, and $\eta_{\mathrm{NIST}} = 1$. The resulting $APRC$ values are $51{,}566\ \mu\mathrm{m}^2\cdot\mu\mathrm{W}$ for this work, $5{,}230{,}000\ \mu\mathrm{m}^2\cdot\mu\mathrm{W}$ for Ref. 1, and $2{,}760{,}000\ \mu\mathrm{m}^2\cdot\mu\mathrm{W}$ for Ref. 2.

For the digital-circuit group, we explicitly include the peripheral overhead required for stochastic sampling in our FTJ system so that the comparison is performed at the same macro

level as CMOS implementations. Specifically, the area A includes the DAC area and the sampling sense-amplifier area, both estimated under a 40 nm CMOS technology, and the corresponding power consumption P is included in the same manner. Using this macro definition, our FTJ scheme uses A = 160 μm^2, P = 282 μW, and η_{NIST} = 0.875. Ref. 1 reports A = 10,000 μm^2, P = 523 μW, and η_{NIST} = 1, while Ref. 2 reports A = 2,300 μm^2, P = 1,200 μW, and η_{NIST} = 1. The resulting *APRC* values are 51,566 μm^2·μW for this work, 5,230,000 μm^2·μW for Ref. 1, and 2,760,000 μm^2·μW for Ref. 2.

For the memory-based group, we use cell-level area and the reported stochastic-operation power because circuit overhead is not consistently reported in the literature. Under this cell-level definition, our FTJ cell uses A = 4 μm^2 with P = 282 μW and η_{NIST} = 0.875. Ref. 3 reports A = 2,500 μm^2, P = 862 μW, and η_{NIST} = 0.82, while Ref. 4 reports A = 6.25 μm^2, P = 1,470 μW, and η_{NIST} = 1. The resulting *APRC* values are 1,289 μm^2·μW for this work, 2,628,049 μm^2·μW for Ref. 3, and 9,188 μm^2·μW for Ref. 4. Ref. 12 is excluded from the APRC comparison because η_{NIST} is not reported.

Fig. S34 visualizes these comparisons as three-dimensional scatter plots with A, P, and η_{NIST} as the axes, where the desirable region corresponds to low area, low power, and high η_{NIST}. Fig. S35 summarizes the same results using *APRC* for direct numerical comparison within each reporting-consistent group.

Supplementary References

[1] Pamula, V. R. et al. A 65-nm CMOS 3.2-to-86 Mb/s 2.58 pJ/bit highly digital true-random-number generator with integrated de-correlation and bias correction. IEEE Solid-State Circuits Lett. 1, 237-240 (2018).

[2] Kim, J. & Chae, H. A 10-Gb/s true random number generator using ML-resistant middle square method. IEEE J. Solid-State Circuits 59, 2321-2329 (2024).

[3] Li, X. et al. Random Telegraph Noise in Metal-Oxide Memristors for True Random Number Generators: A Materials Study. Adv. Funct. Mater. 31, 2102172 (2021).

[4] Song, M. S. et al. Optimization of Random Telegraph Noise Characteristics in Memristor for True Random Number Generator. Adv. Intell. Syst. 5, 2200358 (2023).

[5] Peng, X., Liu, R. & Yu, S. Optimizing Weight Mapping and Data Flow for Convolutional Neural Networks on Processing-in-Memory Architectures. IEEE Trans. Circuits Syst. I Regul. Pap. 67, 4 (2020).

[6] Kingma, D. P. & Welling, M. Auto-encoding variational Bayes. arXiv preprint arXiv:1312.6114 (2013).

[7] Doersch, C. Tutorial on variational autoencoders. arXiv preprint arXiv:1606.05908 (2016).

[8] Liu, Z., Luo, P., Wang, X. & Tang, X. Deep learning face attributes in the wild. Proc. IEEE Int. Conf. Comput. Vis. 3730-3738 (2015).

[9] He, K., Zhang, X., Ren, S. & Sun, J. Deep residual learning for image recognition. Proc. IEEE Conf. Comput. Vis. Pattern Recognit. 770-778 (2016).

[10] Jiang, H., Li, W., Huang, S. & Yu, S. Analog-to-Digital Converter Design Exploration for Compute-in-Memory Accelerators. IEEE Des. Test 39, 48-55 (2022).

[11] Chen, P., Peng, X. & Yu, S. DNN+ NeuroSim: An end-to-end benchmarking framework for compute-in-memory accelerators with versatile device technologies. 2017 IEEE Int. Electron Devices Meet. (IEDM) (2019).

[12] Liu, B. et al. Bi2O2Se-based bimode noise generator for the application of generative adversarial networks. ACS Appl. Mater. Interfaces 15, 49478-49486 (2023).
