# Supplementary material for: CMOS-compatible ferroelectric tunnel junctions integrate stochastic sampling and deterministic computing for image generation
Source: Nat Commun. 2026 May 8;17:6236. doi: 10.1038/s41467-026-72969-6 (PMC13369162; doi:10.1038/s41467-026-72969-6)
Supplement: Supplementary file 2 — Transparent Peer Review file [file 41467_2026_72969_MOESM2_ESM.pdf]

Peer Review File

CMOS compatible ferroelectric tunnel junctions integrate stochastic sampling and deterministic computing for image generation

Corresponding Author: Professor Jong-Ho Lee

This file contains all reviewer reports in order by version, followed by all author rebuttals in order by version.

Version 0:

Reviewer comments:

Reviewer #1

(Remarks to the Author)
The manuscript is clearly written, the experiments are carefully executed, and the results are convincing for a proof-of-concept device. Nevertheless, major revisions are required to clarify the underlying physics and quantify system-level scalability. Addressing the points below will significantly strengthen the paper and justify its claims of “unified” and “scalable” on-chip generation.

1.Is the generation of RTN noise and the corresponding defect characteristics really regulated by ALD? If so, please explain how to achieve controllability?
2.Please report standard randomness tests (NIST SP 800-22 or TestU01) on the raw bitstreams. State whether the sequences pass i.i.d. and unpredictability metrics.
3.Please discuss the impact of noise background on bit-error-rate when reference current Iref drifts owing to temperature or cycling.
4.The decoder is still emulated on an external GPU. Please provide a complete mapping strategy (weight programming, ADC/DAC resolution, area, and energy) for implementing both encoder and decoder in a 1kb~1Mb FTJ cross-bar array.
5.Estimate minimum array size required for real-time (30 fps) 128×128 CelebA generation, including: TOPS required for 256×64k VMM,ADC energy (pJ per MAC) and RTN sampling latency overhead.
6.Please discuss non-idealities (IR-drop, sneak path, write disturb) and their effect on VMM linearity and FID degradation.
7.It could be useful to expand Table 1 into a quantitative comparison of energy and area efficiency.
8.Fig. 7h shows FID saturation at 10^5 cycles. Please verify whether this is limited by FTJ on/off ratio degradation, or external GPU numerical precision.
9.Please provide conductance distribution histograms (LRS/HRS and intermediate states) after 10^3, 10^5, and 10^6 cycles to directly link device drift to FID increase.
10.It could be useful to superimpose the t-SNE or UMAP embedding plots of 10,000 generated images on top of the real data in Figures 7d and 8d, in order to visually quantify the degree of distribution overlap.
11.It could be useful to add Inception Score (IS) curves alongside FID for dual-metric convergence.
12.Please specify read-pulse amplitude/width used during VMM.
13.Is there an error in the time unit of the PUND image in Figure 3b? Can the ferroelectric polarization reversal process last for such a long time?
14.When the reading voltage exceeds 3.4V, does the RTN still exist?
15.In the manuscript, the author has cited some references to explain the process of the transition between 1/f noise and RTN noise. Could some additional explanations be provided in the main text to facilitate readers' understanding?
16.Why does the frequency in Figure 4c increase from 100Hz to 1000Hz?
17.Some recent advances about the application of noise in ferroelectric memory should be included, such as:
[1] Y. Huang, Q. Wu, T. Gong, J. Yang, Q. Luo, M. Liu, Nature Communications 2025, 16.
[2] M. Song, R. H. Koo, J. Kim, C. H. Han, J. Yim, J. Ko, S. Yoo, D. H. Choe, S. Kim, W. Shin, D. Kwon, Nat Commun 2025, 16, 6879.

Reviewer #2

(Remarks to the Author)
This paper presents an array-level demonstration of an FTJ-based design that supports both stochastic sampling for latent-variable inference and deterministic computing for vector matrix multiplication. The key contribution is the circuit-level demonstration and analysis of the RTN effect on latent sampling. Moreover, the voltage-dependent behavior within FTJ is intrinsic and matches the features of stochastic and deterministic computing.

Strengths
1. Demonstrating stochastic and deterministic operations on the same FTJ array is compelling and reduces system complexity.
2. Directly connecting RTN characteristics to sampling behavior provides a useful bridge between device physics and VAE tasks.
3. The experiments include a thorough evaluation across different voltages and conditions.

Here are a few questions and comments:
1. The dual functionality of the FTJ has been successfully demonstrated, and the analysis of the optimal operating voltage for effective VAE performance (i.e., balanced tau_h and tau_l) is shown in Figures 5 and 6 (particularly 5c and 6d). Does the 3.2 V result from the trap analysis generally apply to the entire 20 *20 array? Are there notable cell-to-cell variations within the array? If across different arrays, will this voltage result fluctuate?
2. As discussed in the paper, Figure 4a and 4c indicate that I<10nA can be used for muliplition. Could the author explain more why the corresponding low-frequency noise can be ignored by the computation? Is there a threshold for S_I/(I^2) or gamma that indicates that the 1/f noise is not dominate?
3. The VAE used for MNIST generation (Supplementary Figure 10) contains fully connected layer with more than 400 parameters. How is parameter mapping managed in the proposed design?
4. According to the Supplementary Table, training on the CelebA dataset requires about 1,562 iterations per epoch and 10 epochs total, exceeding 10^5 cycles overall. How can the FTJ design accommodate such a large number of training iterations?
5. The latency and power of the random-sampling process could be compared with prior art, such as CMOS-based or ReRAM-based random number generators/sampling process.
6. The reference [6] has a minor error in the publication venue.

Reviewer #3

(Remarks to the Author)
I co-reviewed this manuscript with one of the reviewers who provided the listed reports. This is part of the Nature Communications initiative to facilitate training in peer review and to provide appropriate recognition for Early Career Researchers who co-review manuscripts.

Reviewer #4

(Remarks to the Author)
Review of NCOMMS-25-67053-T “CMOS-Compatible FTJ Hardware Unifying Stochastic Sampling and Deterministic Computing for On-Chip Image Generation”

Summary
The manuscript reports a CMOS-compatible ferroelectric tunnel junction (FTJ) platform that unifies stochastic sampling and deterministic vector–matrix multiplication (VMM) within the same device type. By exploiting voltage-controlled random telegraph noise (RTN) at high current for stochastic latent vector generation, and multilevel ferroelectric conductance at low current for deterministic VMM, the authors design a hardware-native variational autoencoder (VAE). They demonstrate wafer-scale fabrication of 20×20 FTJ arrays, extensive device-level characterization, circuit-level random bitstream generation, and application-level validation on MNIST and CelebA image synthesis tasks.
This is a wide-ranging work spanning materials, device physics, circuits, and system demonstrations. It addresses a challenge in hardware generative AI, viz. reconciling the opposing requirements of stochastic sampling and deterministic decoding in a scalable hardware platform.

Major points
1. Scope of innovation and positioning
o The core novelty is apparent, yet the manuscript should articulate more distinctly how it advances beyond prior demonstrations of memristor-based stochastic sampling and in-memory generative models (e.g., Song Nat. Commun. 2025; Ding Commun. Mater. 2025; Lin Nat. Mach. Intell. 2023; Wang Sci. Adv. 2025).
o Table 1 mixes specific materials and categories; it should be reorganized by device types, with consistent terminology (ReRAM/RRAM, FTJ) and comparisons that explicitly cover stochastic sampling mechanisms, noise utilization stages, and integration strategies.

2. Use of FTJ cells for stochastic vs deterministic modes
o It remains unclear whether the same physical cells can actively toggle between stochastic and deterministic roles during

one inference, or whether subsets of the array are fixed/dedicated to each function. This distinction matters because reprogramming trained weights repeatedly may not be feasible or cost-effective at all. Please clarify the system-level usage strategy and (re)programming accuracy, complexity and overhead.

3. Deterministic FTJ precision / resolution
o The deterministic VMM demonstrations show gradual conductance tuning, but the reproducible number of levels or effective bits per cell is never specified. Are the decoders assumed to use full-precision analog weights, or a limited multi-bit resolution? Explicit quantification would allow readers to judge the scalability to larger neural networks. E.g., commercial RRAM cells are 1-bit and never analog. In this regard, I'm not sure how to read Fig. 3h, how can I know how many bits/cell?

4. Sampling speed and throughput
o The optimal sampling time of ≈36 ms is several orders of magnitude slower than digital RNG or alternative memristor noise sources. The throughput implications should be discussed, including how many samples are required to generate a single MNIST or CelebA image, and whether architectural modifications (e.g. parallelism) could overcome this bottleneck.

5. Power and endurance
o Stochastic operation requires biasing into a high-current regime. Please report typical current levels and estimate the energy per random bit/sample, and compare to CMOS or in-memory RNG baselines. Also discuss whether the reported endurance (~10^5 cycles) suffices for practical inference workloads that repeatedly require stochastic sampling.

6. Comparisons with digital approaches
o The manuscript argues CMOS/GPU alternatives are less efficient, but provides no quantitative evidence. Thorough comparisons in area efficiency, power per sample, or latency seem necessary to justify your claims.

7. Scalability and variability
o Memristive device variability is a key concern. While reproducibility data across 10–20 devices are encouraging, a more explicit discussion on:
- how variability distorts latent distributions or biases image outputs;
- potential IR-drop, routing, or yield issues when arrays scale beyond 20×20;
- implications for manufacturing scalability using industry HfO2 processes.

8. Presentation of noise characterization
o Figures 4–5 contain highly detailed RTN analyses. Consider moving some of these results to the Supplementary, emphasizing instead the clear link from trap physics -> controllable stochasticity -> image generation quality.
9. Clarity of system integration
o A computational flow diagram showing explicitly how stochastic sampling outputs are combined with deterministic weight arrays through the VAE pipeline would largely improve accessibility, especially for a broad NC readership.

Minor points
• Ensure consistency of terminology in Table 1 and throughout the text (ReRAM vs RRAM; device categories vs specific materials).
• Several typographical issues should be corrected, e.g. "In constrast" → "In contrast" (p.16, line 400).
• σ_output N could be better explained in figure captions for readers less familiar with this metric.

Recommendation
The manuscript is promising and potentially impactful. However, given the missing clarifications on FTJ cell usage, precision, energy/latency trade-offs, and scalability, I recommend a major revision. Addressing these points will strengthen the positioning of this work and ensure it withstands scrutiny from both the materials and systems communities.

Version 1:

Reviewer comments:

Reviewer #1

(Remarks to the Author)
The authors have made a substantial effort to address the initial concerns. Several issues should be addressed to fully meet the journal's standards：

1. Please translate the 1 mV per-cell trimming scheme into hardware cost. If each column needs a ≥10-bit DAC, the area could easily outweigh the FTJ array itself.

2. Fig. 3h only verifies binary (LRS/HRS) linearity. To justify "4-bit (16-state)" weights, please report the programmed 16 uniformly spaced conductance levels, and discuss how this quantisation noise affects CelebA FID compared to ideal 8-bit weights.

3. Please clarify whether "reading pulses cause fatigue" or only "writing pulses are counted as 10^5". If the endurance of

reading pulses is not limited, please provide the read disturbance experiment data.

4. Please use the three-dimensional indicators of area-energy-random quality to directly compare the superiority coefficients of the FTJ scheme with digital circuits.

Reviewer #2

(Remarks to the Author)
All my comments have been addressed, and the revision looks good to me. I do not have further comments on the revised manuscript.

Reviewer #3

(Remarks to the Author)
I co-reviewed this manuscript with one of the reviewers who provided the listed reports. This is part of the Nature Communications initiative to facilitate training in peer review and to provide appropriate recognition for Early Career Researchers who co-review manuscripts.

Reviewer #4

(Remarks to the Author)
Thanks for the revision. In this round, the authors have provided an extensive and high-quality revision that addresses nearly all major concerns raised in the first round. The manuscript is technically strengthened, better organized, and much clearer in presentation.

Summary of improvements
- The distinction between stochastic and deterministic operation of the FTJ array is now explicit: a small region operates in the RTN window (~3.2 V) for stochastic sampling, while the remaining majority performs deterministic VMM decoding at 2 V without weight reprogramming.
- Deterministic precision is quantified at an effective 4-bit (16 state) resolution per FTJ, extended to 64 levels per differential pair, supported by measured LTP/LTD parameters.
- Wafer-scale ALD uniformity, per-device bias trimming, endurance, and IR-drop tolerance are demonstrated through new experimental data (Figs. R1–R5, R9).
- Throughput, area, and energy efficiency are analyzed quantitatively in Supplementary Note 7, while Table 1 has been expanded to benchmark FTJ performance against CMOS and ReRAM implementations. Figures have been reorganized, and a clear flow diagram has been added for system readability.

Minor residual points
1. The 36 ms sampling latency and associated hardware overhead (~4,552 FTJs for 30 fps) are discussed only in Supplementary Note 7. I suggest briefly acknowledging this trade-off in the main Discussion section.
2. The comparison works in updated Table 1 (Refs. 5-11) remain relatively dated (2017-2023). Tables 2-3 in "Transforming memristor noises into computational innovations" (Ding et al., Commun. Mater. 2025) may help identify more recent ReRAM noise-based computing works for comparison.

Version 2:

Reviewer comments:

Reviewer #1

(Remarks to the Author)
The authors have satisfactorily addressed all my concerns. The manuscript is now technically solid and presents a complete story from device physics to system-level benchmarking. I recommend the paper for publication in Nature Communications.

Reviewer #4

(Remarks to the Author)
The authors have further strengthened the manuscript through clear experimental validation, quantitative analyses, and transparent discussion. Key improvements include the realistic hardware assessment of the trimming scheme, addition of multi-level programming statistics and quantization-noise impact on CelebA performance, experimental confirmation of read-cycle endurance, and a comprehensive area-power-randomness comparison. The discussion now also acknowledges throughput and sampling-latency trade-offs. Overall, the revision is technically sound & well posed, and satisfactorily addresses all key concerns. I support the acceptance of this manuscript.

Open Access This Peer Review File is licensed under a Creative Commons Attribution 4.0 International License, which permits use, sharing, adaptation, distribution and reproduction in any medium or format, as long as you give appropriate credit to the original author(s) and the source, provide a link to the Creative Commons license, and indicate if changes were made.
In cases where reviewers are anonymous, credit should be given to 'Anonymous Referee' and the source.
The images or other third party material in this Peer Review File are included in the article's Creative Commons license, unless indicated otherwise in a credit line to the material. If material is not included in the article's Creative Commons license and your intended use is not permitted by statutory regulation or exceeds the permitted use, you will need to obtain permission directly from the copyright holder.
To view a copy of this license, visit https://creativecommons.org/licenses/by/4.0/

Revision and Correction (NCOMMS-25-67053-T)

Thank you again for submitting your manuscript "CMOS-Compatible FTJ Hardware Unifying Stochastic Sampling and Deterministic Computing for On-Chip Image Generation" to Nature Communications. We have now received reports from 4 reviewers and, after careful consideration, we have decided to invite a major revision of the manuscript.

As you will see from the reports copied below, while the reviewers find your work of interest, they raise important concerns. Reviewer #2, #3 and #4 raise concerns regarding the variability of the FTJ devices, including the operating voltage. Reviewer #4 is concerned about the switching between stochastic and deterministic roles during inference. Besides, we would also suggest you to follow reviewer #4`s comment and benchmark your work properly in Table 1. We find that these concerns limit the strength of the study, and therefore we ask you to address them with additional work, specifically with extensive supporting experiments and discussion to better substantiate your claims. Without substantial revisions, we will be unlikely to send the paper back to review.

If you feel that you are able to comprehensively address the reviewers' concerns, please provide a point-by-point response to these comments along with your revision. Please show all changes in the manuscript text file with track changes or color highlighting. If you are unable to address specific reviewer requests or find any points invalid, please explain why in the point-by-point response.

Dear Reviewers,

We, the authors, appreciate the time and effort that the reviewers dedicated to providing feedback on our manuscript and are grateful for the insightful comments on our work. We read the comments and questions carefully and prepared a response sheet for them. Please refer to the responses below. The responses that need to be addressed in the revised manuscript are highlighted so that the reviewers can trace them with more ease. Also, the overall sentences have been refined with more concise expressions and better English grammar. We heartily thank all the comments and coordination again.

Reviewer #1

The manuscript is clearly written, the experiments are carefully executed, and the results are convincing for a proof-of-concept device. Nevertheless, major revisions are required to clarify the underlying physics and quantify system-level scalability. Addressing the points below will significantly strengthen the paper and justify its claims of "unified" and "scalable" on-chip generation.

Response:

We, the authors, are very much thankful for the detailed comments and instructive suggestions. We have revised the manuscript in light of the useful comments and suggestions of the reviewer. Each comment and suggestion has been followed and addressed in the response attached below.

We, the authors, send the deepest appreciation for the encouragement and comments again.

1.Is the generation of RTN noise and the corresponding defect characteristics really regulated by ALD? If so, please explain how to achieve controllability?

Response:

Thank you for your comment. As the reviewer pointed out, clarifying whether ALD regulates RTN and explaining how controllability is achieved is important. Fig. R1(a) shows an ellipsometry thickness map of ALD HZO measured at 49 points across a 6-inch wafer, while Fig. R1(b) plots thickness versus radial distance from the wafer center. The data confirm high wafer-scale uniformity with only a slight decrease in thickness toward the edge, and the positions of the ten devices (D1–D10) used for RTN characterization are indicated on the map.

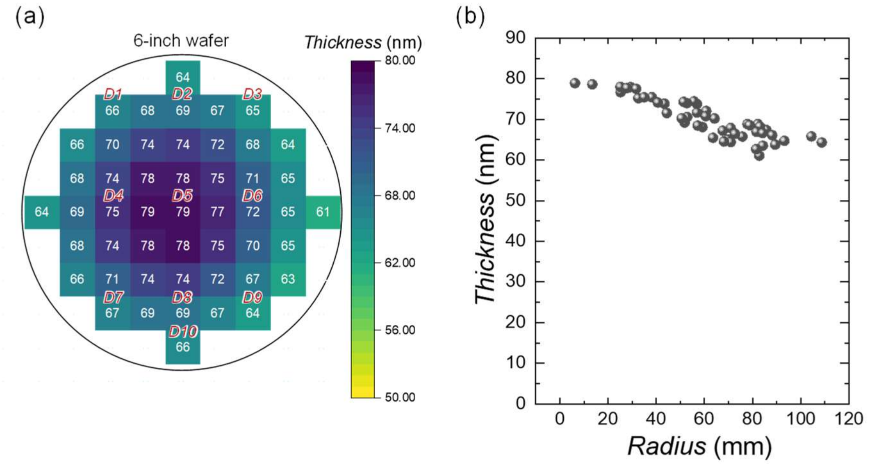

Fig. R1. Wafer-scale thickness uniformity of ALD HZO. (a) Six-inch wafer map showing ellipsometry-measured HZO thickness at 49 sampled points. The locations of the 10 devices (D1 to D10) used for RTN characterization are also marked on the map. (b) Thickness versus radial distance from the wafer center, showing a slight decreasing trend toward the edge.

Fig. R2(a) and Fig. R2(b) then show, for these ten devices biased at $V = 3.28$ V, the histograms of τ_L and τ_H with Poisson fits, indicating a robust two-state RTN process with nearly balanced occupancy at this bias. Fig. R3(a) shows the applied read voltage per device used for the measurements, and Fig. R3(b) shows the fitted time constants τ_L and τ_H per device at the same bias. A small but finite spread is observed, which we attribute to ALD-related variations in trap depth and energy level. In our view, ALD deposition statistically confines the distribution of RTN-active defects across the wafer, but it does not allow deterministic placement or complete suppression of individual traps, and therefore cannot be used to "perfectly" control the RTN generation itself. Instead, we use this process-induced uniformity as a baseline and pursue practical controllability of the RTN statistics at the circuit level. These minor differences did not affect CelebA generation performance in our study, although tighter control may be needed for more precision-demanding tasks. Motivated by this, we devised and validated a simple electrical calibration method during the revision.

To verify this controllability, we fine-tuned the read bias in 0.001 V steps based on the imbalance between τ_L and τ_H. When τ_L exceeded τ_H, V was increased by 0.001 V; when τ_H was larger, V was reduced by 0.001 V. This

adjustment was repeated until both dwell times converged within tolerance. Fig. R4(a) shows the τ_L histograms after per-device equalization within 3.25 to 3.30 V, and Fig. R4(b) shows the corresponding τ_H histograms, both exhibiting more balanced occupancy than in Fig. R2. Fig. R5(a) summarizes the optimized bias for each device after equalization, and Fig. R5(b) shows the fitted τ_L and τ_H values under these optimized conditions, now closely clustered compared with Fig. R3(b).

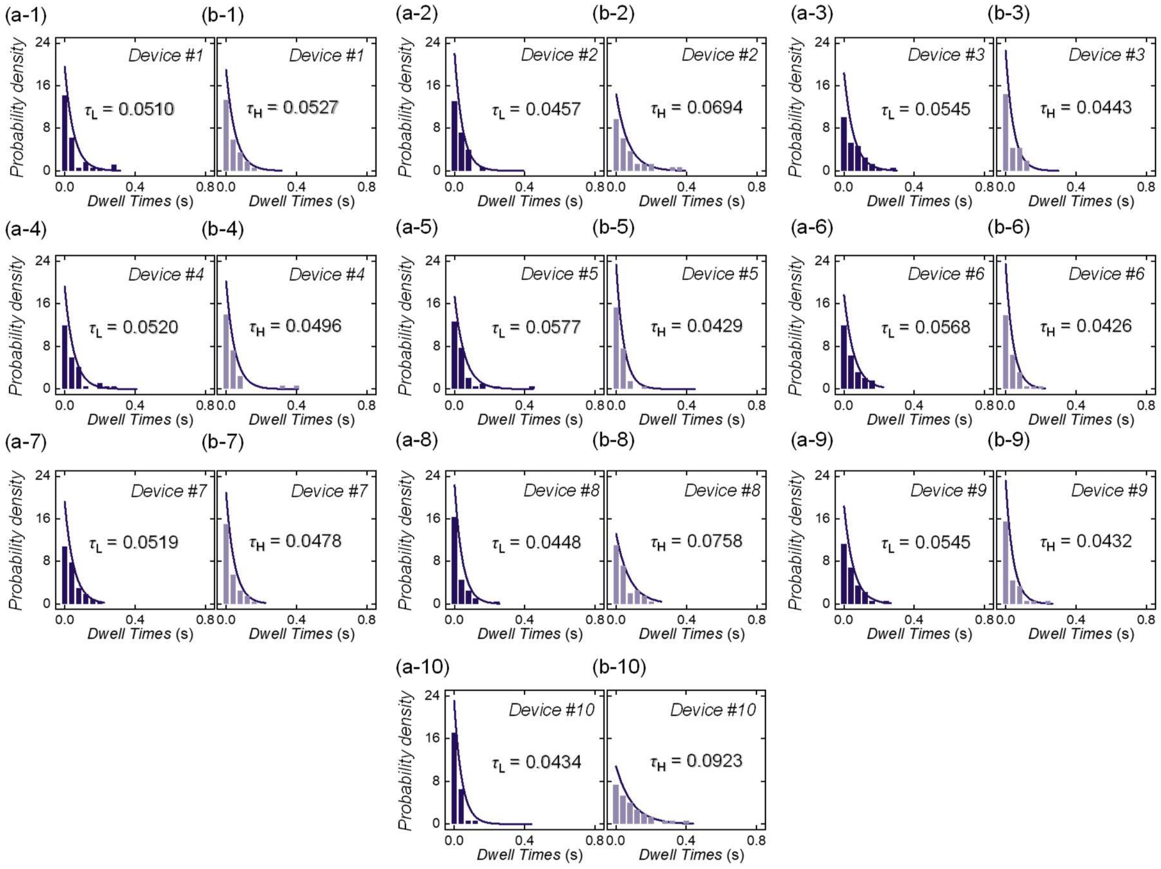

Fig. R2. RTN dwell-time statistics at a single bias (a) Histograms of the low-state dwell time τ_L for 10 devices measured at V = 3.28 V with Poisson (exponential) fits. (b) Histograms of the high-state dwell time τ_H for the same devices and bias with Poisson fits. Across devices the RTN statistics follow Poisson processes and the occupancy of the two levels is balanced at this bias.

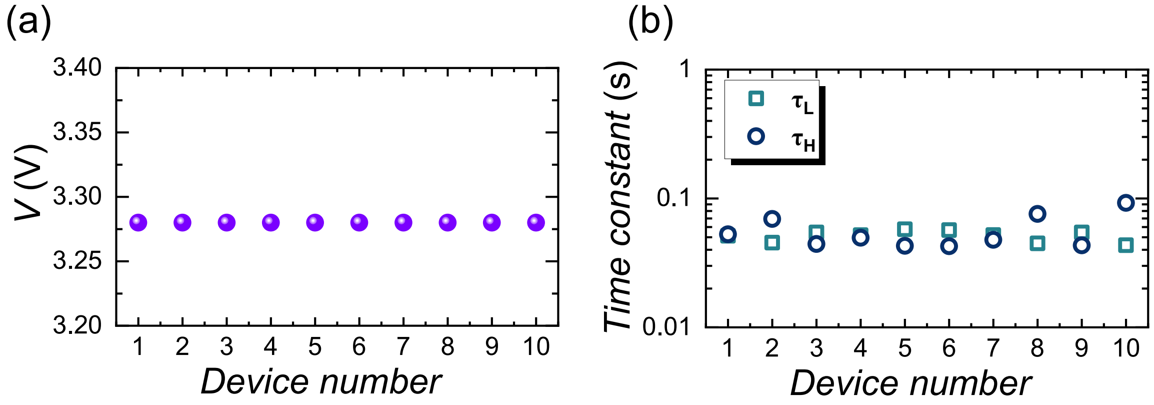

Fig. R3. Device-wise bias and fitted time constants. (a) Applied read voltage for each device used in Fig. R1. (b) Dwell-time constants extracted from Poisson fitting, reported per device for both τ_L and τ_H.

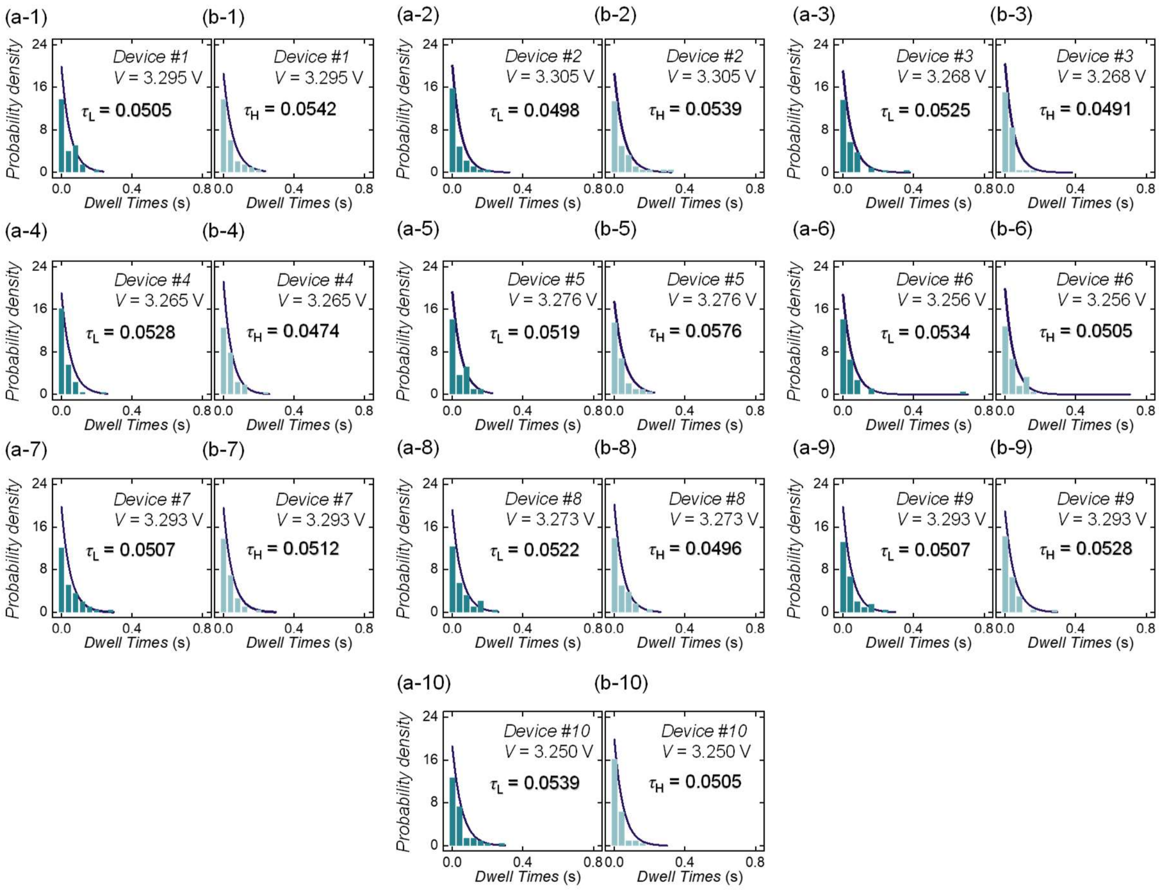

Fig. R4. Bias equalization across devices for balanced occupancy. (a) Histograms of τ_L with Poisson fits for 10 devices, each biased at its own equalized voltage in the range 3.25–3.30 V to balance state occupancy. (b) Corresponding τ_H histograms with Poisson fits. Compared with Fig. R1, per-device bias equalization yields

more balanced occupancy. Inset shows the applied voltage for each device.

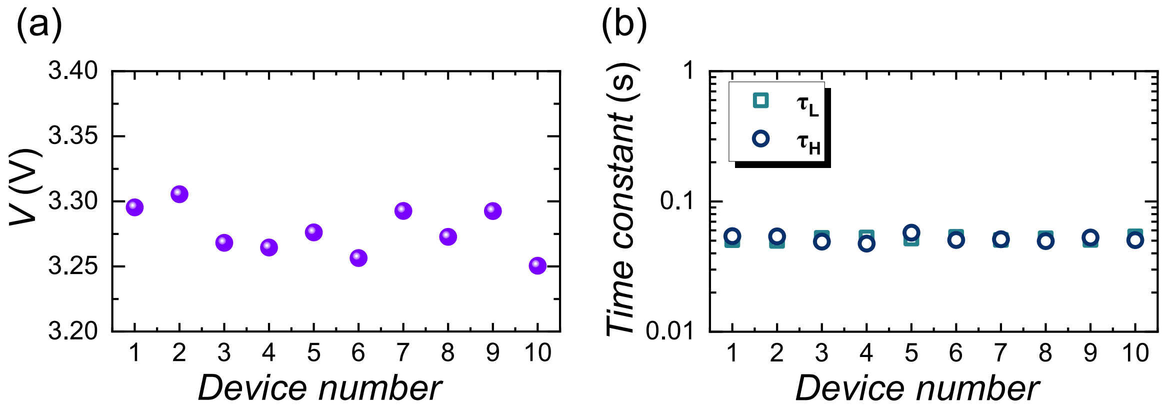

Fig. R5. Summary of equalized bias and fitted constants. (a) Optimized read voltage for each device. (b) Dwell-time constants τ_L and τ_H under the optimized bias for each device, showing markedly tighter clustering than in Fig. R2b.

These results align with the RTN mechanism described in the manuscript, where small field adjustments shift the relative alignment between the trap level and the TiN work function, tuning the transition probabilities toward a 50:50 ratio. In practice, such voltage trimming can be implemented per cell or per column using existing word-line or bit-line DAC architectures, enabling parallel generation of uniform random bitstreams even with minor ALD-induced variability.

Following the reviewer's suggestion, we have added a concise description of the ALD-related variation, the voltage adjustment procedure, and its system-level feasibility in the revised manuscript. Please refer to the revised manuscript to find the modifications.

Corresponding change in manuscript: Yes

Location of Change: Supplementary Figure 12-16

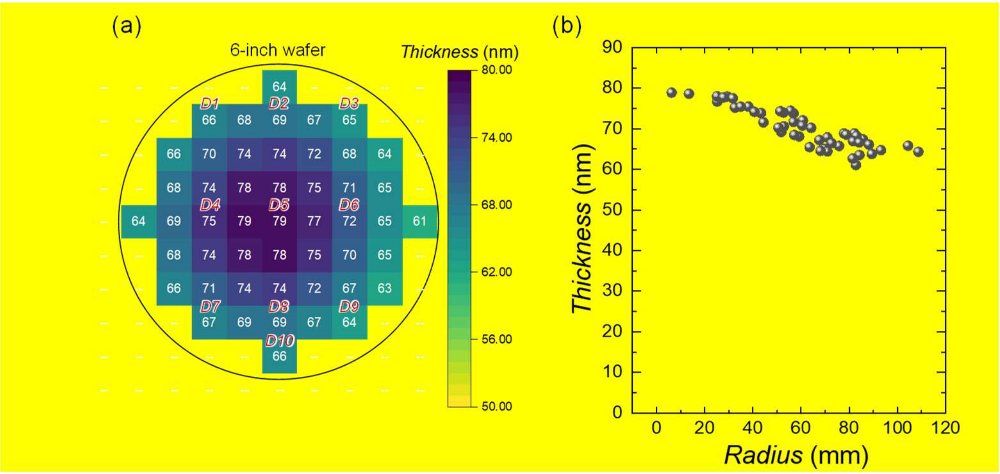

Supplementary Figure 12. Wafer-scale thickness uniformity of ALD HZO. (a) Six-inch wafer map showing ellipsometry-measured HZO thickness at 49 sampled points. The locations of the 10 devices (D1 to D10) used for RTN characterization are also marked on the map. (b) Thickness versus radial distance from the wafer center, showing a slight decreasing trend toward the edge.

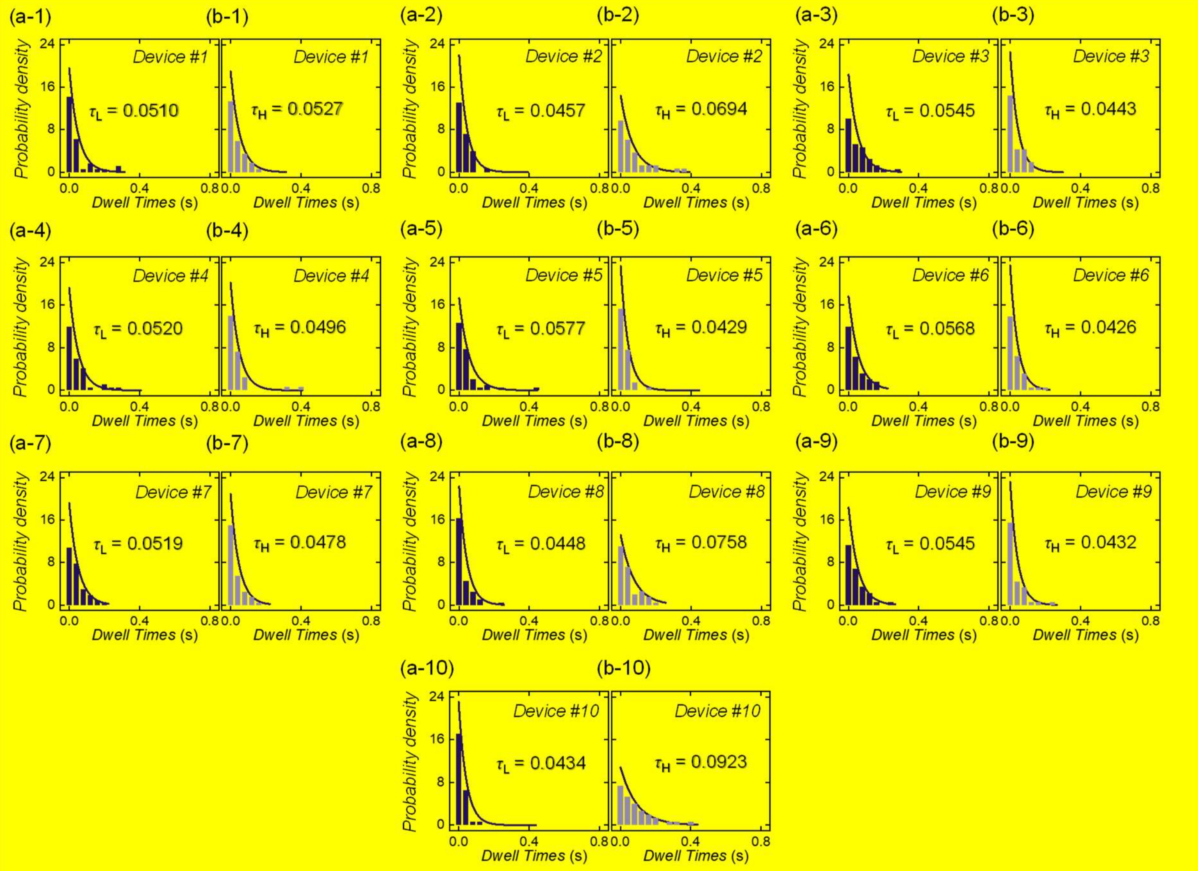

Supplementary Figure 13. RTN dwell-time statistics at a single bias (a) Histograms of the low-state dwell time τ_L for 10 devices measured at V = 3.28 V with Poisson (exponential) fits. (b) Histograms of the high-state dwell time τ_H for the same devices and bias with Poisson fits. Across devices the RTN statistics follow Poisson processes and the occupancy of the two levels is balanced at this bias.

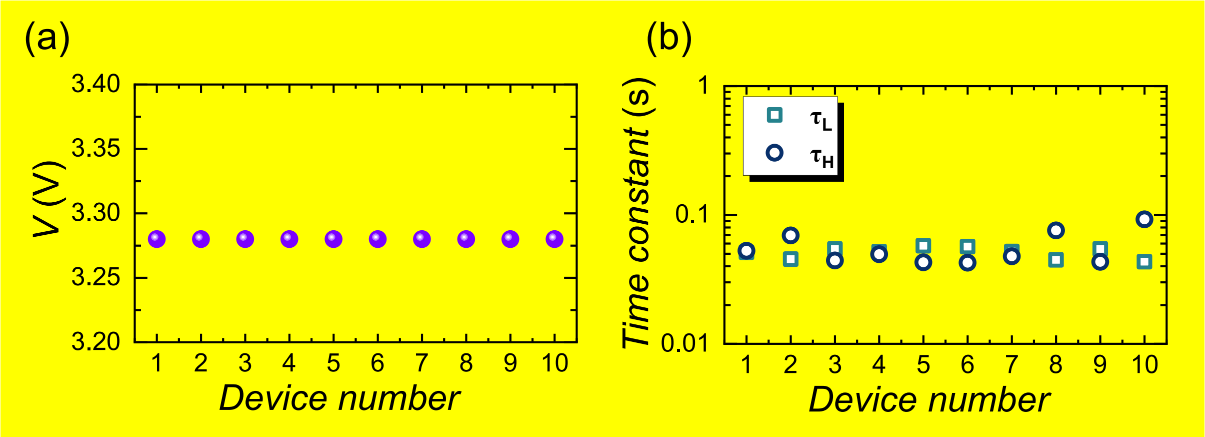

Supplementary Figure 14. Device-wise bias and fitted time constants. (a) Applied read voltage for each device used in Fig. R1. (b) Dwell-time constants extracted from Poisson fitting, reported per device for both τ_L and τ_H.

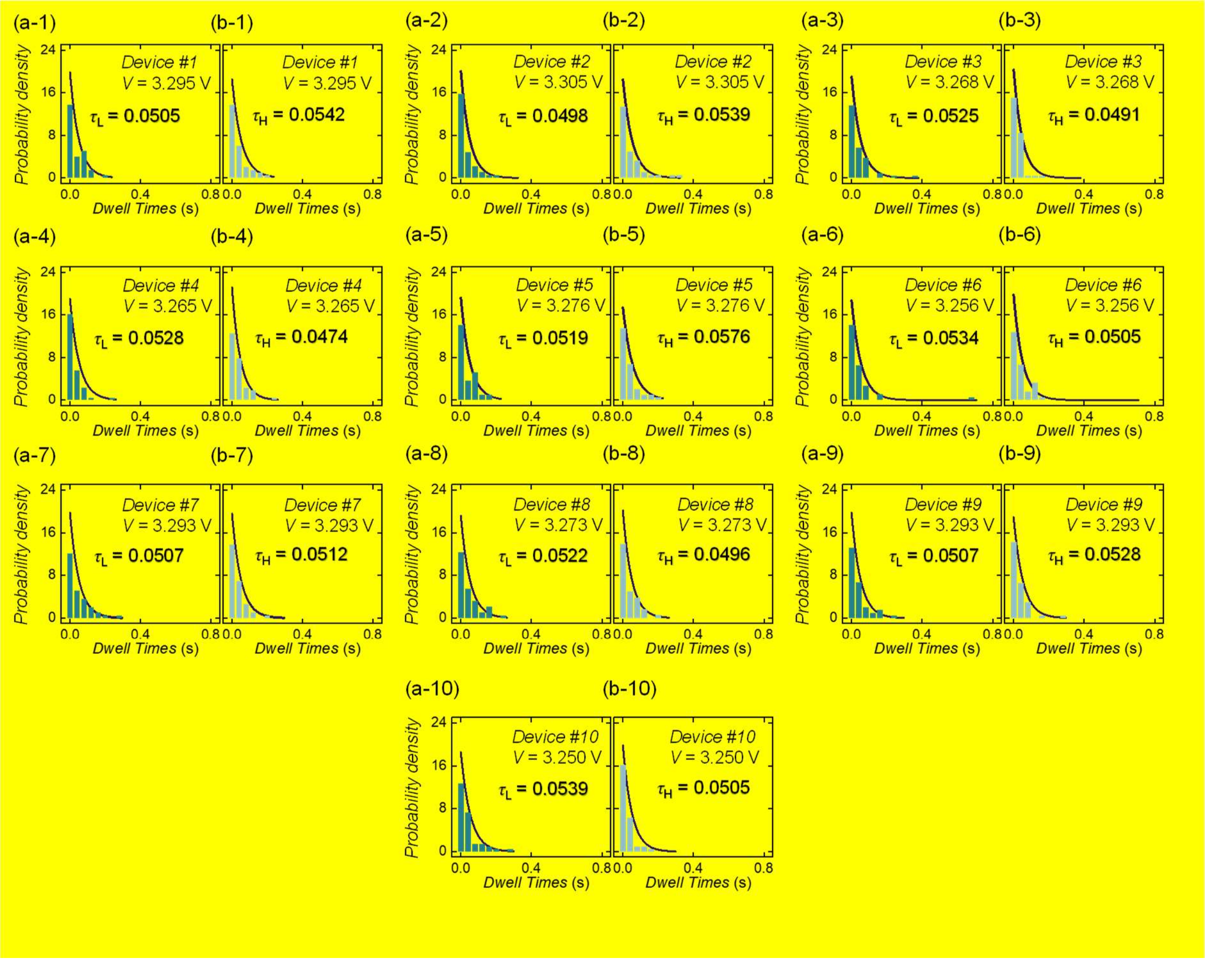

Supplementary Figure 15. Bias equalization across devices for balanced occupancy. (a) Histograms of τ_L with Poisson fits for 10 devices, each biased at its own equalized voltage in the range 3.25–3.30 V to balance state occupancy. (b) Corresponding τ_H histograms with Poisson fits. Compared with Fig. R1, per-device

bias equalization yields more balanced occupancy. Inset shows the applied voltage for each device.

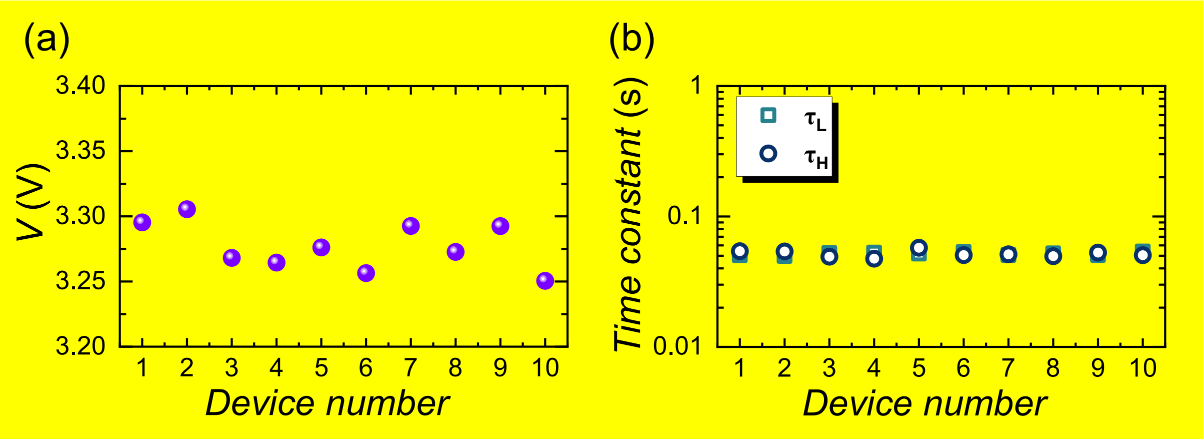

Supplementary Figure 16. Summary of equalized bias and fitted constants. (a) Optimized read voltage for each device. (b) Dwell-time constants τ_L and τ_H under the optimized bias for each device, showing markedly tighter clustering than in Fig. R2b.

Location of Change: Results – (pages 12-13)

To further validate that the RTN characteristics originate from ALD-related traps and remain controllable across the wafer, we measured the HZO thickness and RTN uniformity over a 6-inch wafer (Supplementary Fig. 12). The thickness map and device-wise dwell-time statistics (Supplementary Fig. 13 and Supplementary Fig. 14) confirm uniform Poisson-type RTN behavior with minor device-to-device variation caused by small differences in trap depth and energy level. By iteratively adjusting the read bias in 1 mV steps according to the τL/τH imbalance, the distributions were equalized across all devices (Supplementary Fig. 15 and Supplementary Fig. 16). This simple voltage-trimming method, readily implemented through word- or bit-line DAC control, enables parallel generation of uniform random bitstreams even with slight ALD-induced variability.

2. Please report standard randomness tests (NIST SP 800-22 or TestU01) on the raw bitstreams. State whether the sequences pass i.i.d. and unpredictability metrics.

Response:

Thank you for your comment. As the reviewer pointed out, reporting standard randomness tests and clarifying independent and identically distributed (i.i.d.) behavior are important.

Table R1 presents the NIST SP 800-22 results for a 40 k-bit raw bitstream directly taken from the RTN regime without any post-processing. Once the 0/1 ratio is balanced, several basic tests pass, while a number of mathematically stringent tests remain challenging because the RTN process inherently exhibits short-range correlation between consecutive bits. Fig. R6(a) shows the bit-transition probability *P* between successive bits, confirming a small deviation from the ideal 0.5, which aligns with the residual dependence observed in the failed NIST items. For our target of hardware image generation, unbiased sampling with weak dependence is sufficient for balanced latent coverage and high FID performance, in contrast to cryptographic TRNG applications that require strong unpredictability.

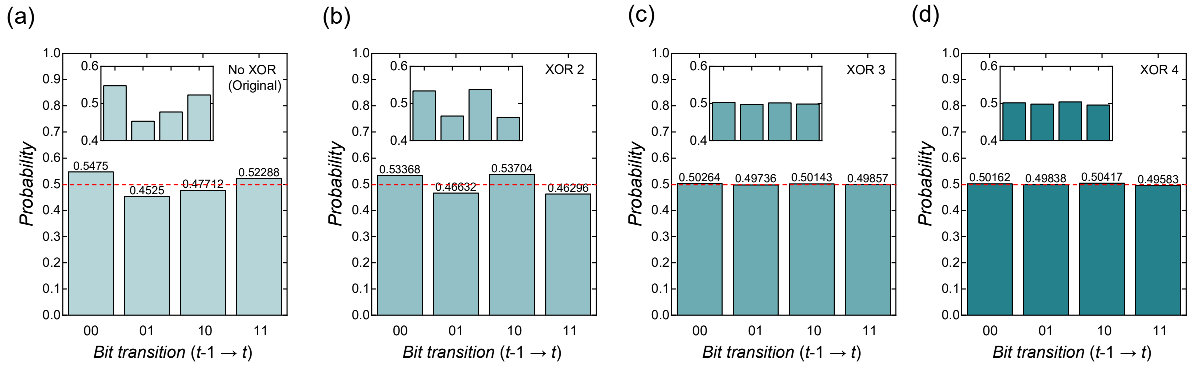

Fig. R6. XOR post-processing improves Markov transition balance. (a) Bit-transition probability *P* for the raw, no-XOR bitstream. (b) Same metric after XOR-2 combining. (c) Same metric after XOR-3 combining. Increasing XOR depth drives the transition probabilities toward the ideal 0.5.

To explore how independence can be improved when required, we applied XOR post-processing, a standard low-overhead technique widely used in hardware random number generators. In our implementation, XOR2 denotes the exclusive OR of every two consecutive raw bits from the same RTN stream, and XOR3 denotes the exclusive OR of every three consecutive bits. Fig. R6(b) and Fig. R6(c) show that increasing the XOR depth progressively moves the transition probability toward 0.5, effectively suppressing short-range memory. Consistently, Table R2 and Table R3 report more NIST tests passing for XOR2 and near-saturated improvement for XOR3. Because XOR operates directly on consecutive bits with negligible area and timing overhead, it provides a simple and practical path to approach i.i.d.-like behavior when stronger independence is desired.

No XOR
(Original)

NIST 800-22	P-value	Result
Frequency	0.0	**Fail**
Block Frequency	0.0	**Fail**
Runs	0.0	**Fail**
Longest Runs	0.7439	**Pass**
Matrix Rank	0.6875	**Pass**
Discrete Fourier Transform (DFT)	0.0379	**Pass**
Non-Overlapping Template	0.9999	**Pass**
Overlapping Template	0.0	**Fail**
Maurer’s Universal Statistical Test	0.0012	**Fail**
Linear Complexity	0.0	**Fail**
Serial	0.0	**Fail**
Approximate Entropy	0.0	**Fail**
Cumulative Sums (Forward)	0.0	**Fail**
Cumulative Sums (Backward)	0.0	**Fail**
Random Excursions	0.0306	**Pass**
Random Excursions Variant	0.3214	**Pass**

Table R1. NIST SP 800-22 results for the original bitstream (no XOR).

XOR 2

NIST 800-22	P-value	Result
Frequency	0.0	**Fail**
Block Frequency	0.0	**Fail**
Runs	0.0	**Fail**
Longest Runs	0.2107	**Pass**
Matrix Rank	0.6440	**Pass**
Discrete Fourier Transform (DFT)	0.1772	**Pass**
Non-Overlapping Template	0.9999	**Pass**
Overlapping Template	0.0	**Fail**
Maurer's Universal Statistical Test	0.0	**Fail**
Linear Complexity	0.0	**Fail**
Serial	0.0	**Fail**
Approximate Entropy	0.0	**Fail**
Cumulative Sums (Forward)	0.0	**Fail**
Cumulative Sums (Backward)	0.0	**Fail**
Random Excursions	0.1188	**Pass**
Random Excursions Variant	0.2650	**Pass**

Table R2. NIST SP 800-22 results for XOR2 post-processed bitstream.

XOR 3

NIST 800-22	P-value	Result
Frequency	0.6579	Pass
Block Frequency	0.7528	Pass
Runs	0.1228	Pass
Longest Runs	0.5260	Pass
Matrix Rank	0.9603	Pass
Discrete Fourier Transform (DFT)	0.1041	Pass
Non-Overlapping Template	0.9999	Pass
Overlapping Template	0.0	Fail
Maurer's Universal Statistical Test	0.8979	Pass
Linear Complexity	0.0	Fail
Serial	0.2163	Pass
Approximate Entropy	0.2177	Pass
Cumulative Sums (Forward)	0.8687	Pass
Cumulative Sums (Backward)	0.8687	Pass
Random Excursions	0.3290	Pass
Random Excursions Variant	0.2412	Pass

Table R3. NIST SP 800-22 results for XOR3 post-processed bitstream.

We have provided the NIST SP 800-22 results for raw and XOR-processed streams in the supplementary note 3 together with Tables R1–R3 and the transition analysis in Fig. R6. Please refer to the revised manuscript to find the modifications.

Corresponding change in manuscript: Yes

Location of Change: Results - (page 16)

We validated randomness using NIST SP 800-22 on raw and XOR-processed RTN bitstreams are provided in the Supplementary Note 3.

Location of Change: Supplementary Figure 22.

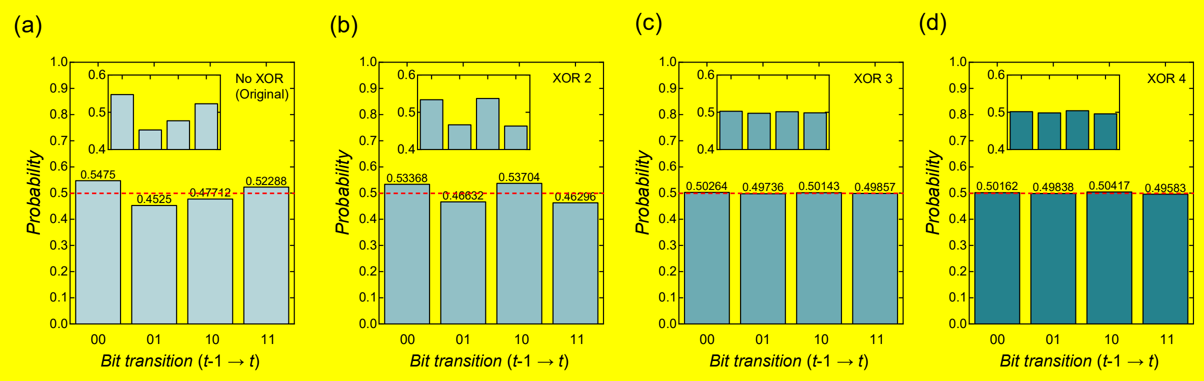

Supplementary Figure 22. XOR post-processing improves Markov transition balance. (a) Bit-transition probability P for the raw, no-XOR bitstream. (b) Same metric after XOR-2 combining. (c) Same metric after XOR-3 combining. Increasing XOR depth drives the transition probabilities toward the ideal 0.5.

Location of Change: Supplemetary Note 3

Supplementary Note 3. Randomness tests and XOR post-processing

We evaluated the statistical quality of the RTN bitstreams using the NIST SP 800-22 test suite. The raw bitstream was obtained directly from the RTN regime without any post-processing. For each condition we collected 40 kbits and applied the standard NIST decision rule with a significance level α equal to 0.01. The representative sampling setting corresponds to the balanced RTN condition used in the main text. This evaluation complements the Markov transition analysis where we quantify the conditional probability of a bit given the previous bit.

Table SN1 reports the NIST SP 800-22 results for the raw bitstream with no post-processing. Once the proportion of zeros and ones is balanced, several tests pass. A few mathematically strict items remain below threshold, which is consistent with the short-range dependence inherent to a two-state RTN process. Fig. S22(a) shows the bit-transition probability between successive bits for this raw case and confirms a small deviation from the ideal value of one half.

To improve independence when required, we applied lightweight XOR post-processing that is widely used in hardware random number generators. We define XOR2 as the exclusive OR of every two consecutive raw bits from the same RTN stream, and XOR3 as the exclusive OR of every three consecutive raw bits from the same stream. Fig. S22(b) and Fig. S22(c) show that increasing the XOR depth progressively drives the transition probability toward one half, indicating suppression of short-range memory. Consistently, Table SN2 and Table SN3 show more NIST items passing for XOR2 and near-saturated improvement for XOR3.

These results should be interpreted in the context of our target application. Our goal is on-chip image generation rather than cryptographic true random number generation. For balanced latent sampling and good image quality, unbiasedness and weak dependence are sufficient. The raw RTN already meets this requirement under the balanced operating point, and XOR post-processing offers a simple and low-overhead path to approach i.i.d.-like behavior when stronger independence is desired. Tables SN1 to SN3 and Fig. SN1 provide the full numerical summary and transition analysis supporting these conclusions.

No XOR
(Original)

NIST 800-22	P-value	Result
Frequency	0.0	Fail
Block Frequency	0.0	Fail
Runs	0.0	Fail
Longest Runs	0.7439	Pass
Matrix Rank	0.6875	Pass
Discrete Fourier Transform (DFT)	0.0379	Pass
Non-Overlapping Template	0.9999	Pass
Overlapping Template	0.0	Fail
Maurer's Universal Statistical Test	0.0012	Fail
Linear Complexity	0.0	Fail
Serial	0.0	Fail
Approximate Entropy	0.0	Fail
Cumulative Sums (Forward)	0.0	Fail
Cumulative Sums (Backward)	0.0	Fail
Random Excursions	0.0306	Pass
Random Excursions Variant	0.3214	Pass

Table SN1. NIST SP 800-22 results for the original bitstream (no XOR).

XOR 2

NIST 800-22	P-value	Result
Frequency	0.0	**Fail**
Block Frequency	0.0	**Fail**
Runs	0.0	**Fail**
Longest Runs	0.2107	**Pass**
Matrix Rank	0.6440	**Pass**
Discrete Fourier Transform (DFT)	0.1772	**Pass**
Non-Overlapping Template	0.9999	**Pass**
Overlapping Template	0.0	**Fail**
Maurer’s Universal Statistical Test	0.0	**Fail**
Linear Complexity	0.0	**Fail**
Serial	0.0	**Fail**
Approximate Entropy	0.0	**Fail**
Cumulative Sums (Forward)	0.0	**Fail**
Cumulative Sums (Backward)	0.0	**Fail**
Random Excursions	0.1188	**Pass**
Random Excursions Variant	0.2650	**Pass**

Table SN2. NIST SP 800-22 results for XOR2 post-processed bitstream.

XOR 3

NIST 800-22	P-value	Result
Frequency	0.6579	Pass
Block Frequency	0.7528	Pass
Runs	0.1228	Pass
Longest Runs	0.5260	Pass
Matrix Rank	0.9603	Pass
Discrete Fourier Transform (DFT)	0.1041	Pass
Non-Overlapping Template	0.9999	Pass
Overlapping Template	0.0	Fail
Maurer's Universal Statistical Test	0.8979	Pass
Linear Complexity	0.0	Fail
Serial	0.2163	Pass
Approximate Entropy	0.2177	Pass
Cumulative Sums (Forward)	0.8687	Pass
Cumulative Sums (Backward)	0.8687	Pass
Random Excursions	0.3290	Pass
Random Excursions Variant	0.2412	Pass

Table SN3. NIST SP 800-22 results for XOR3 post-processed bitstream.

3. Please discuss the impact of noise background on bit-error-rate when reference current Iref drifts owing to temperature or cycling.

Response:

Thank you for your comment. As the reviewer pointed out, it is important to clarify how the background noise affects the bit-error rate (BER) when the reference current I_{Ref} drifts due to temperature or cycling.

Fig. R7(a) schematically illustrates how BER responds as I_{Ref} varies under two noise regimes, depending on the presence of RTN. In the low-noise (1/f-dominant) regime, the read-current distribution forms a single Gaussian peak, and I_{Ref} is necessarily positioned near its center. When I_{Ref} drifts because of temperature or device aging, even a small offset moves the threshold toward one tail of the Gaussian, causing a rapid increase in BER. In contrast, when RTN is present, the read-current distribution consists of two well-separated levels. The decision margin between the high and low states is much wider, and the effect of I_{Ref} variation is strongly suppressed.

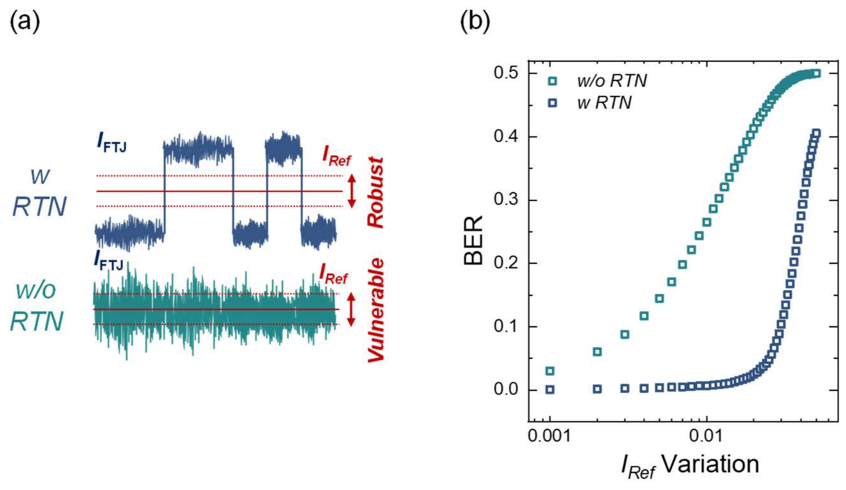

Fig. R7. Robustness to I_{Ref} drift with and without RTN. (a) Schematic explaining why an RTN-driven two-level current provides larger decision margins around I_{Ref} than a low-noise signal, making sensing less sensitive to I_{Ref} drift. (b) Bit-error rate (BER) versus I_{Ref} variation for RTN operation (V = 3.3 V) and non-RTN operation (V = 2.9 V). RTN operation shows lower BER for the same drift magnitude.

Because both temperature and cycling affect I_{Ref} differently depending on the reference-generation circuit, a universal criterion is difficult to define. Therefore, we evaluated the BER as a function of the absolute magnitude of I_{Ref} variation, which provides a common figure of merit. Fig. R7(b) plots the BER versus normalized I_{Ref} deviation for RTN and non-RTN operations. When RTN is absent, even small changes in I_{Ref} cause large BER increases, whereas in the RTN regime, the BER remains extremely low as long as the drift is smaller than the RTN fluctuation amplitude (approximately 5%). In practical systems, this drift can be kept below that level using standard temperature-compensated or self-referenced bias circuits [R1].

Following the reviewer's suggestion, we have added a detailed discussion on how background noise and I_{Ref} drift affect BER, supported by Fig. R7(a) and R7(b), and cited relevant references on reference-current compensation

methods. Please refer to the revised manuscript to find the modifications.

[R1] Yoo, C., & Park, J. (2007). CMOS current reference with supply and temperature compensation. Electronics Letters, 43(25), 1422-1424.

Corresponding change in manuscript: Yes

Location of Change: Results – (pages 10)

Supplementary Fig. 11 (a) shows how noise in I_{Ref} affects the proportion of the 0 and 1 states. Supplementary Fig. 11 (b) shows the bit-error ratio (BER) of '0's to '1's as a function of I_{Ref} variation. In the absence of RTN, most current values cluster around a single mean, causing significant shifts in the 0/1 ratio as I_{Ref} changes. However, in the presence of RTN, the two states are clearly distinguishable, and the 0/1 sequence is generated with uniform robustness against noise and variations.

Location of Change: Supplementary Figure 11

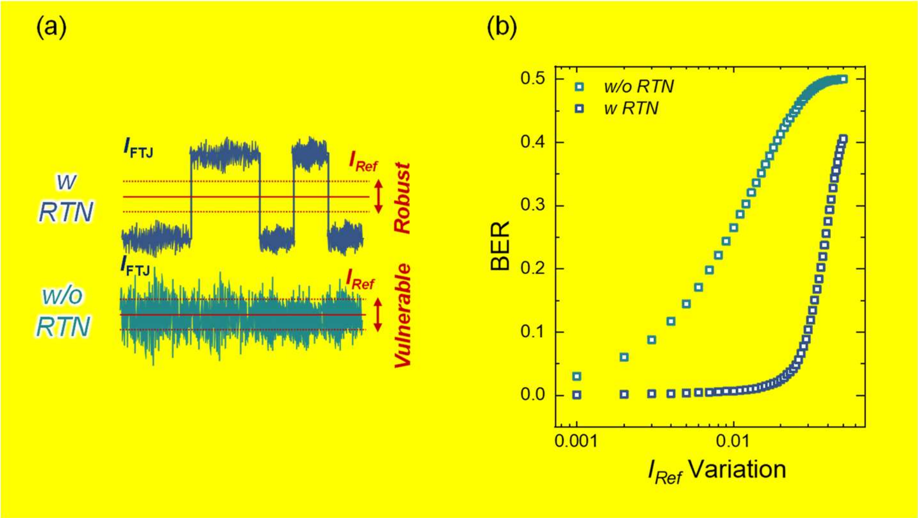

Supplementary Figure 11. Robustness to I_{Ref} drift with and without RTN. (a) Schematic explaining why an RTN-driven two-level current provides larger decision margins around I_{Ref} than a low-noise signal, making sensing less sensitive to I_{Ref} drift. (b) Bit-error rate (BER) versus I_{Ref} variation for RTN operation (V = 3.3 V) and non-RTN operation (V = 2.9 V). RTN operation shows lower BER for the same drift magnitude.

4. The decoder is still emulated on an external GPU. Please provide a complete mapping strategy (weight programming, ADC/DAC resolution, area, and energy) for implementing both encoder and decoder in a 1kb~1Mb FTJ cross-bar array.

Response:

Thank you for your comment. Below we clarify our hardware mapping assumptions. In a standard VAE, the encoder is needed only during training and is not used for image generation at inference. Accordingly, we emulate the encoder in software and provide a hardware mapping assumption for the decoder on FTJ crossbar arrays. Our scheme assumes tiled FTJ macros (Fig. R8) scalable from ~1 kb (32 × 32 array) to 1 Mb (1024 × 1024 array) effective memory capacity. Each real-valued weight is realized by a differential pair of FTJs to encode positive/negative weight values. Inputs are driven on word-lines using pulse-width modulators (PWMs), where pulse duration is proportional to the input value. The resulting bit-line currents are integrated per column, and the integrator outputs are digitized by ADCs to produce the VMM result.

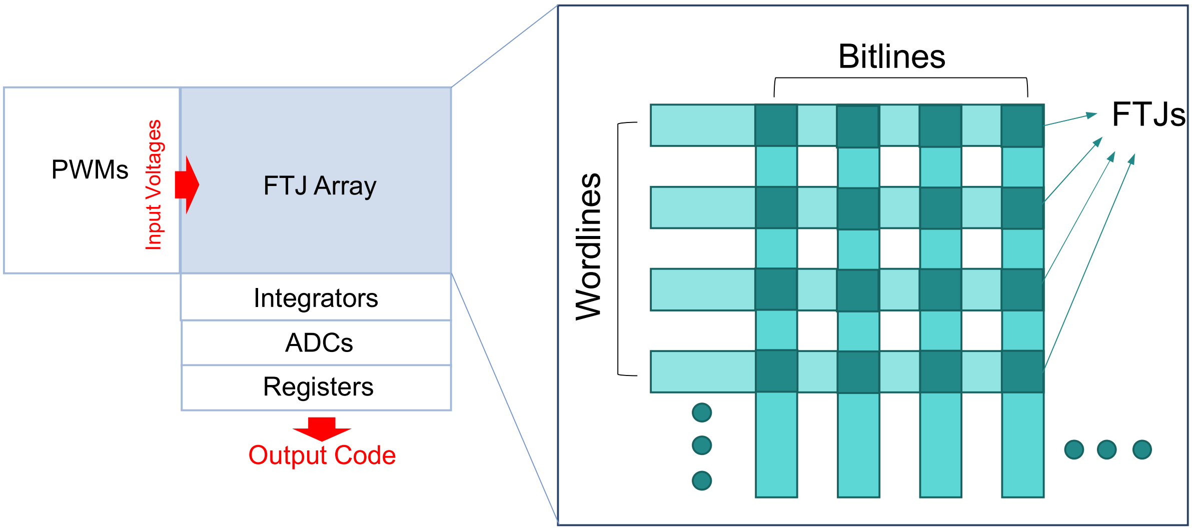

Fig. R8. FTJ cross-bar VMM macro for the VAE decoder. Inputs are applied as voltage pulses on word-lines. bit-line currents are integrated and digitized by ADCs, and the output code is latched in registers.

Weights are programmed as FTJ conductance using method [R1] with verify-and-adjust pulses until the device falls within the target bin of the quantized weight. Referring to the LTP/LTD characteristics, a single FTJ supports 32 stable conductance levels. Using a differential pair yields 64 effective levels per weight parameter. Input precision is set by the PWM (input DAC) timing resolution. For internal decoder layers, we use 5-bit ADCs, which we found sufficient given the 64-level weight quantization. For the final layer, we use an 8-bit ADC to preserve image detail before de-normalizing to pixel space. This split (5-bit internal / 8-bit output) maintained CelebA image quality while containing area and energy.

We have added the detailed mapping strategy of the decoder in Supplementary Note 3. Please refer to the revised manuscript for the corresponding modifications.

[R1] X. Peng, R. Liu, and S. Yu, “Optimizing Weight Mapping and Data Flow for Convolutional Neural Networks on Processing-in-Memory Architectures”, IEEE Transactions on Circuits and Systems-I: Regular Papers, 67, 4, (2020)

Corresponding change in manuscript: Yes

Location of Change: Supplementary Note 2

Supplementary Note 2. Detailed explanation of the mapping strategy of the VAE decoder.

Our scheme assumes tiled FTJ macros (Figure. S19) scalable from ~1 kb (32 × 32 array) to 1 Mb (1024 × 1024 array) effective memory capacity. Each real-valued weight is realized by a differential pair of FTJs to encode positive/negative weight values. Inputs are driven on word-lines using pulse-width modulators (PWMs), where pulse duration is proportional to the input value. The resulting bit-line currents are integrated per column, and the integrator outputs are digitized by ADCs to produce the VMM result.

Weights are programmed as FTJ conductance using method [5] with verify-and-adjust pulses until the device falls within the target bin of the quantized weight. Referring to the LTP/LTD characteristics, a single FTJ supports 32 stable conductance levels. Using a differential pair yields 64 effective levels per weight parameter. Input precision is set by the PWM (input DAC) timing resolution. For internal decoder layers, we use 5-bit ADCs, which we found sufficient given the 64-level weight quantization. For the final layer, we use an 8-bit ADC to preserve image detail before de-normalizing to pixel space. This split (5-bit internal / 8-bit output) maintained CelebA image quality while containing area and energy.

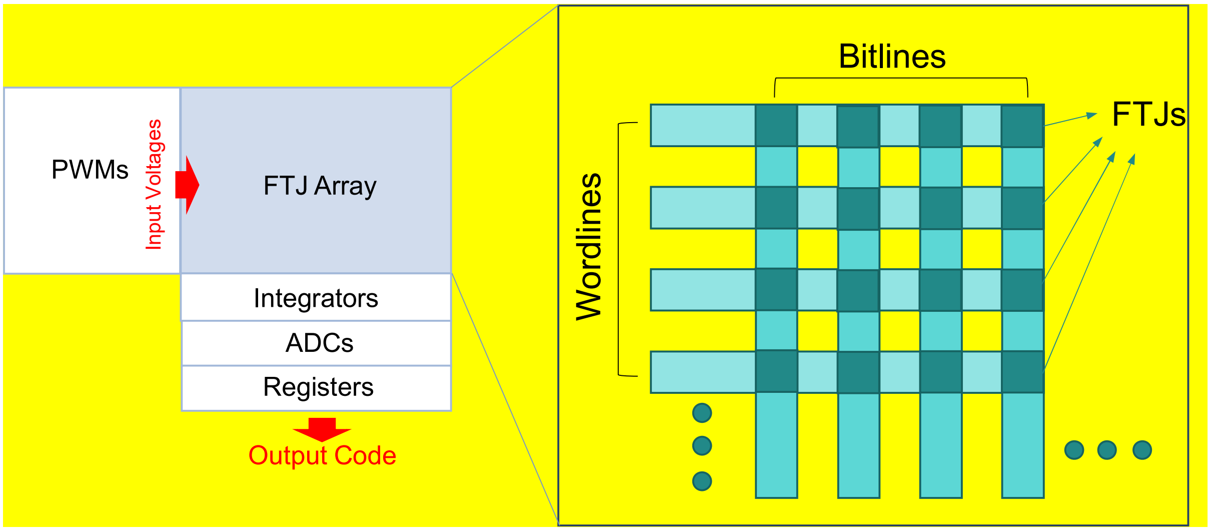

Supplementary Figure 19. FTJ cross-bar VMM macro for the VAE decoder. Inputs are applied as voltage pulses on word-lines. bit-line currents are integrated and digitized by ADCs, and the output code is latched in registers.

[R1] X. Peng, R. Liu, and S. Yu, “Optimizing Weight Mapping and Data Flow for Convolutional Neural Networks on Processing-in-Memory Architectures”, IEEE Transactions on Circuits and Systems-I: Regular Papers, 67, 4,

(2020)

Location of Change: Results – (pages 14)

And Supplementary Note 2 provides a detailed explanation of the mapping strategy of the VAE decoder.

5. Estimate minimum array size required for real-time (30 fps) 128×128 CelebA generation, including: TOPS required for 256×64k VMM,ADC energy (pJ per MAC) and RTN sampling latency overhead.

Response:

Thank you for your comment. We address the requirement from two perspectives, RTN sampling and VMM throughput/energy.

RTN sampling: To sustain 30 fps, a 256-dimensional latent vector must be sampled 30 times per second. Our study uses bitstream sampling with ≥16 streams, so real-time operation requires 256×30×16=122,880 samplings. Assuming a 36 ms sampling window per FTJ, a single device can provide ~27 samples. Thus ~4,552 FTJs are needed solely for RTN sampling. As software optimization is not our focus, this number can be reduced in practice by lowering the latent dimension or the number of bitstreams.

VMM operation and minimum arraying: We assume multiple arrays, since a single 2-D array cannot cover the full VAE decoder at once. In our decoder, one 128 × 128 image generation requires about 3.57 Giga-MAC. At 30 fps this is ~100 GOPS (≈ 0.1 TOPS). With 256 × 256 FTJ arrays and differential weights (two devices per parameter for positive/negative value representation), the minimum array count is 413 (256 for fully connected layers and 157 for the transposed convolutional layers) [R1]. A 256 × 256 array performs 131,072 operations (MACs) per cycle (counting multiply and add separately as 256 × 256 × 2). Even at a modest 10 MHz operation of the macro, it yields ~1.31 TOPS per array. The different layers demand different workloads (especially convolution). For instance, the last layer with 128 × 128 kernel slides, which is the slowest layer, still meets real-time targets, at 10 MHz the per-inference time is ~1.64 ms, so additional replicated arrays for parallelism are not required.

ADC overhead and read energy: The total ADC energy equals the number of ADC activations per inference counts the sampling energy. For our network, the ADC operates 23,396,352 times per inference. Assuming 1 pJ/sample [R2], this is ~23 μJ total, or ~6.44 fJ per MAC. The read-path energy from synaptic currents, in a worst-case estimate (read time 100 ns, LRS current 2.6 μA, read voltage 2 V, and 3.57 × 10^9 operations with all weights and inputs at 1), is ~1.85 mJ per inference (~600 fJ/MAC). In a more realistic setting, shorter read time (5 ns) [R3] and average current ~0.1 × LRS from training statistics, the read energy drops to ~9 μJ per inference (~3 fJ/MAC), which is lower than the ADC contribution.

We have added these details to Supplementary Note 4. Please refer to the revised manuscript for the corresponding modifications.

[R1] X. Peng, R. Liu, and S. Yu, "Optimizing Weight Mapping and Data Flow for Convolutional Neural Networks on Processing-in-Memory Architectures", IEEE Transactions on Circuits and Systems-I: Regular Papers, 67, 4, (2020)

[R2] H. Jiang, W. Li, S. Huang, and S. Yu, "Analog-to-Digital Converter Design Exploration for Compute-in-Memory Accelerators", IEEE Design and Test, 39, 2, 48-55 (2022).

[R3] P. Chen, X. Peng, and S. Yu, “DNN+ NeuroSim: An end-to-end benchmarking framework for compute-in-memory accelerators with versatile device technologies”, 2017 IEEE International Electron Devices Meeting (IEDM), (2019)

Corresponding change in manuscript: Yes

Location of Change: Results – (page 20)

Supplementary Note 7 presents throughput, array requirements, and energy overhead for RTN sampling, VMM operation, and ADCs, evaluated at 30 fps on CelebA.

Location of Change: Supplementary Note 7

Supplementary Note 7. Throughput, array, and energy overhead in terms of RTN Sampling, VMM operation, and ADCs (30 fps, CelebA)

RTN sampling: To sustain 30 fps, a 256-dimensional latent vector must be sampled 30 times per second. Our study uses bitstream sampling with ≥16 streams, so real-time operation requires 256×30×16=122,880 samplings. Assuming a 36 ms sampling window per FTJ, a single device can provide ~27 samples. Thus ~4,552 FTJs are needed solely for RTN sampling. As software optimization is not our focus, this number can be reduced in practice by lowering the latent dimension or the number of bitstreams.

VMM operation and minimum arraying: We assume multiple arrays, since a single 2-D array cannot cover the full VAE decoder at once. In our decoder, one 128 × 128 image generation requires about 3.57 Giga-MAC. At 30 fps this is ~100 GOPS (≈ 0.1 TOPS). With 256 × 256 FTJ arrays and differential weights (two devices per parameter for positive/negative value representation), the minimum array count is 413 (256 for fully connected layers and 157 for the transposed convolutional layers) [5]. A 256 × 256 array performs 131,072 operations (MACs) per cycle (counting multiply and add separately as 256 × 256 × 2). Even at a modest 10 MHz operation of the macro, it yields ~1.31 TOPS per array. The different layers demand different workloads (especially convolution). For instance, the last layer with 128 × 128 kernel slides, which is the slowest layer, still meets real-time targets, at 10 MHz the per-inference time is ~1.64 ms, so additional replicated arrays for parallelism are not required.

ADC overhead and read energy: The total ADC energy equals the number of ADC activations per inference counts the sampling energy. For our network, the ADC operates 23,396,352 times per inference. Assuming 1 pJ/sample [6], this is ~23 µJ total, or ~6.44 fJ per MAC. The read-path energy from synaptic currents, in a worst-case estimate (read time 100 ns, LRS current 2.6 µA, read voltage 2 V, and 3.57 × 10^9 operations with all weights and inputs at 1), is ~1.85 mJ per inference (~600 fJ/MAC). In a more realistic setting, shorter read time (5 ns) [7] and average current ~0.1× LRS from training statistics, the read energy drops to ~9 µJ per inference (~3 fJ/MAC), which is lower than the ADC contribution.

[R1] X. Peng, R. Liu, and S. Yu, “Optimizing Weight Mapping and Data Flow for Convolutional Neural Networks on Processing-in-Memory Architectures”, IEEE Transactions on Circuits and Systems-I: Regular Papers, 67, 4, (2020)

[R2] H. Jiang, W. Li, S. Huang, and S. Yu, “Analog-to-Digital Converter Design Exploration for Compute-in-Memory Accelerators”, IEEE Design and Test, 39, 2, 48-55 (2022).

[R3] P. Chen, X. Peng, and S. Yu, “DNN+ NeuroSim: An end-to-end benchmarking framework for compute-in-memory accelerators with versatile device technologies”, 2017 IEEE International Electron Devices Meeting (IEDM), (2019)

6. Please discuss non-idealities (IR-drop, sneak path, write disturb) and their effect on VMM linearity and FID degradation.

Response:

Thank you for your comment. In our decoder macro, neither word-lines (WLs) nor bit-lines (BLs) are left floating during VMM. Column integrators hold the BLs at virtual ground. Under this biasing, classic crossbar sneak paths are eliminated, as also reported in [R1, R2]. Consequently, the dominant non-ideality is IR-drop.

For IR-drop, we assume an FTJ cell footprint of $4F^2$ (square of side 2F) and analyze a sufficiently large 1024×1024 array. From our process data, the metal sheet resistance is ≈0.02 Ω/□. The worst-case line resistance for the farthest cell is then ~2048 × 0.02 ≈ 40 Ω (WL or BL). With an aggregate read current of ~230 µA during current-sum, the maximum voltage loss is ~10 mV per metal line. Even if we conservatively assume ~10 mV on both WL and BL, the corresponding attenuation of the largest cell's contribution is only ~7%. We model this as an address-dependent gain (weights near the voltage/ground sources experience smaller loss than those farther away) and simulate image generation. In our simulations, the impact of IR-drop translates to negligible FID degradation, as summarized in Fig. R9. FID remains stable at the 1024×1024 scale, and smaller arrays further reduce both parasitic resistance and summed current, making IR-drop even less significant.

We have added a dedicated discussion of IR-drop, sneak paths, and write disturb in the revised manuscript and Supplementary Information, and we updated Fig. R9 accordingly. Please refer to the revised manuscript for these changes.

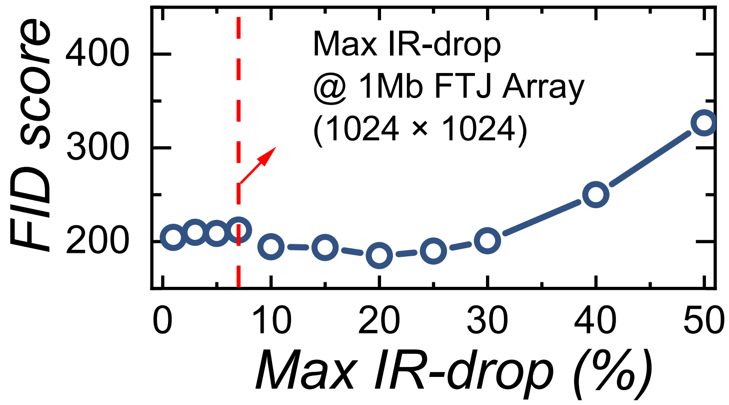

Fig. R9. FID degrades with increasing IR-drop. Red dashed line marks the worst-case IR-drop for a 1 Mb (1024×1024) FTJ array.

[R1] X. Peng, S. Huang, Y. Luo, X. Sun, S. Yu, DNN+NeuroSim: An end-to-end benchmarking framework for compute-in-memory accelerators with versatile device technologies. IEEE International Electron Devices Meeting (IEDM), 771-774 (2019).

[R2] C. Yu, T. Yoo, K. T. C. Chai, T. T. H. Kim, B. Kim, A 65-nm 8T SRAM compute-in-memory macro with column ADCs for processing neural networks. IEEE Journal of Solid-State Circuits 57, 3466–3476 (2022)

Corresponding change in manuscript: Yes

Location of Change: Results – (page 20)

Figure 8(f) presents the FID score as a function of the N_{cycle} applied to the FTJ in the decoder, showing that stable image generation is maintained up to 10^5 read cycles. Effects of RTN operating region and write-cycle count on CelebA generation are shown in Supplementary Fig. 17. Also, the change in FID for CelebA images generated under varying IR-drop is presented in Supplementary Fig. 32. This indicates that the proposed FTJ array can generate high-quality images without being electrically affected by IR-drop.

Location of Change: Supplementary Fig. 32

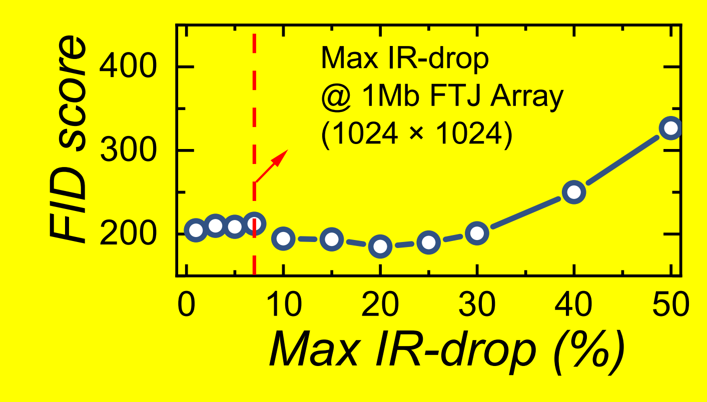

Supplementary Fig. 32. FID degrades with increasing IR-drop. Red dashed line marks the worst-case IR-drop for a 1 Mb (1024×1024) FTJ array.

7. It could be useful to expand Table 1 into a quantitative comparison of energy and area efficiency.

Response:

Thank you for your comment. Using the estimates in our response to **comment 5**, generating one image consumes ~9 µJ in the synaptic path. At 30 fps, this corresponds to 270 µW for synapses. Including ADC energy, the total is ~950 µW. In TOPS/W terms, the energy per MAC is 9.44 fJ (synapse + ADC), which results in 106 TOPS/W. For area, each FTJ is a two-terminal device occupying ~4F^2 (a square of side 2F), as in conventional memristors.

									Latent Sampling		Decoder Mapping
**Work**	**Latent Sampling**	**Decoder Mapping**	**Device type**	**CMOS compatibility**	**Array Size**	**Task**	**Implementation**	**Cell Area**	**Speed***	**Power****	**TOPS/W**
This work	O	O	Both FTJ	O	400	MNIST, CelebA	Circuit-level	2 × 2 µm^2	36 ms	282 µW (FTJ-only)	106 (FTJ & ADC)
[5]	O	-	Bi_2O_2Se	X	1 (Single Device)	MNIST	Device-level	10 × 10 - 40 × 40 µm^2	10 ms	98.1-337 µW	-
[6]	O	-	ReRAM	X	1 (Single Device)	TRNG-only	Circuit-level	5 × 5 - 50 × 50 µm^2	50-250 ms	1.23- 862 µW	-
[7]	O	-	ReRAM	X	1 (Single Device)	TRNG-only	Circuit-level	2.5 × 2.5 µm^2	100 ms	1.47 mW	-
[8]	-	O	ReRAM	O	Only Simulation	MNIST	Simulation-level	-	-	-	4.6
[9]	-	O	1T-1R	O	1000	MNIST	Circuit-level	-	-	-	-
[10]	O	-	CMOS	O	-	TRNG	Circuit-level	-	11.6-312 ns	38.9-91.6 mW	
[11]	-	O	CMOS	O	-	Neural Networks	Commercial	-	-	-	2.3

*Sampling time per bit

**Normalized for 30 fps Human Face Image (128 ×128) Generation

Tera operations per second (TOPS)

Table R4. Comparison of our work with previous reported hardware generative models.

Because several of the works previously cited in Table 1 focus on qualitative discussions rather than comparable hardware metrics, we have added more appropriate references and expanded Table 1 into a quantitative comparison of area and energy efficiency. Please refer to the revised manuscript for these updates.

Corresponding change in manuscript: Yes

Location of Change: Table 1

									Latent Sampling		Decoder Mapping
Work	Latent Sampling	Decoder Mapping	Device type	CMOS compatibility	Array Size	Task	Implementation	Cell Area	Speed*	Power**	TOPS/W
This work	O	O	Both FTJ	O	400	MNIST, CelebA	Circuit-level	2 × 2 μm^2	36 ms	282 μW (FTJ-only)	106 (FTJ & ADC)
[5]	O	-	Bi_2O_2Se	X	1 (Single Device)	MNIST	Device-level	10 × 10 - 40 × 40 μm^2	10 ms	98.1-337 μW	-
[6]	O	-	ReRAM	X	1 (Single Device)	TRNG-only	Circuit-level	5 × 5 - 50 × 50 μm^2	50-250 ms	1.23- 862 μW	-
[7]	O	-	ReRAM	X	1 (Single Device)	TRNG-only	Circuit-level	2.5 × 2.5 μm^2	100 ms	1.47 mW	-
[8]	-	O	ReRAM	O	Only Simulation	MNIST	Simulation-level	-	-	-	4.6
[9]	-	O	1T-1R	O	1000	MNIST	Circuit-level	-	-	-	-
[10]	O	-	CMOS	O	-	TRNG	Circuit-level	-	11.6-312 ns	38.9-91.6 mW	
[11]	-	O	CMOS	O	-	Neural Networks	Commercial	-	-	-	2.3

*Sampling time per bit

**Normalized for 30 fps Human Face Image (128 ×128) Generation

Tera operations per second (TOPS)

Table 1. Comparison of our work with previous reported hardware generative models.

Location of Change: Results – (page 2)

As a result, prior efforts have largely depended on hybrid architectures or off-chip software-based stochastic modules, constraining system scalability, energy efficiency, and integration density [5-11]. Moreover, in mature CMOS-based technologies, separate studies have been proposed to perform each role, implementing stochastic functionality [8] and handling deterministic models [11,12].

Location of Change: Results – (page 21)

FTJ-based platform is also quantitatively benchmarked against the mature CMOS-based designs in area, power per sample, and latency (Table 1). For VMM specifically, our FTJ arrays achieve <10 fJ/MAC, over 40× lower than the ~430 fJ/MAC reported for 8-bit MACs on a widely deployed TPU [11], and sustain ~1.31 TOPS per 256×256 array at 10 MHz, with throughput scaling through array replication.

8. Fig. 7h shows FID saturation at 10^5 cycles. Please verify whether this is limited by FTJ on/off ratio degradation, or external GPU numerical precision.

Response:

Thank you for your comment. As the reviewer pointed out, directly linking cycling-induced drift in conductance distributions to the observed FID increase is important.

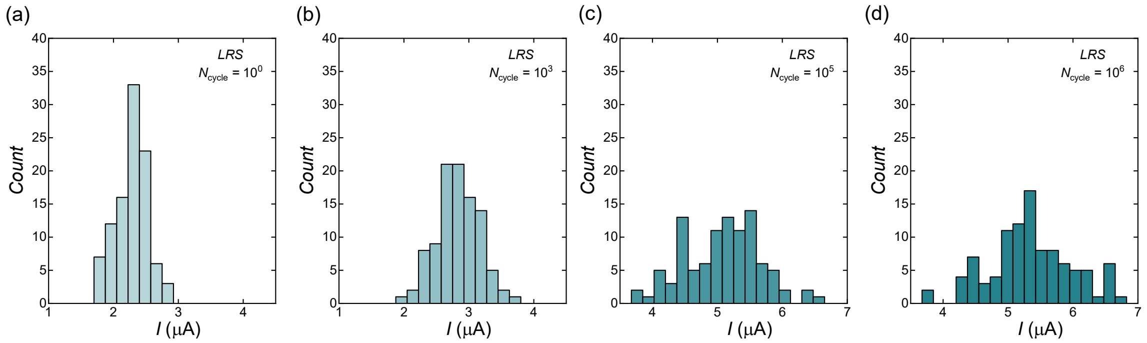

Fig. R10. Cycle-dependent current distribution in LRS. Histograms of cell current in LRS at (a) $N_{\text{cycle}} = 10^0$, (b) $N_{\text{cycle}} = 10^3$, (c) $N_{\text{cycle}} = 10^5$, and (d) $N_{\text{cycle}} = 10^6$.

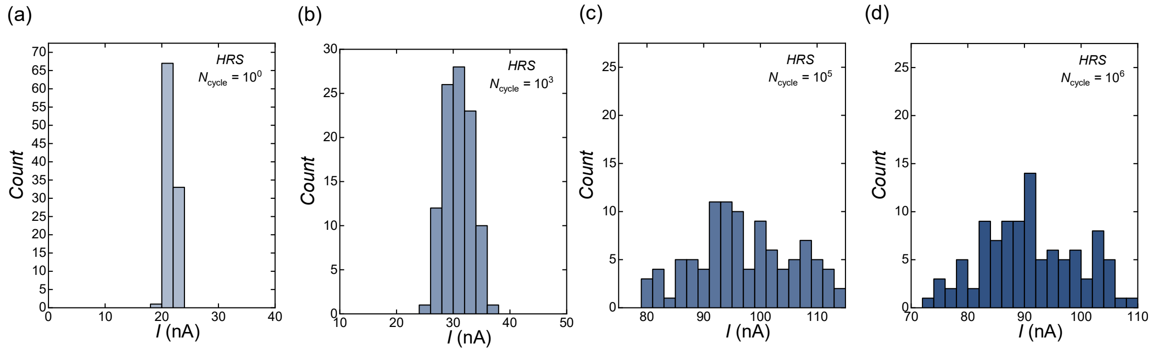

Fig. R11. Cycle-dependent current distribution in HRS. Histograms of cell current in LRS at (a) $N_{\text{cycle}} = 10^0$, (b) $N_{\text{cycle}} = 10^3$, (c) $N_{\text{cycle}} = 10^5$, and (d) $N_{\text{cycle}} = 10^6$.

Fig. R10 shows LRS current histograms at $N_{\text{cycle}} = 10^0$, 10^3, 10^5, and 10^6. The peak shifts modestly and broadens with cycling, indicating increased variability. Fig. R11 shows the corresponding HRS histograms at the same cycle counts**.** The HRS peak broadens and drifts more strongly than LRS, increasing LRS–HRS overlap. Consistent with this observation, Fig. R12 quantifies the spread as σ_I/I_{avg} versus N_{cycle} and shows a steeper increase for HRS than for LRS, confirming that HRS variability becomes the dominant contributor to overlap at high cycle counts. This quantitative trend in Fig. R12 directly supports the histogram-level drift in Fig. R10–R11 and explains why the effective on/off ratio degrades with cycling.

This progressive overlap reduces the effective on/off ratio and increases read errors during VMM accumulation,

which explains the FID increase observed at high cycle counts. Therefore, suppressing drift and the cycling-induced increase of variation in the HRS will be an important focus of future work, and we thank the reviewer for bringing up this point. Following the reviewer's suggestion, we have placed these histograms directly below the original FID plot in the revised manuscript so that readers can see the one-to-one correspondence between distribution drift and FID degradation. Please refer to the revised manuscript to find the modifications.

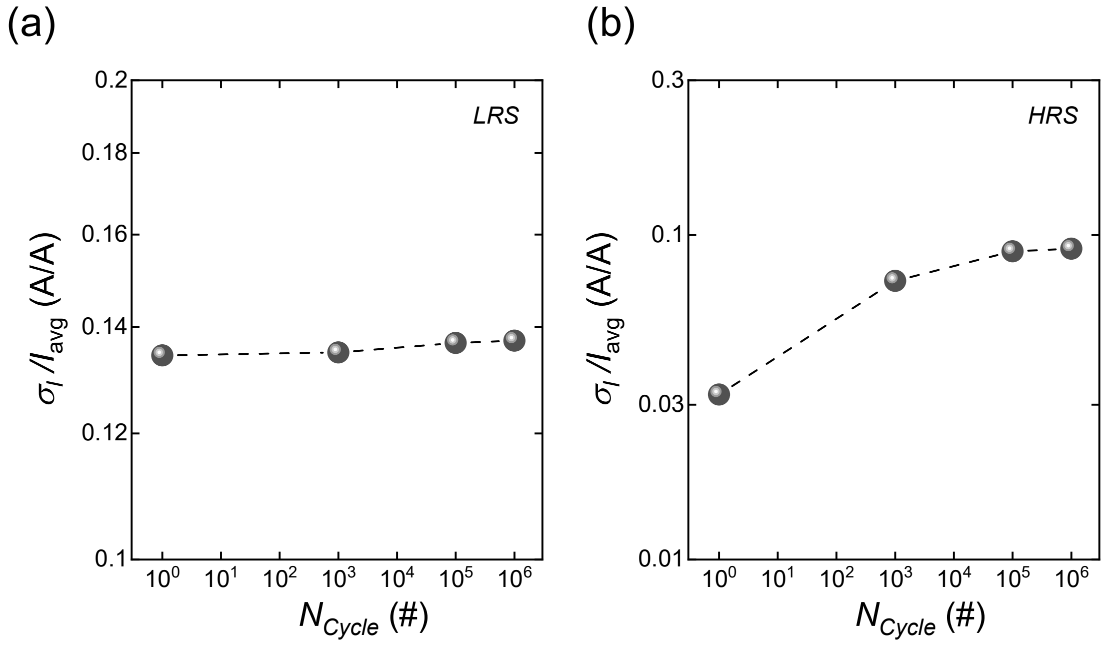

Fig. R12. Spread of current over cycling. (a) σ_I/I_{avg} versus N_{cycle} in LRS. (b) σ_I/I_{avg} versus N_{cycle} in HRS.

Corresponding change in manuscript: Yes

Location of Change: Fig. 6(i)

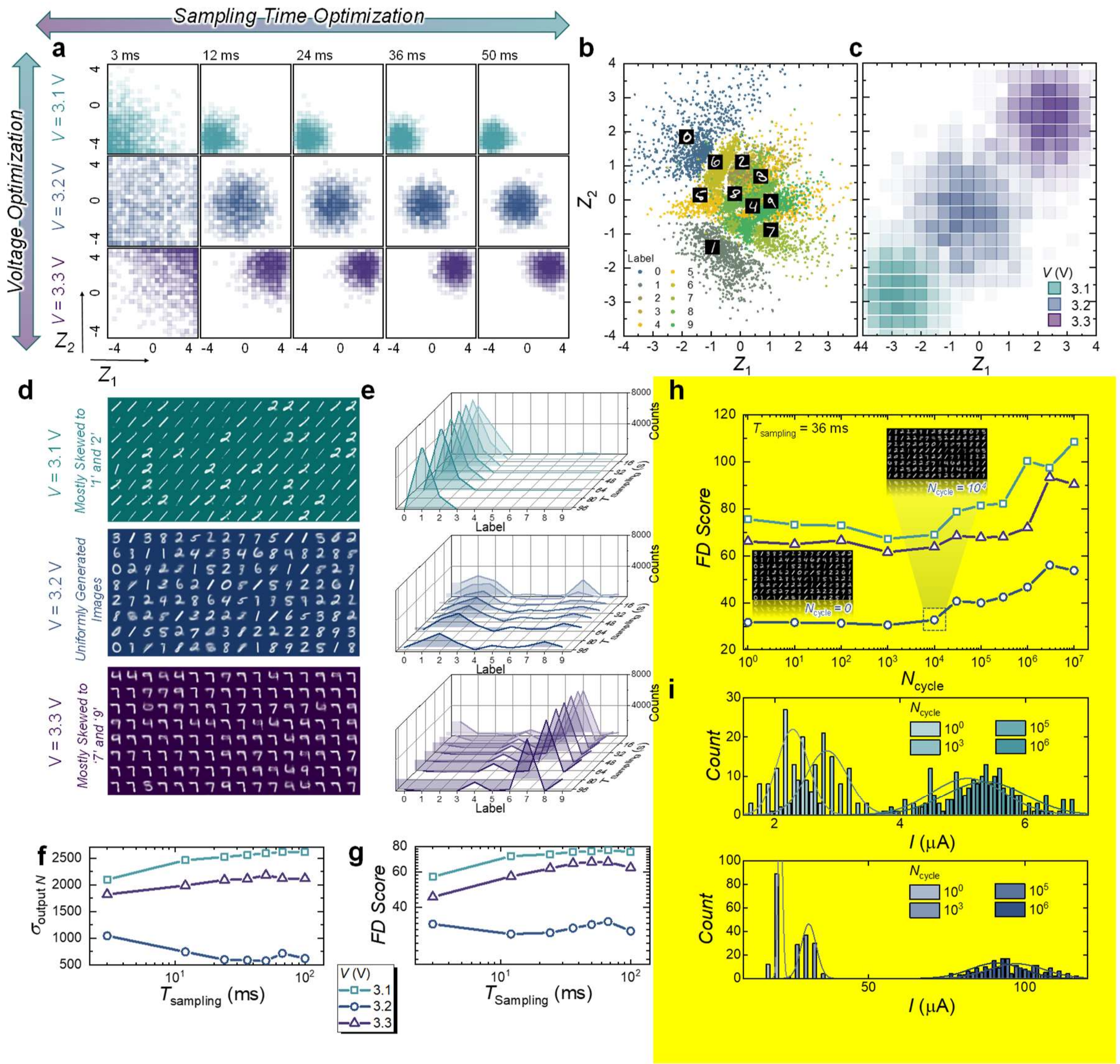

Figure 6. Evaluation of latent space distribution and image generation performance under varying voltage and sampling conditions. a Distribution of generated latent vectors for each V and T_{Sampling}, with biased distributions observed at 3.1 V and 3.3 V. **b** Latent space for the actual MNIST dataset, showing the ideal distribution for accurate image generation. **c** Distribution of random variables in the latent space generated by varying V at fixed T_{Sampling} = 36 ms. **d** Generated MNIST images at different voltages. At V = 3.1 V, only digits "1" and "2" are generated, while V =3.2 V produces all digits evenly, and V =3.3 V is biased toward digits "7" and "9." **e** Distribution of generated images by label across different voltages and sampling conditions, with V = 3.2 V showing the most uniform distribution. **f** $\sigma_{\text{output N}}$ of generated images by label, with V = 3.2 V and T_{Sampling} = 36 ms showing the lowest deviation. **g** FD score of generated images, with V = 3.2 V and T_{Sampling} = 36 ms presenting the lowest FD score, indicating the highest image diversity. **h** FD score versus N_{cycle}s, confirming robust FTJ operation in the absence of RTN. **i** Cycle-dependent current distribution in HRS (up) and LRS (down). Histograms of cell current in LRS at (a) $N_{\text{cycle}} = 10^0$, (b) $N_{\text{cycle}} = 10^3$, (c) $N_{\text{cycle}} = 10^5$, and (d) $N_{\text{cycle}} = 10^6$.

Location of Change: Results – (page 19)

Figure 6(h) presents the FD score as a function of N_{cycle}, demonstrating that stable image generation is maintained even up to 10^5 generation cycles. Figure 6(i) further shows the cycle-dependent current distributions in HRS (top) and LRS (bottom): histograms at (a) $N_{cycle}=10^0$, (b) 10^3, (c) 10^5, and (d) 10^6. The HRS peak broadens and drifts more strongly than LRS, increasing their overlap and effectively reducing the on/off ratio, which correlates with the slight FD increase at high cycle counts. Effects of RTN operating region and write-cycle count on MNIST generation are shown in Supplementary Fig. 26. This result confirms the robustness and reliability of the system under repeated operation.

9. Please provide conductance distribution histograms (LRS/HRS and intermediate states) after 10^3, 10^5, and 10^6 cycles to directly link device drift to FID increase.

Response:

Thank you for your comment. As the reviewer pointed out, providing conductance distribution histograms after cycling is important to directly connect device drift with the observed FID increase. Fig. R10 shows LRS current histograms at $N_{\text{cycle}} = 10^0$, 10^3, 10^5, and 10^6, and Fig. R11 shows the corresponding HRS histograms; representative intermediate-state histograms under the same conditions are also included. Based on our response to **comment 8**, and to make this linkage clearer to readers, we placed these cycle-resolved histograms immediately below Fig. 7h so the correspondence between distribution drift and FID degradation is visible at a glance. Following the reviewer's suggestion, we have incorporated Figures R11 and R12 as the new Fig. 7(i) in the manuscript, along with the accompanying explanation in the revised text. Please refer to the revised manuscript for these updates.

Corresponding change in manuscript: Yes

Location of Change: Fig. 6(i)

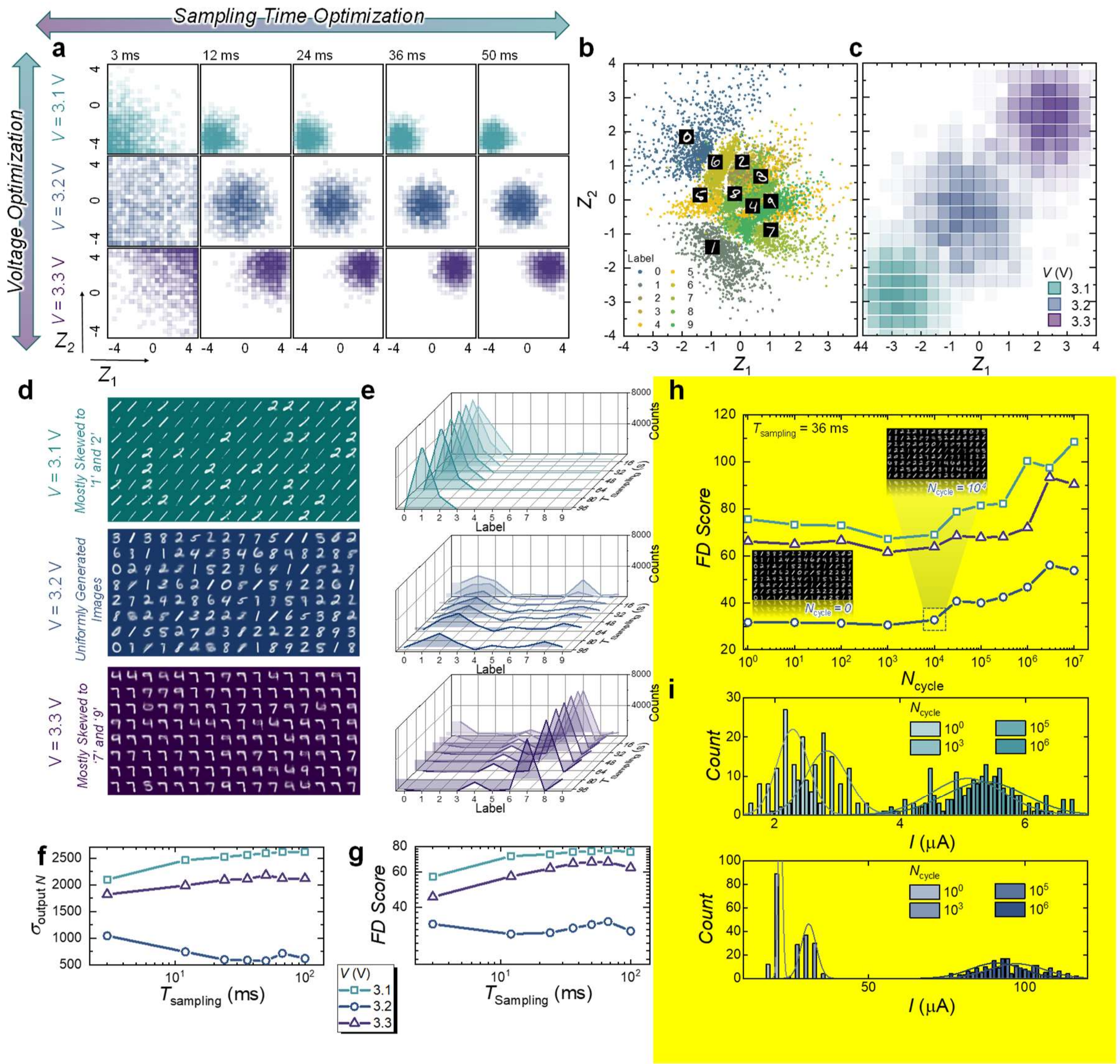

Figure 6. Evaluation of latent space distribution and image generation performance under varying voltage and sampling conditions. a Distribution of generated latent vectors for each V and T_{Sampling}, with biased distributions observed at 3.1 V and 3.3 V. **b** Latent space for the actual MNIST dataset, showing the ideal distribution for accurate image generation. **c** Distribution of random variables in the latent space generated by varying V at fixed T_{Sampling} = 36 ms. **d** Generated MNIST images at different voltages. At V = 3.1 V, only digits "1" and "2" are generated, while V =3.2 V produces all digits evenly, and V =3.3 V is biased toward digits "7" and "9." **e** Distribution of generated images by label across different voltages and sampling conditions, with V = 3.2 V showing the most uniform distribution. **f** $\sigma_{\text{output N}}$ of generated images by label, with V = 3.2 V and T_{Sampling} = 36 ms showing the lowest deviation. **g** FD score of generated images, with V = 3.2 V and T_{Sampling} = 36 ms presenting the lowest FD score, indicating the highest image diversity. **h** FD score versus N_{cycle}s, confirming robust FTJ operation in the absence of RTN. **i** Cycle-dependent current distribution in HRS (up) and LRS (down). Histograms of cell current in LRS at (a) $N_{\text{cycle}} = 10^0$, (b) $N_{\text{cycle}} = 10^3$, (c) $N_{\text{cycle}} = 10^5$, and (d) $N_{\text{cycle}} = 10^6$.

Location of Change: Results – (page 19)

Figure 6(h) presents the FD score as a function of N_{cycle}, demonstrating that stable image generation is maintained even up to 10^5 generation cycles. Figure 6(i) further shows the cycle-dependent current distributions in HRS (top) and LRS (bottom): histograms at (a) $N_{cycle}=10^0$, (b) 10^3, (c) 10^5, and (d) 10^6. The HRS peak broadens and drifts more strongly than LRS, increasing their overlap and effectively reducing the on/off ratio, which correlates with the slight FD increase at high cycle counts. Effects of RTN operating region and write-cycle count on MNIST generation are shown in Supplementary Fig. 26. This result confirms the robustness and reliability of the system under repeated operation.

10. It could be useful to superimpose the t-SNE or UMAP embedding plots of 10,000 generated images on top of the real data in Figures 7d and 8d, in order to visually quantify the degree of distribution overlap.

Response:

Thank you for your valuable suggestion. To directly assess distribution overlap, we now present Fig. R13 and Fig. R14 with a consistent embedding and coloring pipeline. In Fig. R13, we show the t-SNE layout of 10,000 ideally software-generated CelebA images as a reference manifold: each image is first embedded by a ResNet-50 encoder pretrained on ImageNet (classifier head removed) and L_2-normalized, then reduced to 128D via PCA fit once on the normalized real-data reference subset; we apply the same linear projection to all images to ensure comparability. The 2D layout is obtained with t-SNE (target perplexity 30, capped at (n_{fit}−1)/3) using PC-based initialization, early exaggeration, and an automatic learning-rate schedule; for scalability, we fit *t*-SNE on a uniform random subset with a fixed seed and place remaining points by out-of-sample interpolation (inverse-distance-weighted mapping from *k* nearest neighbors in PCA space). Points are colored by *k*-means clusters defined in PCA space on the reference, and this cluster palette is reused throughout for one-to-one comparison.

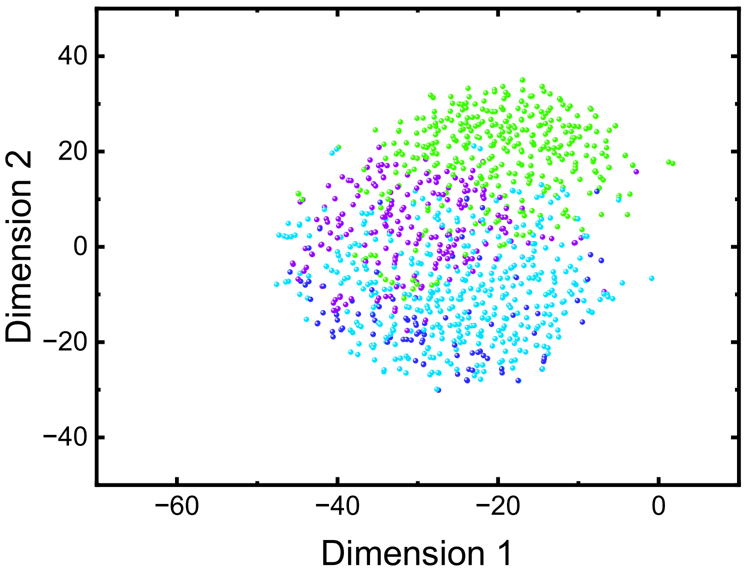

Fig. R13. *t*-SNE embeddings of ramdomly chosen 10k original CelebA images per condition. Points are colored by *k*-means clusters defined in PCA space.

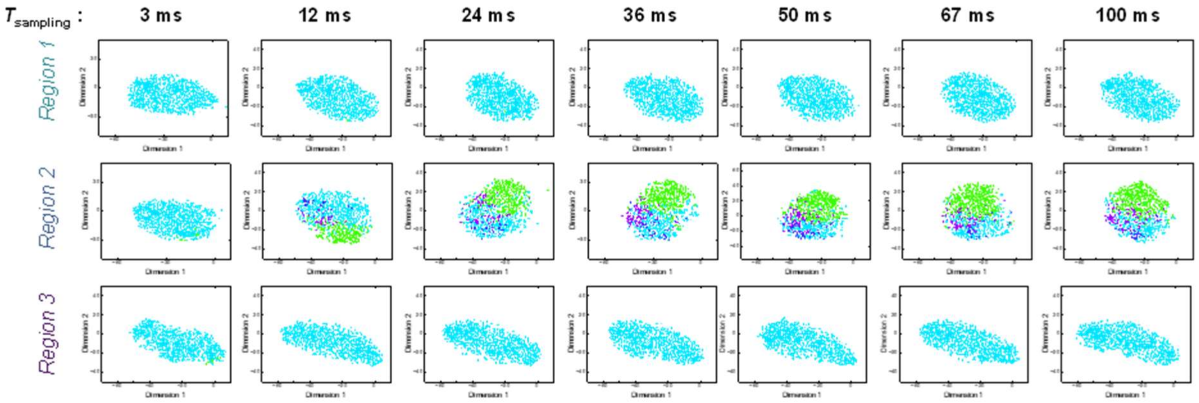

Fig. 14. *t*-SNE embeddings of 10k generated CelebA images per condition. Rows show Region 1 to Region 3 and columns sweep $T_{sampling}$ from 3 ms to 100 ms. Points are colored by *k*-means clusters defined in PCA space. Layouts are aligned to reveal how distribution coverage and cluster structure vary with region and $T_{sampling}$.

Using the identical feature and embedding settings, Fig. R14 visualizes the *t*-SNE layouts of 10,000 hardware-generated CelebA images across conditions, with rows denoting Region 1-3 and columns sweeping $T_{sampling}$ from 3 ms to 100 ms. The layouts are aligned to Fig. R13 and rendered with the same cluster assignments, making shifts in coverage and internal cluster geometry directly visible across regions and sampling times. We observe that, as the generation parameters approach the identified optimal condition, the cluster structure and spatial support in Fig. R14 converge toward those in Fig. R13, indicating improved alignment with the target data manifold. Following the reviewer's suggestion, we have added Figures R13 and R14 to the Supporting Information, together with the accompanying explanation in the revised text. Please refer to the revised manuscript for these updates.

Corresponding change in manuscript: Yes

Location of Change: Results – (page 20)

Supplementary Fig. 29 and Supplementary Fig. 30 and Supplementary Note 6 show t-SNE visualizations of ideally software-generated images and CelebA images generated under hardware constraints, respectively. As the generation settings approach the identified optimal point, the t-SNE plot resembles that of the ideal software-generated images. This convergence in the embedding space substantiates our claim that the proposed optimal condition best aligns the generator with the target data manifold.

Location of Change: Fig. R13, Fig. R14

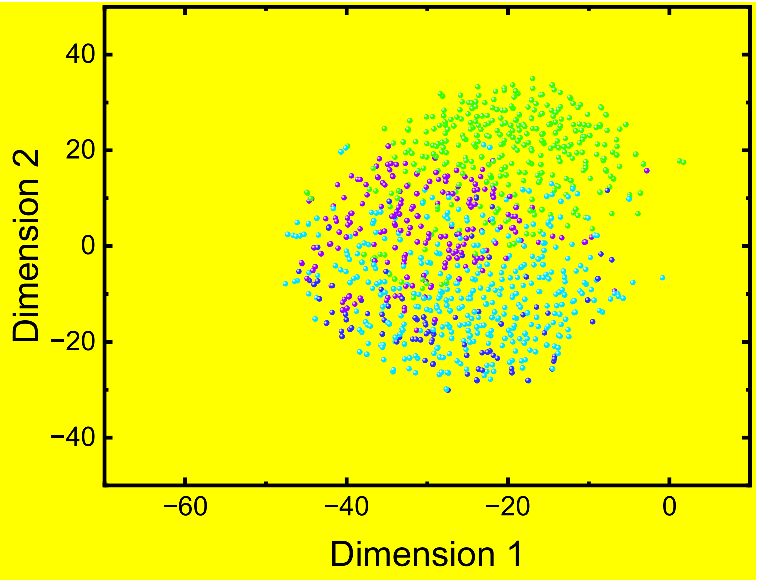

Supplementary Figure 29. ***t*****-SNE embeddings of ramdomly chosen 10k original CelebA images per condition. Points are colored by *k*-means clusters defined in PCA space.**

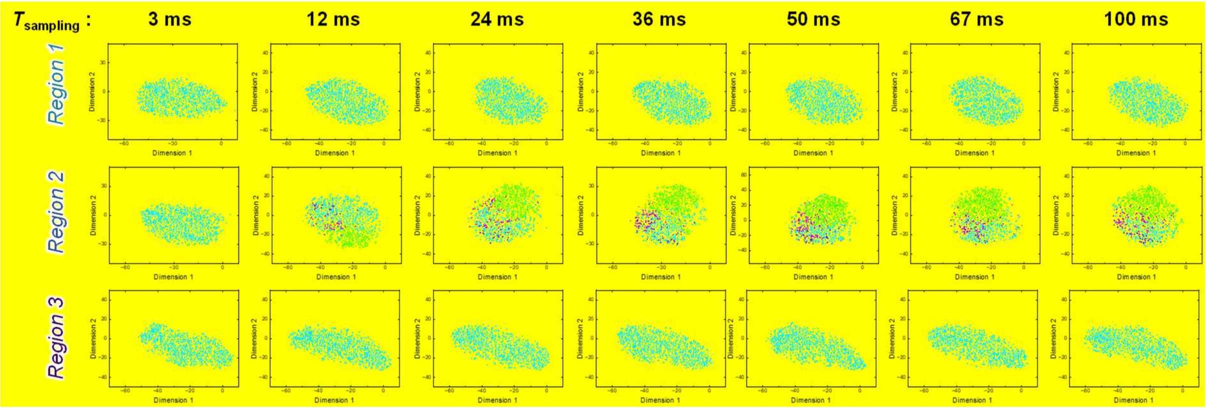

Supplementary Figure 30. ***t*****-SNE embeddings of 10k generated CelebA images per condition.** Rows show Region 1 to Region 3 and columns sweep T_{sampling} from 3 ms to 100 ms. Points are colored by *k*-means clusters defined in PCA space. Layouts are aligned to reveal how distribution coverage and cluster structure vary with region and T_{sampling}.

Location of Change: Supplementary Note 6

Supplementary Note 6. Methodology for *t*-SNE Embedding of CelebA Images

This note describes the procedure used to embed a large set of the generated CelebA images into two dimensions for visualization and analysis. The method is designed to preserve local neighborhood structure in a fixed feature space while remaining scalable and reproducible.

Each image is first mapped to a high dimensional feature vector by a pretrained encoder (ResNet-50 pretrained with ImageNet, which is supported in torchvision module). The classifier head is removed. Every feature vector is normalized to unit length using the L_2 norm. This step reduces scale effects and stabilizes the geometry used in later stages.

Before applying t-SNE, the features are compressed by principal component analysis (PCA) to 128 dimensions. PCA is fit once on the normalized real-base subset, and the same linear projection is then applied to all samples. This linear reduction concentrates signal and suppresses high dimensional noise, which improves the stability and efficiency of the non-linear embedding.

For each point in the PCA space we form a local neighborhood distribution using a Gaussian kernel centered on that point. The kernel bandwidth is chosen separately for each point by a binary search so that the resulting conditional distribution has the target perplexity, which acts as an effective neighbor count. We use a default perplexity of 30 and cap it so it does not exceed about one third of the number of fitted points minus one. The pairwise relations are then symmetrized by averaging the two directional conditionals to produce a single similarity value for each pair. In the 2D space we measure similarity with the Student t-kernel, which assigns higher similarity to nearby points and tapers slowly with distance. The 2D coordinates are initialized from the top principal components and refined for a fixed number of iterations using the standard *t*-SNE update with early exaggeration in the initial phase and an automatic learning rate schedule. For large data, we fit *t*-SNE on a uniform random subset with a fixed seed and place the remaining points by out of sample interpolation. Each held out point is mapped to the inverse distance weighted average of the 2D coordinates of its k-nearest fitted neighbors found in the PCA space, with a small positive constant added to distances for numerical stability. The result is a two-dimensional layout that preserves local neighbor structure and is suitable for visualization and downstream analysis.

11. It could be useful to add Inception Score (IS) curves alongside FID for dual-metric convergence.

Response:

Thank you for your comment. As the reviewer noted, Inception Score (IS) and FID are the two most common image-generation metrics. We initially planned to report both, but in our simulations IS behaved inconsistently with perceived image quality, whereas FID tracked quality reliably.

"Though this has been pointed out elsewhere (Rosca et al., 2017), it is worth restating: applying the Inception Score to generative models trained on datasets other than ImageNet gives misleading results. The most common use of Inception Score on non-ImageNet datsets is for generative models trained on CIFAR-10, because it is quite a bit smaller and more manageable to train on than ImageNet. We have also seen the score used on datasets of bedrooms, flowers, celebrity faces, and more. The original proposal of the Inception Score was for the evaluation of models trained on CIFAR-10." [R1]

As discussed in [R1], applying IS to datasets other than ImageNet can yield misleading results, especially for single-class datasets (e.g., faces in CelebA), where class-diversity signals do not reflect generative quality. Consistent with recent image-generation papers using single-class datasets, we therefore report FID only. Like our work, many papers [R2-R4] that use a single-class dataset exclude the Inception Score and report only FID. FID is a representative metric for evaluating the diversity of generated images. We respectfully ask the reviewer to consider that our manuscript focuses on achieving diverse image generation via optimization of the random sampling (random-number) process, and we hope you will understand the resulting difficulty. We also believe that reporting only FID does not materially detract from the essence of this study. We again thank you for the helpful comment.

[R1] S. Barratt, and R. Sharma, "A Note on the Inception Score," *arXiv preprint*, arXiv:1801.01973, 2018.

[R2] Y. Jiang, S. Chang, and Z. Wang, "TransGAN: Two Pure Transformers Can Make One Strong GAN, and That Can Scale Up," 35th Conference on Neural Information Processing Systems (NeurIPS), 2021.

[R3] H. Zhu, W. Wu, W. Zhu, L. Jiang, S. Tang, L. Zhang, Z. Liu, and C. C. Loy, "CelebV-HQ: A Large-Scale Video Facial Attributes Dataset," European Conference on Computer Vision (ECCV), 2022.

[R4] C. Yu, et al., "Towards High-Fidelity Text-Guided 3D Face Generation and Manipulation Using only Images," Proceedings of the IEEE/CVF International Conference on Computer Vision (ICCV), 2023.

Corresponding change in manuscript: Yes

Location of Change: Results – (page 18)

We report FD score rather than the Inception Score for MNIST because Inception Score relies on an ImageNet-pretrained classifier that is poorly matched to 28×28, single-domain digit images, leading to misleading results [51].

Location of Change: Results – (page 19)

We report FID as the primary quantitative metric for generated CelebA images because, in line with prior observations that the Inception Score can be unreliable and misleading on non-ImageNet, single-class datasets, We report FD score rather than the Inception Score for MNIST because Inception Score relies on an ImageNet-pretrained classifier that is poorly matched to 28×28, single-domain digit images, leading to midleading results [54]. whereas FID more faithfully tracks perceptual quality and diversity [54-57].

12. Please specify read-pulse amplitude/width used during VMM.

Response:

Thank you for your comment. In our experiments, the read voltage amplitude was 2 V and the pulse width was 10 ms. Please refer to the revised manuscript to find the modification.

Corresponding change in manuscript: Yes

Location of Change: Results – (page 8)

For decoder VMM we operate at a low read voltage of 2 V, 10 ms to stay in the RTN free regime and to reduce power and IR drop while preserving linear accumulation.

13. Is there an error in the time unit of the PUND image in Figure 3b? Can the ferroelectric polarization reversal process last for such a long time?

Response:

Thank you for your comment. We appreciate your careful observation regarding the time unit in Figure 3b.

There was a labeling error in the original figure: the *x*-axis should be μs (microseconds), not s (seconds). The polarization reversal occurs on the microsecond timescale in HZO under our measurement conditions.

We have corrected the axis label and updated the caption and text in the revised manuscript.

Corresponding change in manuscript: Yes

Location of Change: Fig. 3

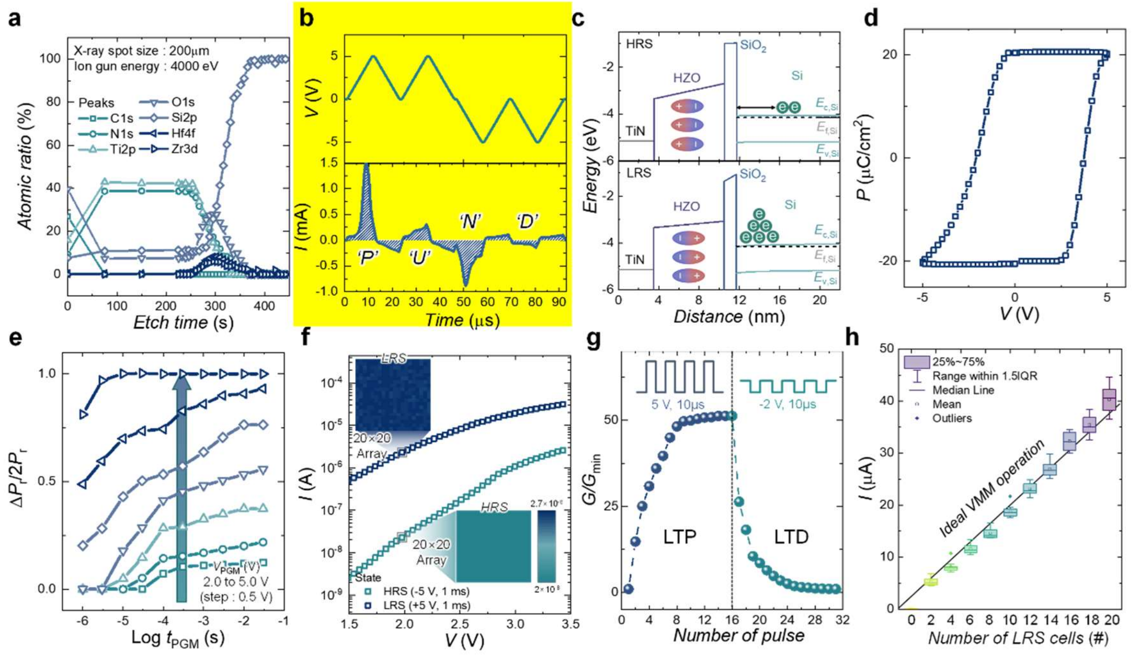

Figure 3. Overview of the structural, electrical, and functional characteristics of the FTJ. a Depth XPS analysis results, confirming the TiN–HZO–SiO_2–n^+ poly-Si structure. **b** PUND pulse waveforms (top) and *I* curves (bottom) showing polarization switching in the HZO layer. **c** Band diagrams of FTJ under different polarization states. HRS (top) and LRS (bottom). **d** *P-V* curve showing stable remnant polarization ($2P_r$ > 40 μC/cm²) in an HZO layer. **e** $\Delta P/2P_r$ versus t_{PGM} as a parameter of V_{PGM}. **f** *I-V* curves of the FTJ in LRS and HRS states. The inset shows the conductance map of the 20×20 NOR FTJ array, with a clear TER ratio (> 70), indicating distinct LRS and HRS states. **g** LTP-LTD characteristics of the FTJ. **h** VMM results from the 20×20 NOR FTJ array, demonstrating highly uniform VMM operation.

14. When the reading voltage exceeds 3.4V, does the RTN still exist?

Response:

We thank the reviewer for this insightful question. As the reviewer pointed out, bias dependent RTN behavior is important for clarifying the device physics. Fig. 5(c) shows the extracted dwell times as a function of read voltage. As the bias increases, the field tilts the barrier between the dominant HZO trap and the TiN electrode, making trapping and detrapping asymmetric so that τ_L decreases and τ_H increases. Around $V \approx 3.2$ V the two times are comparable and clear two level RTN appears. At lower bias less than or equal to 2.9 V or higher bias greater than or equal to 3.5 V one transition dominates, the occupancy collapses to a single state, and RTN disappears.

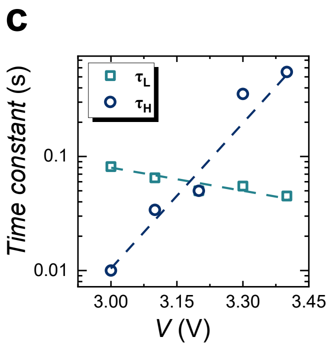

Fig. 5c Relationship between extracted τ_H and τ_L as a function of *V*.

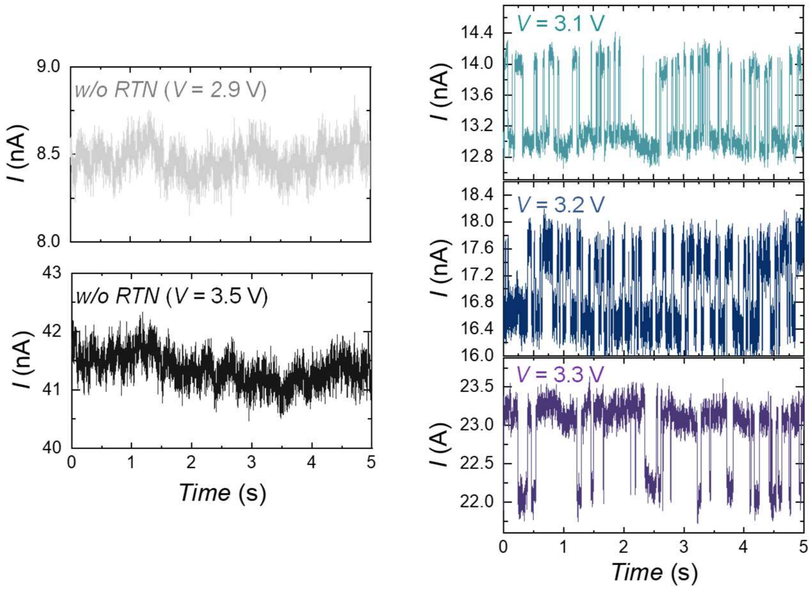

Fig. R15. DC read-current traces at various biases. Time-domain read-current signals were recorded for 5 s at 3200 Hz under different read voltages. The left panels show the RTN-free regions (*V*=2.9 V and *V* =3.5 V), exhibiting 1/f-like noise without distinct two-level switching. The right panels show the RTN-active region (*V*=3.1–3.3 V), where clear two-level fluctuations appear.

To verify this interpretation in the time domain, we added DC read current measurements. Fig. R15 shows read

current traces recorded for 5 s at 3200 Hz across bias. The left panels at $V = 2.9$ V and $V = 3.5$ V show RTN free $1/f$ like fluctuations, while the right panels at $V = 3.1$ to 3.3 V show distinct two-level switching. This confirms that RTN appears only within a mid-bias window. Both the high bias and low bias regimes are RTN free and thus suitable for deterministic VMM. For VMM we use a low read voltage of 2 V to maximize power efficiency and linear accumulation, while RTN sampling is carried out near 3.2 V where τ_L is approximately equal to τ_H. Following the reviewer's suggestion, we have added the corresponding explanation in the revised manuscript. Please refer to the revised manuscript for these updates.

Corresponding change in manuscript: Yes

Location of Change: Results – (page 12)

When the bias moves away from this alignment window (V=3.1 to 3.3 V) the two rates become highly imbalanced and τ_L and τ_H diverge, the two level signature collapses into a single preferred state, and the noise becomes $1/f$ due to Poole Frenkel assisted transport through a distributed ensemble of traps. This bias dependent mechanism links the band alignment in Fig. 4(i) and the dwell time trends in Fig. 4(g) to the observed change from RTN to $1/f$ noise. Note that RTN appears only in the mid bias window around 3.1 to 3.3 V where τ_H and τ_H are comparable, and it is suppressed at or below 2.9 V and at or above 3.5 V. Therefore, decoder VMM is performed at a low read voltage of 2 V in the RTN free regime to reduce power and IR drop while preserving linear accumulation, and RTN is utilized near 3.2 V for stochastic latent sampling.

15. In the manuscript, the author has cited some references to explain the process of the transition between 1/f noise and RTN noise. Could some additional explanations be provided in the main text to facilitate readers' understanding?

Response:

Thank you for your comment. We appreciate your question regarding the transition from $1/f$ noise to RTN ($1/f^2$), and we agree that clarifying the physical origin will improve readability.

In our devices, RTN arises from electron exchange between the TiN electrode and a dominant trap in the HZO tunneling layer, producing discrete two-level current fluctuations with a characteristic $1/f^2$ spectrum. The dwell times of the two RTN levels are governed by the trapping time constant (τ): a longer τ yields a longer low-current state, whereas a shorter τ extends the high-current state. By tuning the bias voltage, we modulate τ and thereby the RTN balance, which in turn improves the quality of VAE-generated images (see Fig. 5).

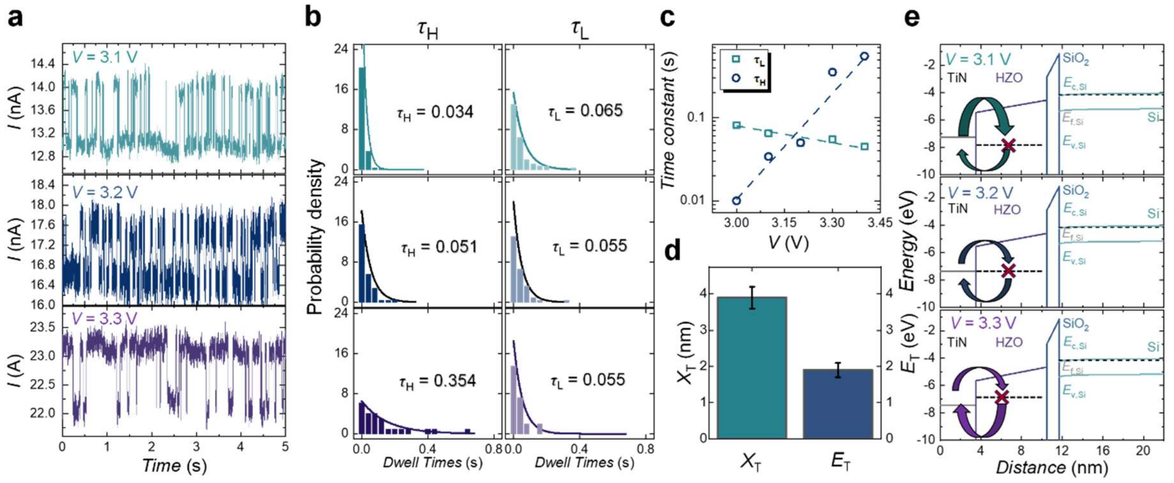

Figure 5. Voltage-dependent RTN characteristics and trap analysis in the FTJ. a DC transient measurements at different voltages (V = 3.1 V, 3.2 V, 3.3 V), showing varying dominance of low and high current states. **b** Histograms of time durations in high (τ_H) and low (τ_L) current states, fitted to Poisson distributions. **c** Relationship between extracted τ_H and τ_L as a function of V. **d** Extracted X_T and E_T of the dominant trap causing RTN in the FTJ. **e** Energy band diagrams of the FTJ under different applied voltages (V = 3.1 V, 3.2 V, and 3.3 V).

Conversely, in voltage ranges where the trap energy level in HZO differs significantly from the TiN Fermi level (e.g., V < 3.0 V or V > 3.4 V, see Fig. 4), the dominant trap does not effectively participate in electron exchange. In this regime, carrier transport is dominated by Poole–Frenkel emission from a broader ensemble of traps, leading to continuous $1/f$ noise rather than discrete RTN.

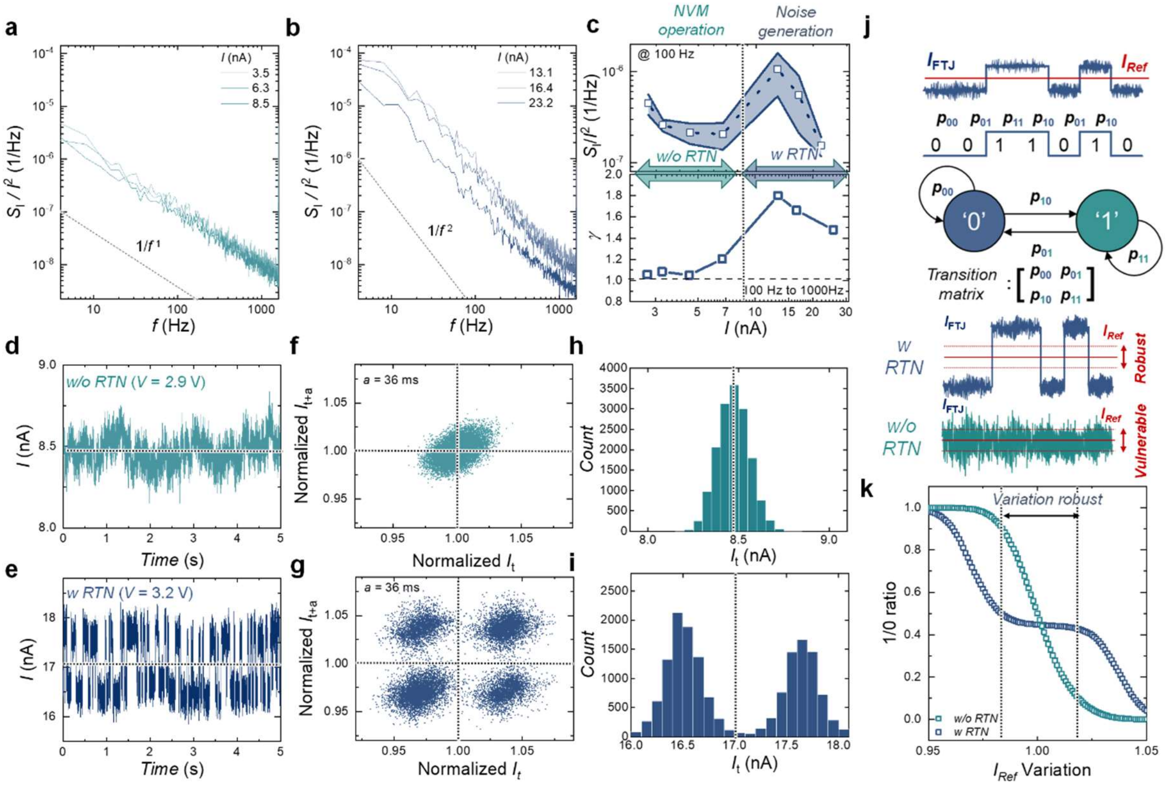

Figure 4. Analysis of RTN behavior and noise mechanisms in the FTJ with varying electrical conditions. a Relationship between S_I/I^2 and frequency for currents I < 10 nA, showing the dominance of 1/f noise. **b** Relationship between S_I/I^2 and frequency for currents I > 10 nA, exhibiting 1/f^2 noise indicative of RTN presence. **c** Plots of S_I/I^2 versus current at 100 Hz (top) and γ versus current (bottom), highlighting a change in the noise mechanism around I = 10 nA. **d** DC transient measurement at V = 2.9 V, showing no RTN behavior. **e** DC transient measurement at V = 3.2 V, clearly exhibiting RTN. **f** Time-lag plot at V = 2.9 V. **g** Time-lag plot at V = 3.2 V. **h** Histogram of current distribution at V = 2.9 V. **i** Histogram of current distribution at V = 3.2 V. **j** Illustration of generating a 0/1 sequence based on the FTJ current response, modeled as a Markov chain. **k** Plot of the ratio of '0's to '1's as a function of I_{Ref}, demonstrating improved state separation and robustness due to the presence of RTN.

We have added this voltage-dependent explanation to the revised manuscript so readers can more intuitively understand how trap-mediated tunneling underlies the transition from 1/f noise to RTN.

Corresponding change in manuscript: Yes

Location of Change: Results – (page 12)

When the bias moves away from this alignment window (V=3.1 to 3.3 V) the two rates become highly imbalanced and τ_L and τ_H diverge, the two level signature collapses into a single preferred state, and the noise becomes 1/f due

to Poole Frenkel assisted transport through a distributed ensemble of traps. This bias dependent mechanism links the band alignment in Fig. 4(i) and the dwell time trends in Fig. 4(g) to the observed change from RTN to 1/*f* noise. Note that RTN appears only in the mid bias window around 3.1 to 3.3 V where τ_H and τ_H are comparable, and it is suppressed at or below 2.9 V and at or above 3.5 V. Therefore, decoder VMM is performed at a low read voltage of 2 V in the RTN free regime to reduce power and IR drop while preserving linear accumulation, and RTN is utilized near 3.2 V for stochastic latent sampling.

16. Why does the frequency in Figure 4c increase from 100Hz to 1000Hz?

Response:

Thank you for your comment. We appreciate your question regarding Fig. 4c and agree that our original caption could be clearer.

In Fig. 4c, "100–1000 Hz" denotes the frequency window used to extract the exponent γ from the normalized power spectral density, $S_I/I^2 \propto 1/f^\gamma$. We estimate γ by fitting $\log(S_I/I^2)$ versus $\log f$ within that band, not by implying that the device operating frequency increases from 100 to 1000 Hz. This window avoids low-frequency drift and mains interference below 100 Hz and remains above the instrument noise floor where RTN ($1/f^2$) and $1/f^1$ behavior are reliably measured.

We have revised the inset label to "fit range: 100–1000 Hz" and added a clarifying sentence in the main text of the revised manuscript.

Corresponding change in manuscript: Yes

Location of Change: Fig. 4c

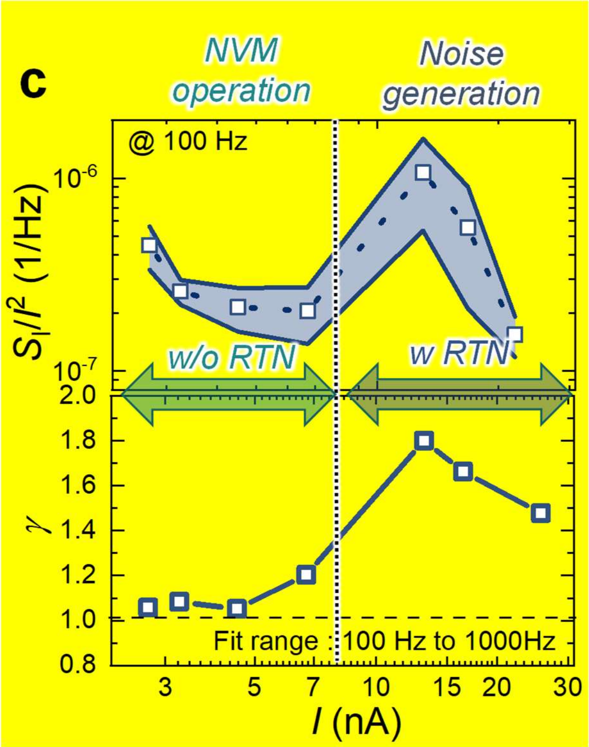

Fig. 4c Plots of S_I/I^2 versus current at 100 Hz (top) and γ versus current (bottom), highlighting a change in the noise mechanism around $I = 10$ nA. The value of γ is fitted in the 100–1000 Hz frequency range.

Location of Change: Results – (page 9)

We extracted the exponent γ by fitting $\log(S_I/I^2)$ versus $\log f$ within 100 to 1000 Hz to minimize low-frequency drift and measurement noise.

17. Some recent advances about the application of noise in ferroelectric memory should be included, such as:

[1] Y. Huang, Q. Wu, T. Gong, J. Yang, Q. Luo, M. Liu, Nature Communications 2025, 16.

[2] M. Song, R. H. Koo, J. Kim, C. H. Han, J. Yim, J. Ko, S. Yoo, D. H. Choe, S. Kim, W. Shin, D. Kwon, Nat Commun 2025, 16, 6879.

Response:

We thank the reviewer for this helpful suggestion. As the reviewer pointed out, recent studies have explored the use of intrinsic noise in ferroelectric devices for probabilistic computing. Huang et al. (Nat. Commun. 2025, 16) and Song et al. (Nat. Commun. 2025, 16, 6879) demonstrated ferroelectric implementations of Bayesian neural networks in which device noise is engineered to realize continuous analog weight distributions for uncertainty-aware classification. These works highlight the potential of ferroelectric noise for probabilistic inference, but they focus on continuous weight modulation rather than discrete sampling and address classification tasks rather than image generation. In contrast, our work represents, to our knowledge, the first hardware implementation of image generation, where voltage-controlled RTN in hafnia FTJs provides discrete stochastic sampling of latent vectors and deterministic VMM decoding within the same array. We have added this discussion and the recommended references to the Introduction of the revised manuscript. Please refer to the revised manuscript for these updates.

Corresponding change in manuscript: Yes

Location of Change: Introduction – (page 3)

While recent studies leverage device noise to realize continuous weight distributions for classification, our work focuses on hardware-native generation by using RTN for latent sampling and FTJ-based VMM for decoding within a single array [21,22].

Overall, this is a well-executed and impactful study that bridges device-level innovation with neural network applications, making it highly relevant to the fields of neuromorphic computing, ferroelectric memory, and probabilistic AI. The findings are technically sound and experimentally well-supported, and the manuscript should be considered for publication after addressing the minor points mentioned above.

We, the authors, send the deepest appreciation for the encouragement and comments again.

Reviewer #2

This paper presents an array-level demonstration of an FTJ-based design that supports both stochastic sampling for latent-variable inference and deterministic computing for vector matrix multiplication. The key contribution is the circuit-level demonstration and analysis of the RTN effect on latent sampling. Moreover, the voltage-dependent behavior within FTJ is intrinsic and matches the features of stochastic and deterministic computing.

Strengths

1. Demonstrating stochastic and deterministic operations on the same FTJ array is compelling and reduces system complexity.

2. Directly connecting RTN characteristics to sampling behavior provides a useful bridge between device physics and VAE tasks.

3. The experiments include a thorough evaluation across different voltages and conditions.

Here are a few questions and comments:

Response:

We, the authors, are very much thankful for the detailed comments and instructive suggestions. We have revised the manuscript in light of the useful comments and suggestions of the reviewer. Each comment and suggestion has been followed and addressed in the response attached below.

1. The dual functionality of the FTJ has been successfully demonstrated, and the analysis of the optimal operating voltage for effective VAE performance (i.e., balanced tau_h and tau_l) is shown in Figures 5 and 6 (particularly 5c and 6d). Does the 3.2 V result from the trap analysis generally apply to the entire 20 *20 array? Are there notable cell-to-cell variations within the array? If across different arrays, will this voltage result fluctuate?

Response:

Thank you for your comment. As the reviewer pointed out, clarifying whether ALD regulates RTN and explaining how controllability is achieved is important. Fig. R1(a) shows an ellipsometry thickness map of ALD HZO measured at 49 points across a 6-inch wafer, while Fig. R1(b) plots thickness versus radial distance from the wafer center. The data confirm high wafer-scale uniformity with only a slight decrease in thickness toward the edge, and the positions of the ten devices (D1–D10) used for RTN characterization are indicated on the map.

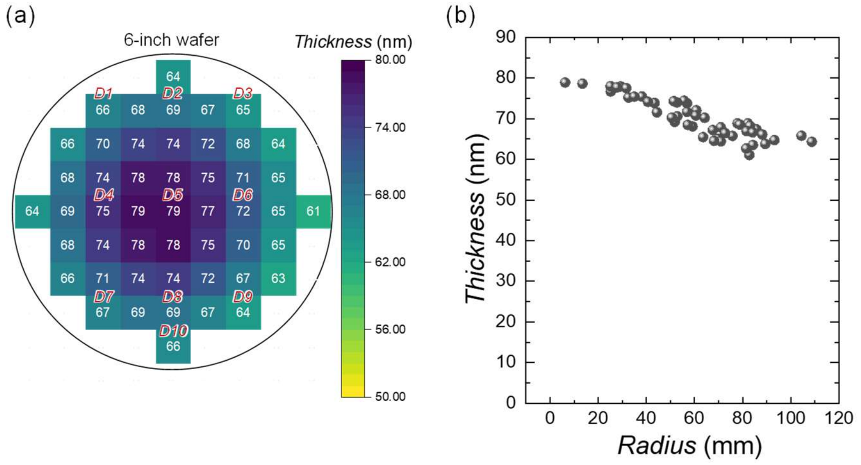

Fig. R1. Wafer-scale thickness uniformity of ALD HZO. (a) Six-inch wafer map showing ellipsometry-measured HZO thickness at 49 sampled points. The locations of the 10 devices (D1 to D10) used for RTN characterization are also marked on the map. (b) Thickness versus radial distance from the wafer center, showing a slight decreasing trend toward the edge.

Fig. R2(a) and Fig. R2(b) then show, for these ten devices biased at $V = 3.28$ V, the histograms of τ_L and τ_H with Poisson fits, indicating a robust two-state RTN process with nearly balanced occupancy at this bias. Fig. R3(a) shows the applied read voltage per device used for the measurements, and Fig. R3(b) shows the fitted time constants τ_L and τ_H per device at the same bias. A small but finite spread is observed, which we attribute to ALD-related variations in trap depth and energy level. These minor differences did not affect CelebA generation performance in our study, although tighter control may be needed for more precision-demanding tasks. Motivated by this, we devised and validated a simple electrical calibration method during the revision.

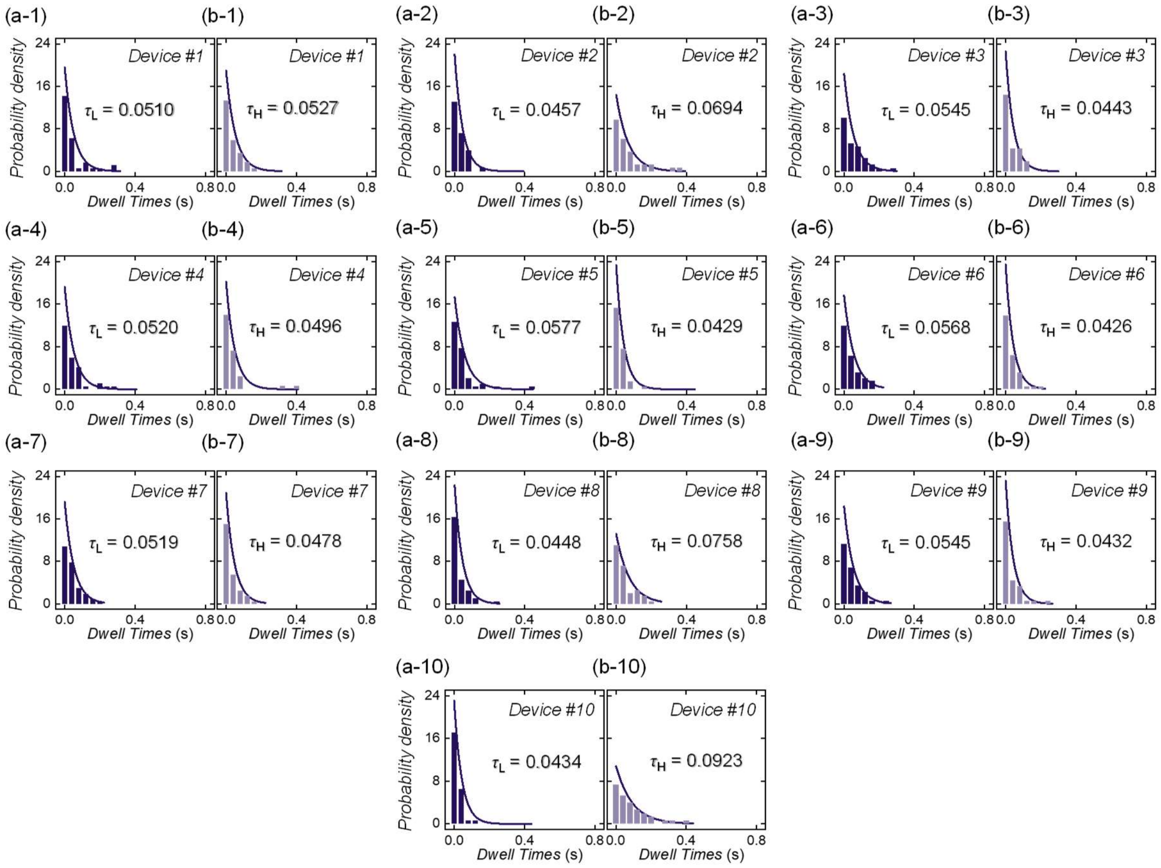

Fig. R2. RTN dwell-time statistics at a single bias (a) Histograms of the low-state dwell time τ_L for 10 devices measured at $V = 3.28$ V with Poisson (exponential) fits. (b) Histograms of the high-state dwell time τ_H for the same devices and bias with Poisson fits. Across devices the RTN statistics follow Poisson processes and the occupancy of the two levels is balanced at this bias.

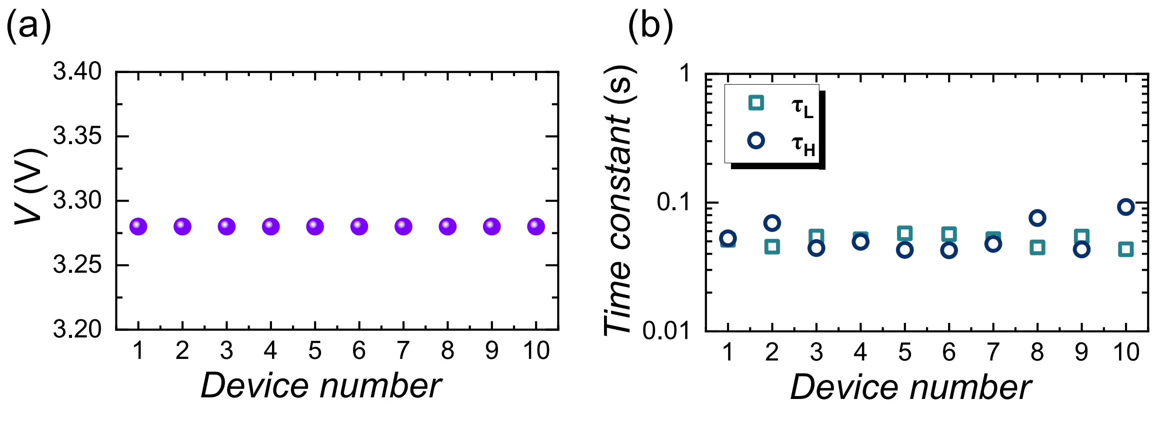

Fig. R3. Device-wise bias and fitted time constants. (a) Applied read voltage for each device used in Fig. R1. (b) Dwell-time constants extracted from Poisson fitting, reported per device for both τ_L and τ_H.

To verify this controllability, we fine-tuned the read bias in 0.001 V steps based on the imbalance between τ_L and τ_H. When τ_L exceeded τ_H, V was increased by 0.001 V; when τ_H was larger, V was reduced by 0.001 V. This adjustment was repeated until both dwell times converged within tolerance. Fig. R4(a) shows the τ_L histograms after per-device equalization within 3.25 to 3.30 V, and Fig. R4(b) shows the corresponding τ_H histograms, both exhibiting more balanced occupancy than in Fig. R2. Fig. R5(a) summarizes the optimized bias for each device after equalization, and Fig. R5(b) shows the fitted τ_L and τ_H values under these optimized conditions, now closely clustered compared with Fig. R3(b).

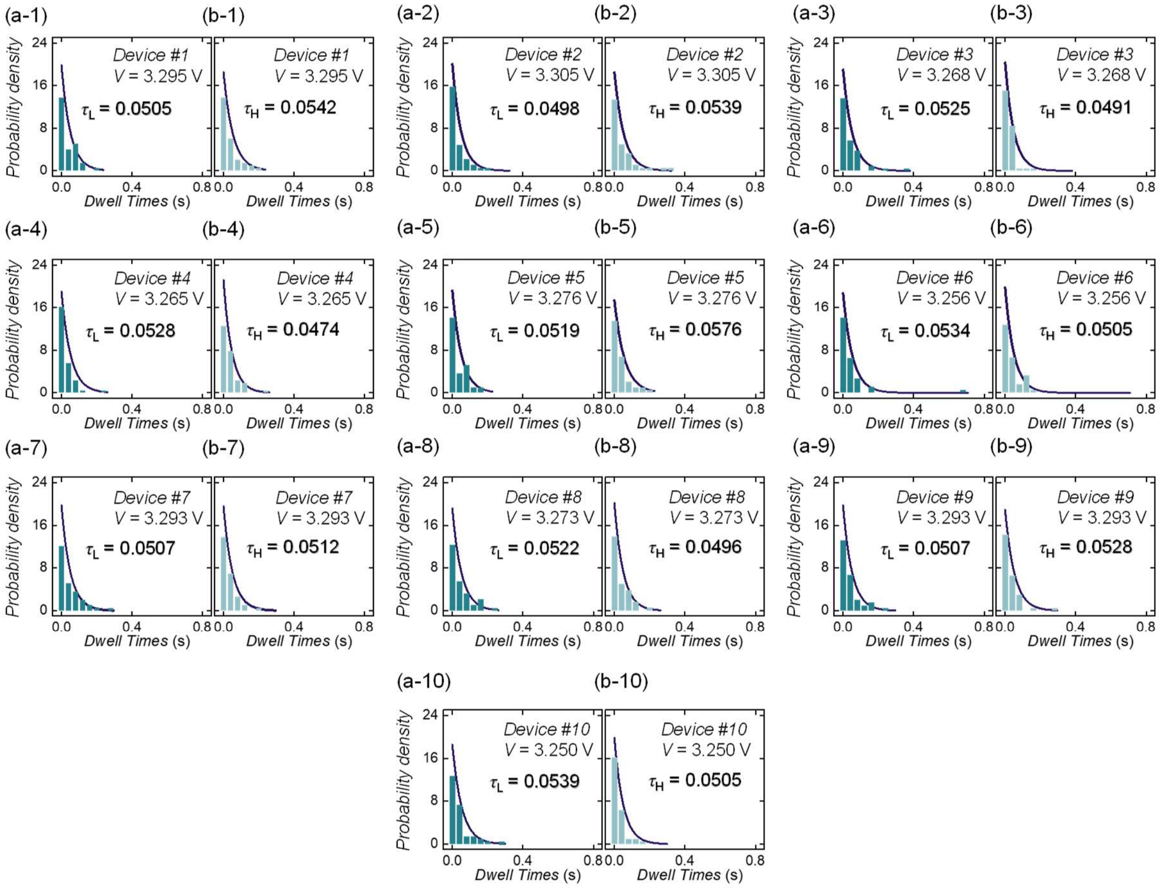

Fig. R4. Bias equalization across devices for balanced occupancy. (a) Histograms of τ_L with Poisson fits for 10 devices, each biased at its own equalized voltage in the range 3.25–3.30 V to balance state occupancy. (b) Corresponding τ_H histograms with Poisson fits. Compared with Fig. R1, per-device bias equalization yields more balanced occupancy. Inset shows the applied voltage for each device.

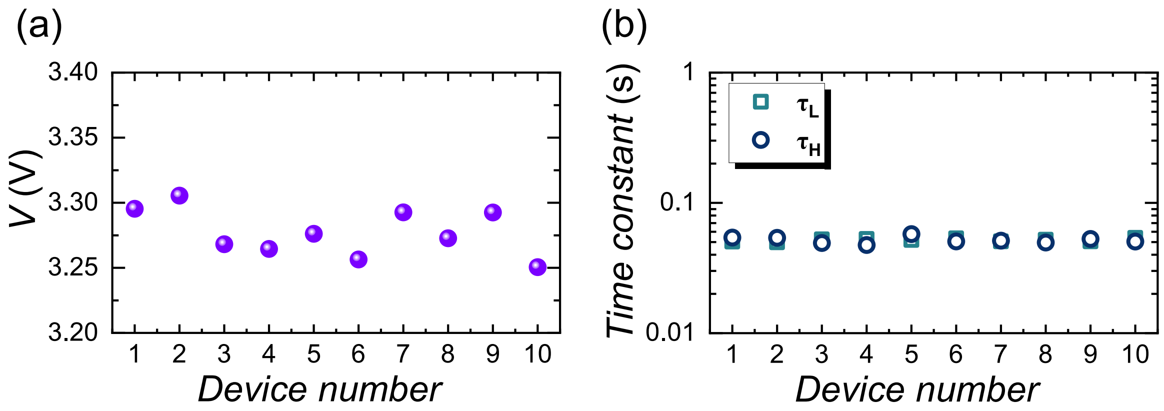

Fig. R5. Summary of equalized bias and fitted constants. (a) Optimized read voltage for each device. (b) Dwell-time constants τ_L and τ_H under the optimized bias for each device, showing markedly tighter clustering than in Fig. R2b.

These results align with the RTN mechanism described in the manuscript, where small field adjustments shift the relative alignment between the trap level and the TiN work function, tuning the transition probabilities toward a 50:50 ratio. In practice, such voltage trimming can be implemented per cell or per column using existing word-line or bit-line DAC architectures, enabling parallel generation of uniform random bitstreams even with minor ALD-induced variability.

Following the reviewer's suggestion, we have added a concise description of the ALD-related variation, the voltage adjustment procedure, and its system-level feasibility in the revised manuscript. Please refer to the revised manuscript to find the modifications.

Corresponding change in manuscript: Yes

Location of Change: Supplementary Figure 12-16

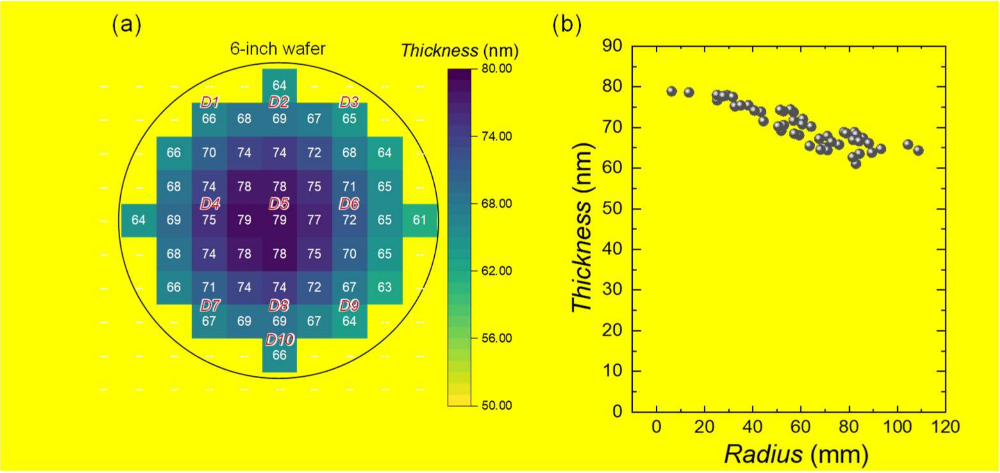

Supplementary Figure 12. Wafer-scale thickness uniformity of ALD HZO. (a) Six-inch wafer map showing

ellipsometry-measured HZO thickness at 49 sampled points. The locations of the 10 devices (D1 to D10) used for RTN characterization are also marked on the map. (b) Thickness versus radial distance from the wafer center, showing a slight decreasing trend toward the edge.

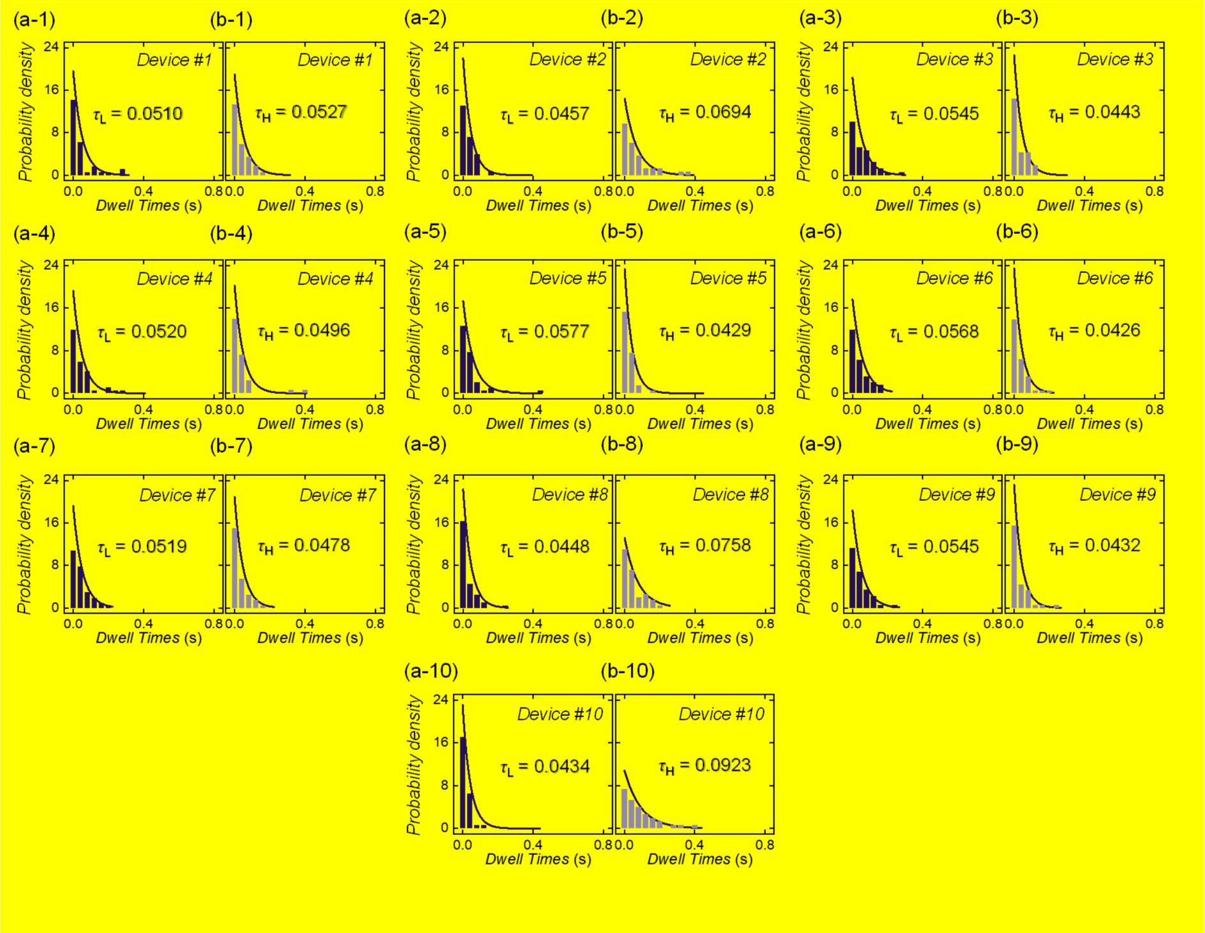

Supplementary Figure 13. RTN dwell-time statistics at a single bias (a) Histograms of the low-state dwell time τ_L for 10 devices measured at V = 3.28 V with Poisson (exponential) fits. (b) Histograms of the high-state dwell time τ_H for the same devices and bias with Poisson fits. Across devices the RTN statistics follow Poisson processes and the occupancy of the two levels is balanced at this bias.

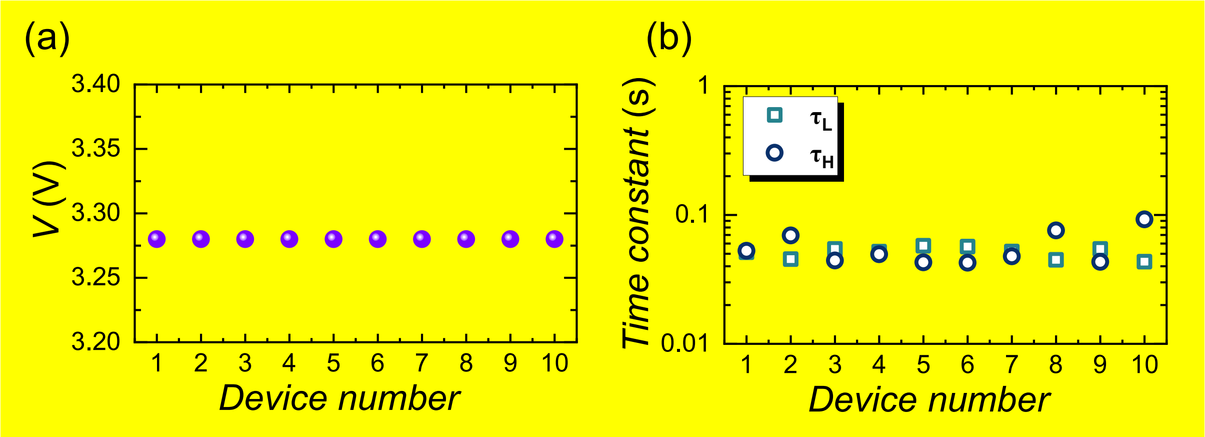

Supplementary Figure 14. Device-wise bias and fitted time constants. (a) Applied read voltage for each device used in Fig. R1. (b) Dwell-time constants extracted from Poisson fitting, reported per device for both τ_L and τ_H.

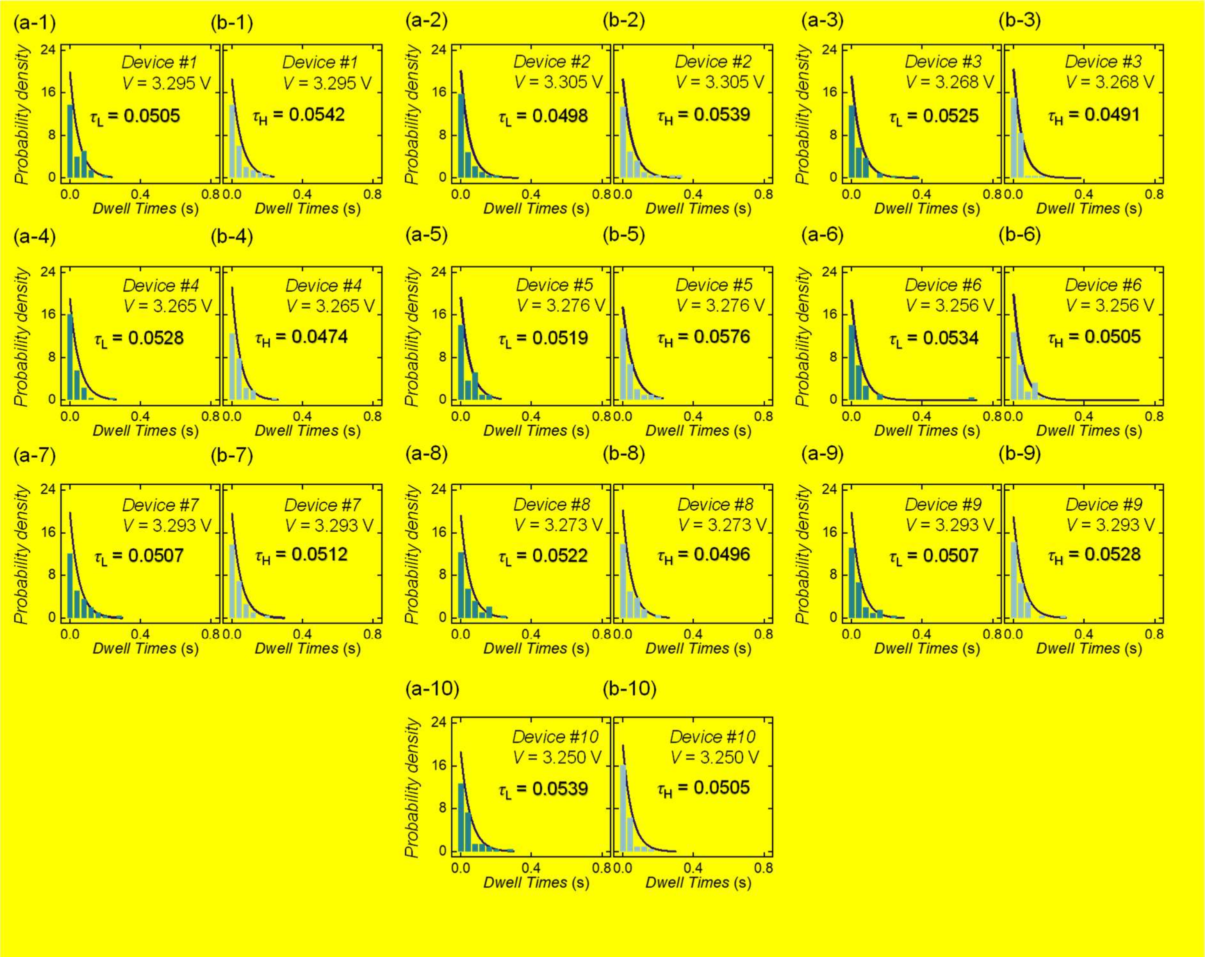

Supplementary Figure 15. Bias equalization across devices for balanced occupancy. (a) Histograms of τ_L with Poisson fits for 10 devices, each biased at its own equalized voltage in the range 3.25–3.30 V to balance state occupancy. (b) Corresponding τ_H histograms with Poisson fits. Compared with Fig. R1, per-device

bias equalization yields more balanced occupancy. Inset shows the applied voltage for each device.

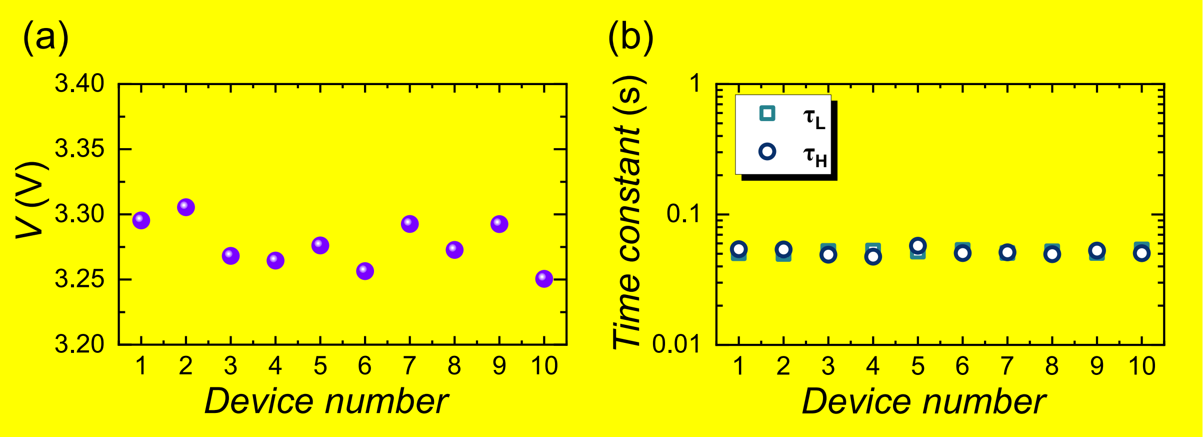

Supplementary Figure 16. Summary of equalized bias and fitted constants. (a) Optimized read voltage for each device. (b) Dwell-time constants τ_L and τ_H under the optimized bias for each device, showing markedly tighter clustering than in Fig. R2b.

Location of Change: Results – (pages 12-13)

To further validate that the RTN characteristics originate from ALD-related traps and remain controllable across the wafer, we measured the HZO thickness and RTN uniformity over a 6-inch wafer (Supplementary Fig. 12). The thickness map and device-wise dwell-time statistics (Supplementary Fig. 13 and Supplementary Fig. 14) confirm uniform Poisson-type RTN behavior with minor device-to-device variation caused by small differences in trap depth and energy level. By iteratively adjusting the read bias in 1 mV steps according to the τ_L/τ_H imbalance, the distributions were equalized across all devices (Supplementary Fig. 15 and Supplementary Fig. 16). This simple voltage-trimming method, readily implemented through word- or bit-line DAC control, enables parallel generation of uniform random bitstreams even with slight ALD-induced variability.

2. As discussed in the paper, Figure 4a and 4c indicate that I<10nA can be used for muliplition. Could the author explain more why the corresponding low-frequency noise can be ignored by the computation? Is there a threshold for S_I/(I^2) or gamma that indicates that the 1/f noise is not dominate?

Response:

Thank you for your comment. As the reviewer pointed out, it is important to clarify why low-frequency noise can be ignored during multiplication and to explain why the central limit theorem (CLT) does not apply to RTN.

For $1/f$ noise, the fluctuations are short-range correlated and stationary. When the read current is integrated over time during vector–matrix multiplication (VMM), the instantaneous variations are smoothed by temporal averaging. As the integration window becomes longer and more devices contribute to the summation, the accumulated signal approaches its mean according to the CLT, and the residual fluctuation becomes negligible for computation.

In contrast, RTN ($1/f^2$) originates from two discrete current states with long correlation times. Because successive transitions are not independent, the CLT does not hold even for long integration periods. The integrated output retains non-Gaussian statistics with memory of prior states, and the variance does not diminish with longer integration.

To verify this behavior, we used quantile-quantile (Q-Q) plots to compare the empirical distribution of integrated signals with a theoretical Gaussian. For each integration window, consecutive current samples were grouped into blocks of N samples, summed to form S_N, standardized as $Z_N = (S_N - \mu_N)/(\sigma_N^{1/2})$), and their empirical quantiles were plotted on the y-axis against the theoretical quantiles of a standard normal distribution on the x-axis.

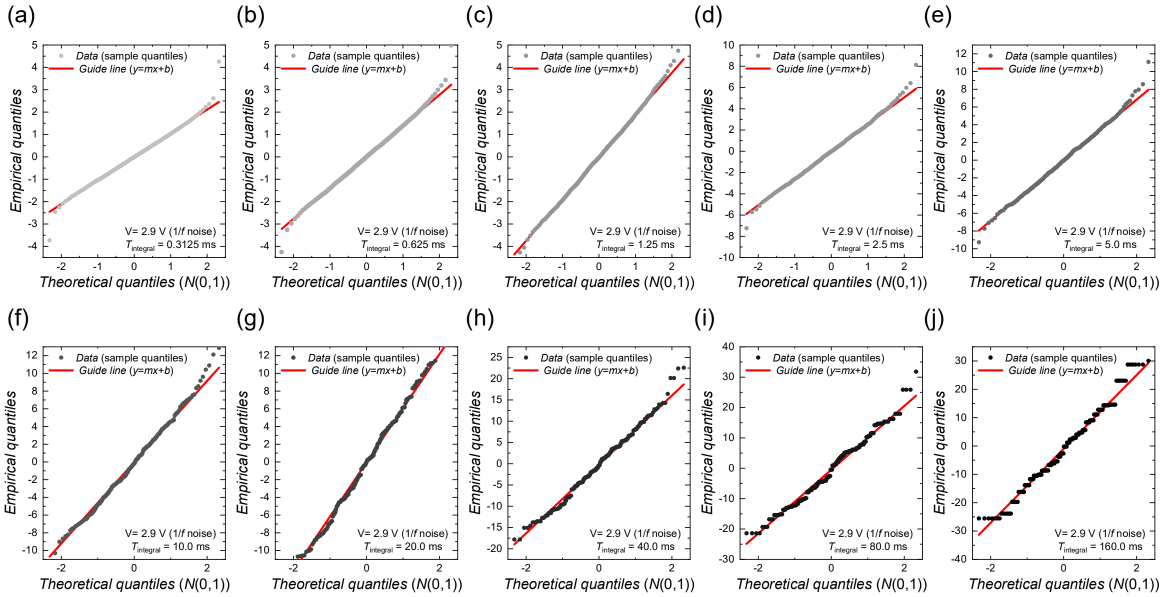

Fig. R16. Q-Q plots of the standardized block sums $Z_N = (S_N - \mu_N)/(\sigma_N^{1/2})$) for a $1/f$ noise signal ($V = 2.9$ V). The x-axis shows theoretical quantiles of $N(0,1)$, and the y-axis shows empirical quantiles of Z_N. Dots shows the empirical quantiles; the red line is a data-based guideline, $y = mx + b$. Panels correspond to the different

integration times: (a) 0.3125 ms, (b) 0.625 ms, (c) 1.25 ms, (d) 2.50 ms, (e) 5.0 ms, (f) 10.0 ms, (g) 20.0 ms, (h) 40.0 ms, (i) 80.0 ms, (j) 160.0 ms). The 1/*f* noise aligns with the 45-degree line even at short integration time, indicating rapid convergence to normality.

Fig. R16 shows the 1/*f* regime at $V = 2.9$ V, where the data points align with the 45-degree reference line even at short integration windows. This confirms that the integrated current follows a Gaussian distribution, consistent with the CLT, and that 1/*f* fluctuations are effectively averaged out during VMM. Fig. R17, Fig. R18, and Fig. R19 show the RTN regime at $V = 3.1$ V, 3.2 V, and 3.3 V. In these plots, the Q–Q curves exhibit persistent *S*-shaped curvature and heavy tails across all integration times, confirming that RTN maintains long-range correlation and does not converge to Gaussian statistics even under prolonged averaging.

These results demonstrate that deterministic VMM can reliably operate in the 1/*f* regime, where integration naturally suppresses fluctuations, whereas the RTN ($1/f^2$) regime retains its non-Gaussian statistics and is therefore reserved for stochastic sampling. Following the reviewer's suggestion, we have added this CLT-based explanation, detailed description of the Q–Q analysis method, and references to Fig. R16–R19 in the revised manuscript. Please refer to the revised manuscript to find the modifications.

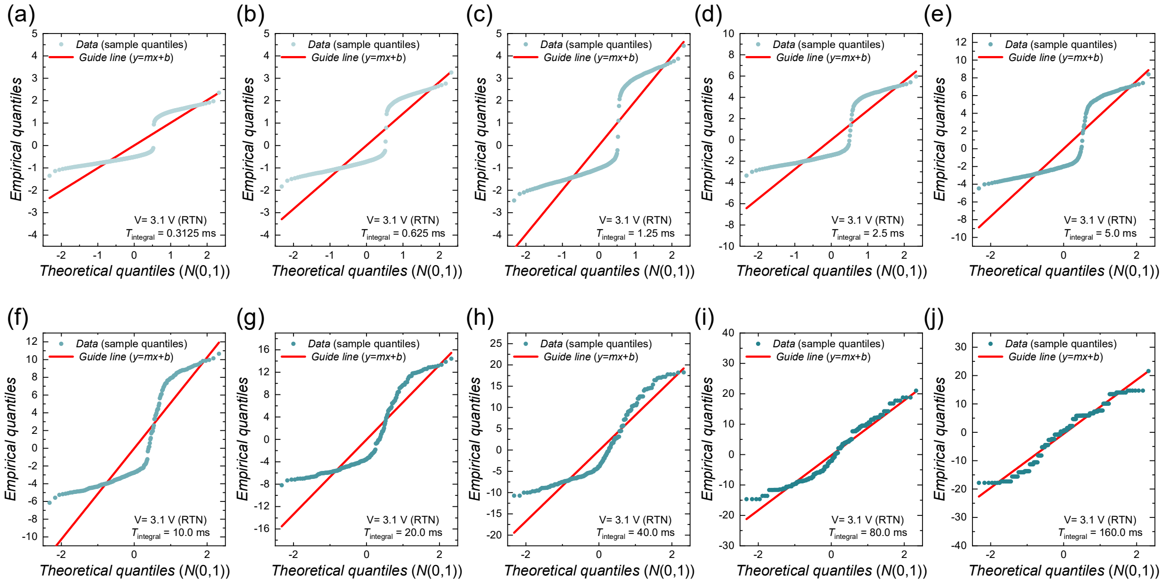

Fig. R17. QQ plots of the standardized block sums $Z_N = (S_N - \mu_N)/(\sigma_N^{1/2})$ for an RTN signal ($V = 3.1$ V). The *x*-axis shows theoretical quantiles of *N*(0,1), and the *y*-axis shows empirical quantiles of Z_N. Dots show the empirical quantiles; the red line is a data-based guideline, $y = mx + b$. Panels correspond to the different integration times: (a) 0.3125 ms, (b) 0.625 ms, (c) 1.25 ms, (d) 2.50 ms, (e) 5.0 ms, (f) 10.0 ms, (g) 20.0 ms, (h) 40.0 ms, (i) 80.0 ms, (j) 160.0 ms). The RTN signal retains an S-shaped curvature and heavy tails due to long-range correlation.

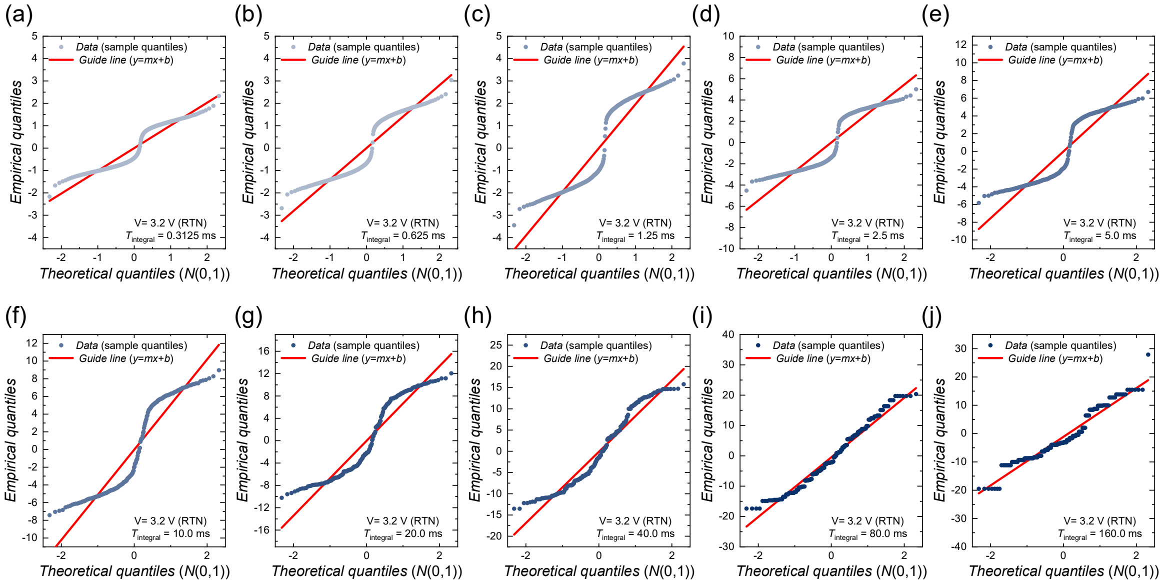

Fig. R18. QQ plots of the standardized block sums $Z_N = (S_N - \mu_N)/(\sigma N^{1/2})$ for an RTN signal (V = 3.2 V). The x-axis shows theoretical quantiles of $N(0,1)$, and the y-axis shows empirical quantiles of Z_N. Dots show the empirical quantiles; the red line is a data-based guideline, $y = mx + b$. Panels correspond to the different integration times: (a) 0.3125 ms, (b) 0.625 ms, (c) 1.25 ms, (d) 2.50 ms, (e) 5.0 ms, (f) 10.0 ms, (g) 20.0 ms, (h) 40.0 ms, (i) 80.0 ms, (j) 160.0 ms). The RTN signal retains an S-shaped curvature and heavy tails due to long-range correlation.

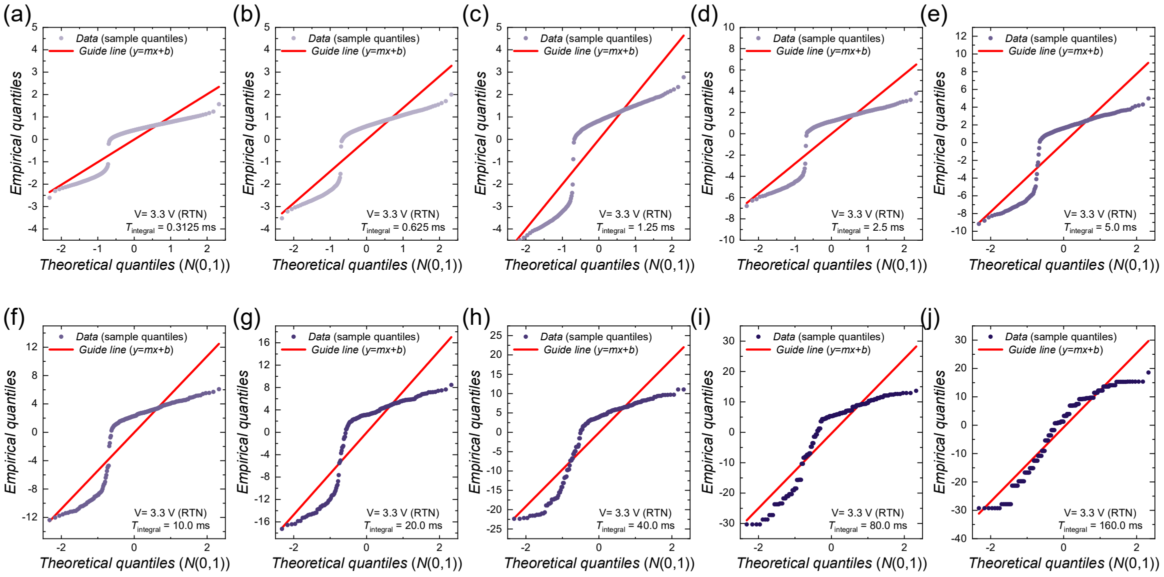

Fig. R19. QQ plots of the standardized block sums $Z_N = (S_N - \mu_N)/(\sigma N^{1/2})$ for an RTN signal (V = 3.3 V). The x-axis shows theoretical quantiles of $N(0,1)$, and the y-axis shows empirical quantiles of Z_N. Dots show the empirical quantiles; the red line is a data-based guideline, $y = mx + b$. Panels correspond to the different

integration times: (a) 0.3125 ms, (b) 0.625 ms, (c) 1.25 ms, (d) 2.50 ms, (e) 5.0 ms, (f) 10.0 ms, (g) 20.0 ms, (h) 40.0 ms, (i) 80.0 ms, (j) 160.0 ms). The RTN signal retains an S-shaped curvature and heavy tails due to long-range correlation.

Corresponding change in manuscript: Yes

Location of Change: Result – (page 13)

The ability to control RTN behavior through voltage adjustments provides an effective mechanism for neural network-based image generation systems. Firstly, by modulating the applied voltage, FTJs not only enable the generation of RTN but also allow for its suppression, enabling seamless transitions between stochastic and deterministic operations. We further note that deterministic VMM is reliable in the $1/f$ regime, whereas $1/f^2$ RTN is not suitable for VMM and is used for stochastic sampling. The central limit theorem rationale and the Q–Q analysis supporting this distinction are provided in Supplementary Fig. 17, 18, 19, 20 and Supplementary Note 1.

Location of Change: Supplementary Note 1

Supplementary Note 1. Central limit behavior and Q–Q analysis for 1/*f* and RTN regimes

We analyze the statistics of time integrated read current using a quantile–quantile framework and we connect the observed behavior to the applicability of the central limit theorem (CLT). The method is as follows. Consecutive current samples are grouped into non overlapping blocks of N points and summed to form S_N. The sums are standardized as $Z_N = (S_N - \mu_N)/(\sigma_N^{1/2}))$ where μ_N and σ_N are the empirical mean and standard deviation of S_N. We then plot the empirical quantiles of Z_N against the theoretical quantiles of a standard normal distribution. If the integrated signal is effectively Gaussian, the Q–Q points follow a straight line with unit slope. Deviations from linearity reveal non Gaussian tails or correlation that violates the independence assumption required for the central limit theorem.

Figure R17 shows the Q–Q plots for the $1/f$ regime at $V = 2.9$ V. The integration window is swept from 0.3125 ms to 160 ms. The points align closely with the 45 degree reference over all windows, which indicates rapid convergence toward a normal distribution under time integration. This behavior supports reliable deterministic accumulation during VMM in the $1/f$ regime.

Figures R18, R19, and R20 show the RTN regime at $V = 3.1$ V, $V = 3.2$ V, and $V = 3.3$ V. Each figure sweeps the same set of integration windows. The Q–Q curves display persistent S shaped curvature with heavy tails that remain even as the window increases. The curvature indicates that long range temporal correlation survives the integration and that successive samples are not independent. As a result Z_N does not converge to a standard normal distribution within practical windows. This behavior is consistent with a two level process whose dwell times are set by τ_L and τ_H and whose transitions retain memory over many samples.

Taken together these results explain the operating policy used in the manuscript. In the $1/f$ regime the time integration inherent to VMM averages out short range fluctuations so the output approaches its mean and deterministic accumulation is reliable. In the RTN regime the long correlation prevents convergence to a Gaussian

distribution within practical windows, so the integrated output retains stochasticity that is useful for latent sampling but not for precise multiplication. For this reason we operate the decoder VMM at low read voltage in the RTN free region (V= 2.0 V) and we use bias near 3.2 V to obtain balanced τ_L and τ_H for stochastic sampling.

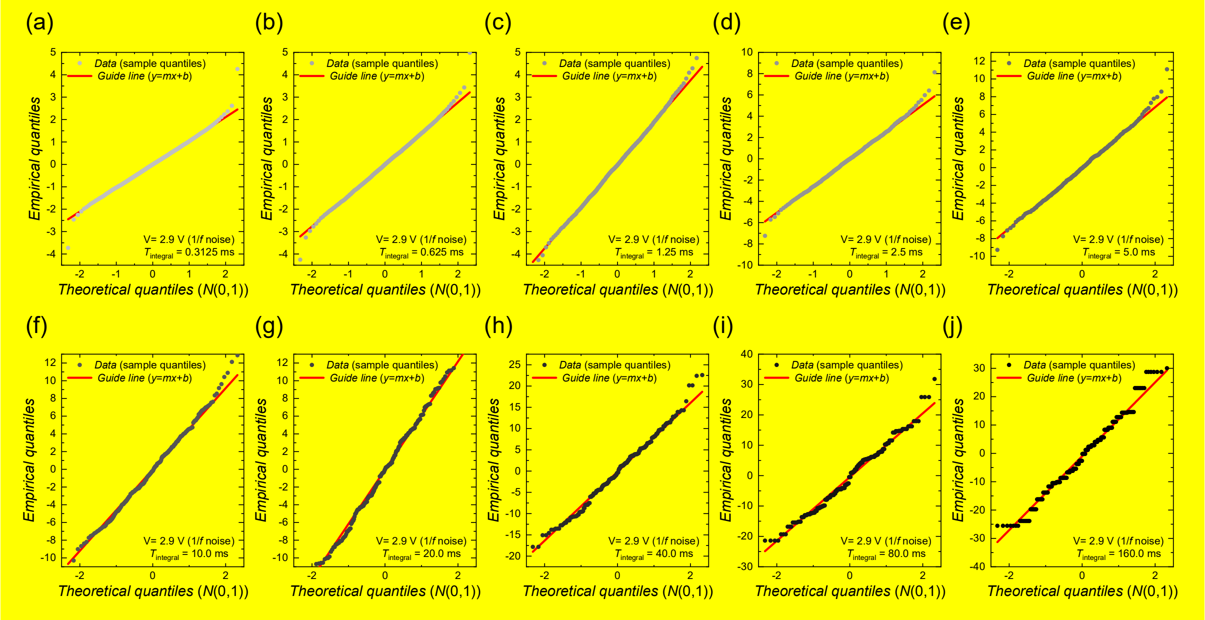

Supplementary Figure 17. Q-Q plots of the standardized block sums $Z_N = (S_N - \mu_N)/(\sigma_N^{1/2})$ for a 1/f noise signal (V = 2.9 V). The x-axis shows theoretical quantiles of N(0,1), and the y-axis shows empirical quantiles of Z_N. Dots shows the empirical quantiles; the red line is a data-based guideline, $y = mx + b$. Panels correspond to the different integration times: (a) 0.3125 ms, (b) 0.625 ms, (c) 1.25 ms, (d) 2.50 ms, (e) 5.0 ms, (f) 10.0 ms, (g) 20.0 ms, (h) 40.0 ms, (i) 80.0 ms, (j) 160.0 ms). The 1/f noise aligns with the 45-degree line even at short integration time, indicating rapid convergence to normality.

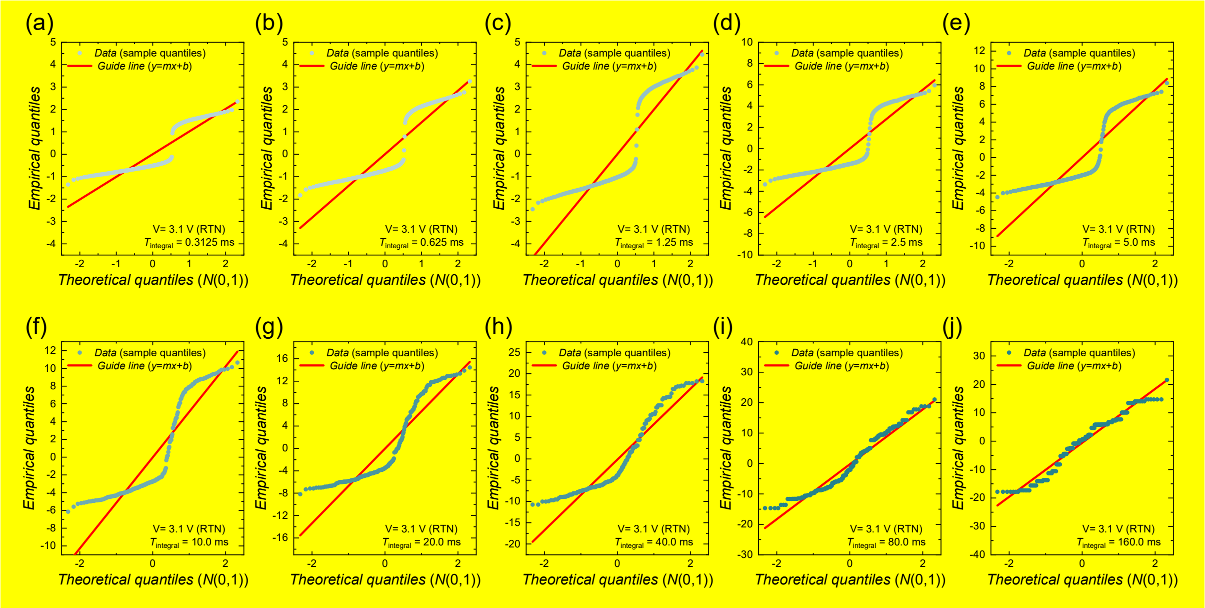

Supplementary Figure 18. QQ plots of the standardized block sums $Z_N = (S_N - \mu_N)/(\sigma_N^{1/2})$) for an RTN signal ($V$ = 3.1 V). The x-axis shows theoretical quantiles of N(0,1), and the y-axis shows empirical quantiles of Z_N. Dots show the empirical quantiles; the red line is a data-based guideline, $y = mx + b$. Panels correspond to the different integration times: (a) 0.3125 ms, (b) 0.625 ms, (c) 1.25 ms, (d) 2.50 ms, (e) 5.0 ms, (f) 10.0 ms, (g) 20.0 ms, (h) 40.0 ms, (i) 80.0 ms, (j) 160.0 ms). The RTN signal retains an S-shaped curvature and heavy tails due to long-range correlation.

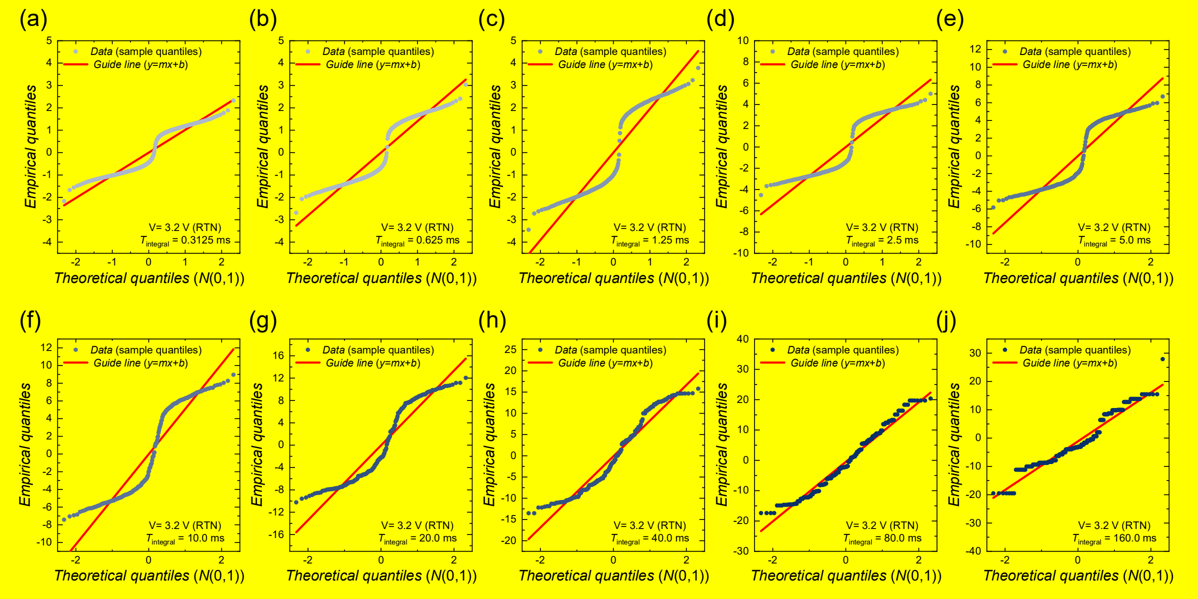

Supplementary Figure 19. QQ plots of the standardized block sums $Z_N = (S_N - \mu_N)/(\sigma N^{1/2})$ for an RTN signal ($V = 3.2$ V). The x-axis shows theoretical quantiles of $N(0,1)$, and the y-axis shows empirical quantiles of Z_N. Dots show the empirical quantiles; the red line is a data-based guideline, $y = mx + b$. Panels correspond to the different integration times: (a) 0.3125 ms, (b) 0.625 ms, (c) 1.25 ms, (d) 2.50 ms, (e) 5.0 ms, (f) 10.0 ms, (g) 20.0 ms, (h) 40.0 ms, (i) 80.0 ms, (j) 160.0 ms). The RTN signal retains an S-shaped curvature and heavy tails due to long-range correlation.

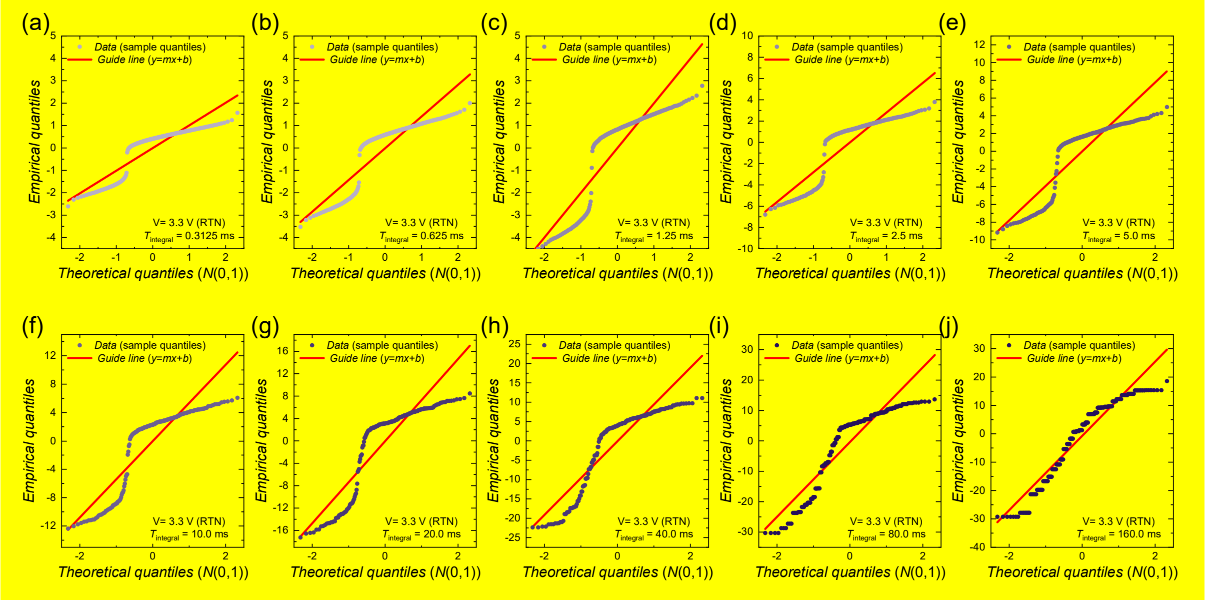

Supplementary Figure 20. QQ plots of the standardized block sums $Z_N = (S_N - \mu_N)/(\sigma N^{1/2})$ for an RTN signal (V = 3.3 V). The x-axis shows theoretical quantiles of N(0,1), and the y-axis shows empirical quantiles of Z_N. Dots show the empirical quantiles; the red line is a data-based guideline, $y = mx + b$. Panels correspond to the different integration times: (a) 0.3125 ms, (b) 0.625 ms, (c) 1.25 ms, (d) 2.50 ms, (e) 5.0 ms, (f) 10.0 ms, (g) 20.0 ms, (h) 40.0 ms, (i) 80.0 ms, (j) 160.0 ms). The RTN signal retains an S-shaped curvature and heavy tails due to long-range correlation.

3. The VAE used for MNIST generation (Supplementary Figure 10) contains fully connected layer with more than 400 parameters. How is parameter mapping managed in the proposed design?

Response:

Thank you for your comment. As the reviewer correctly points out, it is important to clarify which parts were implemented in hardware and how parameter mapping and VMM non-idealities were handled for the MNIST study. The RTN-based sampling block was physically implemented, as shown in Fig. 5a,b. By contrast, the decoder VMM path was not instantiated as a full hardware macro; rather, it was implemented in software based on the non-idealities measured from the fabricated 20 × 20 array.

To detail this assumption: device nonlinearity extracted from Fig. 3(g) was used to define a device-informed 4-bit (16-state) conductance partition for weights. The LTP and LTD update curves were fitted with $\beta_{\mathrm{LTP}} = 4.9$ and $\beta_{\mathrm{LTD}} = 8.2$, and weight targets followed these β-dependent update characteristics (instead of ideal uniform spacing). To capture array-level behavior during VMM, we incorporated an error model calibrated from Fig. 3(h) (20 × 20 array, 2 V read): each matrix–vector output yyy was multiplied by a random factor rand(1,σ) whose scale σ was obtained from the measured distribution of column-summed currents (Fig. 3(h)), i.e., $y \rightarrow y \cdot \mathrm{rand}(1,\sigma)$. This reproduces the observed spread while preserving the linear accumulation verified in Fig. 3(h) as the number of activated (LRS) cells increases at 2 V.

Following the reviewer's suggestion, we have clarified in the main text which components are implemented in hardware (RTN sampling; Fig. 5a,b) and which are evaluated in hardware-informed software (the decoder VMM, using a β-informed 4-bit conductance partition from Fig. 3g and an array-level error model calibrated from Fig. 3h). Please refer to the revised manuscript for these modifications.

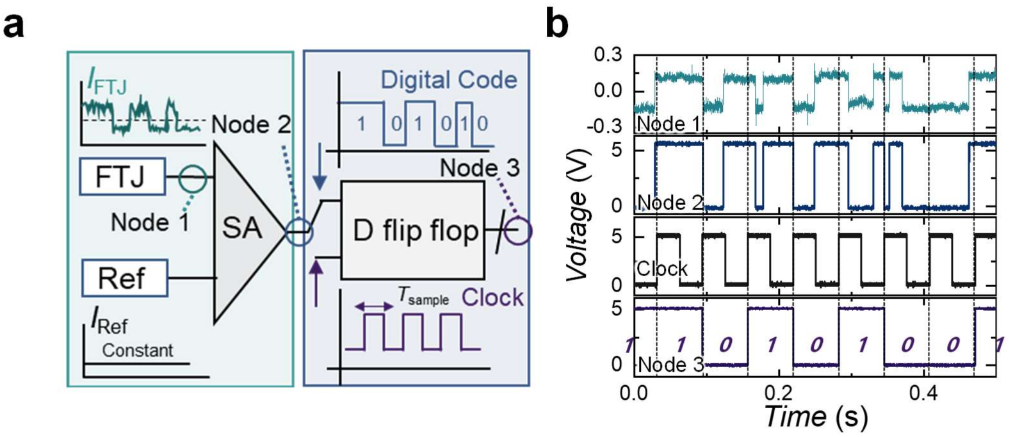

Figure 5. Circuit implementation and optimization of FTJ-based random number generation. (a) Schematic diagram of the FTJ-based circuit used to verify the output of a random source output. The circuit includes an FTJ, a sense amplifier, and a D flip-flop to generate a random bitstream. (b) Transient results of the

FTJ-based circuit, showing how RTN is converted into a voltage signal (Node 1), digitalized (Node 2), and outputted as a random sequence (Node 3).

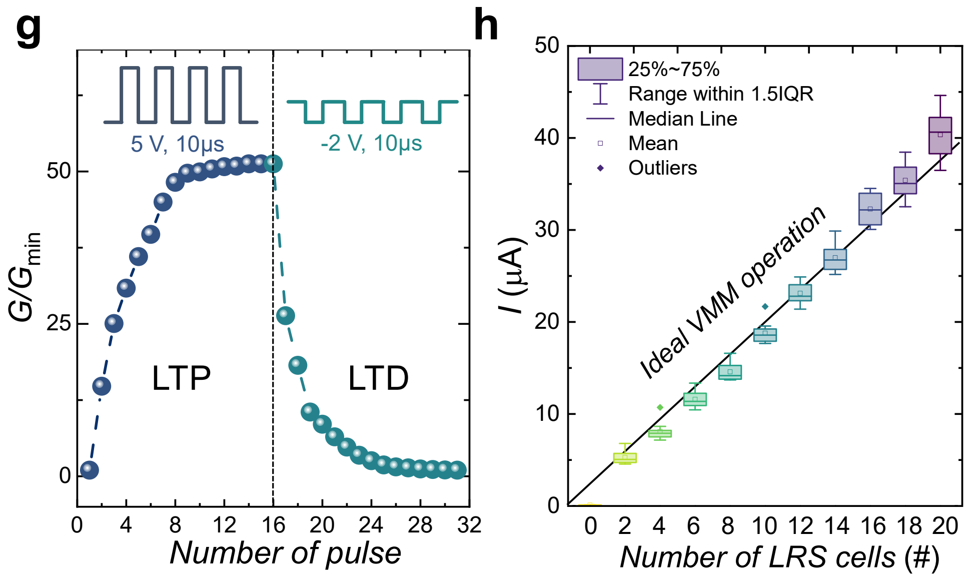

Figure 3 (g) LTP-LTD characteristics of the FTJ. (h) VMM results from the 20×20 NOR FTJ array, demonstrating highly uniform VMM operation.

Corresponding change in manuscript: Yes

Location of Change: Results – (page 8)

The update curves exhibit nonlinearity with β_{LTP} = 4.9 and β_{LTD} = 8.2. Based on these measured nonlinearities, the decoder uses a device-informed 4-bit (16-state) conductance partition, and weight mapping assigns targets according to the β-dependent update characteristics.

Location of Change: Results – (page 8)

Figure 3(h) demonstrates vector–matrix multiplication (VMM) results obtained from the fabricated 20 × 20 NOR-type FTJ array by randomly programming cells to HRS/LRS and stepwise increasing the number of activated (LRS) devices under a 2 V read bias, showing that the column-summed current scales linearly with the activated-device count and thus confirming array-level VMM linearity in the deterministic regime. For decoder VMM we operate at a low read voltage of 2 V, 10 ms to stay in the RTN free regime and to reduce power and IR drop while preserving linear accumulation.

Location of Change: Results – (page 14)

Note that the RTN-based sampler is implemented in hardware (Fig. 5a,b), whereas the decoder VMM is realized in hardware-informed software. In this implementation, weights are mapped to 4-bit (16-state) conductance levels derived from the measured LTP/LTD nonlinearities (β_{LTP} = 4.9, β_{LTD} = 8.2; Fig. 3g), and array-level non-idealities are incorporated by applying a multiplicative noise factor with variance σ extracted from the 20 × 20 array measurements at 2 V (Fig. 3h).

4. According to the Supplementary Table, training on the CelebA dataset requires about 1,562 iterations per epoch and 10 epochs total, exceeding 10^5 cycles overall. How can the FTJ design accommodate such a large number of training iterations?

Response:

Thank you for your comment. As the reviewer pointed out, it is important to clarify how the FTJ devices accommodate the number of training updates used for CelebA. We address this in three parts.

First, the total number of weight updates in our training run is below 10^5. CelebA is trained for 10 epochs with about 1,562 iterations per epoch. This corresponds to about 15,620 optimizer steps in total. This count refers to parameter update steps and not to repeated hard reprogramming of every device across its full conductance window.

Second, Supplementary Fig. 3 shows that the devices remain functional up to 10^7 switching events and that the remnant polarization stays largely unchanged up to 10^5 cycles with only minor degradation. The total of about 15,620 optimizer steps is well within this endurance envelope.

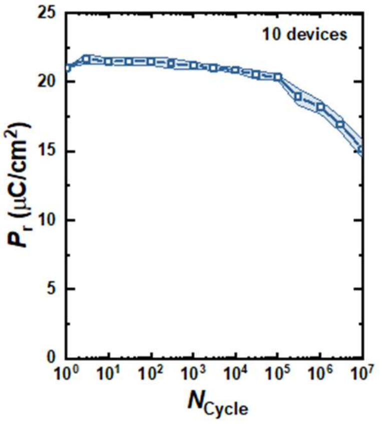

Supplementary Figure 3. Polarization endurance characteristics of the fabricated FTJ device. P_r values are normalized by the initial P_r ($P_{r,initial}$). One rectangular program pulse (5 V, 10 μs) followed by one erase pulse (−5 V, 10 μs) constitutes a single cycle (N_{cycle} = 1). The devices exhibit a slight initial increase in polarization, indicative of a wake-up phenomenon, followed by minor degradation beyond 10^5 cycles, demonstrating robust and stable endurance characteristics. The measurements were performed on 10 devices, and the standard deviation is represented by error bars (shaded in light blue).

Third, the 10^5 endurance shown in Supplementary Fig. 3 is a very harsh condition relative to neuromorphic learning. The protocol defines each cycle as a full program pulse followed by a full erase pulse, which forces a large polarization swing at every cycle. Neuromorphic training does not apply such full window reprogramming. Instead it uses small verify and adjust nudges that correspond to partial domain motion rather than full reversal. As a result the effective device stress per update is much smaller and many steps remain in the current quantization bin or move by only one bin. Fig. R20 corroborates this by showing trajectories of representative decoder weights over 10 epochs plotted as normalized weight versus epoch. The curves change gradually without abrupt jumps,

which indicates incremental updates with limited verify and adjust pulses per step and supports the partial switching interpretation.

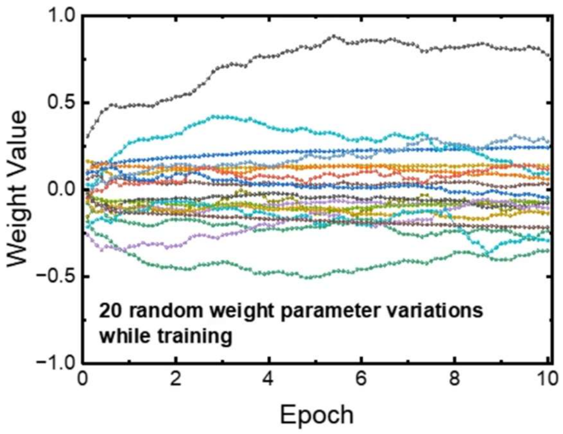

Fig. R20. Evolution of 20 randomly selected weights during CelebA training. Trajectories of 20 randomly chosen network weights over the first 10 epochs. The *x*-axis represents epoch and the *y*-axis shows normalized weight. Each curve corresponds to one parameter and exhibits very small, gradual changes per epoch with no abrupt jumps, indicating slow drift and minimal per-epoch reprogramming demand.

Overall, the actual update count is about 1.6×10^4, the devices sustain 10^5 level cycling with minor degradation even under harsh endurance conditions and remain functional to 10^7, and the incremental nature of learning imposes far milder stress than conventional endurance testing.

Furthermore, to identify why the FID saturates beyond 10^5 cycles, we performed additional measurements and analysis. We extracted cycle resolved LRS and HRS current distributions at $N_{\mathrm{cycle}} = 10^0$, 10^3, 10^5, and 10^6 and quantified σ_{I} over I_{avg} as a function of N_{cycle}. Fig. R10 and Fig. R11 show the histograms and Fig. R12 summarizes σ_{I} over I_{avg}. The HRS spread increases faster than LRS, which reduces the on off ratio and increases read errors during VMM accumulation. This mechanism explains the FID trend at high cycle counts.

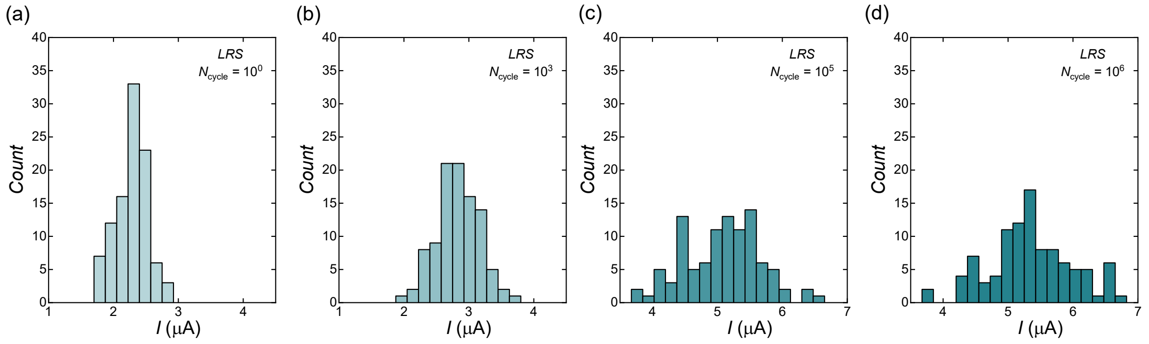

Fig. R10. Cycle-dependent current distribution in LRS. Histograms of cell current in LRS at (a) $N_{\mathrm{cycle}} = 10^0$, (b) $N_{\mathrm{cycle}} = 10^3$, (c) $N_{\mathrm{cycle}} = 10^5$, and (d) $N_{\mathrm{cycle}} = 10^6$.

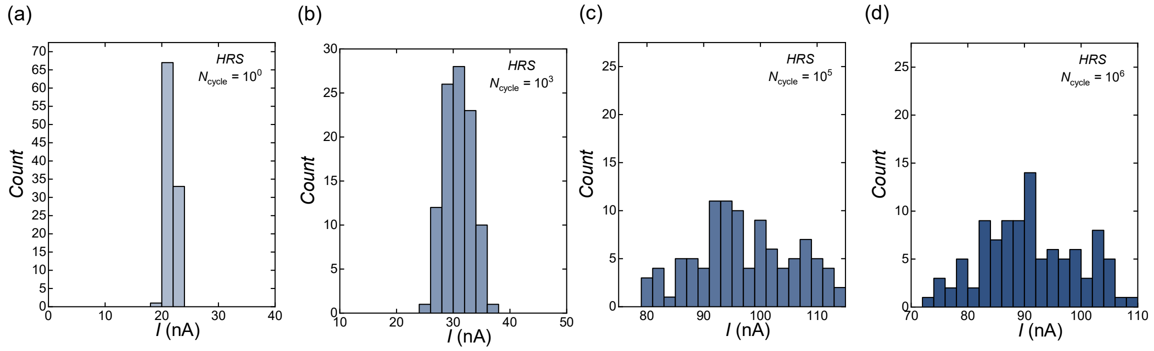

Fig. R11. Cycle-dependent current distribution in HRS. Histograms of cell current in LRS at (a) $N_{\mathrm{cycle}} = 10^0$, (b) $N_{\mathrm{cycle}} = 10^3$, (c) $N_{\mathrm{cycle}} = 10^5$, and (d) $N_{\mathrm{cycle}} = 10^6$.

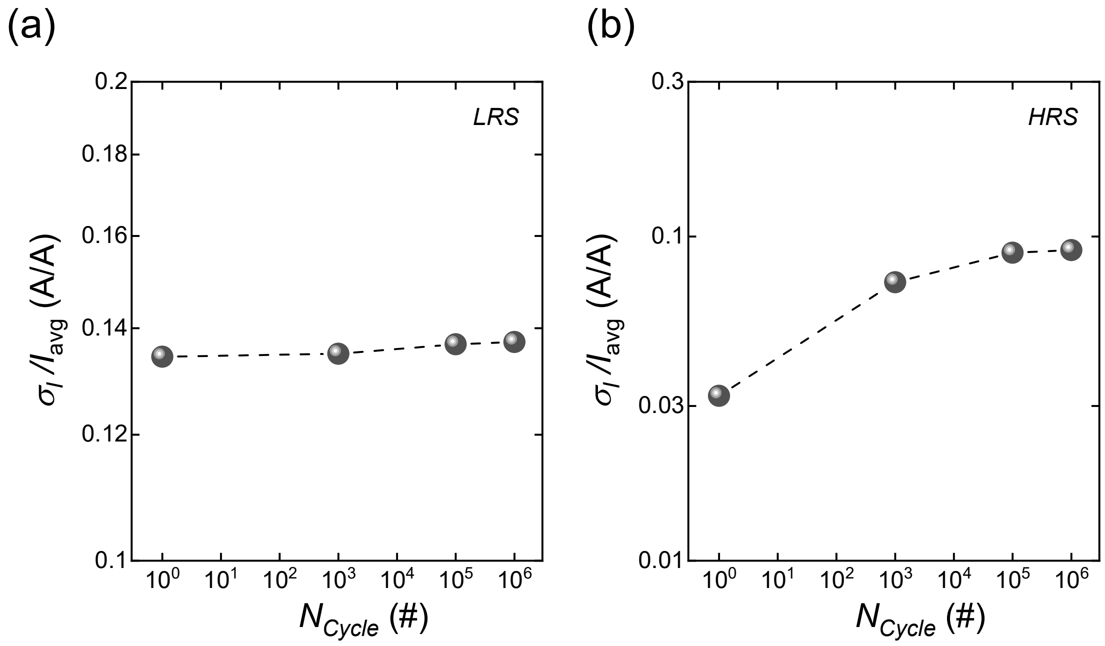

Fig. R12. Spread of current over cycling. (a) $\sigma_I/I_{\mathrm{avg}}$ versus N_{cycle} in LRS. (b) $\sigma_I/I_{\mathrm{avg}}$ versus N_{cycle} in HRS.

We have clarified these points and referenced Supplementary Fig. 3, Fig. R10, Fig. R11, Fig. R12 and Fig. R20 in the revised manuscript. Please refer to the revised manuscript for these updates.

Corresponding change in manuscript: Yes

Location of Change: Fig. 6(i)

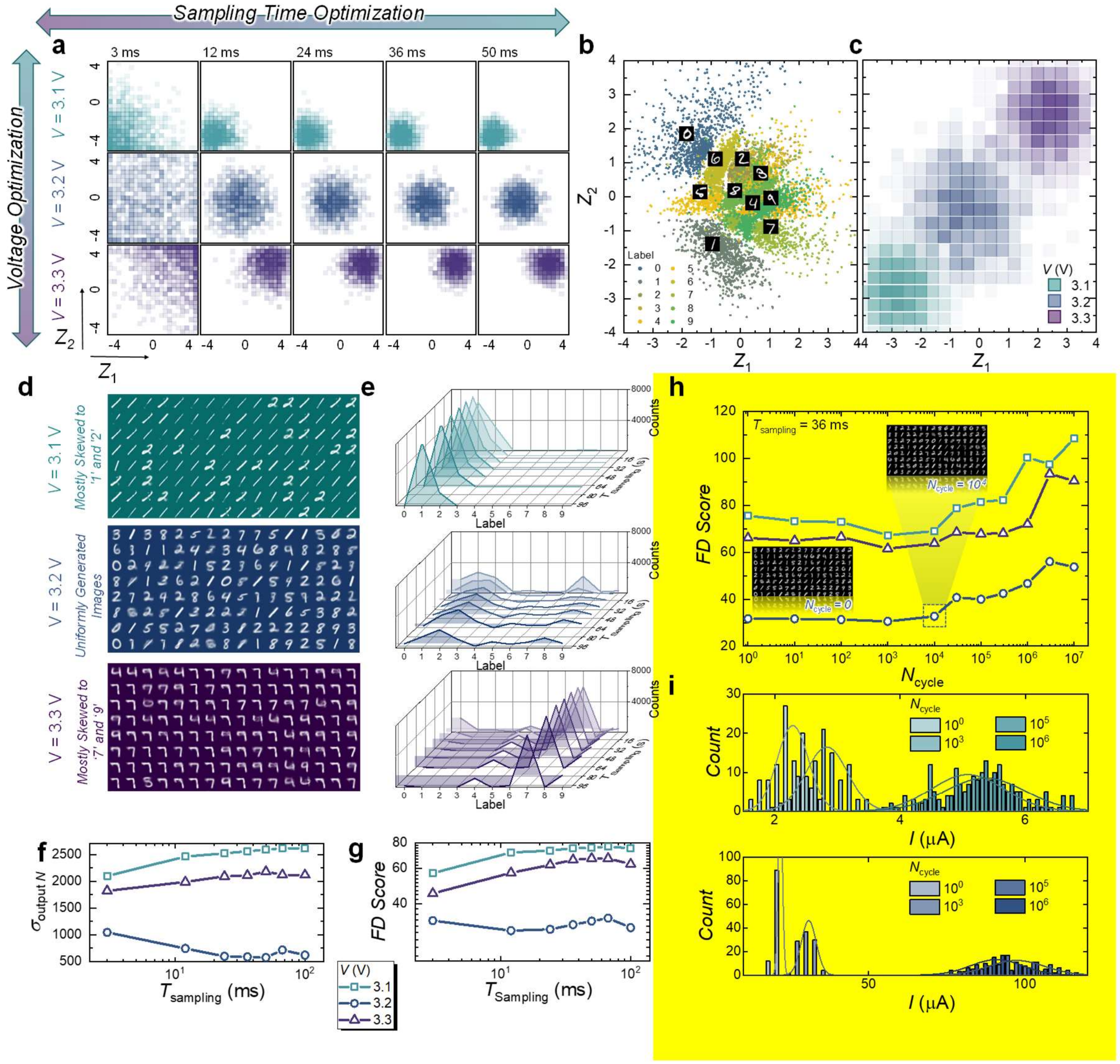

Figure 6. Evaluation of latent space distribution and image generation performance under varying voltage and sampling conditions. a Distribution of generated latent vectors for each V and $T_{Sampling}$, with biased distributions observed at 3.1 V and 3.3 V. **b** Latent space for the actual MNIST dataset, showing the ideal distribution for accurate image generation. **c** Distribution of random variables in the latent space generated by varying V at fixed $T_{Sampling}$ = 36 ms. **d** Generated MNIST images at different voltages. At V = 3.1 V, only digits "1" and "2" are generated, while V =3.2 V produces all digits evenly, and V =3.3 V is biased toward digits "7" and "9." **e** Distribution of generated images by label across different voltages and sampling conditions, with V = 3.2 V showing the most uniform distribution. **f** $\sigma_{output\ N}$ of generated images by label, with V = 3.2 V and $T_{Sampling}$ = 36 ms showing the lowest deviation. **g** FD score of generated images, with V = 3.2 V and $T_{Sampling}$ = 36 ms presenting the lowest FD score, indicating the highest image diversity. **h** FD score versus N_{cycle}s, confirming robust FTJ operation in the absence of RTN. **i** Cycle-dependent current distribution in HRS (up) and LRS (down). Histograms of cell current in LRS at (a) $N_{cycle} = 10^0$, (b) $N_{cycle} = 10^3$, (c) $N_{cycle} = 10^5$, and (d) $N_{cycle} = 10^6$.

Location of Change: Results – (page 19)

Figure 6(h) presents the FD score as a function of N_{cycle}, demonstrating that stable image generation is maintained even up to 10^5 generation cycles. Figure 6(i) further shows the cycle-dependent current distributions in HRS (top) and LRS (bottom): histograms at (a) $N_{cycle}=10^0$, (b) 10^3, (c) 10^5, and (d) 10^6. The HRS peak broadens and drifts more strongly than LRS, increasing their overlap and effectively reducing the on/off ratio, which correlates with the slight FD increase at high cycle counts. Effects of RTN operating region and write-cycle count on MNIST generation are shown in Supplementary Fig. 13. This result confirms the robustness and reliability of the system under repeated operation.

5. The latency and power of the random-sampling process could be compared with prior art, such as CMOS-based or ReRAM-based random number generators/sampling process.

Response:

Thank you for your comment. To focus the comparison on latency and power in random sampling, we selected more appropriate prior works (CMOS- and ReRAM-based) and compiled them in Table 1. The per-sample energy is the sum of the FTJ read current term and the sense-amplifier/flip-flop overhead. For the FTJ term alone, 3.2 V×20 nA×36 ms = 2.3 nJ per random sample. On a per-image basis this is 2.3 nJ × 16 (bitstreams) × 256 (latent dimension) = 9.4 μJ (282 μW). The sampling latency is 36 ms, as discussed in the main text. We have extended Table 1 with this quantitative comparison.

Corresponding change in manuscript: Yes

Location of Change: Table 1

									Latent Sampling		Decoder Mapping
**Work**	**Latent Sampling**	**Decoder Mapping**	**Device type**	**CMOS compatibility**	**Array Size**	**Task**	**Implementation**	**Cell Area**	**Speed***	**Power****	**TOPS/W**
This work	O	O	Both FTJ	O	400	MNIST, CelebA	Circuit-level	2 × 2 μm²	36 ms	282 μW (FTJ-only)	106 (FTJ & ADC)
[5]	O	-	Bi_2O_2Se	X	1 (Single Device)	MNIST	Device-level	10 × 10 - 40 × 40 μm²	10 ms	98.1-337 μW	-
[6]	O	-	ReRAM	X	1 (Single Device)	TRNG-only	Circuit-level	5 × 5 - 50 × 50 μm²	50-250 ms	1.23- 862 μW	-
[7]	O	-	ReRAM	X	1 (Single Device)	TRNG-only	Circuit-level	2.5 × 2.5 μm²	100 ms	1.47 mW	-
[8]	-	O	ReRAM	O	Only Simulation	MNIST	Simulation-level	-	-	-	4.6
[9]	-	O	1T-1R	O	1000	MNIST	Circuit-level	-	-	-	-
[10]	O	-	CMOS	O	-	TRNG	Circuit-level	-	11.6-312 ns	38.9-91.6 mW	
[11]	-	O	CMOS	O	-	Neural Networks	Commercial	-	-	-	2.3

*Sampling time per bit
**Normalized for 30 fps Human Face Image (128 ×128) Generation
Tera operations per second (TOPS)

Table 1. Comparison of our work with previous reported hardware generative models.

Location of Change: Results – (page 2)

As a result, prior efforts have largely depended on hybrid architectures or off-chip software-based stochastic modules, constraining system scalability, energy efficiency, and integration density [5-11]. Moreover, in mature CMOS-based technologies, separate studies have been proposed to perform each role, implementing stochastic functionality [8] and handling deterministic models [11,12].

Location of Change: Results – (page 20)

Supplementary Note 7 presents throughput, array requirements, and energy overhead for RTN sampling, VMM

operation, and ADCs, evaluated at 30 fps on CelebA.

Location of Change: Results – (page 21)

FTJ-based platform is also quantitatively benchmarked against the mature CMOS-based designs in area, power per sample, and latency (Table 1). For VMM specifically, our FTJ arrays achieve <10 fJ/MAC, over 40× lower than the ~430 fJ/MAC reported for 8-bit MACs on a widely deployed TPU [11], and sustain ~1.31 TOPS per 256×256 array at 10 MHz, with throughput scaling through array replication.

Location of Change: Supplementary Note 7

Supplementary Note 7. Throughput, array, and energy overhead in terms of RTN Sampling, VMM operation, and ADCs (30 fps, CelebA)

RTN sampling: To sustain 30 fps, a 256-dimensional latent vector must be sampled 30 times per second. Our study uses bitstream sampling with ≥16 streams, so real-time operation requires 256×30×16=122,880 samplings. Assuming a 36 ms sampling window per FTJ, a single device can provide ~27 samples. Thus ~4,552 FTJs are needed solely for RTN sampling. As software optimization is not our focus, this number can be reduced in practice by lowering the latent dimension or the number of bitstreams.

VMM operation and minimum arraying: We assume multiple arrays, since a single 2-D array cannot cover the full VAE decoder at once. In our decoder, one 128 × 128 image generation requires about 3.57 Giga-MAC. At 30 fps this is ~100 GOPS (≈ 0.1 TOPS). With 256 × 256 FTJ arrays and differential weights (two devices per parameter for positive/negative value representation), the minimum array count is 413 (256 for fully connected layers and 157 for the transposed convolutional layers) [5]. A 256 × 256 array performs 131,072 operations (MACs) per cycle (counting multiply and add separately as 256 × 256 × 2). Even at a modest 10 MHz operation of the macro, it yields ~1.31 TOPS per array. The different layers demand different workloads (especially convolution). For instance, the last layer with 128 × 128 kernel slides, which is the slowest layer, still meets real-time targets, at 10 MHz the per-inference time is ~1.64 ms, so additional replicated arrays for parallelism are not required.

ADC overhead and read energy: The total ADC energy equals the number of ADC activations per inference counts the sampling energy. For our network, the ADC operates 23,396,352 times per inference. Assuming 1 pJ/sample [6], this is ~23 µJ total, or ~6.44 fJ per MAC. The read-path energy from synaptic currents, in a worst-case estimate (read time 100 ns, LRS current 2.6 µA, read voltage 2 V, and 3.57×10^9 operations with all weights and inputs at 1), is ~1.85 mJ per inference (~600 fJ/MAC). In a more realistic setting, shorter read time (5 ns) [7] and average current ~0.1× LRS from training statistics, the read energy drops to ~9 µJ per inference (~3 fJ/MAC), which is lower than the ADC contribution.

[R1] X. Peng, R. Liu, and S. Yu, "Optimizing Weight Mapping and Data Flow for Convolutional Neural Networks on Processing-in-Memory Architectures", IEEE Transactions on Circuits and Systems-I: Regular Papers, 67, 4, (2020)

[R2] H. Jiang, W. Li, S. Huang, and S. Yu, “Analog-to-Digital Converter Design Exploration for Compute-in-Memory Accelerators”, IEEE Design and Test, 39, 2, 48-55 (2022).

[R3] P. Chen, X. Peng, and S. Yu, “DNN+ NeuroSim: An end-to-end benchmarking framework for compute-in-memory accelerators with versatile device technologies”, 2017 IEEE International Electron Devices Meeting (IEDM), (2019)

6. The reference [6] has a minor error in the publication venue.

Response:

Thank you for your comment. We have corrected the publication venue in the revised manuscript.

Please refer to the revised manuscript to find the modification.

Corresponding change in manuscript: Yes

Location of Change: Reference

[6] Chen, F., Song, L. & Chen, Y. ReGAN: A pipelined ReRAM-based accelerator for generative adversarial networks. ASP-DAC, 178–183 (2018).

Reviewer #3

I co-reviewed this manuscript with one of the reviewers who provided the listed reports. This is part of the Nature Communications initiative to facilitate training in peer review and to provide appropriate recognition for Early Career Researchers who co-review manuscripts.

Response:

We, the authors, are very much thankful for the detailed comments and instructive suggestions. We have revised the manuscript in light of the useful comments and suggestions of the reviewer. Each comment and suggestion has been followed and addressed in the response attached.

We, the authors, send the deepest appreciation for the encouragement and comments again.

Reviewer #4

Review of NCOMMS-25-67053-T "CMOS-Compatible FTJ Hardware Unifying Stochastic Sampling and Deterministic Computing for On-Chip Image Generation"

Summary

The manuscript reports a CMOS-compatible ferroelectric tunnel junction (FTJ) platform that unifies stochastic sampling and deterministic vector–matrix multiplication (VMM) within the same device type. By exploiting voltage-controlled random telegraph noise (RTN) at high current for stochastic latent vector generation, and multilevel ferroelectric conductance at low current for deterministic VMM, the authors design a hardware-native variational autoencoder (VAE). They demonstrate wafer-scale fabrication of 20×20 FTJ arrays, extensive device-level characterization, circuit-level random bitstream generation, and application-level validation on MNIST and CelebA image synthesis tasks.

This is a wide-ranging work spanning materials, device physics, circuits, and system demonstrations. It addresses a challenge in hardware generative AI, viz. reconciling the opposing requirements of stochastic sampling and deterministic decoding in a scalable hardware platform.

Response:

We, the authors, are very much thankful for the detailed comments and instructive suggestions. We have revised the manuscript in light of the useful comments and suggestions of the reviewer. Each comment and suggestion has been followed and addressed in the response attached.

We, the authors, send the deepest appreciation for the encouragement and comments again.

1. Scope of innovation and positioning

o The core novelty is apparent, yet the manuscript should articulate more distinctly how it advances beyond prior demonstrations of memristor-based stochastic sampling and in-memory generative models (e.g., Song Nat. Commun. 2025; Ding Commun. Mater. 2025; Lin Nat. Mach. Intell. 2023; Wang Sci. Adv. 2025).

o Table 1 mixes specific materials and categories; it should be reorganized by device types, with consistent terminology (ReRAM/RRAM, FTJ) and comparisons that explicitly cover stochastic sampling mechanisms, noise utilization stages, and integration strategies.

Response:

Thank you for your comment. As the reviewer pointed out, it is important to clarify how this work advances beyond previous demonstrations of memristor based stochastic sampling and in memory generative models and to improve the organization of Table 1.

First, we clarified how our contribution advances beyond Song Nat. Commun. 2025, Ding Commun. Mater. 2025, Lin Nat. Mach. Intell. 2023, and Wang Sci. Adv. 2025. These studies either leverage noise to realize continuous probabilistic weights for classification or survey noise as a functional resource, or they focus on deterministic in memory acceleration without on chip latent sampling. They do not combine voltage controlled physical sampling and deterministic decoding within a single CMOS compatible platform. Our work demonstrates on chip latent vector sampling using RTN together with on array VMM decoding and therefore addresses the co implementation of stochasticity and determinism required by generation rather than classification. This distinction and the integration scope are now stated explicitly in the Introduction.

Second, we reorganized Table 1 as requested. The table is now grouped by device type with consistent terminology for ReRAM or RRAM, FTJ, PCM, and transistor based implementations. For each work we added columns that report the stochastic mechanism, the stage where noise is utilized, and the integration strategy. The stochastic mechanism column distinguishes RTN, $1/f$ noise, and engineered continuous distributions. The noise utilization stage column specifies whether noise is used for latent sampling, weight perturbation, or training regularization. The integration strategy column indicates whether sampling and decoding occur in the same array, in separate arrays, or with off chip components. We also extended the table with area and energy metrics when available and moved the expanded quantitative comparison to Table R4 in the revision set. With this organization the reader can see at a glance that our FTJ platform is the only entry that uses a single array to provide both bias tunable stochastic sampling and deterministic VMM decoding for image generation.

We have revised the Introduction accordingly and updated Table to reflect these changes. Please refer to the revised manuscript for the updated positioning and comparisons.

Corresponding change in manuscript: Yes

Location of Change: Table 1

									Latent Sampling		Decoder Mapping
**Work**	**Latent Sampling**	**Decoder Mapping**	**Device type**	**CMOS compatibility**	**Array Size**	**Task**	**Implementation**	**Cell Area**	**Speed***	**Power****	**TOPS/W**
This work	O	O	Both FTJ	O	400	MNIST, CelebA	Circuit-level	2 × 2 μm²	36 ms	282 μW (FTJ-only)	106 (FTJ & ADC)
[5]	O	-	Bi_2O_2Se	X	1 (Single Device)	MNIST	Device-level	10 × 10 - 40 × 40 μm²	10 ms	98.1-337 μW	-
[6]	O	-	ReRAM	X	1 (Single Device)	TRNG-only	Circuit-level	5 × 5 - 50 × 50 μm²	50-250 ms	1.23- 862 μW	-
[7]	O	-	ReRAM	X	1 (Single Device)	TRNG-only	Circuit-level	2.5 × 2.5 μm²	100 ms	1.47 mW	-
[8]	-	O	ReRAM	O	Only Simulation	MNIST	Simulation-level	-	-	-	4.6
[9]	-	O	1T-1R	O	1000	MNIST	Circuit-level	-	-	-	-
[10]	O	-	CMOS	O	-	TRNG	Circuit-level	-	11.6-312 ns	38.9-91.6 mW	
[11]	-	O	CMOS	O	-	Neural Networks	Commercial	-	-	-	2.3

*Sampling time per bit

**Normalized for 30 fps Human Face Image (128 ×128) Generation

Tera operations per second (TOPS)

Table 1. Comparison of our work with previous reported hardware generative models.

Location of Change: Introduction – (page 2)

As a result, prior efforts have largely depended on hybrid architectures or off-chip software-based stochastic modules, constraining system scalability, energy efficiency, and integration density [5-11]. Moreover, in mature CMOS-based technologies, separate studies have been proposed to perform each role, implementing stochastic functionality [8] and handling deterministic models [11,12].

Location of Change: Introduction – (page 3)

While recent studies leverage device noise to realize continuous weight distributions for classification, our work focuses on hardware-native generation by using RTN for latent sampling and FTJ-based VMM for decoding within a single array [21,22].

Location of Change: Results – (page 20)

Supplementary Note 7 presents throughput, array requirements, and energy overhead for RTN sampling, VMM operation, and ADCs, evaluated at 30 fps on CelebA.

Location of Change: Results – (page 21)

FTJ-based platform is also quantitatively benchmarked against the mature CMOS-based designs in area, power per sample, and latency (Table 1). For VMM specifically, our FTJ arrays achieve <10 fJ/MAC, over 40× lower than the ~430 fJ/MAC reported for 8-bit MACs on a widely deployed TPU [11], and sustain ~1.31 TOPS per 256×256 array at 10 MHz, with throughput scaling through array replication.

2. Use of FTJ cells for stochastic vs deterministic modes

o It remains unclear whether the same physical cells can actively toggle between stochastic and deterministic roles during one inference, or whether subsets of the array are fixed/dedicated to each function. This distinction matters because reprogramming trained weights repeatedly may not be feasible or cost-effective at all. Please clarify the system-level usage strategy and (re)programming accuracy, complexity and overhead.

Response:

Thank you for your comment. As the reviewer pointed out, it is important to clarify how the same FTJ array supports both stochastic and deterministic modes during inference and whether repeated reprogramming of trained weights is required.

Our architecture does not require reprogramming of trained weights for each inference. The array adopts a role partitioning strategy rather than dynamic retuning. A small fixed portion of the array operates in the stochastic mode to generate latent vectors through voltage controlled RTN, while the remaining majority of the array performs deterministic VMM decoding with the pre trained weights. Both regions use the same FTJ cell structure, allowing full co integration on the same chip or wafer without additional fabrication steps. Trained weights are programmed once and remain fixed during inference. This is the novelty of our approach. Prior systems either do not implement both functions or, when both are present, they rely on different device types and require chip to chip interconnection. In contrast, our design provides both stochastic sampling and deterministic decoding within one FTJ array and one process stack.

This integration is enabled by the bias dependent noise regime of the same device. At a low read voltage around 2 V the FTJ exhibits $1/f$ noise with short correlation times. Temporal averaging during VMM suppresses these fluctuations and enables stable deterministic accumulation. When the bias is increased to the mid bias window around 3.1 to 3.3 V the same device enters the RTN regime, where discrete trapping and detrapping events produce stochastic binary transitions that are suitable for latent sampling. These two behaviors, $1/f$ for deterministic computation and RTN for stochastic sampling, arise from the same device physics and are separated only by voltage.

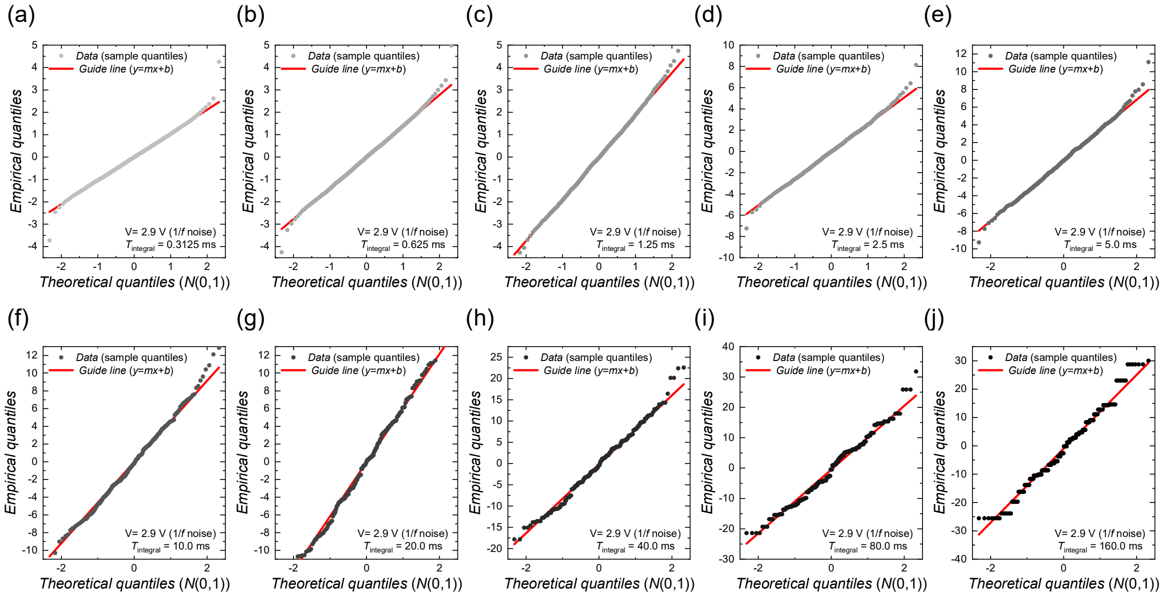

Fig. R16. QQ plots of the standardized block sums $Z_N = (S_N - \mu_N)/(\sigma N^{1/2})$ for a 1/*f* noise signal ($V = 2.9$ V). The *x*-axis shows theoretical quantiles of *N*(0,1), and the *y*-axis shows empirical quantiles of Z_N. Dots shows the empirical quantiles; the red line is a data-based guideline, $y = mx + b$. Panels correspond to the different integration times: (a) 0.3125 ms, (b) 0.625 ms, (c) 1.25 ms, (d) 2.50 ms, (e) 5.0 ms, (f) 10.0 ms, (g) 20.0 ms, (h) 40.0 ms, (i) 80.0 ms, (j) 160.0 ms). The 1/*f* noise aligns with the 45-degree line even at short integration time, indicating rapid convergence to normality.

To verify this behavior, we used quantile-quantile (Q-Q) plots to compare the empirical distribution of integrated signals with a theoretical Gaussian. For each integration window, consecutive current samples were grouped into blocks of N samples, summed to form S_N, standardized as $Z_N = (S_N - \mu_N)/(\sigma_N{}^{1/2})$, and their empirical quantiles were plotted on the *y*-axis against the theoretical quantiles of a standard normal distribution on the *x*-axis.

Fig. R16 shows the 1/*f* regime at $V = 2.9$ V, where the data points align with the 45-degree reference line even at short integration windows. This confirms that the integrated current follows a Gaussian distribution, consistent with the CLT, and that 1/*f* fluctuations are effectively averaged out during VMM. Fig. R17, Fig. R18, and Fig. R19 show the RTN regime at $V = 3.1$ V, 3.2 V, and 3.3 V. In these plots, the Q–Q curves exhibit persistent *S*-shaped curvature and heavy tails across all integration times, confirming that RTN maintains long-range correlation and does not converge to Gaussian statistics even under prolonged averaging.

These results demonstrate that deterministic VMM can reliably operate in the 1/*f* regime, where integration naturally suppresses fluctuations, whereas the RTN ($1/f^2$) regime retains its non-Gaussian statistics and is therefore reserved for stochastic sampling. Following the reviewer's suggestion, we have added this CLT based explanation, a description of the Q–Q analysis method, and the role partitioning policy with references to Fig. R16 to Fig. R19 in the revised manuscript. Please refer to the revised manuscript for these updates.

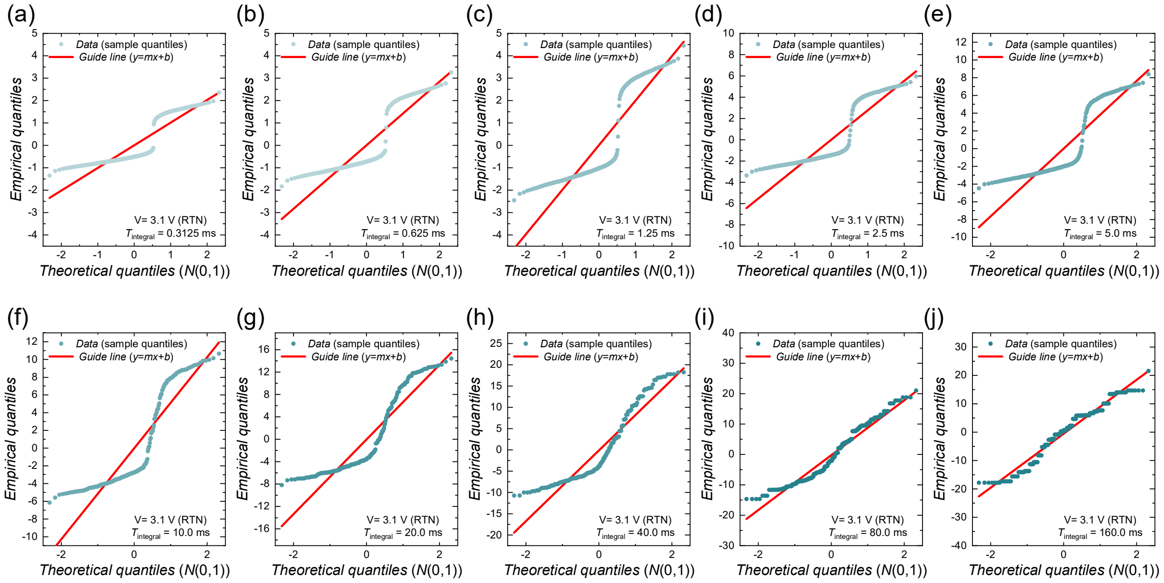

Fig. R17. QQ plots of the standardized block sums $Z_N = (S_N - \mu_N)/(\sigma N^{1/2})$ for an RTN signal ($V = 3.1$ V). The x-axis shows theoretical quantiles of $N(0,1)$, and the y-axis shows empirical quantiles of Z_N. Dots show the empirical quantiles; the red line is a data-based guideline, $y = mx + b$. Panels correspond to the different integration times: (a) 0.3125 ms, (b) 0.625 ms, (c) 1.25 ms, (d) 2.50 ms, (e) 5.0 ms, (f) 10.0 ms, (g) 20.0 ms, (h) 40.0 ms, (i) 80.0 ms, (j) 160.0 ms). The RTN signal retains an S-shaped curvature and heavy tails due to long-range correlation.

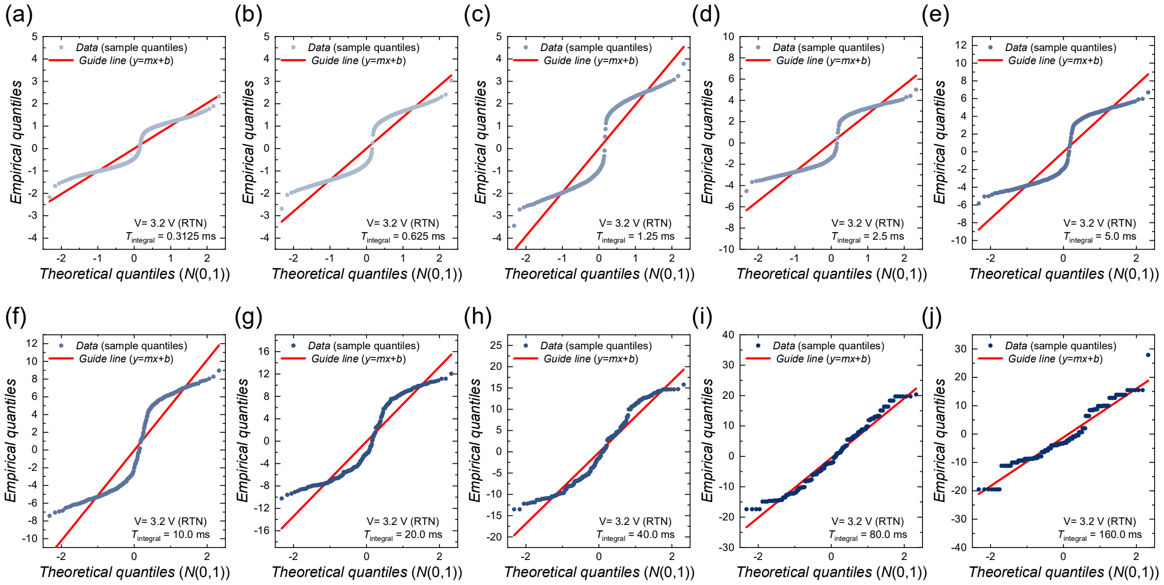

Fig. R18. QQ plots of the standardized block sums $Z_N = (S_N - \mu_N)/(\sigma N^{1/2})$ for an RTN signal ($V = 3.2$ V). The x-axis shows theoretical quantiles of $N(0,1)$, and the y-axis shows empirical quantiles of Z_N. Dots show the

empirical quantiles; the red line is a data-based guideline, $y = mx + b$. Panels correspond to the different integration times: (a) 0.3125 ms, (b) 0.625 ms, (c) 1.25 ms, (d) 2.50 ms, (e) 5.0 ms, (f) 10.0 ms, (g) 20.0 ms, (h) 40.0 ms, (i) 80.0 ms, (j) 160.0 ms). The RTN signal retains an S-shaped curvature and heavy tails due to long-range correlation.

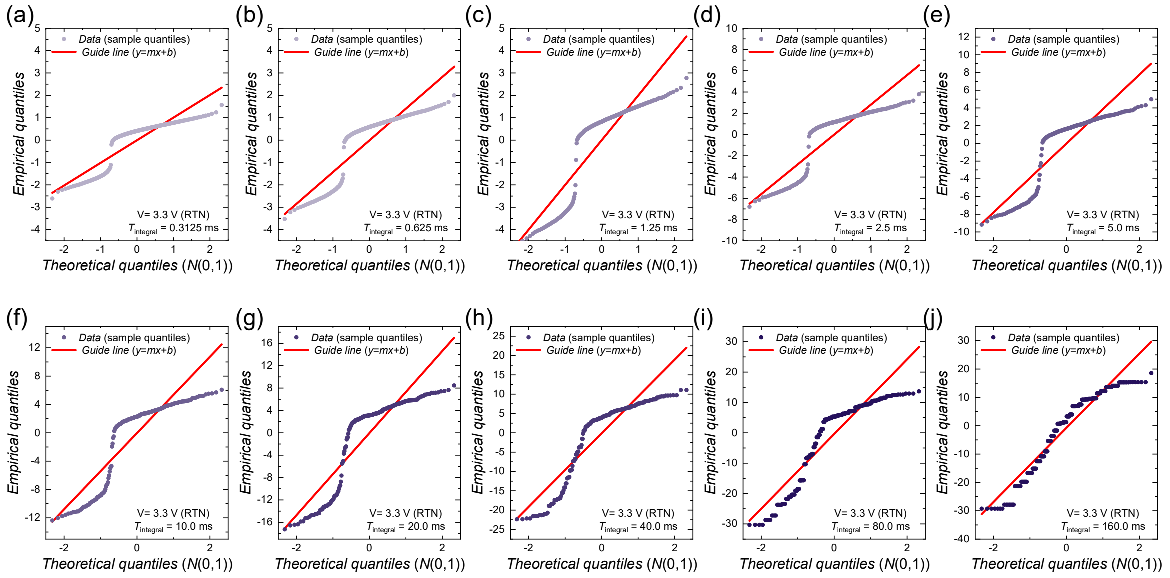

Fig. R19. QQ plots of the standardized block sums $Z_N = (S_N - \mu_N)/(\sigma N^{1/2})$ for an RTN signal (V = 3.3 V). The x-axis shows theoretical quantiles of $N(0,1)$, and the y-axis shows empirical quantiles of Z_N. Dots show the empirical quantiles; the red line is a data-based guideline, $y = mx + b$. Panels correspond to the different integration times: (a) 0.3125 ms, (b) 0.625 ms, (c) 1.25 ms, (d) 2.50 ms, (e) 5.0 ms, (f) 10.0 ms, (g) 20.0 ms, (h) 40.0 ms, (i) 80.0 ms, (j) 160.0 ms). The RTN signal retains an S-shaped curvature and heavy tails due to long-range correlation.

Corresponding change in manuscript: Yes

Location of Change: Result – (page 13)

The ability to control RTN behavior through voltage adjustments provides an effective mechanism for neural network-based image generation systems. Firstly, by modulating the applied voltage, FTJs not only enable the generation of RTN but also allow for its suppression, enabling seamless transitions between stochastic and deterministic operations. We further note that deterministic VMM is reliable in the $1/f$ regime, whereas $1/f^2$ RTN is not suitable for VMM and is used for stochastic sampling. The central limit theorem rationale and the Q–Q analysis supporting this distinction are provided in Supplementary Fig. 17, 18, 19, 20 and Supplementary Note 1.

Location of Change: Supplementary Note 1

Supplementary Note 1. Central limit behavior and Q–Q analysis for $1/f$ and RTN regimes

We analyze the statistics of time integrated read current using a quantile–quantile framework and we connect the observed behavior to the applicability of the central limit theorem (CLT). The method is as follows. Consecutive current samples are grouped into non overlapping blocks of N points and summed to form S_N. The sums are standardized as $Z_N = (S_N - \mu_N)/(\sigma_N^{1/2}))$ where μ_N and σ_N are the empirical mean and standard deviation of S_N. We then plot the empirical quantiles of Z_N against the theoretical quantiles of a standard normal distribution. If the integrated signal is effectively Gaussian, the Q–Q points follow a straight line with unit slope. Deviations from linearity reveal non Gaussian tails or correlation that violates the independence assumption required for the central limit theorem.

Figure R17 shows the Q–Q plots for the $1/f$ regime at $V = 2.9$ V. The integration window is swept from 0.3125 ms to 160 ms. The points align closely with the 45 degree reference over all windows, which indicates rapid convergence toward a normal distribution under time integration. This behavior supports reliable deterministic accumulation during VMM in the $1/f$ regime.

Figures R18, R19, and R20 show the RTN regime at $V = 3.1$ V, $V = 3.2$ V, and $V = 3.3$ V. Each figure sweeps the same set of integration windows. The Q–Q curves display persistent S shaped curvature with heavy tails that remain even as the window increases. The curvature indicates that long range temporal correlation survives the integration and that successive samples are not independent. As a result Z_N does not converge to a standard normal distribution within practical windows. This behavior is consistent with a two level process whose dwell times are set by τ_L and τ_H and whose transitions retain memory over many samples.

Taken together these results explain the operating policy used in the manuscript. In the $1/f$ regime the time integration inherent to VMM averages out short range fluctuations so the output approaches its mean and deterministic accumulation is reliable. In the RTN regime the long correlation prevents convergence to a Gaussian distribution within practical windows, so the integrated output retains stochasticity that is useful for latent sampling but not for precise multiplication. For this reason we operate the decoder VMM at low read voltage in the RTN free region (V= 2.0 V) and we use bias near 3.2 V to obtain balanced τ_L and τ_H for stochastic sampling.

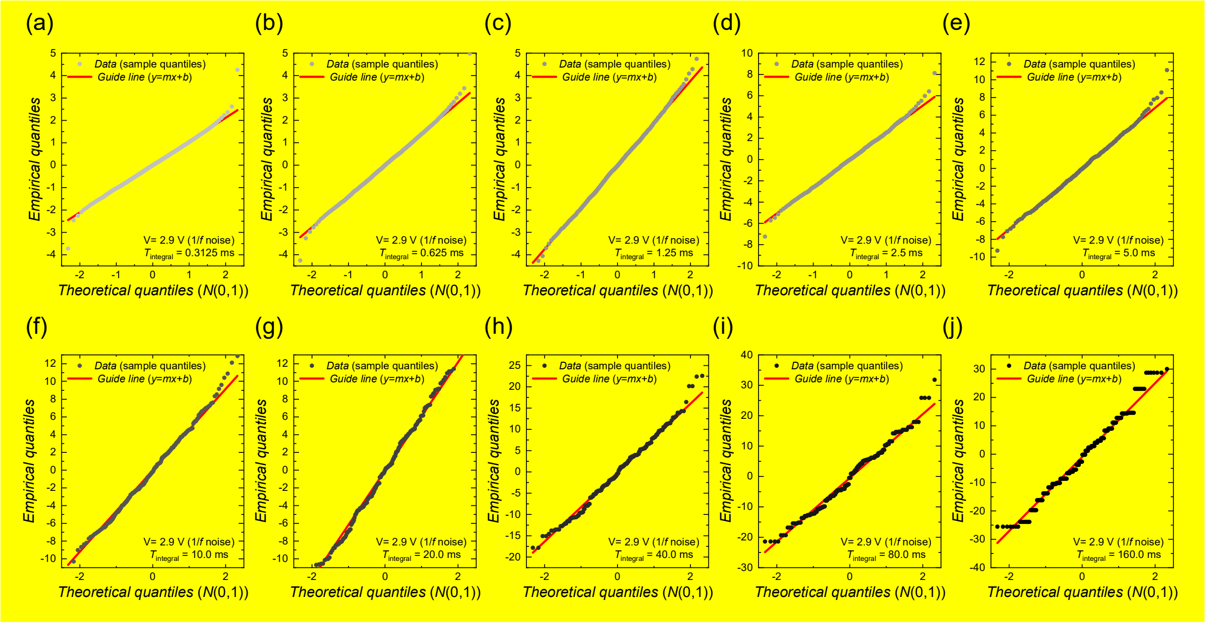

Supplementary Figure 17. Q-Q plots of the standardized block sums $Z_N = (S_N - \mu_N)/(\sigma_N^{1/2})$ for a 1/*f* noise signal (V = 2.9 V). The *x*-axis shows theoretical quantiles of *N*(0,1), and the *y*-axis shows empirical quantiles of Z_N. Dots shows the empirical quantiles; the red line is a data-based guideline, $y = mx + b$. Panels correspond to the different integration times: (a) 0.3125 ms, (b) 0.625 ms, (c) 1.25 ms, (d) 2.50 ms, (e) 5.0 ms, (f) 10.0 ms, (g) 20.0 ms, (h) 40.0 ms, (i) 80.0 ms, (j) 160.0 ms). The 1/*f* noise aligns with the 45-degree line even at short integration time, indicating rapid convergence to normality.

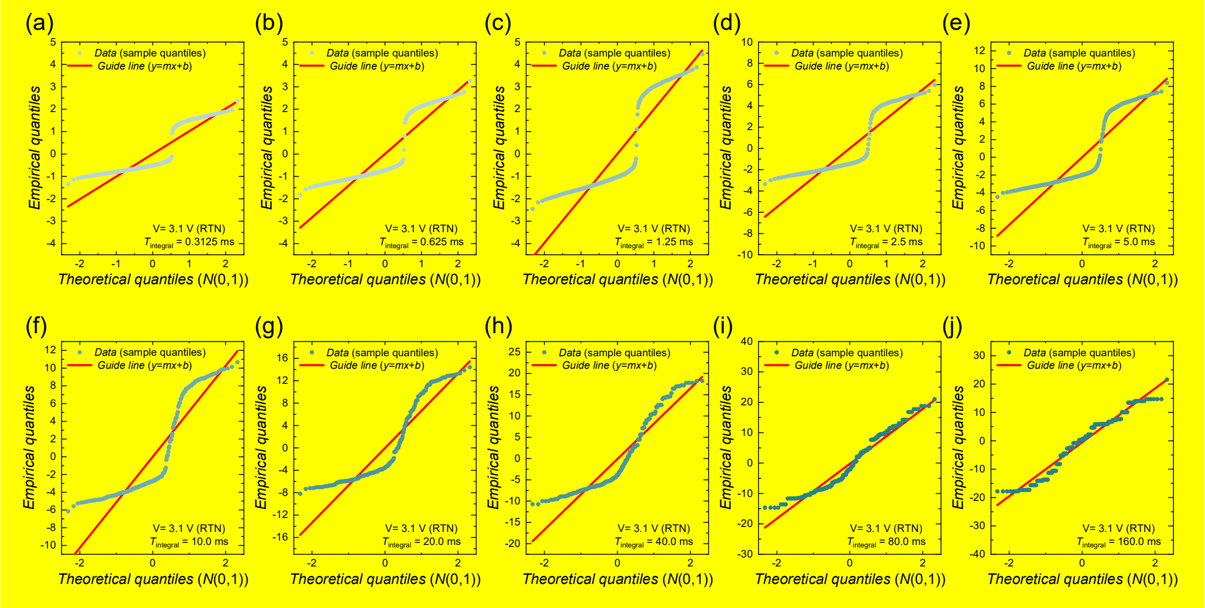

Supplementary Figure 18. QQ plots of the standardized block sums $Z_N = (S_N - \mu_N)/(\sigma_N^{1/2})$ for an RTN signal (V = 3.1 V). The *x*-axis shows theoretical quantiles of *N*(0,1), and the *y*-axis shows empirical quantiles of Z_N. Dots show the empirical quantiles; the red line is a data-based guideline, $y = mx + b$. Panels correspond to the different integration times: (a) 0.3125 ms, (b) 0.625 ms, (c) 1.25 ms, (d) 2.50 ms, (e) 5.0 ms, (f) 10.0 ms,

(g) 20.0 ms, (h) 40.0 ms, (i) 80.0 ms, (j) 160.0 ms). The RTN signal retains an S-shaped curvature and heavy tails due to long-range correlation.

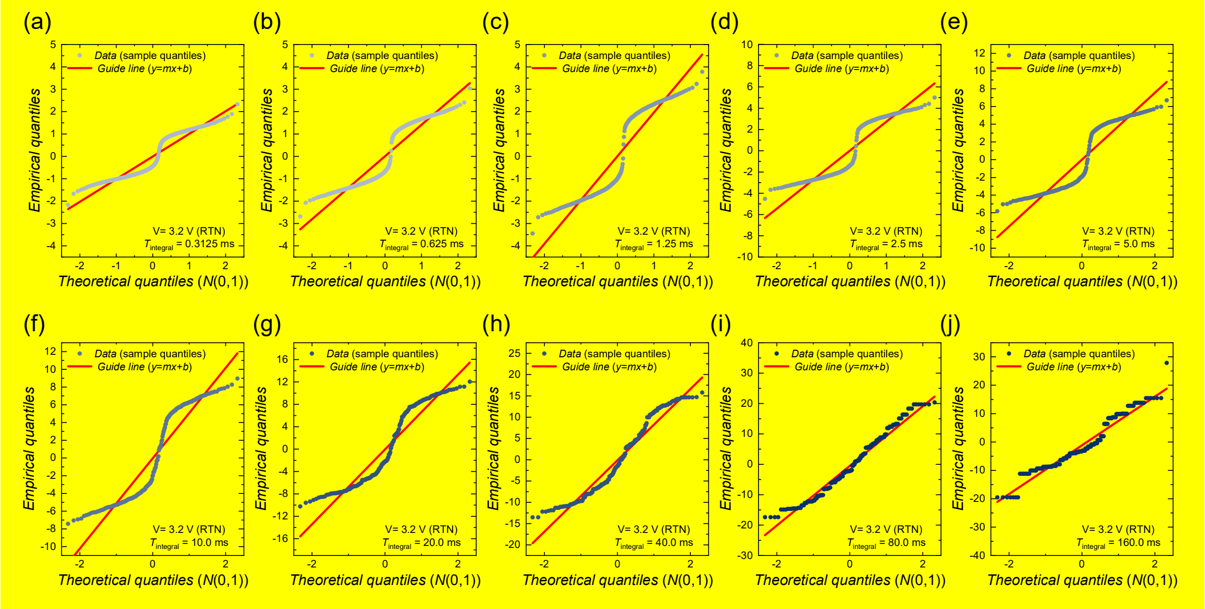

Supplementary Figure 19. QQ plots of the standardized block sums $Z_N = (S_N - \mu_N)/(\sigma_N^{1/2})$) for an RTN signal ($V$ = 3.2 V). The x-axis shows theoretical quantiles of $N(0,1)$, and the y-axis shows empirical quantiles of Z_N. Dots show the empirical quantiles; the red line is a data-based guideline, $y = mx + b$. Panels correspond to the different integration times: (a) 0.3125 ms, (b) 0.625 ms, (c) 1.25 ms, (d) 2.50 ms, (e) 5.0 ms, (f) 10.0 ms, (g) 20.0 ms, (h) 40.0 ms, (i) 80.0 ms, (j) 160.0 ms). The RTN signal retains an S-shaped curvature and heavy tails due to long-range correlation.

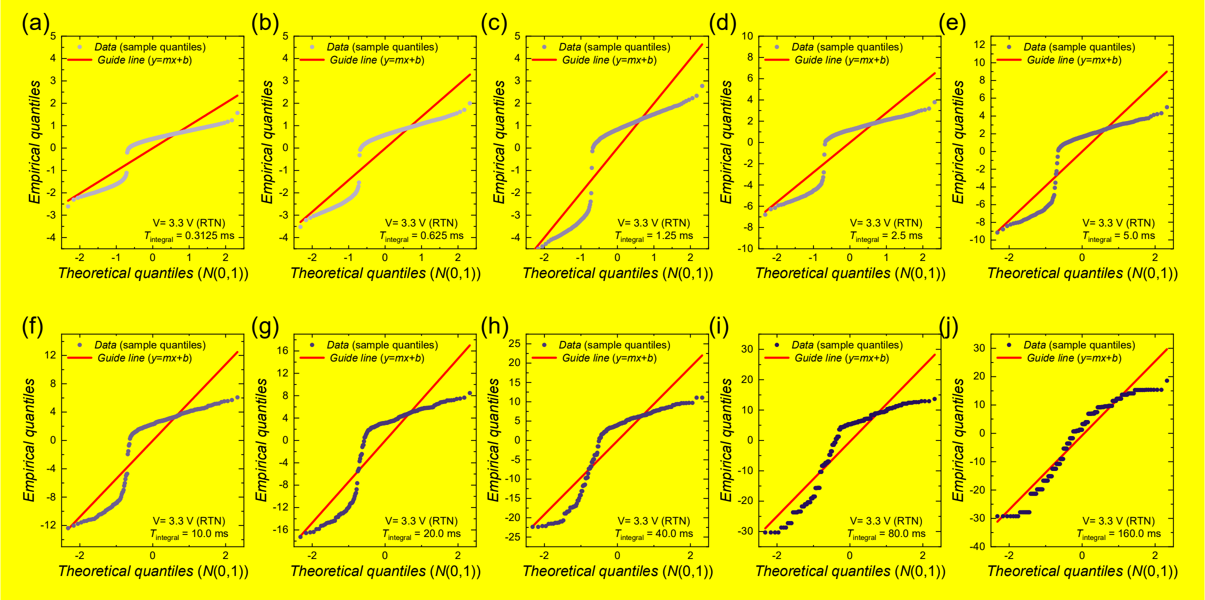

Supplementary Figure 20. QQ plots of the standardized block sums $Z_N = (S_N - \mu_N)/(\sigma N^{1/2})$) for an RTN signal ($V$ = 3.3 V). The x-axis shows theoretical quantiles of $N(0,1)$, and the y-axis shows empirical quantiles of Z_N. Dots show the empirical quantiles; the red line is a data-based guideline, $y = mx + b$. Panels correspond to the different integration times: (a) 0.3125 ms, (b) 0.625 ms, (c) 1.25 ms, (d) 2.50 ms, (e) 5.0 ms, (f) 10.0 ms, (g) 20.0 ms, (h) 40.0 ms, (i) 80.0 ms, (j) 160.0 ms). The RTN signal retains an S-shaped curvature and heavy tails due to long-range correlation.

3. Deterministic FTJ precision / resolution

o The deterministic VMM demonstrations show gradual conductance tuning, but the reproducible number of levels or effective bits per cell is never specified. Are the decoders assumed to use full-precision analog weights, or a limited multi-bit resolution? Explicit quantification would allow readers to judge the scalability to larger neural networks. E.g., commercial RRAM cells are 1-bit and never analog. In this regard, I'm not sure how to read Fig. 3h, how can I know how many bits/cell?

Response:

The decoder assumes an effective 4-bit resolution per weight, but this resolution is not obtained through linear quantization. Instead, it reflects the measured non-ideal device characteristics of the FTJ. As shown in Fig. R21, the long-term potentiation (LTP) and long-term depression (LTD) behaviors exhibit nonlinear update profiles, characterized by fitting parameters of β_{LTP} = 4.9 and β_{LTD} = 8.2, respectively. During weight mapping, this nonlinearity is directly incorporated so that the conductance distribution follows the physical device response rather than an idealized uniform quantization grid.

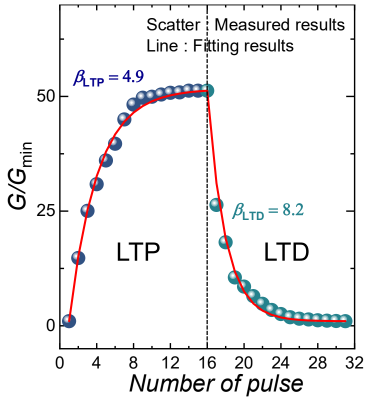

Fig. R21. LTP–LTD characteristics of the FTJ: the solid line shows the nonlinear fit, while the scatter points are the measured data (β_{LTP} = 4.9, β_{LTD} = 8.2).

In addition, Fig. 3h is not a measure of bit resolution, but rather a validation of array-level linearity in vector–matrix multiplication (VMM). In that experiment, cells within a 20 × 20 array were randomly programmed to either the high-resistance state (HRS) or the low-resistance state (LRS), and the number of active (LRS) devices was incrementally increased. The total read current increased linearly with the number of active cells, confirming that the array exhibits accurate linear summation behavior under deterministic read conditions (2 V), where RTN effects are negligible.

To make this point explicit and prevent possible reader confusion, we have added clarifying text to the main manuscript and to the caption of Fig. 3h. Please refer to the revised version for these modifications.

Corresponding change in manuscript: Yes

Corresponding change in manuscript: Yes

Location of Change: Results – (page 8)

The update curves exhibit nonlinearity with β_{LTP} = 4.9 and β_{LTD} = 8.2. Based on these measured nonlinearities, the decoder uses a device-informed 4-bit (16-state) conductance partition, and weight mapping assigns targets according to the β-dependent update characteristics.

Location of Change: Results – (page 8)

Figure 3(h) demonstrates vector–matrix multiplication (VMM) results obtained from the fabricated 20 × 20 NOR-type FTJ array by randomly programming cells to HRS/LRS and stepwise increasing the number of activated (LRS) devices under a 2 V read bias, showing that the column-summed current scales linearly with the activated-device count and thus confirming array-level VMM linearity in the deterministic regime. For decoder VMM we operate at a low read voltage of 2 V, 10 ms to stay in the RTN free regime and to reduce power and IR drop while preserving linear accumulation.

Location of Change: Results – (page 14)

Note that the RTN-based sampler is implemented in hardware (Fig. 5a,b), whereas the decoder VMM is realized in hardware-informed software. In this implementation, weights are mapped to 4-bit (16-state) conductance levels derived from the measured LTP/LTD nonlinearities (β_{LTP} = 4.9, β_{LTD} = 8.2; Fig. 3g), and array-level non-idealities are incorporated by applying a multiplicative noise factor with variance σ extracted from the 20 × 20 array measurements at 2 V (Fig. 3h).

4. Sampling speed and throughput

o The optimal sampling time of ≈36 ms is several orders of magnitude slower than digital RNG or alternative memristor noise sources. The throughput implications should be discussed, including how many samples are required to generate a single MNIST or CelebA image, and whether architectural modifications (e.g. parallelism) could overcome this bottleneck.

Response:

Thank you for your comment. We address the requirement from two perspectives, RTN sampling and VMM throughput/energy.

RTN sampling: To sustain 30 fps, a 256-dimensional latent vector must be sampled 30 times per second. Our study uses bitstream sampling with ≥16 streams, so real-time operation requires 256×30×16=122,880 samplings. Assuming a 36 ms sampling window per FTJ, a single device can provide ~27 samples. Thus ~4,552 FTJs are needed solely for RTN sampling. As software optimization is not our focus, this number can be reduced in practice by lowering the latent dimension or the number of bitstreams.

VMM operation and minimum arraying: We assume multiple arrays, since a single 2-D array cannot cover the full VAE decoder at once. In our decoder, one 128 × 128 image generation requires about 3.57 Giga-MAC. At 30 fps this is ~100 GOPS (≈ 0.1 TOPS). With 256 × 256 FTJ arrays and differential weights (two devices per parameter for positive/negative value representation), the minimum array count is 413 (256 for fully connected layers and 157 for the transposed convolutional layers) [R1]. A 256 × 256 array performs 131,072 operations (MACs) per cycle (counting multiply and add separately as 256 × 256 × 2). Even at a modest 10 MHz operation of the macro, it yields ~1.31 TOPS per array. The different layers demand different workloads (especially convolution). For instance, the last layer with 128 × 128 kernel slides, which is the slowest layer, still meets real-time targets, at 10 MHz the per-inference time is ~1.64 ms, so additional replicated arrays for parallelism are not required.

ADC overhead and read energy: The total ADC energy equals the number of ADC activations per inference counts the sampling energy. For our network, the ADC operates 23,396,352 times per inference. Assuming 1 pJ/sample [R2], this is ~23 µJ total, or ~6.44 fJ per MAC. The read-path energy from synaptic currents, in a worst-case estimate (read time 100 ns, LRS current 2.6 µA, read voltage 2 V, and 3.57×10^9 operations with all weights and inputs at 1), is ~1.85 mJ per inference (~600 fJ/MAC). In a more realistic setting, shorter read time (5 ns) [R3] and average current ~0.1 × LRS from training statistics, the read energy drops to ~9 µJ per inference (~3 fJ/MAC), which is lower than the ADC contribution.

These details are added in the Supplementary Note 7. Please refer to the modified manuscript.

[R1] X. Peng, R. Liu, and S. Yu, "Optimizing Weight Mapping and Data Flow for Convolutional Neural Networks on Processing-in-Memory Architectures", IEEE Transactions on Circuits and Systems-I: Regular Papers, 67, 4, (2020)

[R2] H. Jiang, W. Li, S. Huang, and S. Yu, "Analog-to-Digital Converter Design Exploration for Compute-in-Memory Accelerators", IEEE Design and Test, 39, 2, 48-55 (2022).

[R3] P. Chen, X. Peng, and S. Yu, "DNN+ NeuroSim: An end-to-end benchmarking framework for compute-in-memory accelerators with versatile device technologies", 2017 IEEE International Electron Devices Meeting (IEDM), (2019)

Corresponding change in manuscript: Yes

Location of Change: Results – (page 20)

Supplementary Note 7 presents throughput, array requirements, and energy overhead for RTN sampling, VMM operation, and ADCs, evaluated at 30 fps on CelebA.

Location of Change: Supplementary Note 7

Supplementary Note 7. Throughput, array, and energy overhead in terms of RTN Sampling, VMM operation, and ADCs (30 fps, CelebA)

RTN sampling: To sustain 30 fps, a 256-dimensional latent vector must be sampled 30 times per second. Our study uses bitstream sampling with ≥16 streams, so real-time operation requires 256×30×16=122,880 samplings. Assuming a 36 ms sampling window per FTJ, a single device can provide ~27 samples. Thus ~4,552 FTJs are needed solely for RTN sampling. As software optimization is not our focus, this number can be reduced in practice by lowering the latent dimension or the number of bitstreams.

VMM operation and minimum arraying: We assume multiple arrays, since a single 2-D array cannot cover the full VAE decoder at once. In our decoder, one 128 × 128 image generation requires about 3.57 Giga-MAC. At 30 fps this is ~100 GOPS (≈ 0.1 TOPS). With 256 × 256 FTJ arrays and differential weights (two devices per parameter for positive/negative value representation), the minimum array count is 413 (256 for fully connected layers and 157 for the transposed convolutional layers) [5]. A 256 × 256 array performs 131,072 operations (MACs) per cycle (counting multiply and add separately as 256 × 256 × 2). Even at a modest 10 MHz operation of the macro, it yields ~1.31 TOPS per array. The different layers demand different workloads (especially convolution). For instance, the last layer with 128 × 128 kernel slides, which is the slowest layer, still meets real-time targets, at 10 MHz the per-inference time is ~1.64 ms, so additional replicated arrays for parallelism are not required.

ADC overhead and read energy: The total ADC energy equals the number of ADC activations per inference counts the sampling energy. For our network, the ADC operates 23,396,352 times per inference. Assuming 1 pJ/sample [6], this is ~23 µJ total, or ~6.44 fJ per MAC. The read-path energy from synaptic currents, in a worst-case estimate (read time 100 ns, LRS current 2.6 µA, read voltage 2 V, and 3.57 × 10^9 operations with all weights and inputs at 1), is ~1.85 mJ per inference (~600 fJ/MAC). In a more realistic setting, shorter read time (5 ns) [7] and average current ~0.1× LRS from training statistics, the read energy drops to ~9 µJ per inference (~3 fJ/MAC), which is lower than the ADC contribution.

[R1] X. Peng, R. Liu, and S. Yu, “Optimizing Weight Mapping and Data Flow for Convolutional Neural Networks on Processing-in-Memory Architectures”, IEEE Transactions on Circuits and Systems-I: Regular Papers, 67, 4, (2020)

[R2] H. Jiang, W. Li, S. Huang, and S. Yu, “Analog-to-Digital Converter Design Exploration for Compute-in-Memory Accelerators”, IEEE Design and Test, 39, 2, 48-55 (2022).

[R3] P. Chen, X. Peng, and S. Yu, “DNN+ NeuroSim: An end-to-end benchmarking framework for compute-in-memory accelerators with versatile device technologies”, 2017 IEEE International Electron Devices Meeting (IEDM), (2019)

5. Power and endurance

o Stochastic operation requires biasing into a high-current regime. Please report typical current levels and estimate the energy per random bit/sample, and compare to CMOS or in-memory RNG baselines. Also discuss whether the reported endurance (~10^5 cycles) suffices for practical inference workloads that repeatedly require stochastic sampling.

Response:

Thank you for your comment. The per-sample energy is the sum of the FTJ read current term and the sense-amplifier/flip-flop overhead. For the FTJ term alone, 3.2 V×20 nA×36 ms = 2.3 nJ per random sample. On a per-image basis this is 2.3 nJ×16 (bitstreams) ×256 (latent dimension) = 9.4 μJ. The sampling latency is 36 ms, as discussed in the main text. We have extended Table 1 with this quantitative comparison. To focus the comparison on latency and power in random sampling, we selected more appropriate prior works (CMOS- and ReRAM-based) and compiled them in Table 1.

									Latent Sampling		Decoder Mapping
Work	Latent Sampling	Decoder Mapping	Device type	CMOS compatibility	Array Size	Task	Implementation	Cell Area	Speed*	Power**	TOPS/W
This work	O	O	Both FTJ	O	400	MNIST, CelebA	Circuit-level	2 × 2 μm^2	36 ms	282 μW (FTJ-only)	106 (FTJ & ADC)
[5]	O	-	Bi_2O_2Se	X	1 (Single Device)	MNIST	Device-level	10 × 10 - 40 × 40 μm^2	10 ms	98.1-337 μW	-
[6]	O	-	ReRAM	X	1 (Single Device)	TRNG-only	Circuit-level	5 × 5 - 50 × 50 μm^2	50-250 ms	1.23- 862 μW	-
[7]	O	-	ReRAM	X	1 (Single Device)	TRNG-only	Circuit-level	2.5 × 2.5 μm^2	100 ms	1.47 mW	-
[8]	-	O	ReRAM	O	Only Simulation	MNIST	Simulation-level	-	-	-	4.6
[9]	-	O	1T-1R	O	1000	MNIST	Circuit-level	-	-	-	-
[10]	O	-	CMOS	O	-	TRNG	Circuit-level	-	11.6-312 ns	38.9-91.6 mW	
[11]	-	O	CMOS	O	-	Neural Networks	Commercial	-	-	-	2.3

*Sampling time per bit

**Normalized for 30 fps Human Face Image (128 ×128) Generation

Tera operations per second (TOPS)

Table 1. Comparison of our work with previous reported hardware generative models.

Regarding endurance, the FTJ design has sufficient margin for repeated stochastic operation. Supplementary Fig. 3 shows that the devices remain functional up to 10^7 switching events and that the remnant polarization stays largely unchanged up to 10^5 cycles with only minor degradation. The total of about 15,620 optimizer steps (CelebA is trained for 10 epochs with about 1,562 iterations per epoch. This corresponds to about 15,620 optimizer steps in total.) is well within this endurance envelope.

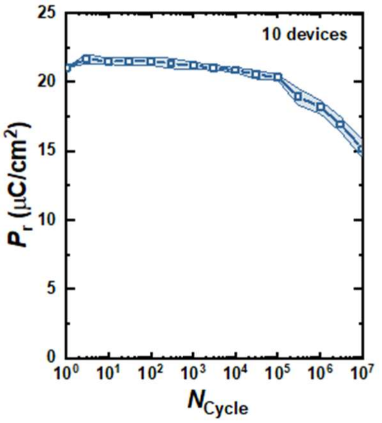

Supplementary Figure 3. Polarization endurance characteristics of the fabricated FTJ device. P_r values are normalized by the initial P_r ($P_{r,initial}$). One rectangular program pulse (5 V, 10 μs) followed by one erase pulse (−5 V, 10 μs) constitutes a single cycle (N_{cycle} = 1). The devices exhibit a slight initial increase in polarization, indicative of a wake-up phenomenon, followed by minor degradation beyond 10^5 cycles, demonstrating robust and stable endurance characteristics. The measurements were performed on 10 devices, and the standard deviation is represented by error bars (shaded in light blue).

In addition, the 10^5 endurance shown in Supplementary Fig. 3 is a very harsh condition relative to neuromorphic learning. The protocol defines each cycle as a full program pulse followed by a full erase pulse, which forces a large polarization swing at every cycle. Neuromorphic training does not apply such full window reprogramming. Instead it uses small verify and adjust nudges that correspond to partial domain motion rather than full reversal. As a result the effective device stress per update is much smaller and many steps remain in the current quantization bin or move by only one bin. Fig. R20 corroborates this by showing trajectories of representative decoder weights over 10 epochs plotted as normalized weight versus epoch. The curves change gradually without abrupt jumps, which indicates incremental updates with limited verify and adjust pulses per step and supports the partial switching interpretation.

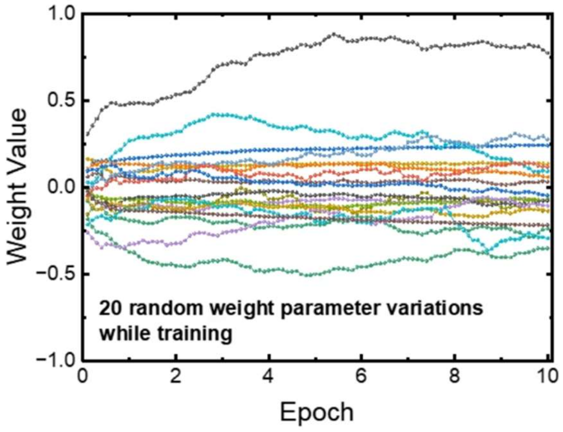

Fig. R20. Evolution of 20 randomly selected weights during CelebA training. Trajectories of 20 randomly chosen network weights over the first 10 epochs. The *x*-axis represents epoch and the *y*-axis shows normalized weight. Each curve corresponds to one parameter and exhibits very small, gradual changes per epoch with no abrupt jumps, indicating slow drift and minimal per-epoch reprogramming demand.

Overall, the actual update count is about 1.6×10^4, the devices sustain 10^5 level cycling with minor degradation and remain functional to 10^7, and the incremental nature of learning imposes far milder stress than conventional endurance testing.

Furthermore, to identify why the FID saturates beyond 10^5 cycles, we performed additional measurements and analysis. We extracted cycle resolved LRS and HRS current distributions at $N_{\text{cycle}} = 10^0$, 10^3, 10^5, and 10^6 and quantified σ_I over I_{avg} as a function of N_{cycle}. Fig. R10 and Fig. R11 show the histograms and Fig. R12 summarizes σ_I over I_{avg}. The HRS spread increases faster than LRS, which reduces the on off ratio and increases read errors during VMM accumulation. This mechanism explains the FID trend at high cycle counts.

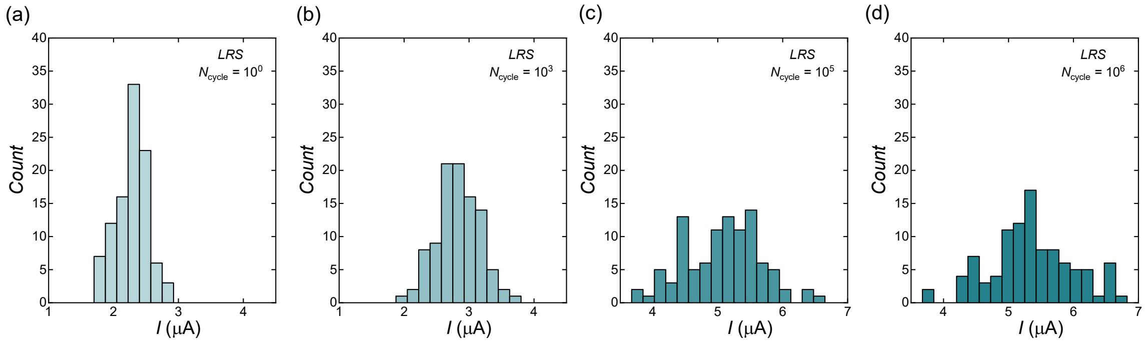

Fig. R10. Cycle-dependent current distribution in LRS. Histograms of cell current in LRS at (a) $N_{\text{cycle}} = 10^0$, (b) $N_{\text{cycle}} = 10^3$, (c) $N_{\text{cycle}} = 10^5$, and (d) $N_{\text{cycle}} = 10^6$.

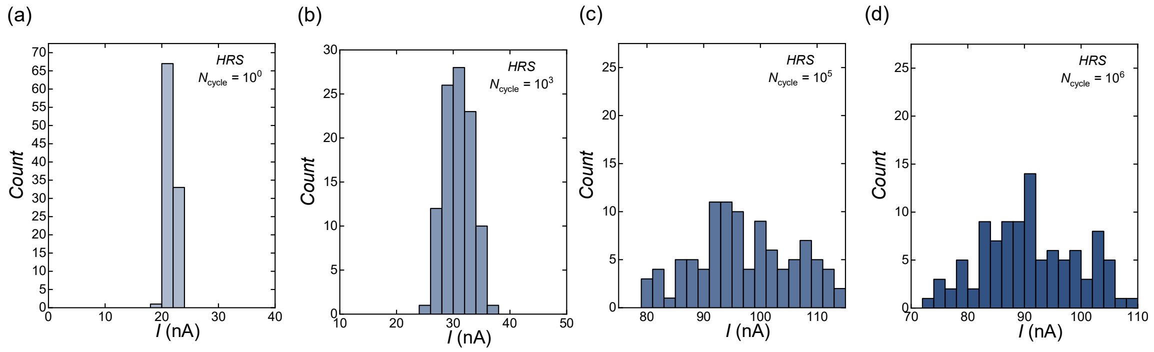

Fig. R11. Cycle-dependent current distribution in HRS. Histograms of cell current in LRS at (a) $N_{cycle} = 10^0$, (b) $N_{cycle} = 10^3$, (c) $N_{cycle} = 10^5$, and (d) $N_{cycle} = 10^6$.

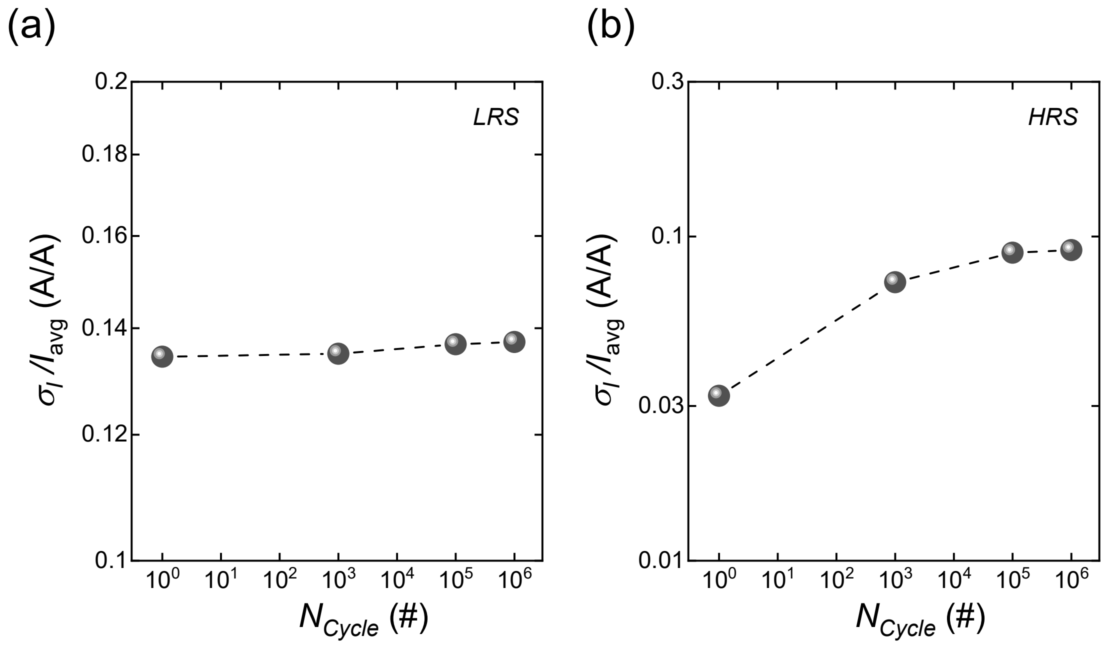

Fig. R12. Spread of current over cycling. (a) σ_I/I_{avg} versus N_{cycle} in LRS. (b) σ_I/I_{avg} versus N_{cycle} in HRS.

We have clarified these points and referenced Table R1, Supplementary Fig. 3, and Fig. R20 in the revised manuscript. Please refer to the revised manuscript for these updates.

Corresponding change in manuscript: Yes

Location of Change: Table 1

									Latent Sampling		Decoder Mapping
Work	Latent Sampling	Decoder Mapping	Device type	CMOS compatibility	Array Size	Task	Implementation	Cell Area	Speed*	Power**	TOPS/W
This work	O	O	Both FTJ	O	400	MNIST, CelebA	Circuit-level	2 × 2 μm²	36 ms	282 μW (FTJ-only)	106 (FTJ & ADC)
[5]	O	-	Bi_2O_2Se	X	1 (Single Device)	MNIST	Device-level	10 × 10 - 40 × 40 μm²	10 ms	98.1-337 μW	-
[6]	O	-	ReRAM	X	1 (Single Device)	TRNG-only	Circuit-level	5 × 5 - 50 × 50 μm²	50-250 ms	1.23- 862 μW	-
[7]	O	-	ReRAM	X	1 (Single Device)	TRNG-only	Circuit-level	2.5 × 2.5 μm²	100 ms	1.47 mW	-
[8]	-	O	ReRAM	O	Only Simulation	MNIST	Simulation-level	-	-	-	4.6
[9]	-	O	1T-1R	O	1000	MNIST	Circuit-level	-	-	-	-
[10]	O	-	CMOS	O	-	TRNG	Circuit-level	-	11.6-312 ns	38.9-91.6 mW	
[11]	-	O	CMOS	O	-	Neural Networks	Commercial	-	-	-	2.3

*Sampling time per bit

**Normalized for 30 fps Human Face Image (128 ×128) Generation

Tera operations per second (TOPS)

Table 1. Comparison of our work with previous reported hardware generative models.

Location of Change: Introduction – (page 2)

As a result, prior efforts have largely depended on hybrid architectures or off-chip software-based stochastic modules, constraining system scalability, energy efficiency, and integration density [5-11]. Moreover, in mature CMOS-based technologies, separate studies have been proposed to perform each role, implementing stochastic functionality [8] and handling deterministic models [11,12].

Location of Change: Introduction – (page 3)

While recent studies leverage device noise to realize continuous weight distributions for classification, our work focuses on hardware-native generation by using RTN for latent sampling and FTJ-based VMM for decoding within a single array [21,22].

Location of Change: Results – (page 20)

Supplementary Note 7 presents throughput, array requirements, and energy overhead for RTN sampling, VMM operation, and ADCs, evaluated at 30 fps on CelebA.

Location of Change: Results – (page 21)

FTJ-based platform is also quantitatively benchmarked against the mature CMOS-based designs in area, power per sample, and latency (Table 1). For VMM specifically, our FTJ arrays achieve <10 fJ/MAC, over 40× lower

than the ~430 fJ/MAC reported for 8-bit MACs on a widely deployed TPU [11], and sustain ~1.31 TOPS per 256×256 array at 10 MHz, with throughput scaling through array replication.

Location of Change: Fig. 6(i)

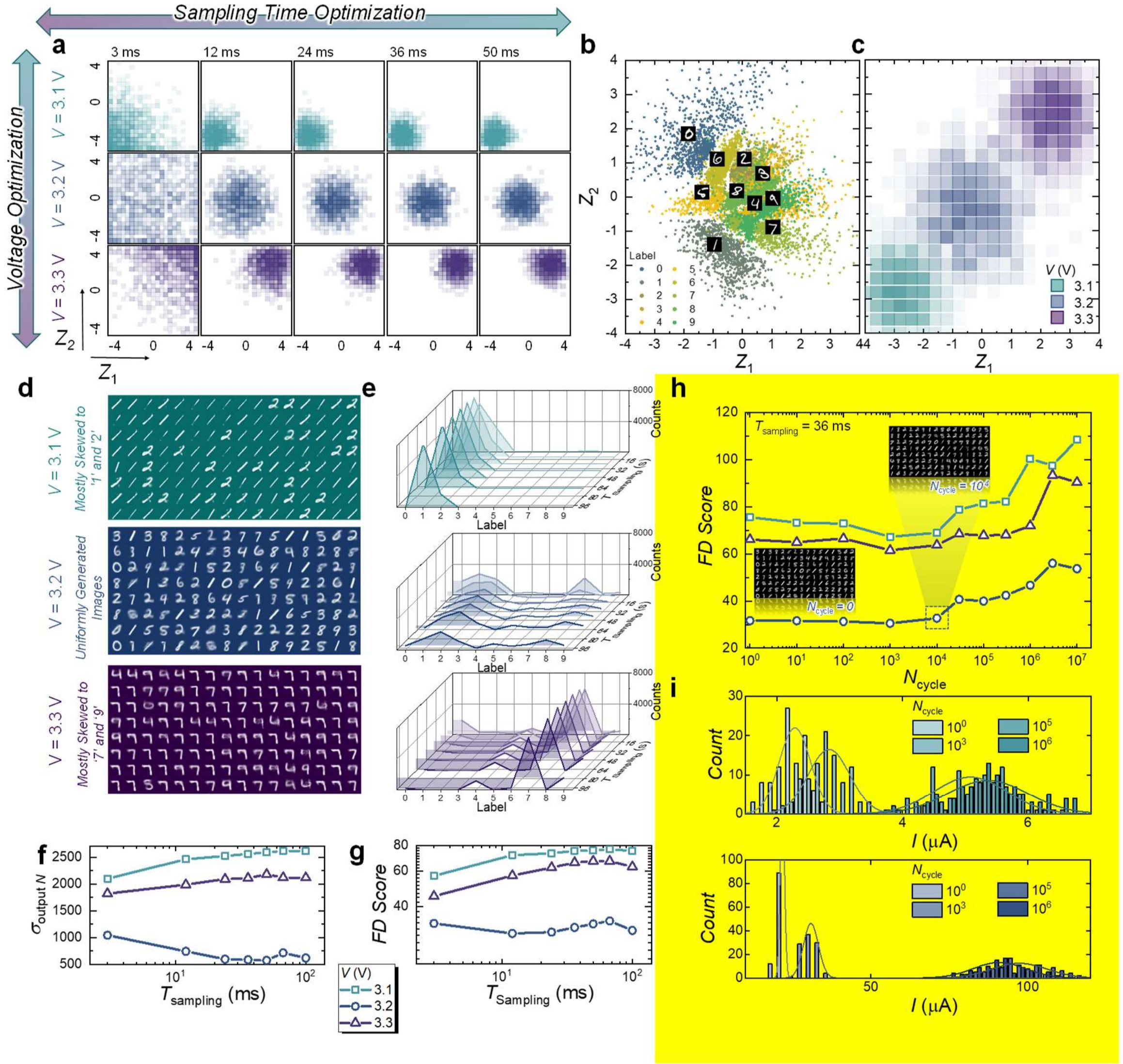

Figure 6. Evaluation of latent space distribution and image generation performance under varying voltage and sampling conditions. **a** Distribution of generated latent vectors for each V and T_{Sampling}, with biased distributions observed at 3.1 V and 3.3 V. **b** Latent space for the actual MNIST dataset, showing the ideal distribution for accurate image generation. **c** Distribution of random variables in the latent space generated by varying V at fixed T_{Sampling} = 36 ms. **d** Generated MNIST images at different voltages. At V = 3.1 V, only digits "1" and "2" are generated, while V =3.2 V produces all digits evenly, and V =3.3 V is biased toward digits "7" and "9." **e** Distribution of generated images by label across different voltages and sampling conditions, with V = 3.2 V showing the most uniform distribution. **f** $\sigma_{\mathrm{output\ N}}$ of generated images by label, with V = 3.2 V and T_{Sampling} = 36 ms showing the lowest deviation. **g** FD score of generated images, with V = 3.2 V and T_{Sampling} = 36 ms presenting the lowest FD score, indicating the highest image diversity. **h** FD score versus N_{cycle}s, confirming robust FTJ

operation in the absence of RTN. **i** Cycle-dependent current distribution in HRS (up) and LRS (down). Histograms of cell current in LRS at (a) N_{cycle} = 10^0, (b) N_{cycle} = 10^3, (c) N_{cycle} = 10^5, and (d) N_{cycle} = 10^6.

Location of Change: Results – (page 19)

Figure 6(h) presents the FD score as a function of N_{cycle}, demonstrating that stable image generation is maintained even up to 10^5 generation cycles. Figure 6(i) further shows the cycle-dependent current distributions in HRS (top) and LRS (bottom): histograms at (a) N_{cycle}=10^0, (b) 10^3, (c) 10^5, and (d) 10^6. The HRS peak broadens and drifts more strongly than LRS, increasing their overlap and effectively reducing the on/off ratio, which correlates with the slight FD increase at high cycle counts. Effects of RTN operating region and write-cycle count on MNIST generation are shown in Supplementary Fig. 26. This result confirms the robustness and reliability of the system under repeated operation.

6. Comparisons with digital approaches

o The manuscript argues CMOS/GPU alternatives are less efficient, but provides no quantitative evidence. Thorough comparisons in area efficiency, power per sample, or latency seem necessary to justify your claims.

Response:

Thank you for your comment. As noted in the main text, our focus is a more scalable and energy-efficient FTJ platform. For random sampling (RNG), we provide quantitative comparisons in **response to comment 5** and in the extended Table 1, covering area, power per sample, and latency against CMOS- and ReRAM-based prior art.

For the VMM, we also benchmark against a widely deployed advanced accelerator TPU (as a representative GPU-class device). Reported figures for 8-bit integer MACs on TPU are ~430 fJ/MAC energy and ~92 TOPS peak throughput [R1]. In our FTJ-based VMM, the energy per MAC is < 10 fJ, roughly implying > 40× energy efficiency improvement relative to the above TPU figure. Throughput varies with array size and replication. A 256 × 256 FTJ array executing at 10 MHz delivers 256 × 256 × 2 × 10 MHz ≈ 1.31 TOPS per array. Larger arrays or multiple replicated arrays increase TOPS proportionally, so meeting or exceeding TPU-class throughput is feasible without sacrificing efficiency. Quantitative metrics are included in the extended Table 1. Please Refer to the modified manuscript.

[R1] Norman P. Jouppi *et al.*, *arXiv preprint*, arXiv:1704.04760, (2017)

Corresponding change in manuscript: Yes

Location of Change: Table R1

									Latent Sampling		Decoder Mapping
Work	Latent Sampling	Decoder Mapping	Device type	CMOS compatibility	Array Size	Task	Implementation	Cell Area	Speed*	Power**	TOPS/W
This work	O	O	Both FTJ	O	400	MNIST, CelebA	Circuit-level	2 × 2 μm²	36 ms	282 μW (FTJ-only)	106 (FTJ & ADC)
[5]	O	-	Bi_2O_2Se	X	1 (Single Device)	MNIST	Device-level	10 × 10 - 40 × 40 μm²	10 ms	98.1-337 μW	-
[6]	O	-	ReRAM	X	1 (Single Device)	TRNG-only	Circuit-level	5 × 5 - 50 × 50 μm²	50-250 ms	1.23- 862 μW	-
[7]	O	-	ReRAM	X	1 (Single Device)	TRNG-only	Circuit-level	2.5 × 2.5 μm²	100 ms	1.47 mW	-
[8]	-	O	ReRAM	O	Only Simulation	MNIST	Simulation-level	-	-	-	4.6
[9]	-	O	1T-1R	O	1000	MNIST	Circuit-level	-	-	-	-
[10]	O	-	CMOS	O	-	TRNG	Circuit-level	-	11.6-312 ns	38.9-91.6 mW	
[11]	-	O	CMOS	O	-	Neural Networks	Commercial	-	-	-	2.3

*Sampling time per bit

**Normalized for 30 fps Human Face Image (128 ×128) Generation

Tera operations per second (TOPS)

Table 1. Comparison of our work with previous reported hardware generative models.

Location of Change: Introduction – (page 2)

As a result, prior efforts have largely depended on hybrid architectures or off-chip software-based stochastic modules, constraining system scalability, energy efficiency, and integration density [5-11]. Moreover, in mature CMOS-based technologies, separate studies have been proposed to perform each role, implementing stochastic functionality [8] and handling deterministic models [11,12].

Location of Change: Results – (page 21)

FTJ-based platform is also quantitatively benchmarked against the mature CMOS-based designs in area, power per sample, and latency (Table 1). For VMM specifically, our FTJ arrays achieve <10 fJ/MAC, over 40× lower than the ~430 fJ/MAC reported for 8-bit MACs on a widely deployed TPU [11], and sustain ~1.31 TOPS per 256×256 array at 10 MHz, with throughput scaling through array replication.

7. Scalability and variability

o Memristive device variability is a key concern. While reproducibility data across 10–20 devices are encouraging, a more explicit discussion on:

- how variability distorts latent distributions or biases image outputs;

- potential IR-drop, routing, or yield issues when arrays scale beyond 20×20;

- implications for manufacturing scalability using industry HfO2 processes.

Response:

Thank you for your comment. As the reviewer pointed out, scalability and variability should be discussed explicitly across device statistics, array level behavior, and process readiness. We therefore summarize wafer scale uniformity and controllability of RTN, quantify IR drop and routing as arrays scale beyond 20×20, and explain how the proposed FTJ platform aligns with industry HfO_2 manufacturing.

We first address variability and its controllability. Fig. R1 shows an ellipsometry thickness map of ALD HZO over a 6 inch wafer and the corresponding radial profile. The film thickness is highly uniform with only a slight edge related decrease, and the positions of the ten devices used for RTN measurements are indicated. For these devices at V = 3.28 V, Fig. R2 presents the dwell time histograms of τ_L and τ_H with Poisson fits, and Fig. R3 summarizes the device wise read bias and fitted time constants. A modest spread is observed that we attribute to small differences in trap depth and energy. The CelebA image generation results already verify that this level of device-to-device variation does not impair image quality or diversity. Nevertheless, to address the reviewer's question and to confirm that the method remains scalable to larger and more complex tasks, we performed additional experiments demonstrating that the controllability of RTN can be further improved by electrical trimming.

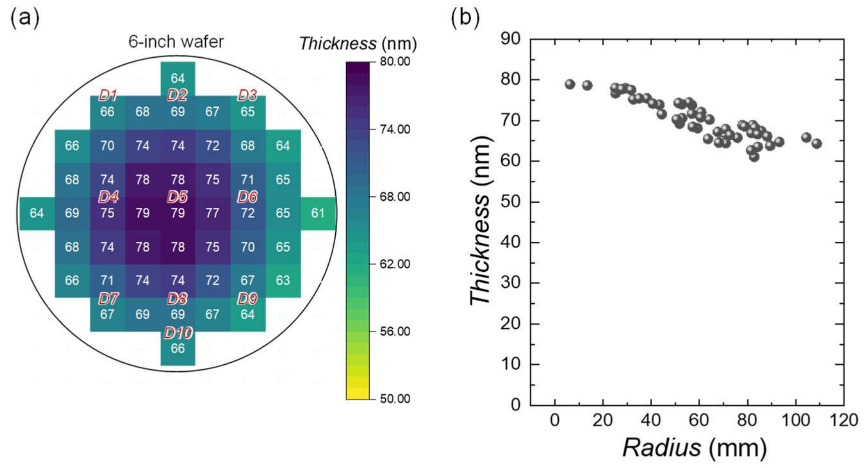

Fig. R1. Wafer-scale thickness uniformity of ALD HZO. (a) Six-inch wafer map showing ellipsometry-measured HZO thickness at 49 sampled points. The locations of the 10 devices (D1 to D10) used for RTN

characterization are also marked on the map. (b) Thickness versus radial distance from the wafer center, showing a slight decreasing trend toward the edge.

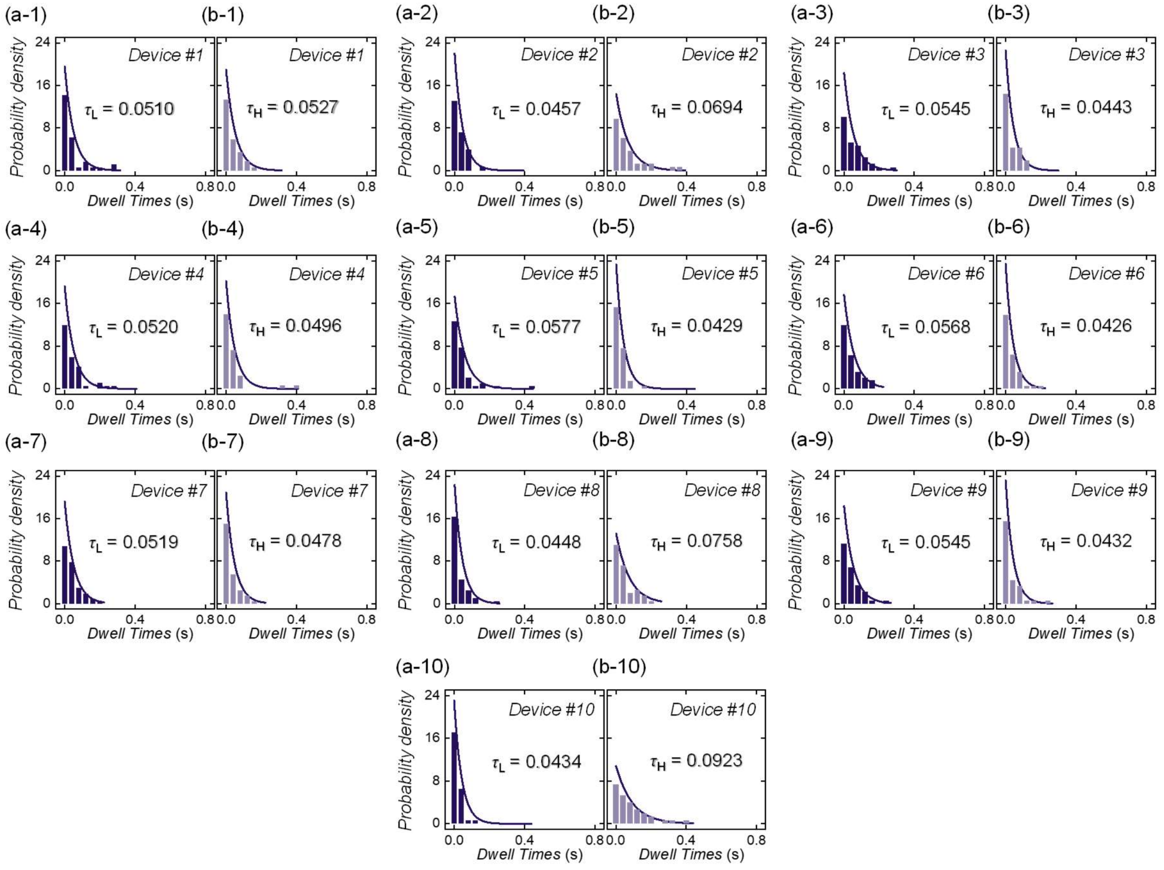

Fig. R2. RTN dwell-time statistics at a single bias (a) Histograms of the low-state dwell time τ_L for 10 devices measured at V = 3.28 V with Poisson (exponential) fits. (b) Histograms of the high-state dwell time τ_H for the same devices and bias with Poisson fits. Across devices the RTN statistics follow Poisson processes and the occupancy of the two levels is balanced at this bias.

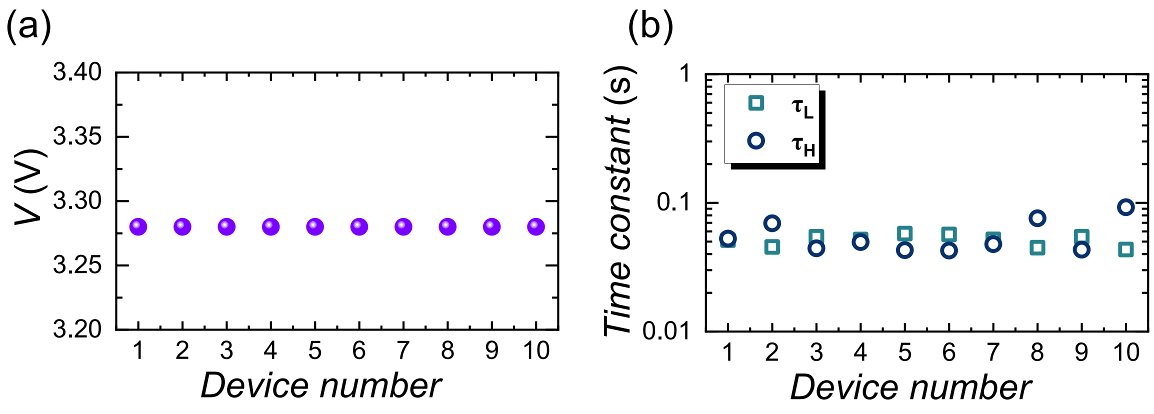

Fig. R3. Device-wise bias and fitted time constants. (a) Applied read voltage for each device used in Fig. R1.

(b) Dwell-time constants extracted from Poisson fitting, reported per device for both τ_L and τ_H.

To verify this controllability, we fine tuned the read bias in 0.001 V steps based on the measured imbalance between τ_L and τ_H. Specifically, when τ_L exceeded τ_H, the bias was increased by 0.001 V. When τ_H was larger, the bias was reduced by 0.001 V. After each adjustment we acquired a 5 s current trace at 3200 Hz and refit the dwell time histograms with exponential models to update τ_L and τ_H. This procedure was repeated until the two dwell times converged within tolerance. Fig. R4(a) shows the τ_L histograms after per device equalization within 3.25 to 3.30 V, and Fig. R4(b) shows the corresponding τ_H histograms, both exhibiting more balanced occupancy than in Fig. R2. Fig. R5(a) summarizes the optimized bias for each device after equalization, and Fig. R5(b) shows the fitted τ_L and τ_H values under these optimized conditions, now closely clustered compared with Fig. R3(b). In practice, this trimming can be applied per device or per column using existing word-line or bit-line DACs, and once set it remains stable over extended operation. These results confirm that, beyond the CelebA demonstration, the same procedure can yield a more uniform latent space and higher-fidelity image generation while simultaneously reducing device-level variability and enhancing RTN controllability.

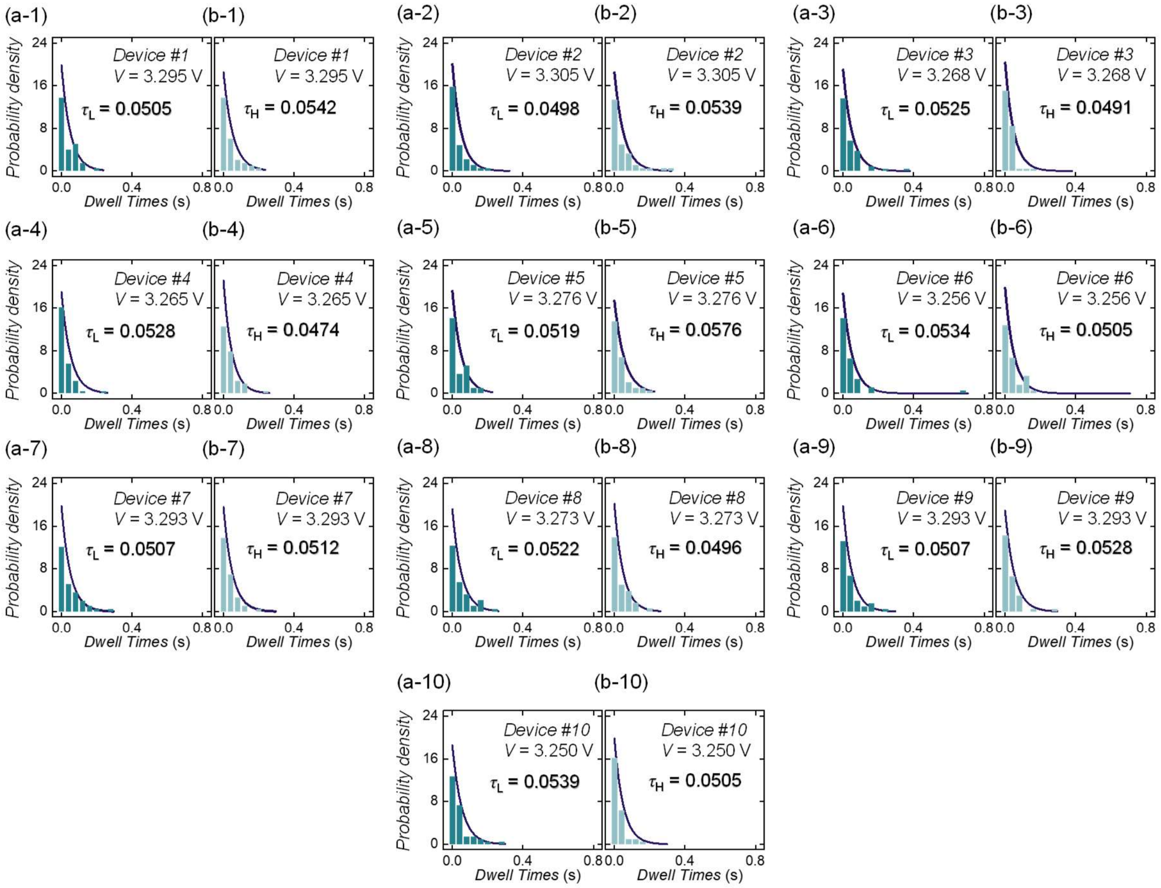

Fig. R4. Bias equalization across devices for balanced occupancy. (a) Histograms of τ_L with Poisson fits for 10 devices, each biased at its own equalized voltage in the range 3.25–3.30 V to balance state occupancy. (b) Corresponding τ_H histograms with Poisson fits. Compared with Fig. R1, per-device bias equalization yields

more balanced occupancy. Inset shows the applied voltage for each device.

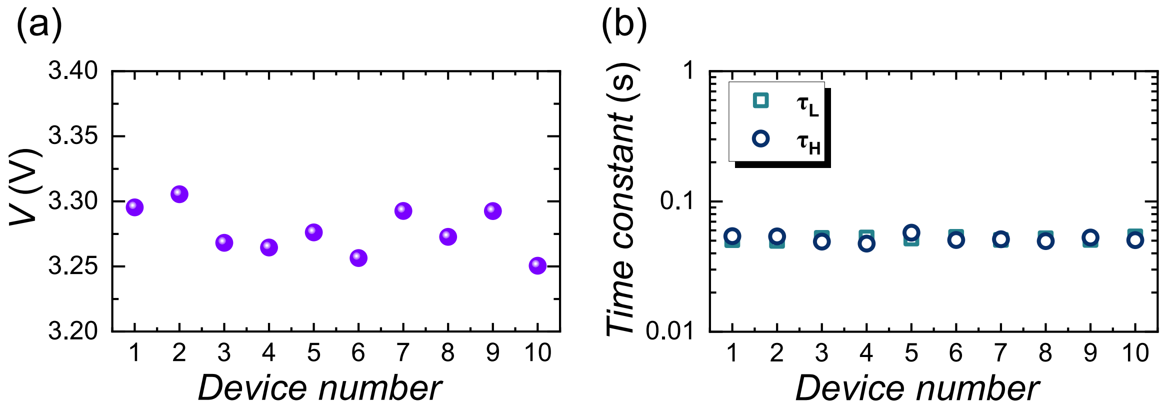

Fig. R5. Summary of equalized bias and fitted constants. (a) Optimized read voltage for each device. (b) Dwell-time constants τ_L and τ_H under the optimized bias for each device, showing markedly tighter clustering than in Fig. R2b.

We next discuss array level scalability, routing, IR drop, and yield beyond the 20×20 demonstrator. The FTJ cell is a standard $4F^2$ cross point with orthogonal word lines and bit lines, so routing follows established RRAM crossbar practice without special requirements. Larger systems are built by tiling macros such as 256×256 or 512×512 with local row drivers, column integrators, and SAR ADCs, and tiles are stitched using conventional back end metal stacks as in compute in memory RRAM chips [R1, R2]. During VMM the column integrators hold the bit lines at virtual ground while the word lines are driven, which eliminates classic crossbar sneak paths. Under these conditions IR drop is the dominant non-ideality. Using our measured sheet resistance of about 0.02 ohm per square, a 1024×1024 array yields a worst case line resistance of about 40 ohm on either a word line or a bit line. With an aggregate read current near 230 mA, the voltage loss is about 10 millivolt per metal line. Even if we conservatively assume 10 mV on both the driven word line and the sensed bit line, the attenuation of the largest cell contribution is only about seven percent. We modeled this as an address dependent gain and simulated image generation. As summarized in Fig. R9, the impact on FID is negligible at the 1024×1024 scale, and smaller tiles further reduce both parasitic resistance and summed current. Yield considerations follow standard practice, including spare rows and columns with remapping, device screening, differential encoding, and per tile operating point calibration.

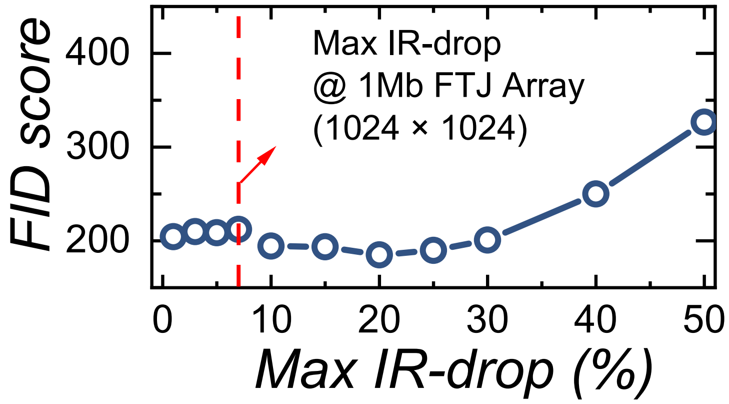

Fig. R9. FID degrades with increasing IR-drop. Red dashed line marks the worst-case IR-drop for a 1 Mb (1024×1024) FTJ array.

Finally, we consider manufacturing scalability with HfO_2 processes. HfO_2 based ferroelectrics are CMOS compatible and share materials and unit processes with high-*k* metal gate technology that has been in production for many years. Atomic layer deposition provides wafer scale control of thickness and stoichiometry and is widely adopted in logic and memory manufacturing [R3-R6]. The wafer level uniformity and device wise reproducibility in Figs. R1 to R5 confirm process maturity, and the simple bias trimming described above closes the residual spread. These points indicate that the proposed FTJ platform fits mainstream back end integration and is manufacturable at scale.

We have added these clarifications in the revised manuscript, including the wafer scale ALD uniformity, the per device bias equalization procedure, the array routing and IR drop analysis with Fig. R9, and the manufacturing considerations for HfO2. Please refer to the revised manuscript for these updates.

[R1] X. Peng, S. Huang, Y. Luo, X. Sun, S. Yu, DNN+NeuroSim: An end-to-end benchmarking framework for compute-in-memory accelerators with versatile device technologies. IEEE International Electron Devices Meeting (IEDM), 771-774 (2019).

[R2] C. Yu, T. Yoo, K. T. C. Chai, T. T. H. Kim, B. Kim, A 65-nm 8T SRAM compute-in-memory macro with column ADCs for processing neural networks. IEEE Journal of Solid-State Circuits 57, 3466–3476 (2022)

[R3] Schroeder, U., Park, M. H., Mikolajick, T. & Hwang, C. S. The fundamentals and applications of ferroelectric HfO2. Nat. Rev. Mater. 7, 653–669 (2022).

[R4] Mikolajick, T., Park, M. H., Begon-Lours, L. & Slesazeck, S. From ferroelectric material optimization to neuromorphic devices. Adv. Mater. 35, 2206042 (2023).

[R5] Park, M. H. et al. Ferroelectricity and antiferroelectricity of doped thin HfO2-based films. Adv. Mater. 27, 1811–1831 (2015).

[R6] Müller, J. et al. Ferroelectricity in simple binary ZrO2 and HfO2. Nano Lett. 12, 4318–4323 (2012).

Corresponding change in manuscript: Yes

Location of Change: Supplementary Figure 12-16

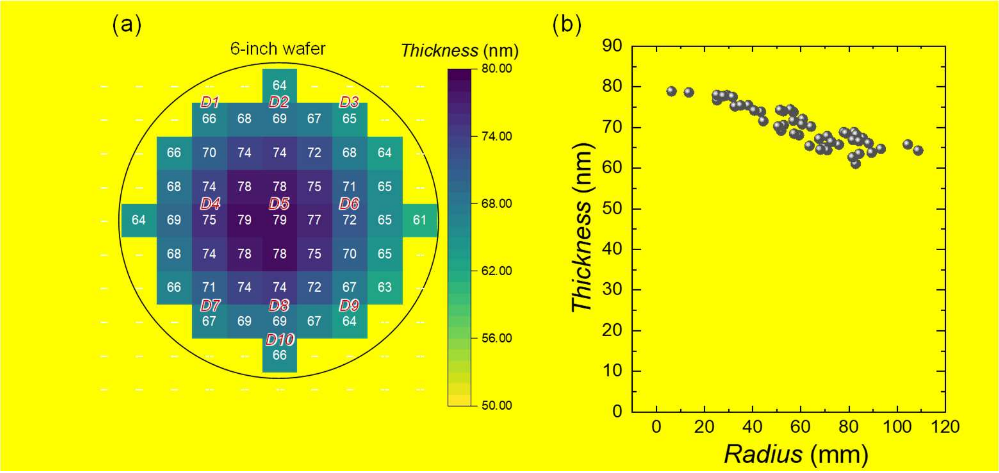

Supplementary Figure 12. Wafer-scale thickness uniformity of ALD HZO. (a) Six-inch wafer map showing ellipsometry-measured HZO thickness at 49 sampled points. The locations of the 10 devices (D1 to D10) used for RTN characterization are also marked on the map. (b) Thickness versus radial distance from the wafer center, showing a slight decreasing trend toward the edge.

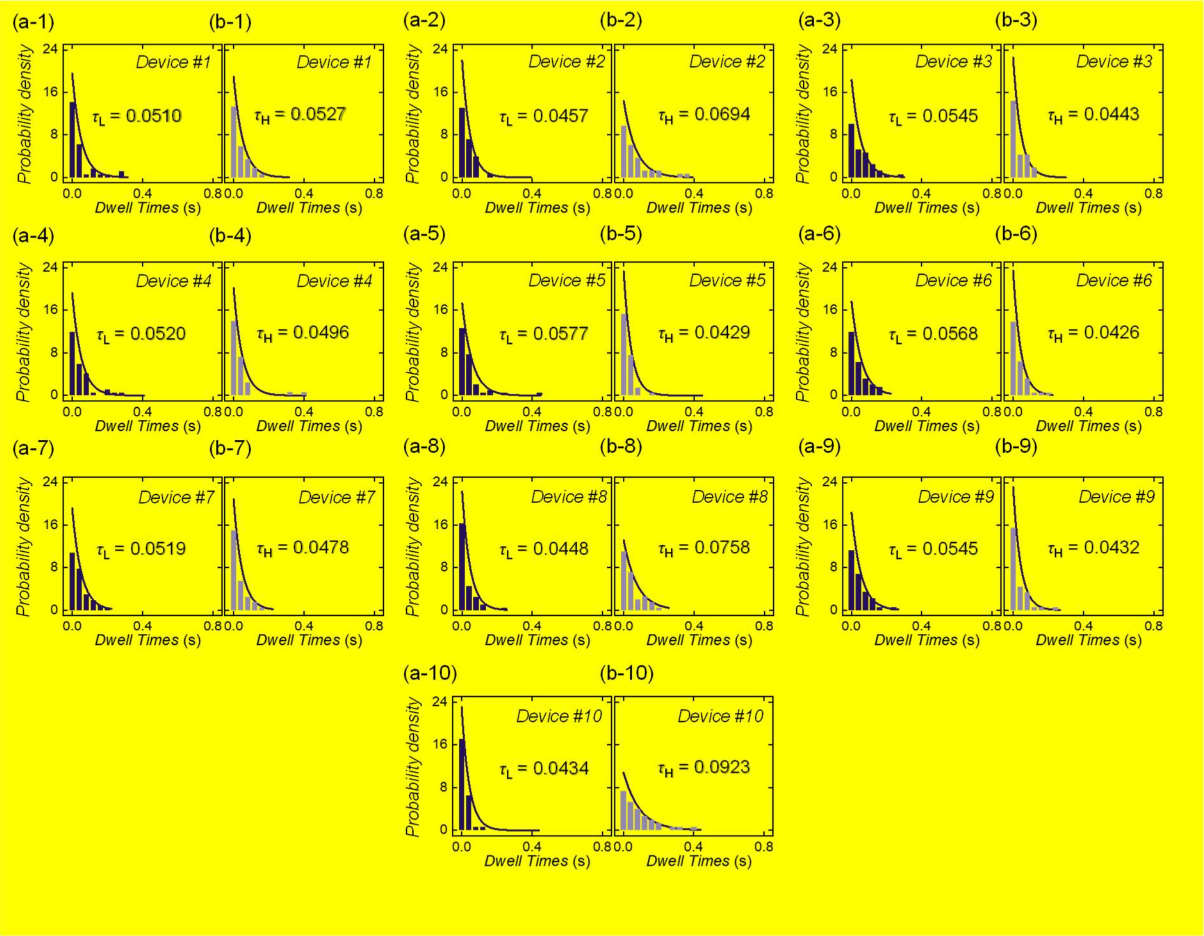

Supplementary Figure 13. RTN dwell-time statistics at a single bias (a) Histograms of the low-state dwell time τ_L for 10 devices measured at V = 3.28 V with Poisson (exponential) fits. (b) Histograms of the high-state dwell time τ_H for the same devices and bias with Poisson fits. Across devices the RTN statistics follow Poisson processes and the occupancy of the two levels is balanced at this bias.

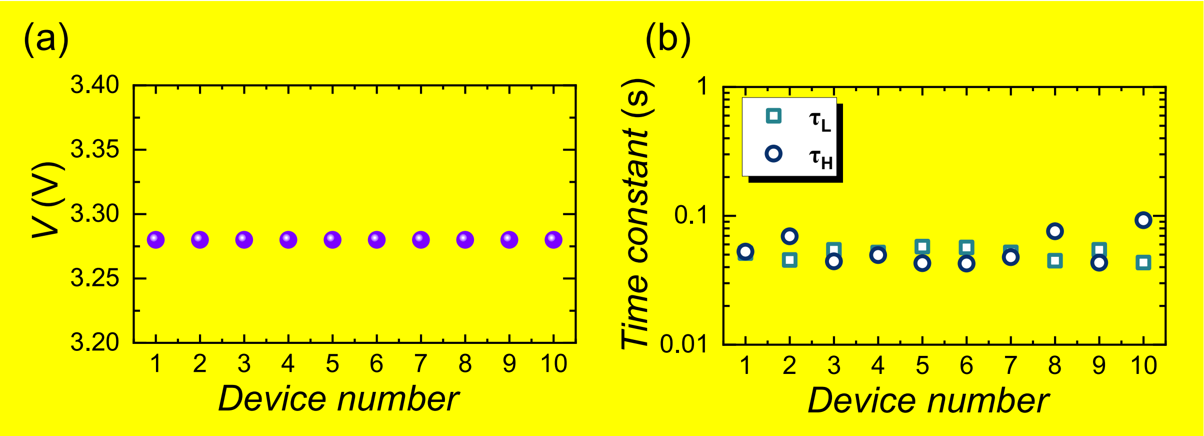

Supplementary Figure 14. Device-wise bias and fitted time constants. (a) Applied read voltage for each device used in Fig. R1. (b) Dwell-time constants extracted from Poisson fitting, reported per device for both τ_L and τ_H.

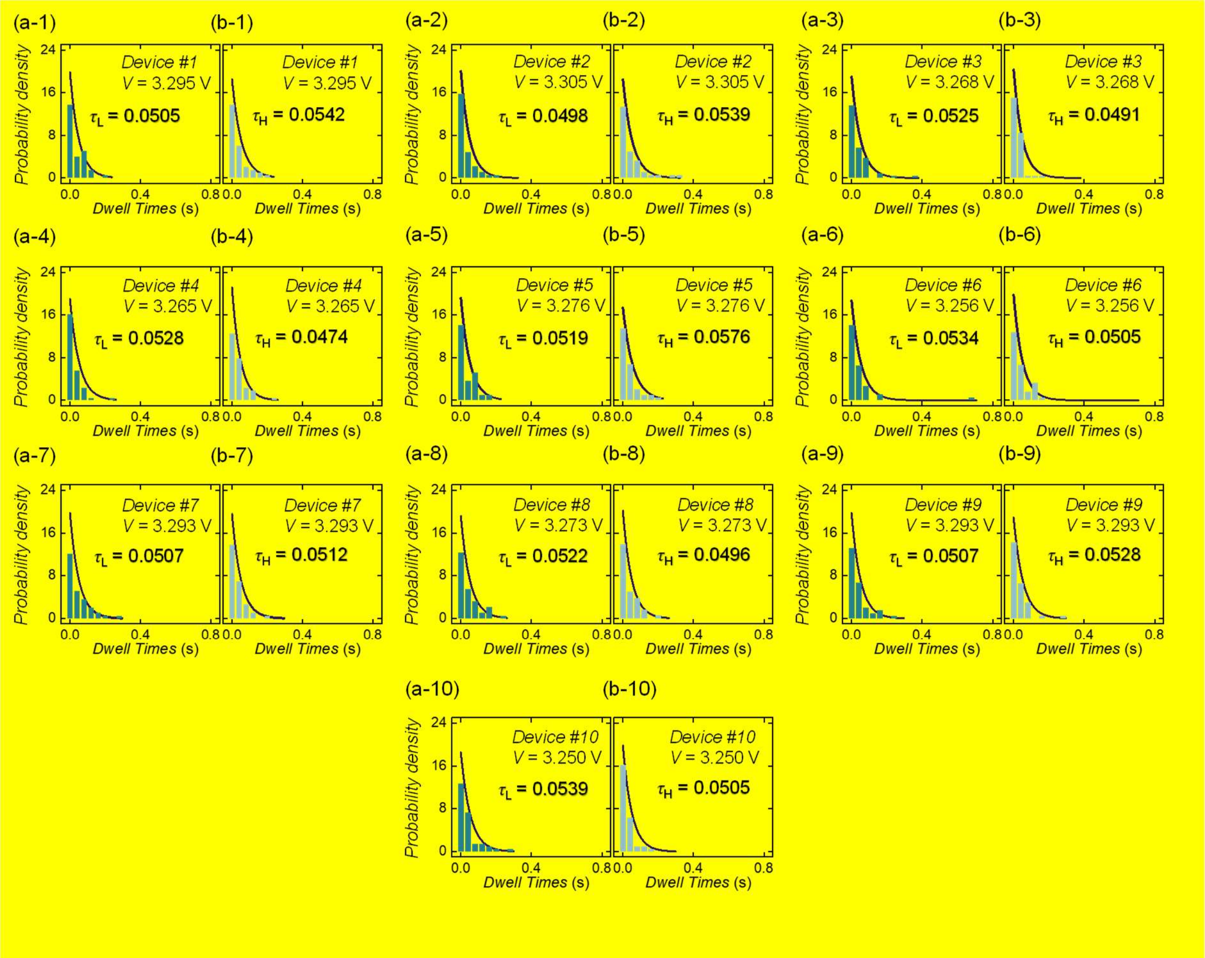

Supplementary Figure 15. Bias equalization across devices for balanced occupancy. (a) Histograms of τ_L with Poisson fits for 10 devices, each biased at its own equalized voltage in the range 3.25–3.30 V to balance state occupancy. (b) Corresponding τ_H histograms with Poisson fits. Compared with Fig. R1, per-device bias equalization yields more balanced occupancy. Inset shows the applied voltage for each device.

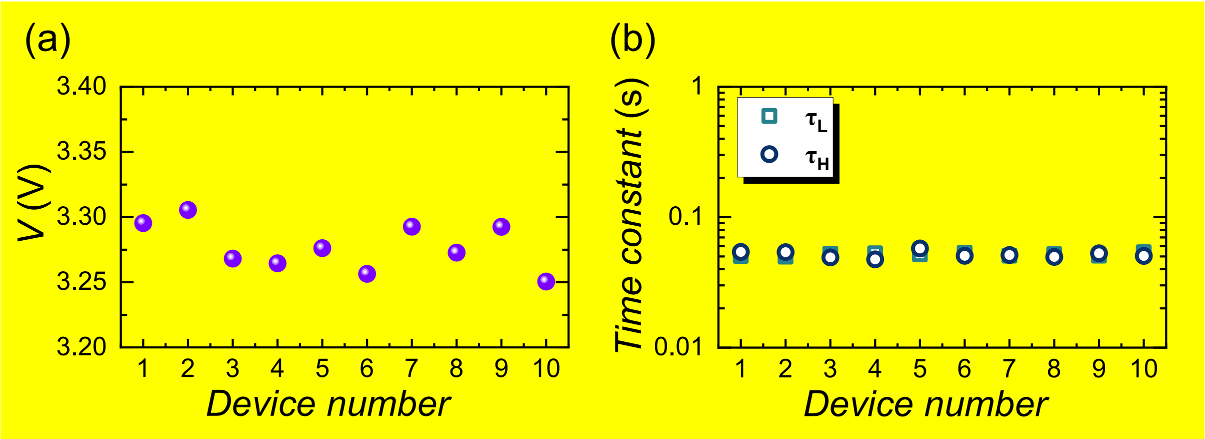

Supplementary Figure 16. Summary of equalized bias and fitted constants. (a) Optimized read voltage for

each device. (b) Dwell-time constants τ_L and τ_H under the optimized bias for each device, showing markedly tighter clustering than in Fig. R2b.

Location of Change: Results – (pages 12-13)

To further validate that the RTN characteristics originate from ALD-related traps and remain controllable across the wafer, we measured the HZO thickness and RTN uniformity over a 6-inch wafer (Supplementary Fig. 12). The thickness map and device-wise dwell-time statistics (Supplementary Fig. 13 and Supplementary Fig. 14) confirm uniform Poisson-type RTN behavior with minor device-to-device variation caused by small differences in trap depth and energy level. By iteratively adjusting the read bias in 1 mV steps according to the τL/τH imbalance, the distributions were equalized across all devices (Supplementary Fig. 15 and Supplementary Fig. 16). This simple voltage-trimming method, readily implemented through word- or bit-line DAC control, enables parallel generation of uniform random bitstreams even with slight ALD-induced variability.

Location of Change: Results – (page 20)

Figure 8(f) presents the FID score as a function of the N_{cycle} applied to the FTJ in the decoder, showing that stable image generation is maintained up to 10^5 read cycles. Effects of RTN operating region and write-cycle count on CelebA generation are shown in Supplementary Fig. 17. Also, the change in FID for CelebA images generated under varying IR-drop is presented in Supplementary Fig. 32. This indicates that the proposed FTJ array can generate high-quality images without being electrically affected by IR-drop.

Location of Change: Supplementary Fig. 32

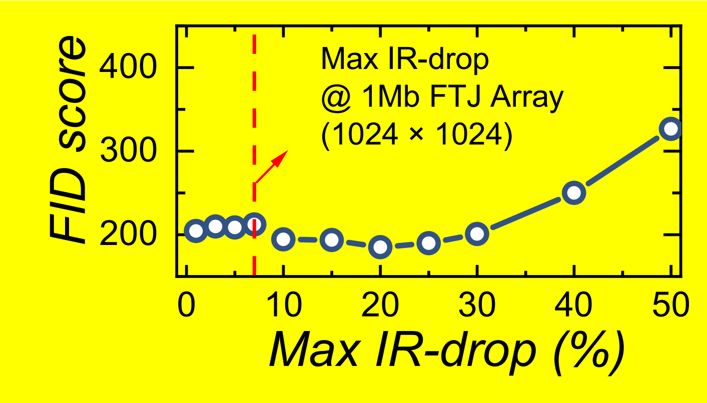

Supplementary Fig. 32. FID degrades with increasing IR-drop. Red dashed line marks the worst-case IR-drop for a 1 Mb (1024×1024) FTJ array.

8. Presentation of noise characterization

o Figures 4–5 contain highly detailed RTN analyses. Consider moving some of these results to the Supplementary, emphasizing instead the clear link from trap physics -> controllable stochasticity -> image generation quality.

Response:

Thank you for your comment. We appreciate your suggestion regarding the presentation of the RTN noise characterization data. Accordingly, we merged the original Figures 4 and 5 into a single figure (Fig.4) in the main text that emphasizes the causal chain from trap position and energy to voltage controlled RTN statistics. Fig. 4(d), 4(e), 4(f), 4(g), 4(h), 4(i), and 4(k) have been moved to the Supplementary Information. Please refer to the revised manuscript to find the modification.

Corresponding change in manuscript: Yes

Location of Change: Fig. 4

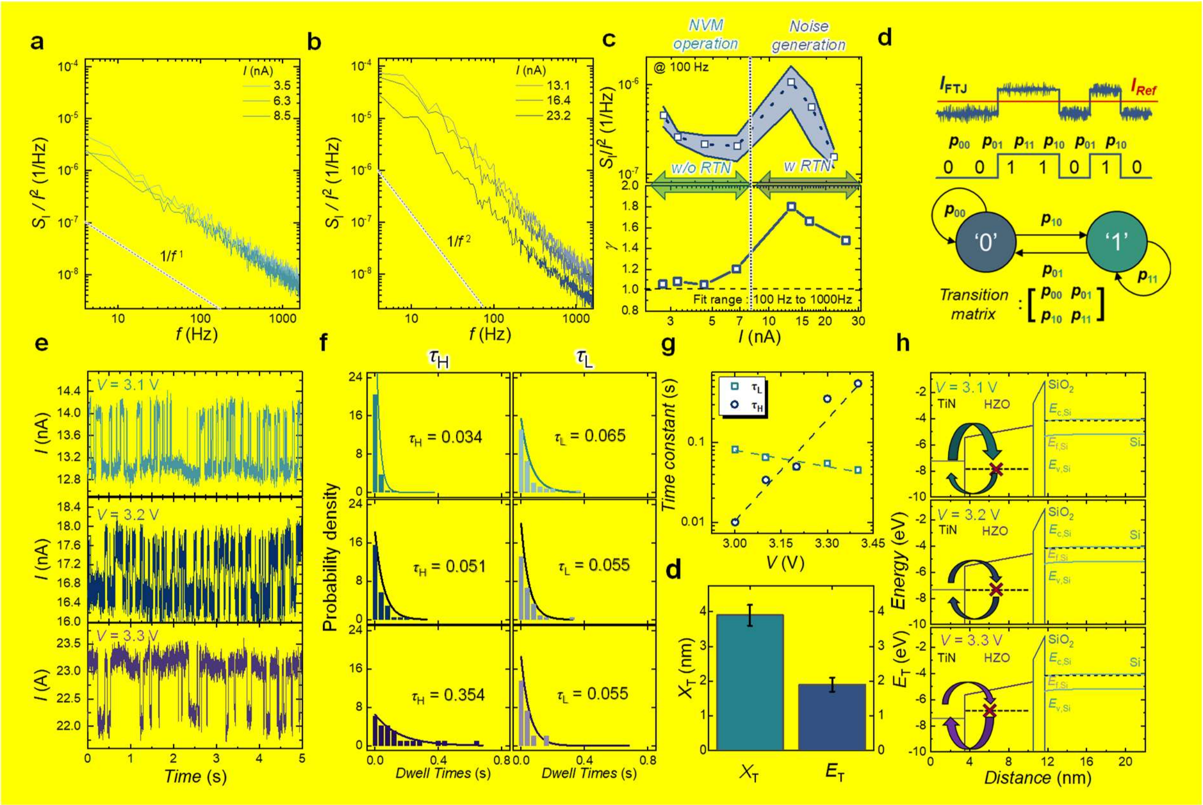

9. Clarity of system integration

o A computational flow diagram showing explicitly how stochastic sampling outputs are combined with deterministic weight arrays through the VAE pipeline would largely improve accessibility, especially for a broad NC readership.

Response:

Thank you for your comment. To improve accessibility for a broad NC readership, we have added a compact computational flow diagram (Fig. R22) that explicitly shows how stochastic sampling outputs are combined with deterministic weight arrays throughout the VAE pipeline. Using CelebA as the example, the diagram illustrates (i) latent-space sampling and (ii) image synthesis through the decoder network, and it annotates where FTJ devices are required and what device characteristics are desirable at each stage. We believe this schematic will help readers grasp the end-to-end concept at a glance and follow the paper more easily. Please refer to the revised manuscript to find the modification.

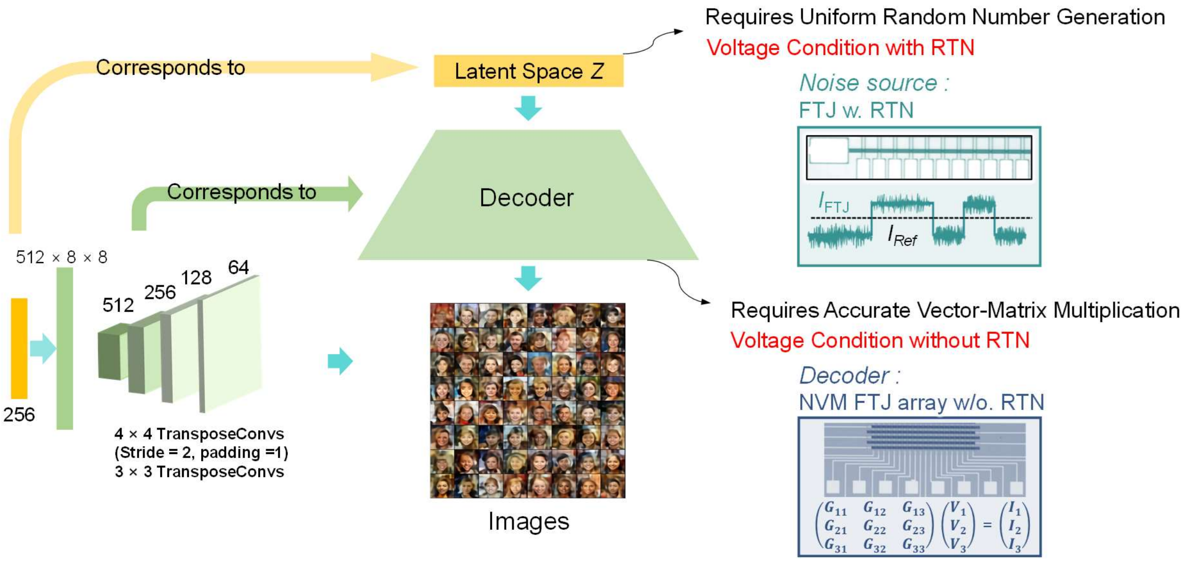

Figure R22. VAE image generation flow: latent vector z is stochastically sampled (FTJ under RTN for uniform RNG) and fed to a transposed convolution decoder whose deterministic weights are realized by an NVM FTJ crossbar operated without RTN, producing images.

Corresponding change in manuscript: Yes

Location of Change: Introduction – (page 4)

This dual-mode FTJ platform thus addresses the longstanding challenge of co-implementing diversity and determinism in hardware-based generative models, offering an energy- and area-efficient, CMOS-compatible pathway toward next-generation neuromorphic and AI accelerators. Supplementary Fig. 1 shows the schematic of FTJ operation, which can be configured to switch between stochastic and deterministic modes as needed for the image generation task.

Location of Change: Supplementary Figure 1

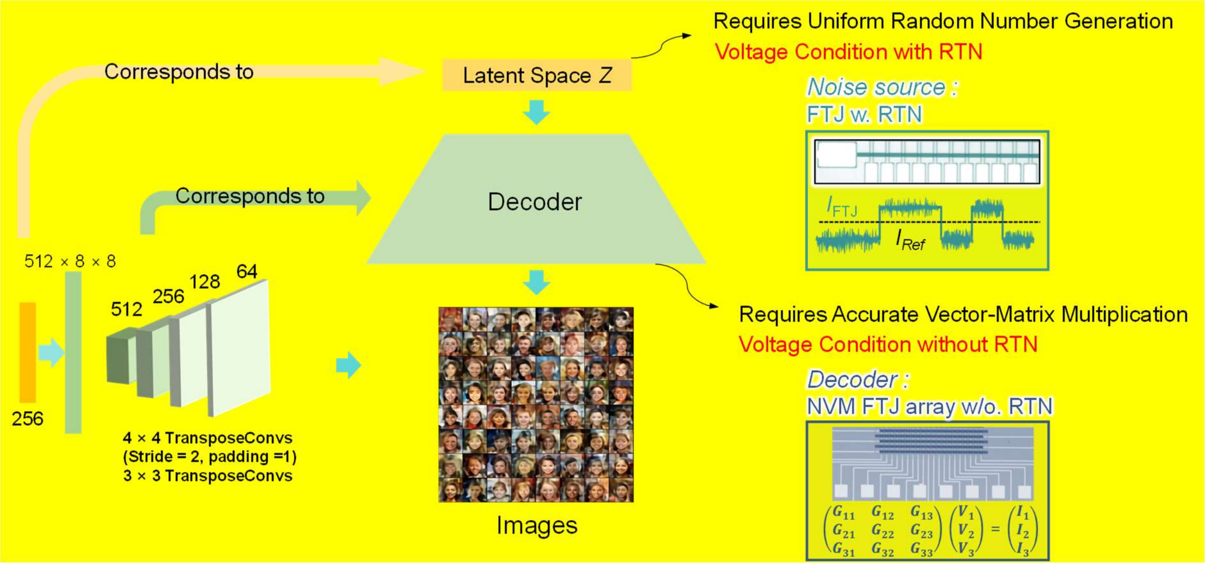

Supplementary Figure 1. VAE image generation flow: latent vector *z* is stochastically sampled (FTJ under RTN for uniform RNG) and fed to a transposed convolution decoder whose deterministic weights are realized by an NVM FTJ crossbar operated without RTN, producing images.

Minor points

- **Ensure consistency of terminology in Table 1 and throughout the text (ReRAM vs RRAM; device categories vs specific materials).**
- **Several typographical issues should be corrected, e.g. "In constrast" → "In contrast" (p.16, line 400).**
- **σ_output N could be better explained in figure captions for readers less familiar with this metric.**

Response:

Thank you for your careful review and constructive suggestions. We have addressed all the minor points as follows.

We ensured consistent terminology throughout the manuscript and Table 1 by standardizing "ReRAM" instead of "RRAM" and unifying device categories and material names. Typographical errors, including the correction of "In constrast" to "In contrast," have been fixed. In addition, we have expanded the figure captions to briefly explain the definition and meaning of σ_{outputN} for readers unfamiliar with this metric.

Please refer to the revised manuscript to find the modification.

Corresponding change in manuscript: Yes

Location of Change: Table 1

									Latent Sampling		Decoder Mapping
Work	Latent Sampling	Decoder Mapping	Device type	CMOS compatibility	Array Size	Task	Implementation	Cell Area	Speed*	Power**	TOPS/W
This work	O	O	Both FTJ	O	400	MNIST, CelebA	Circuit-level	2 × 2 μm^2	36 ms	282 μW (FTJ-only)	106 (FTJ & ADC)
[5]	O	-	Bi_2O_2Se	X	1 (Single Device)	MNIST	Device-level	10 × 10 - 40 × 40 μm^2	10 ms	98.1-337 μW	-
[6]	O	-	ReRAM	X	1 (Single Device)	TRNG-only	Circuit-level	5 × 5 - 50 × 50 μm^2	50-250 ms	1.23- 862 μW	-
[7]	O	-	ReRAM	X	1 (Single Device)	TRNG-only	Circuit-level	2.5 × 2.5 μm^2	100 ms	1.47 mW	-
[8]	-	O	ReRAM	O	Only Simulation	MNIST	Simulation-level	-	-	-	4.6
[9]	-	O	1T-1R	O	1000	MNIST	Circuit-level	-	-	-	-
[10]	O	-	CMOS	O	-	TRNG	Circuit-level	-	11.6-312 ns	38.9-91.6 mW	
[11]	-	O	CMOS	O	-	Neural Networks	Commercial	-	-	-	2.3

*Sampling time per bit

**Normalized for 30 fps Human Face Image (128 ×128) Generation

Tera operations per second (TOPS)

Table 1. Comparison of our work with previous reported hardware generative models.

Location of Change: Results – (page 18)

In contrast, at $V = 3.2$ V, the latent space uniformly includes regions corresponding to all digits from 0 to 9, resulting in a balanced representation of the entire dataset.

Location of Change: Figure 6.

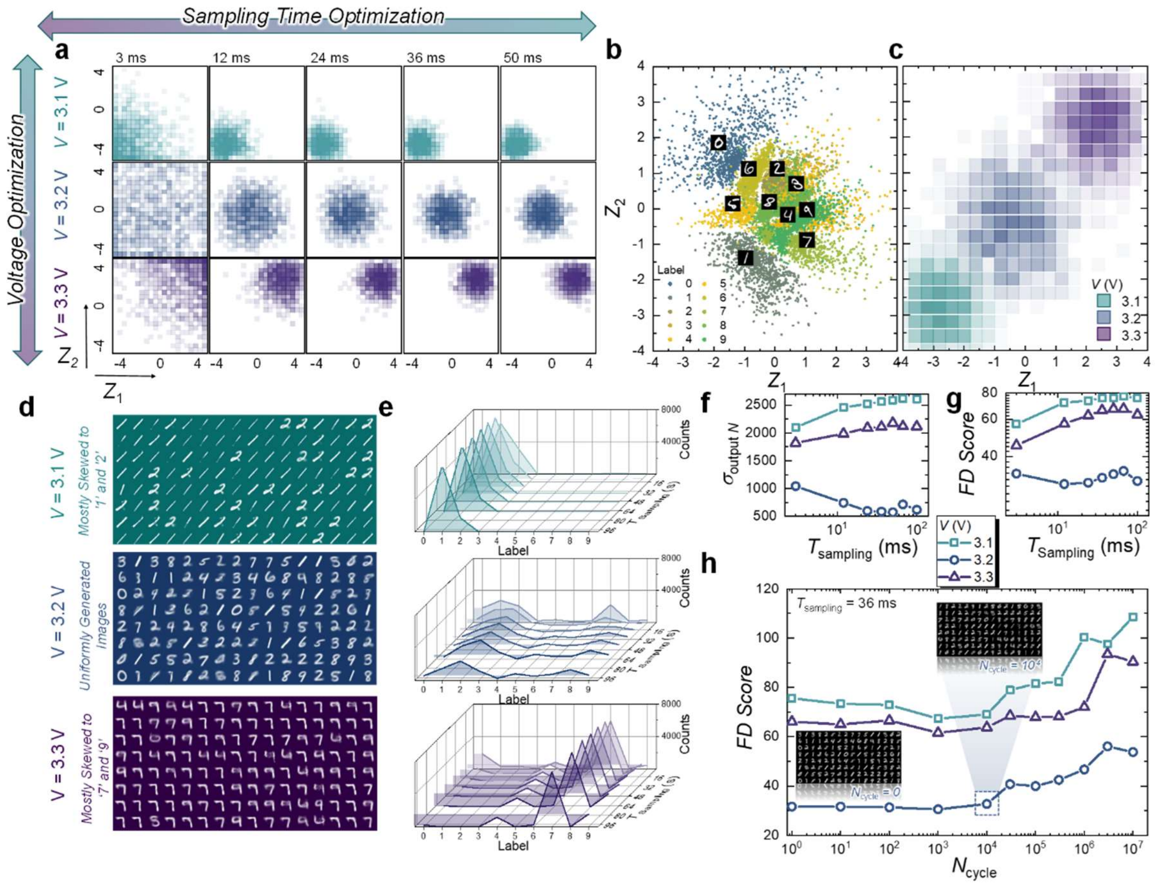

Figure 6. Evaluation of latent space distribution and image generation performance under varying voltage and sampling conditions. a Distribution of generated latent vectors for each V and T_{Sampling}, with biased distributions observed at 3.1 V and 3.3 V. **b** Latent space for the actual MNIST dataset, showing the ideal distribution for accurate image generation. **c** Distribution of random variables in the latent space generated by varying V at fixed $T_{\text{Sampling}} = 36$ ms. **d** Generated MNIST images at different voltages. At $V = 3.1$ V, only digits "1" and "2" are generated, while V =3.2 V produces all digits evenly, and V =3.3 V is biased toward digits "7" and "9." **e** Distribution of generated images by label across different voltages and sampling conditions, with $V = 3.2$ V showing the most uniform distribution. **f** $\sigma_{\text{output N}}$ (standard deviation of images per label), of generated images by label, with $V = 3.2$ V and $T_{\text{Sampling}} = 36$ ms showing the lowest deviation. **g** FD score of generated images, with $V = 3.2$ V and $T_{\text{Sampling}} = 36$ ms presenting the lowest FD score, indicating the highest image diversity. **h** FD score

versus N_{cycle}s, confirming robust FTJ operation in the absence of RTN.

Recommendation

The manuscript is promising and potentially impactful. However, given the missing clarifications on FTJ cell usage, precision, energy/latency trade-offs, and scalability, I recommend a major revision. Addressing these points will strengthen the positioning of this work and ensure it withstands scrutiny from both the materials and systems communities.

Response:

We, the authors, are very much thankful for the detailed comments and instructive suggestions. We have revised the manuscript in light of the useful comments and suggestions of the reviewer. Each comment and suggestion has been followed and addressed in the response attached.

We, the authors, send the deepest appreciation for the encouragement and comments again.

Revision and Correction (NCOMMS- 25-67053B)

Dear Reviewers,

We, the authors, appreciate the time and effort that the reviewers dedicated to providing feedback on our manuscript and are grateful for the insightful comments on our work. We read the comments and questions carefully and prepared a response sheet for them. Please refer to the responses below. The responses that need to be addressed in the revised manuscript are highlighted so that the reviewers can trace them with more ease. Also, the overall sentences have been refined with more concise expressions and better English grammar. We heartily thank all the comments and coordination again.

Reviewer #1

The authors have made a substantial effort to address the initial concerns. Several issues should be addressed to fully meet the journal's standards：

Response:

We, the authors, are very much thankful for the detailed comments and instructive suggestions. We have revised the manuscript in light of the useful comments and suggestions of the reviewer. Each comment and suggestion has been followed and addressed in the response attached below.

We, the authors, send the deepest appreciation for the encouragement and comments again.

1. Please translate the 1 mV per-cell trimming scheme into hardware cost. If each column needs a ≥10-bit DAC, the area could easily outweigh the FTJ array itself.

Response:

Thank you for your comment. We agree that it is important to clarify the circuit-level overhead implied by the 1 mV-step trimming.

In our work, the "1 mV trimming" refers to the calibration step size used to slightly shift the RTN read bias so that the RTN occupancy becomes balanced (τ_L/τ_H is equalized) across devices, rather than implying a large full-scale tuning range. Supplementary Fig. 16 shows histograms of the low- and high-state dwell times (τ_L and τ_H) for multiple devices, where each device is biased at its own equalized read voltage. Importantly, the optimized voltages lie in a narrow window of 3.25–3.30 V. Supplementary Fig. 17 summarizes the corresponding optimized read voltages and extracted dwell-time constants. The key implication is that the required tuning range is only on the order of tens of millivolts, not volts, and thus does not require ≥10-bit resolution. For example, a 6-bit trim over a 50 mV window provides an LSB of ~0.78 mV, already comparable to the 1 mV calibration step, while a 7-bit trim would provide additional margin if desired. Therefore, the trimming hardware requirement is closer to a compact 6–7-bit fine-trim bias DAC (or equivalently, a digitally trimmed limited-range bias generator around the RTN operating point) rather than a high-resolution wide-range DAC.

In addition, this fine trimming is required only for the RTN-based sampler and not for the entire FTJ array used for deterministic VMM. As described in the manuscript, we partition the operation such that a small sampler region uses the RTN window near ~3.2 V. In comparison, the rest of the array executes deterministic VMM at 2 V in the RTN-free regime. Therefore, the trim circuitry scales with the sampler lines rather than with all columns of the compute array. Moreover, because the trim code is static after calibration, the trim can be implemented efficiently (compact monotonic small-range trim DAC, or a shared DAC with multiplexing/sample-and-hold plus per-line digital code storage), avoiding the need to dedicate a large DAC to every column in a fully parallel manner. As one concrete feasibility point, prior work reports a low-area monotonic 6-bit trim DAC in 40-nm CMOS with an area of 8 µm × 18.5 µm per DAC [R1], illustrating that a fine-trim block in this resolution range can be compact. Finally, we note that our devices were fabricated in an academic environment. Further process optimization in an industrial flow would be expected to reduce device-to-device bias variation, which would relax the required trim range and trim-resolution/area overhead.

Finally, we note that per-cell trimming is not mandatory for this system: even when using a single fixed read voltage (i.e., without trimming), the measured dwell times τ_H and τ_L are already comparable for most devices. The 1 mV-step bias adjustment introduced in the previous revision round was provided as an additional design guideline to achieve an ideal ~50% RTN occupancy, rather than a required condition for correct operation (Supplementary Figs. 16 and 17).

We thank the reviewer for raising this important point, as it prompted us to explicitly translate the trimming scheme into a realistic circuit requirement (limited range and moderate resolution) and to clarify that a fixed-bias operation is feasible, thereby strengthening the practicality and hardware-level positioning of our system.

Following the reviewer's suggestion, we have added a clarifying discussion in the revised manuscript that (i) the measured equalization range is limited to ~3.25–3.30 V (tens of mV) and therefore does not require ≥10-bit resolution, and (ii) the trimming applies only to the RTN sampler region and can be implemented using compact fine-trim bias circuitry. Please refer to the revised manuscript to find the modifications.

[R1] A. Drozd, R. Szczygiel, P. Maj, T. Satlawa, P. Grybos, Design of the low area monolithic trim DAC in 40 nm CMOS technology for pixel readout chips, Journal of Instrumentation 9, C12046 (2014).

Location of Change: Results – Page 13

Notably, the equalized read voltages fall within a narrow 3.25–3.30 V window (Supplementary Fig. 16,17), implying that the required tuning range is only on the order of tens of millivolts. Therefore, a compact limited-range 6–7-bit fine-trim bias DAC (≈1 mV LSB) is sufficient, and this trimming is required only for the RTN sampler region rather than the full VMM array.

2. Fig. 3h only verifies binary (LRS/HRS) linearity. To justify "4-bit (16-state)" weights, please report the programmed 16 uniformly spaced conductance levels, and discuss how this quantization noise affects CelebA FID compared to ideal 8-bit weights.

Response:

Thank you for your comment. We agree that it is important to experimentally substantiate the multi-level conductance programmability used for the decoder weights and to quantify the impact of the resulting quantization/variation on CelebA generation quality.

As the reviewer pointed out, Fig. 3h in the original manuscript shows the column-summed current measured at a fixed read bias (2 V, RTN-free regime) as a function of the number of activated devices, where each cell is programmed either to LRS or HRS. This result verifies reliable current accumulation in the deterministic operating regime. It confirms that the summed current scales linearly with the number of contributing devices, which is essential for VMM linearity. However, this binary accumulation test does not demonstrate whether intermediate conductance levels can be programmed with sufficient statistical separation across the measured devices. To answer the reviewer's request, we have performed additional measurements on multi-level programming across 50 different FTJ devices.

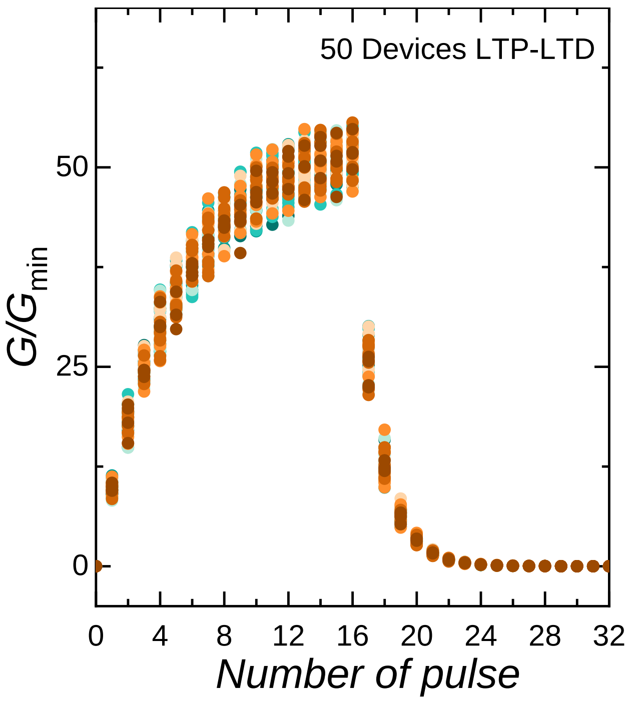

Fig. R1. Pulse number versus normalized conductance for 50 FTJ devices under LTP/LTD programming, showing device-to-device variability in multi-level weight updates.

Fig. R1 shows the measured long-term potentiation/depression (LTP/LTD) characteristics across 50 devices, plotted as normalized versus the number of applied programming pulses. The pulse conditions are identical to

those used in Fig. 3g (LTP: consecutive +5 V, 10 μs pulses; LTD: consecutive −2 V, 10 μs pulses). This figure confirms that the FTJ conductance can be gradually and monotonically modulated over a broad dynamic range through incremental ferroelectric switching, providing the physical basis for multi-level weight programming beyond binary LRS/HRS.

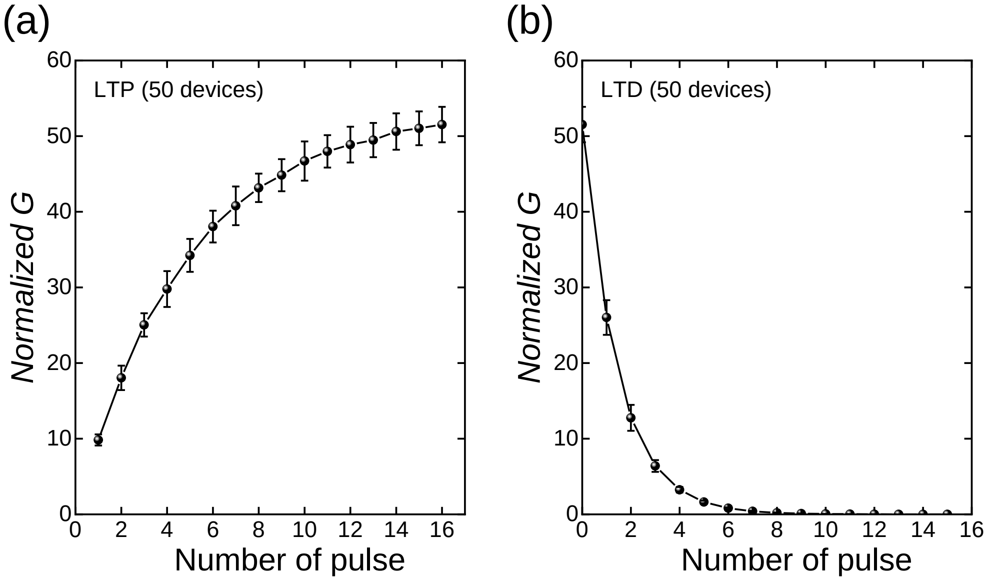

Fig. R2. Averaged (a) LTP and (b) LTD trajectories across 50 devices with standard-deviation error bars.

Fig. R2 shows the mean LTP and LTD curves across 50 devices using symbols connected by lines. Error bars indicate the standard deviation at each pulse index. These statistics confirm gradual conductance modulation suitable for multi-level operation. However, the 16 levels are not equally separable across the full programming range. In particular, in both LTP and LTD, the early pulse steps show clearer separation. By contrast, as programming approaches saturation, the distributions broaden, and adjacent levels become harder to distinguish. The mean conductance still changes gradually, but the individual levels are not perfectly separated.

This non-uniform spacing is already reflected in our device-informed 4-bit mapping. In the original analysis, the 16 targets were derived from the measured LTP and LTD characteristics rather than being placed uniformly, so the update-curve nonlinearity was accounted for. However, the reviewer is correct that the experimentally measured variation around each target level was not included. In this revision, we incorporate the measured level-to-level variation using the spreads extracted from Fig. R2.

Regarding the impact of quantization noise (or variation) on CelebA generation quality, it is well known that aggressive weight quantization and device non-idealities can degrade neural network performance when the induced weight/read error exceeds the model's tolerance margin [R2]. At the same time, compute-in-memory literature consistently reports that neural networks can be robust to low-bit quantization and moderate device noise when the quantization strategy and operating conditions are properly optimized [R3]. Following the reviewer's suggestion, we extended our CelebA decoder simulations to incorporate the measured multi-level non-idealities

explicitly. Fig. R3 compares the CelebA FID under the optimized latent sampling condition (V = 3.2 V and $T_{sampling}$ = 36 ms) for three decoder-weight settings: (i) an ideal 8-bit baseline, (ii) a device-informed 4-bit quantization that reflects the measured LTP/LTD nonlinearity, and (iii) the same 4-bit mapping with additional conductance variation injected according to the measured spreads in Fig. R2, which corresponds to the reviewer's notion of quantization noise. The FID is 210.4 for the ideal 8-bit case, 218.3 when only the device-informed nonlinear 4-bit mapping is applied, and 227.3 when both the nonlinear mapping and the measured variation are included. This negligible change indicates that, under the optimized latent sampling condition, decoder performance is relatively tolerant to the tested level of 4-bit quantization and conductance variation, consistent with prior compute-in-memory reports that low-bit weight quantization and moderate device noise can be tolerated when the mapping and operating conditions are properly optimized [R2, R3].

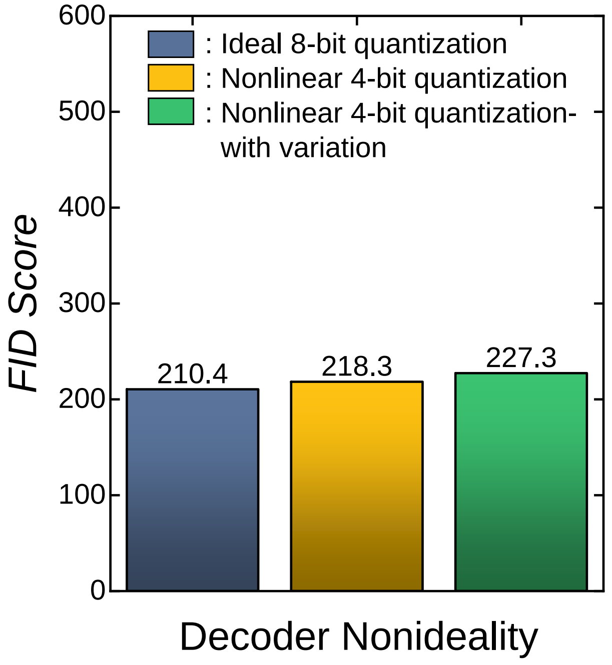

Fig. R3. CelebA FID under decoder weight non-idealities at the optimized sampling condition. CelebA FID comparison at V = 3.2 V and $T_{sampling}$ = 36 ms for three decoder-weight settings: ideal 8-bit weights, device-informed 4-bit mapping reflecting the measured LTP/LTD nonlinearity, and the same 4-bit mapping with additional conductance variation injected according to the measured spreads in Fig. R2.

Finally, to clarify what dominates the overall CelebA quality, Fig. R4 shows that even with ideal 8-bit weights, the FID is highly sensitive to the latent sampling regime. Under the same ideal 8-bit weights, the FID is 210.4 in region 2, but it degrades to 456.6 in region 1 and 375.1 in region 3. These results confirm that, in our current framework, the primary bottleneck is the latent sampling distribution governed by RTN statistics. At the same time, the decoder is comparatively robust to the tested weight quantization and variation. We thank the reviewer for raising this important point, and we will continue to improve decoder accuracy and robustness through tighter multi-level programming control and variability reduction in future work.

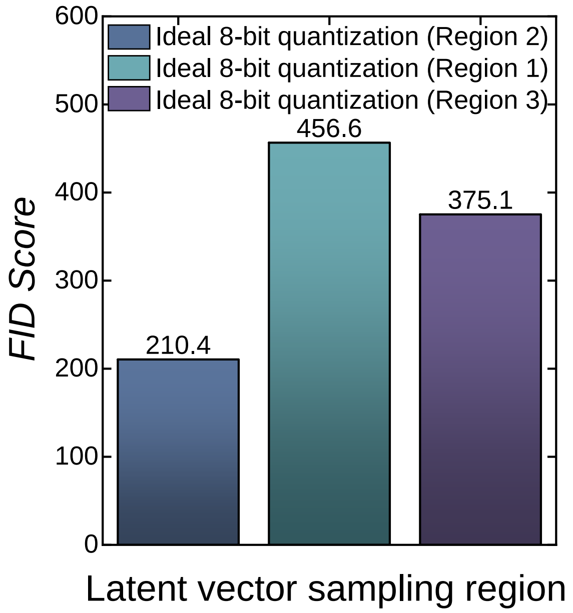

Fig. R4. CelebA FID sensitivity to latent sampling regime with ideal decoder weights. CelebA FID evaluated using ideal 8-bit decoder weights across three latent sampling regimes (regions 1–3). The plot highlights that FID is strongly dependent on the RTN-governed sampling condition and is minimized at the optimized regime (region 2).

In addition, we revised the wording in the manuscript to avoid implying 16 perfectly and uniformly separable physical conductance states. Instead of describing the decoder as using "4-bit (16-state) conductance states," we now describe it as a device informed 4-bit mapping with 16 discrete target bins derived from the measured potentiation and depression characteristics and their statistical spreads. Following the reviewer's comment, we have added the multi-level programming statistics across 50 devices (Figs. R1 and R2), the CelebA FID comparison between ideal 8-bit and device-informed 4-bit weights, including measured quantization variation (Fig. R3), and the additional discussion highlighting the relative sensitivity to sampling conditions (Fig. R4). Please refer to the revised manuscript for the corresponding modifications.

[R2] X. Peng, S. Huang, Y. Luo, X. Sun, S. Yu, DNN+NeuroSim: An end-to-end benchmarking framework for compute-in-memory accelerators with versatile device technologies. IEEE International Electron Devices Meeting (IEDM), 771-774 (2019).

[R3] Y. Kim, H. Kim, J. Park, H. Oh, J. -J. Kim. Mapping binary ResNets on computing-in-memory hardware with low-bit ADCs. Design Automation and Test in Europe (DATE), 856-861 (2021).

Corresponding change in manuscript: Yes

Location of Change: Results – Page 8

The update curves exhibit nonlinearity with β_{LTP} = 4.9 and β_{LTD} = 8.2. Based on these measured nonlinearities, the decoder uses a device-informed 4-bit (16-state) conductance partition, and weight mapping assigns targets according to the β-dependent update characteristics. Results of 50 consecutive LTP/LTD cycles are shown in Supplementary Fig. 5. Supplementary Fig. 6 shows the mean LTP and LTD trajectories across 50 devices as lines, with standard-deviation error bars at each pulse index. In the subsequent simulations, the decoder weights reflect both effects. The nonlinearity is incorporated through the β-based 16-bin target mapping, and the level-to-level variation is injected using the measured spreads from Supplementary Fig. 6.

Location of Change: Supplementary Figure 6

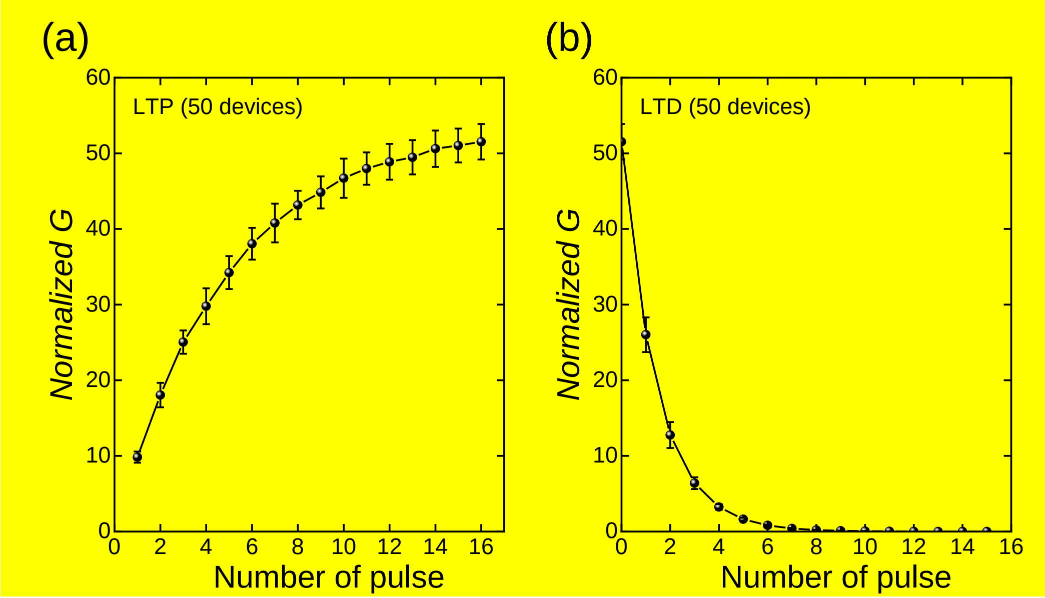

Fig. S6. Averaged (a) LTP and (b) LTD trajectories across 50 devices with standard-deviation error bars.

Location of Change: Results – Page 21

Supplementary Note 7 describes how non-idealities affect the CelebA FID. In particular, it reports the impact of the measured update-curve nonlinearity and the level-to-level variation on the FID in our device-informed simulations.

Location of Change: Supplementary Note 7. Impact of Weight Non-Idealities on CelebA FID

Supplementary Note 7. Impact of Weight Non-Idealities on CelebA FID

Aggressive weight quantization and device non-idealities can degrade neural network performance when the induced weight or read error exceeds the model tolerance. At the same time, compute-in-memory studies have shown that neural networks can remain robust to low-bit quantization and moderate device noise when the

quantization strategy and operating conditions are properly optimized. Motivated by these observations, we extended our CelebA decoder simulations to explicitly incorporate the measured multi-level non-idealities.

Fig. SN7-1 compares the CelebA FID under the optimized latent sampling condition (V = 3.2 V and $T_{sampling}$ = 36 ms) for three decoder-weight settings: (i) an ideal 8-bit baseline, (ii) a device-informed 4-bit quantization that reflects the measured LTP/LTD nonlinearity, and (iii) the same 4-bit mapping with additional conductance variation injected according to the measured spreads in Fig. R2, which corresponds to the reviewer's notion of quantization noise. The FID is 210.4 for the ideal 8-bit case, 218.3 when only the device-informed nonlinear 4-bit mapping is applied, and 227.3 when both the nonlinear mapping and the measured variation are included. This negligible change indicates that, under the optimized latent sampling condition, decoder performance is relatively tolerant to the tested level of 4-bit quantization and conductance variation, consistent with prior compute-in-memory reports that low-bit weight quantization and moderate device noise can be tolerated when the mapping and operating conditions are properly optimized

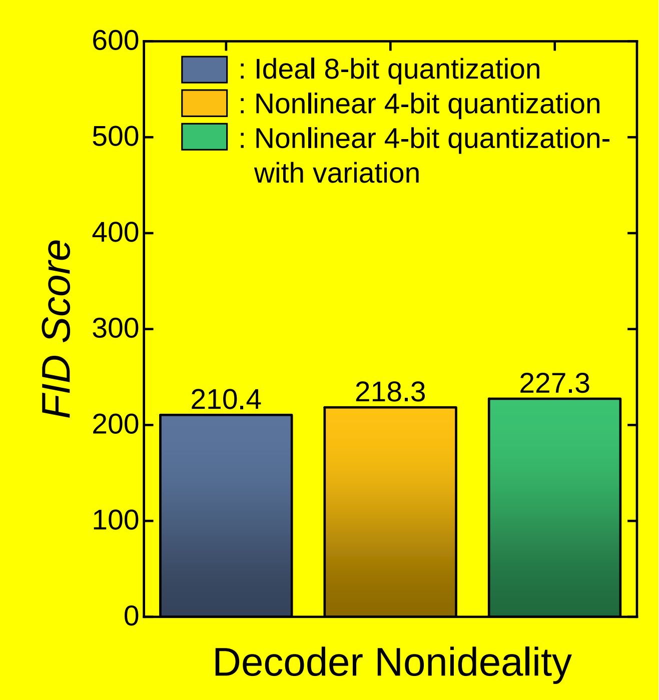

Fig. SN7-1. CelebA FID under decoder weight non-idealities at the optimized sampling condition. CelebA FID comparison at V = 3.2 V and $T_{sampling}$ = 36 ms for three decoder-weight settings: ideal 8-bit weights, device-informed 4-bit mapping reflecting the measured LTP/LTD nonlinearity, and the same 4-bit mapping with additional conductance variation injected according to the measured spreads in Fig. S6.

To clarify what dominates the quality metric, Fig.SN 7-2 shows that even with ideal 8-bit weights, the FID is highly sensitive to the latent sampling regime. Under the same ideal 8-bit weights, the FID is 210.4 in region 2, but it degrades to 456.6 in region 1 and 375.1 in region 3. These results confirm that, in our current framework, the primary bottleneck is the latent sampling distribution governed by RTN statistics. At the same time, the decoder is comparatively robust to the tested weight quantization and variation. We thank the reviewer for raising

this important point, and we will continue to improve decoder accuracy and robustness through tighter multi-level programming control and variability reduction in future work.

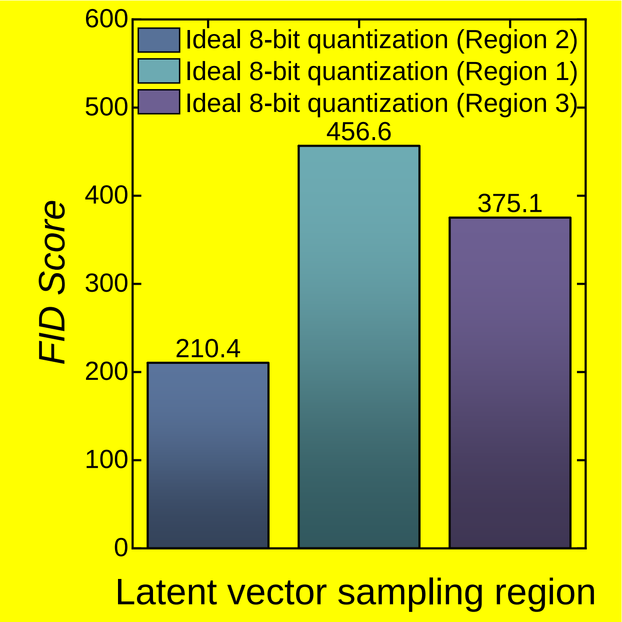

Fig. SN7-2. CelebA FID sensitivity to latent sampling regime with ideal decoder weights. CelebA FID evaluated using ideal 8-bit decoder weights across three latent sampling regimes (regions 1–3). The plot highlights that FID is strongly dependent on the RTN-governed sampling condition and is minimized at the optimized regime (region 2).

3. Please clarify whether "reading pulses cause fatigue" or only "writing pulses are counted as 10^5". If the endurance of reading pulses is not limited, please provide the read disturbance experiment data.

Response:

Thank you for your comment. We agree that some parts of our text and figure captions were written in a way that could confuse readers about whether the cycle count refers to repeated read pulses or to write program and erase cycling, and this should be clarified.

In our manuscript, N_{cycle} refers to write cycling stress, not read operations. One write cycle is defined as one program pulse of +5 V with 10 μs width followed by one erase pulse of -2 V with 10 μs width, as defined in Fig. 3g. After applying a given N_{cycle}, we evaluate image generation performance using the read current distributions measured afterward under the deterministic VMM read condition (Fig. 6h and Fig. 6i). The increased overlap between HRS and LRS at higher N_{cycle} reflects the gradual degradation of TER caused by write cycling, which explains the slight degradation in image quality metric at large cycle counts. The same write cycling definition is used for the CelebA endurance evaluation in Fig. 7f. We have corrected this to "write cycles" and clarified the definition of N_{cycle} to avoid any ambiguity.

Regarding read cycle endurance, the decoder VMM in our system is performed at a low read bias of 2 V with a 10 ms read pulse, which is substantially lower than the write voltages (5 V) used for polarization switching. Therefore, we do not expect the read operation to cause measurable fatigue [R4]. To validate this experimentally, we performed an additional read disturbance test using repeated read pulses under the same read conditions. Fig. R5 shows the HRS and LRS conductance as a function of the number of read pulses ($N_{read\ cycle}$) up to 10^7, and both conductance levels remain essentially unchanged throughout the test. The TER ratio was 113 before applying the read pulses and 116 after 10^7 read operations, confirming that repeated read operations do not measurably disturb the stored states within the tested range.

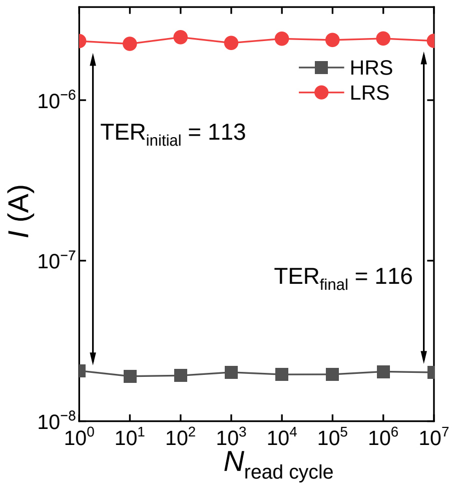

Fig. R5. Read pulse endurance test. HRS/LRS read current evolution versus the $N_{read\ cycle}$ (V_{read} = 2 V, 10 ms), demonstrating negligible read-induced fatigue.

Following the reviewer's suggestion, we have revised the manuscript to state clearly that N_{cycle} denotes the number of write program and erase cycles, and we separated the read cycle endurance evaluation using a dedicated read pulse count ($N_{read\ cycle}$). We also added the read endurance data in Fig. R5 and the corresponding discussion. Please refer to the revised manuscript to find the modifications.

[R4] V. Garcia and M. Bibes, "Ferroelectric tunnel junctions for information storage and processing," Nature Communications 5, 4289 (2014).

Corresponding change in manuscript: Yes

Location of Change: Results – Page 19

To account for this effect, the changes in the on/off ratio as a function of the number of write cycle stress (N_{cycle}) are incorporated into the simulation.

Location of Change: Results – Page 19

This result confirms the robustness and reliability of the system under repeated operation. Note that the read voltage of 2 V is much lower than the 5 V write voltage and therefore introduces negligible electrical stress. The TER ratio as a function of the number of applied read pulses ($N_{read\ cycle}$) under the read condition of 2 V and 1 ms is shown in Fig. S28, and it remains unchanged up to 10^7 cycles.

Location of Change: Supplemetary Figure S28.

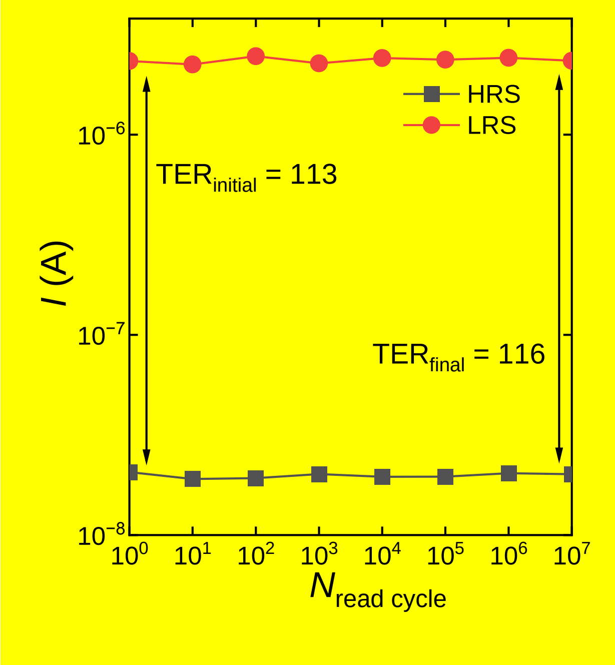

Fig. S28. Read pulse endurance test. HRS/LRS read current evolution versus the $N_{read\ cycle}$ (V_{read} = 2 V, 10 ms), demonstrating negligible read-induced fatigue.

Location of Change: Results – Page 21

Figure 7(f) presents the FID score as a function of the N_{cycle} applied to the FTJ in the decoder, showing that stable image generation is maintained up to 10^5 write cycles.

4. Please use the three-dimensional indicators of area-energy-random quality to directly compare the superiority coefficients of the FTJ scheme with digital circuits.

Response:

Thank you for your comment. We agree that it is important to provide a direct, quantitative comparison against digital-circuit implementations using simultaneous indicators of area, energy/power cost, and random quality.

To address this request, we introduced a unified "Area-Power-Randomness Cost (APRC)" that consolidates the three-dimensional indicators into a single figure of merit. We define the APRC as :

$$APRC = \frac{A \times P}{\eta_{\mathrm{NIST}}}$$

A is the area (μm^2) of the stochastic sampling macro (defined below for each comparison group), P is the power consumption during stochastic operation (in μW), and η_{NIST} is the NIST SP 800-22 test pass ratio used as a quantitative metric for random quality. With this definition, a smaller APRC indicates a more favorable overall trade-off because it corresponds to a smaller footprint, lower power, and higher randomness quality.

Importantly, when defining A and P for comparison with prior work, we separate the literature into two groups because the reported information differs systematically. CMOS-based TRNG and random-generator studies typically provide a complete circuit implementation, so it is meaningful to compare at the macro level, including the peripheral circuits required to output digital bits. In our manuscript, this digital-circuit comparison corresponds to Refs. 10 and 11. For this group, we ensure a fair comparison by explicitly accounting for the peripheral overhead required for stochastic sampling. The area includes the DAC area and the sampling sense-amplifier area, both estimated under a 40 nm CMOS technology, and the corresponding power consumption is included in the same manner.

In contrast, many device-noise-based memory studies report only the device or cell footprint and the device-level stochastic power without providing a complete sampling and digitization circuit scheme. A macro-level area comparison in that case would require arbitrary assumptions about unreported overhead. In our manuscript, this memory-based comparison corresponds to Refs. 6 and 7. We therefore report a macro-level APRC for the digital-circuit group and a cell-level APRC for the memory group to keep the comparison fair.

Fig. R6 summarizes the comparison as two three-dimensional scatter plots with area, power, and NIST pass ratio as the axes. Fig. R6 (a) compares our FTJ scheme with the CMOS circuit implementations in Refs. 10 and 11 using macro-level A and P that include peripheral overhead in both cases. Fig. R6 (b) compares our FTJ scheme with the device-noise-based memory implementations in Refs. 6 and 7 using cell-level A and the reported stochastic-operation P, since circuit overhead is not consistently reported for those works. The CMOS macro comparison uses A = 160 μm^2, P = 282 μW, and η_{NIST} = 0.875 for our work, while Ref. 10 reports A = 10,000 μm^2, P = 523 μW, and η_{NIST} = 1 and Ref. 11 reports A = 2,300 μm^2, P = 1,200 μW, and η_{NIST} = 1. The memory cell comparison uses A = 4 μm^2, P = 282 μW, and η_{NIST} = 0.875 for our work, while Ref. 6 reports A = 2,500 μm^2, P = 862 μW, and η_{NIST} = 0.82 and Ref. 7 reports A = 6.25 μm^2, P = 1,470 μW, and η_{NIST} = 1. In both cases, the desirable region is defined by low area, low power, and high NIST pass ratio, and our data point lies closest to

this desired zone.

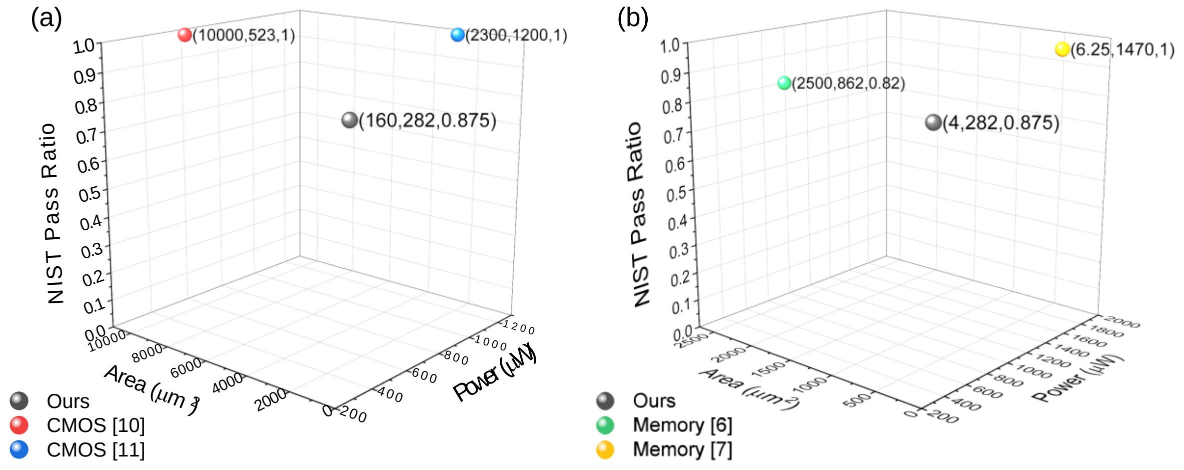

Fig. R6. Three-dimensional area–power–randomness comparison using area as x axis, power as y axis, and NIST pass ratio as z axis. (a) 3D scatter plot comparing the proposed FTJ sampler with CMOS-based implementations (Refs. 10 and 11) using macro-level area and power that include peripheral overhead. (b) 3D scatter plot comparing cell-level sampling metrics of the proposed FTJ sampler with device-noise-based memory works (Refs. 6 and 7), using reported cell footprint and stochastic-operation power.

Fig. R7 summarizes the same comparison using APRC for easier numerical comparison. For the CMOS comparison group, our APRC is 51,566 μm^2·μW, while Refs. 10 and 11 yield 5,230,000 μm^2·μW and 2,760,000 μm^2·μW, respectively. For the memory comparison group, our APRC is 1,289 μm^2·μW, while Refs. 6 and 7 yield 2,628,049 μm^2·μW and 9,188 μm^2·μW, respectively. In both the macro-level CMOS comparison and the cell-level memory comparison, the proposed FTJ scheme achieves the lowest APRC, confirming its superior combined trade-off of compactness, power efficiency, and randomness quality under the reporting constraints of the prior art.

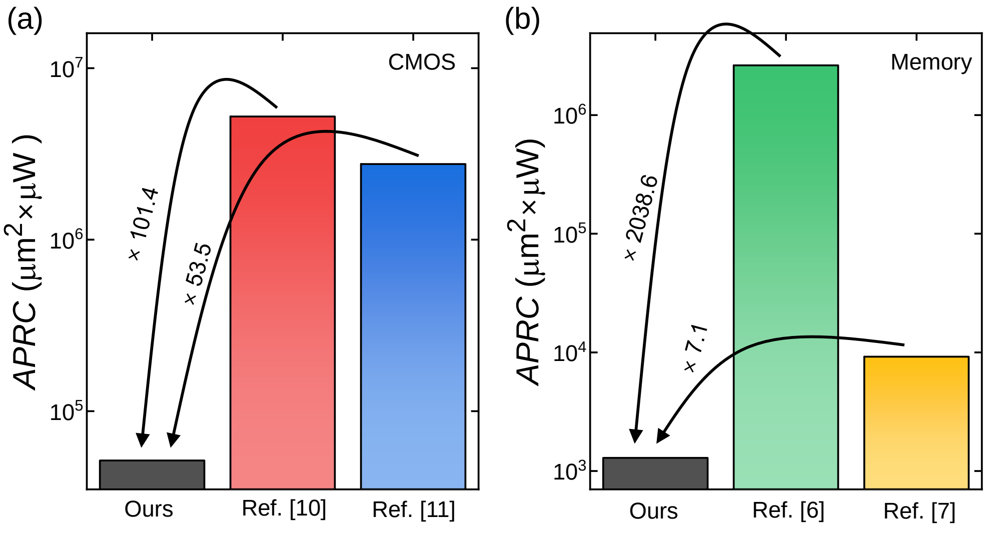

Fig. R7. Area–Power–Randomness Cost (APRC) comparison. (a) APRC box plots for the proposed work and CMOS-based implementations. (b) APRC box plots for the proposed work and device-noise-based memory implementations.

Beyond these numerical indicators, we also emphasize an architectural advantage that is not fully captured by the *APRC* metric. The same CMOS-compatible hafnia FTJ technology supports both stochastic latent sampling through voltage-tunable RTN and deterministic VMM decoding through nonvolatile conductance states within a single device platform. This single-device dual-mode capability reduces the need for heterogeneous hardware blocks and simplifies integration compared to approaches that require separate devices or separate stochastic modules.

Following the reviewer's suggestion, we have added the definition of *APRC*, clarified the area-definition methodology and the reason for separating the CMOS and memory comparison groups, and provided both the three-dimensional plots and the consolidated *APRC* comparison in the revised manuscript. Please refer to the revised manuscript to find the modifications.

Corresponding change in manuscript: Yes

Location of Change: Results – Page 22

Finally, we define the Area–Power–Randomness Cost (APRC) as $APRC = (A \times P) / \eta_{NIST}$, where A is the sampling-hardware area, P is the power during stochastic operation, and η_{NIST} is the NIST SP 800-22 pass ratio. A smaller *APRC* indicates a better trade-off among area, power, and random quality. Fig. S35 visualizes the area–power–randomness comparison in a 3D space, and Fig. S36 summarizes the same results using *APRC* for direct numerical comparison. The detailed definitions of A and P and the fair-comparison methodology are provided in Supplementary Note 9.

Location of Change: Supplementary Figure 35.

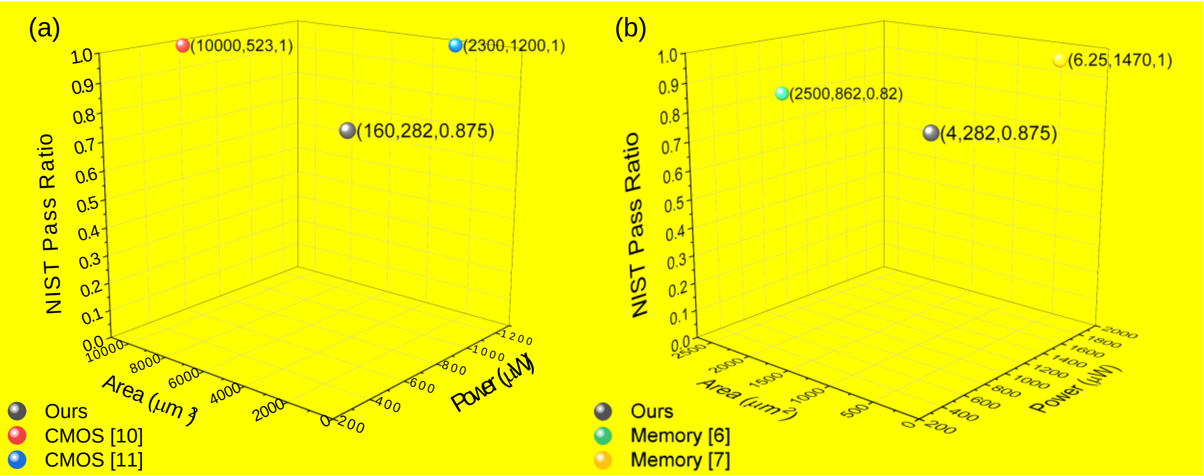

Fig. S35. Three-dimensional area–power–randomness comparison using area as *x* axis, power as *y* axis, and NIST pass ratio as *z* axis. (a) 3D scatter plot comparing the proposed FTJ sampler with CMOS-based implementations (Refs. 10 and 11) using macro-level area and power that include peripheral overhead. (b) 3D scatter plot comparing cell-level sampling metrics of the proposed FTJ sampler with device-noise-based memory works (Refs. 6 and 7), using reported cell footprint and stochastic-operation power.

Location of Change: Supplementary Figure 36.

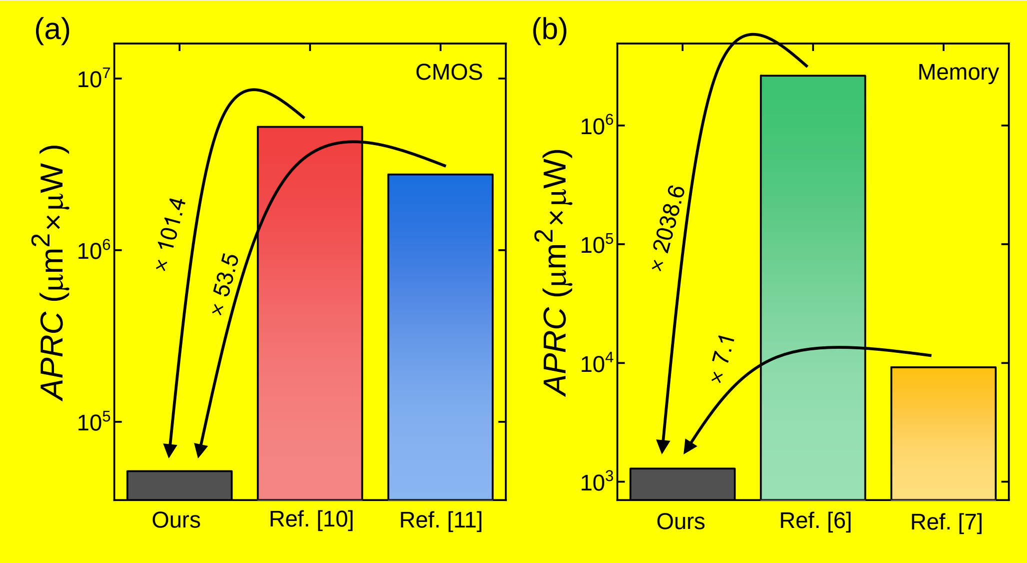

Fig. S36. Area–Power–Randomness Cost (APRC) comparison. (a) APRC box plots for the proposed work and CMOS-based implementations. (b) APRC box plots for the proposed work and device-noise-based memory implementations.

Location of Change: Supplementary Note 9. Definition of APRC and Fair Comparison Methodology

To compare stochastic sampling implementations using area, power, and random quality in a single metric, we define the Area–Power–Randomness Cost as $APRC = (A \times P) / \eta_{\mathrm{NIST}}$. Here A denotes the area of the stochastic sampling hardware, P denotes the power consumption during stochastic operation, and η_{NIST} denotes the NIST SP 800-22 test pass ratio used as a quantitative measure of random quality. With this definition, a smaller APRC indicates a more favorable trade-off because it simultaneously reflects a smaller footprint, lower power, and higher randomness quality.

A practical issue in benchmarking is that prior studies do not report area and power at a uniform level of completeness. CMOS TRNG and random-generator works typically provide a complete circuit implementation, so a macro-level comparison is meaningful because the reported area and power include the peripheral circuitry required to produce a digital random bitstream. In our manuscript, this digital-circuit comparison corresponds to Refs. 10 and 11. By contrast, many device-noise-based memory works report only the device or cell footprint and the device-level power during stochastic operation, while omitting a complete sampling and digitization circuit scheme. In such cases, a macro-level area comparison would require arbitrary assumptions about unreported

overhead, which would reduce transparency. In our manuscript, this memory-based comparison corresponds to Refs. 6 and 7. We therefore separate the comparison into two groups and report *APRC* at the macro level for the CMOS group and at the cell level for the memory group. This separation is intended to keep the comparison fair and technically consistent given what each class of work reports.

For the digital-circuit group, we explicitly include the peripheral overhead required for stochastic sampling in our FTJ system so that the comparison is performed at the same macro level as CMOS implementations. Specifically, the area A includes the DAC area and the sampling sense-amplifier area, both estimated under a 40 nm CMOS technology, and the corresponding power consumption P is included in the same manner. Using this macro definition, our FTJ scheme uses A = 160 μm^2, P = 282 μW, and η_{NIST} = 0.875. Ref. 10 reports A = 10,000 μm^2, P = 523 μW, and η_{NIST} = 1, while Ref. 11 reports A = 2,300 μm^2, P = 1,200 μW, and η_{NIST} = 1. The resulting *APRC* values are 51,566 μm^2·μW for this work, 5,230,000 μm^2·μW for Ref. 10, and 2,760,000 μm^2·μW for Ref. 11.

For the memory-based group, we use cell-level area and the reported stochastic-operation power because circuit overhead is not consistently reported in the literature. Under this cell-level definition, our FTJ cell uses A = 4 μm^2 with P = 282 μW and η_{NIST} = 0.875. Ref. 6 reports A = 2,500 μm^2, P = 862 μW, and η_{NIST} = 0.82, while Ref. 7 reports A = 6.25 μm^2, P = 1,470 μW, and η_{NIST} = 1. The resulting *APRC* values are 1,289 μm^2·μW for this work, 2,628,049 μm^2·μW for Ref. 6, and 9,188 μm^2·μW for Ref. 7. Ref. 5 is excluded from the APRC comparison because η_{NIST} is not reported.

Fig. S35 visualizes these comparisons as three-dimensional scatter plots with A, P, and η_{NIST} as the axes, where the desirable region corresponds to low area, low power, and high η_{NIST}. Fig. S36 summarizes the same results using *APRC* for direct numerical comparison within each reporting-consistent group.

We, the authors, send the deepest appreciation for the encouragement and comments again.

Reviewer #2

All my comments have been addressed, and the revision looks good to me. I do not have further comments on the revised manuscript.

Response:

We, the authors, are very much thankful for the detailed comments and instructive suggestions. We have revised the manuscript in light of the useful comments and suggestions of the reviewer. Each comment and suggestion has been followed and addressed in the response attached below.

We, the authors, send the deepest appreciation for the encouragement and comments again.

Reviewer #3

I co-reviewed this manuscript with one of the reviewers who provided the listed reports. This is part of the Nature Communications initiative to facilitate training in peer review and to provide appropriate recognition for Early Career Researchers who co-review manuscripts.

Response:

We, the authors, are very much thankful for the detailed comments and instructive suggestions. We have revised the manuscript in light of the useful comments and suggestions of the reviewer. Each comment and suggestion has been followed and addressed in the response attached.

We, the authors, send the deepest appreciation for the encouragement and comments again.

Reviewer #4

Thanks for the revision. In this round, the authors have provided an extensive and high-quality revision that addresses nearly all major concerns raised in the first round. The manuscript is technically strengthened, better organized, and much clearer in presentation.

Summary of improvements

- The distinction between stochastic and deterministic operation of the FTJ array is now explicit: a small region operates in the RTN window (~3.2 V) for stochastic sampling, while the remaining majority performs deterministic VMM decoding at 2 V without weight reprogramming.

- Deterministic precision is quantified at an effective 4-bit (16 state) resolution per FTJ, extended to 64 levels per differential pair, supported by measured LTP/LTD parameters.

- Wafer-scale ALD uniformity, per-device bias trimming, endurance, and IR-drop tolerance are demonstrated through new experimental data (Figs. R1–R5, R9).

- Throughput, area, and energy efficiency are analyzed quantitatively in Supplementary Note 7, while Table 1 has been expanded to benchmark FTJ performance against CMOS and ReRAM implementations. Figures have been reorganized, and a clear flow diagram has been added for system readability.

Response:

We, the authors, are very much thankful for the detailed comments and instructive suggestions. We have revised the manuscript in light of the useful comments and suggestions of the reviewer. Each comment and suggestion has been followed and addressed in the response attached.

Minor residual points

1. The 36 ms sampling latency and associated hardware overhead (~4,552 FTJs for 30 fps) are discussed only in Supplementary Note 7. I suggest briefly acknowledging this trade-off in the main Discussion section.

2. The comparison works in updated Table 1 (Refs. 5-11) remain relatively dated (2017-2023). Tables 2-3 in "Transforming memristor noises into computational innovations" (Ding et al., Commun. Mater. 2025) may help identify more recent ReRAM noise-based computing works for comparison.

Response:

Thank you for your comment. We agree that it is important to (i) transparently acknowledge the throughput/area trade-off associated with the optimized RTN sampling condition and (ii) keep the benchmarking table up to date by incorporating more recent noise-based computing works.

Regarding point (1), we have added a brief statement in the main discussion to explicitly note that the optimized sampling setting involves a latency of 36 ms and therefore requires additional parallelism for high-throughput operation, corresponding to an estimated hardware overhead of about 4,552 FTJs for 30 fps. The detailed derivation and assumptions remain in Supplementary Note 8.

Regarding point (2), we thank the reviewer for pointing us to the recent review by Ding et al. (Commun. Mater. 2025), which summarizes recent advances in memristor-noise-based generators. Based on Tables 2–3 of this review, we updated Table 1 to include a more noise-based hardware demonstration. Please refer to the revised manuscript (Discussion section and updated Table 1) to find the modifications.

Thank you again for these helpful suggestions.

Corresponding change in manuscript: Yes

Location of Change: Results – Page 21

We note that the optimized latent sampling condition uses a 36 ms sampling window per FTJ. For real-time generation at 30 frames per second with a 256-dimensional latent vector and 16 parallel bitstreams, the sampler must provide 256 × 30 × 16 samples per second, which corresponds to 122,880 samples per second. Under a 36 ms sampling window, a single FTJ provides about 27 samples per second, which implies on the order of a few thousand FTJs, about 4,552 devices dedicated to RTN sampling. This sampling overhead represents a trade-off of bitstream-based RTN sampling, and it can be reduced by algorithm-level choices such as lowering the latent dimension or reducing the number of bitstreams. For completeness, the throughput, minimum arraying for the decoder VMM, and ADC and read energy overhead under the same 30 fps target are discussed in Supplementary Note 8.

Location of Change: Table 1

								Latent Sampling			Decoder Mapping
Work	Latent Sampling	Decoder Mapping	Device type	CMOS compatibility	Array Size	Task	Implementation	Cell Area	Speed*	Power**	TOPS/W
This work	O	O	Both FTJ	O	400	MNIST, CelebA	Circuit-level	2 × 2 μm²	36 ms	282 μW (FTJ-only)	106 (FTJ & ADC)
[5]	O	-	Bi_2O_2Se	X	1 (Single Device)	MNIST	Device-level	10 × 10 - 40 × 40 μm²	10 ms	98.1-337 μW	-
[6]	O	-	ReRAM	X	1 (Single Device)	TRNG-only	Circuit-level	5 × 5 - 50 × 50 μm²	50-250 ms	1.23- 862 μW	-
[7]	O	-	ReRAM	X	1 (Single Device)	TRNG-only	Circuit-level	2.5 × 2.5 μm²	100 ms	1.47 mW	-
[8]	-	O	ReRAM	O	Only Simulation	MNIST	Simulation-level	-	-	-	4.6
[9]	-	O	1T-1R	O	1000	MNIST	Circuit-level	-	-	-	-
[10]	O	-	CMOS	O	-	TRNG	Circuit-level	0.01 mm²	11.6-312 ns	8.33-523 μW	
[11]	O	-	CMOS	O	-	TRNG	Circuit-level	0.0023 mm²	100 ps	1.2 mW	-
[12]	-	O	CMOS	O	-	Neural Networks	Commercial	-	-	-	2.3
[13]	O	-	MTJ	O	1	P-bit	Circuit-level	0.15 × 130 μm²	695 ms	20-100 μW	-

*Sampling time per bit

**Normalized for 30 fps Human Face Image (128 ×128) Generation

Tera operations per second (TOPS)

We, the authors, send the deepest appreciation for the encouragement and comments again.
